# Heparin-based hydrogel scaffolding alters the transcriptomic profile and increases the chemoresistance of MDA-MB-231 triple-negative breast cancer cells†
†Electronic supplementary information (ESI) available. See DOI: 10.1039/c9bm01481k


**DOI:** 10.1039/c9bm01481k

**Published:** 2020-02-13

**Authors:** Nidhi Menon, Ha X. Dang, Udaya Sree Datla, Maryam Moarefian, Christopher B. Lawrence, Christopher A. Maher, Caroline N. Jones

**Affiliations:** a Graduate Program in Translational Biology , Medicine and Health , Virginia Polytechnic Institute and State University , Blacksburg , VA 24061 , USA . Email: jonescn@vt.edu; b Department of Biological Sciences , Virginia Polytechnic Institute and State University , Blacksburg , VA 24061 , USA; c McDonnell Genome Institute , Washington University in St. Louis , MO 63108 , USA; d Department of Medicine , Washington University School of Medicine , St. Louis , MO 63108 , USA; e Alvin J. Siteman Cancer Center , Washington University in St. Louis , St. Louis , MO 63108 , USA; f Department of Mechanical Engineering , Virginia Polytechnic Institute and State University , Blacksburg , VA 24061 , USA; g Department of Biomedical Engineering , Washington University in St. Louis , MO 63108 , USA

## Abstract

The tumor microenvironment plays a critical role in the proliferation and chemoresistance of cancer cells. Growth factors (GFs) are known to interact with the extracellular matrix (ECM) *via* heparin binding sites, and these associations influence cell behavior. In the present study, we demonstrate the ability to define signals presented by the scaffold by pre-mixing growth factors, such as epidermal growth factor, into the heparin-based (HP-B) hydrogel prior to gelation. In the 3D biomimetic microenvironment, breast cancer cells formed spheroids within 24 hours of initial seeding. Despite higher number of proliferating cells in 2D cultures, 3D spheroids exhibited a higher degree of chemoresistance after 72 hours. Further, our RNA sequencing results highlighted the phenotypic changes influenced by solid-phase GF presentation. Wnt/β-catenin and TGF-β signaling were upregulated in the cells grown in the hydrogel, while apoptosis, IL2-STAT5 and PI3K-AKT-mTOR signaling were downregulated. With emerging technologies for precision medicine in cancer, this nature of fine-tuning the microenvironment is paramount for cultivation and downstream characterization of primary cancer cells and rare circulating tumor cells (CTCs), and effective screening of chemotherapeutic agents.

## Abstract 2

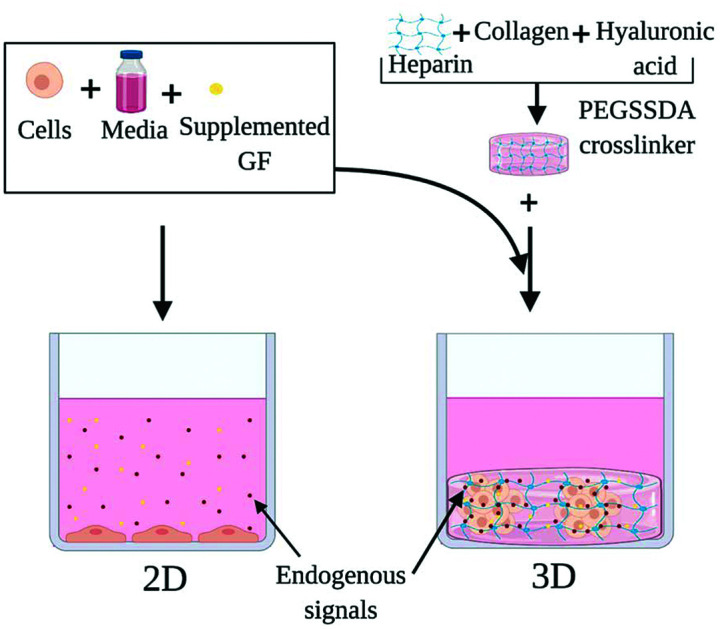
The solid-phase presentation of growth factors in heparin-based hydrogel alters the transcriptomic profile and increases the chemoresistance of MDA-MB-231 cells.

## 1. Introduction

The tumor microenvironment is a result of tumor-derived signals, interactions with the extracellular matrix (ECM) and the surrounding tissue and is known to play a critical role in tumor initiation, progression, and chemoresistance.^1^–^4^ Interactions between the cell and its extracellular environment and neighboring cells are vital for survival, growth, and differentiation.^5^ Conventional two-dimensional (2D) monolayer cultures are incapable of reproducing the characteristic features of tumors *in vivo*. While 2D cell culture experiments have contributed significantly to our understanding of cancer, they are unable to provide key insights into the features of the tumor interactions with the surrounding microenvironment. Recent statistics estimate approximately 90% of chemotherapies and immunotherapies fail when translated to *in vivo* solid tumors, despite promising *in vitro* experments.^6^

The hanging-drop method,^7^ the use of non-adhesive substrates,^8^ and orbital shaking^9^ have been previously used to form spheroids in aqueous culture. However, none of these methods encourage self-assembly, but are rather focused on external physical stimuli to encourage cell aggregation. The use of hydrogels as a biomimetic niche have shown notable changes in cell behavior.^10^–^17^ While previous studies have introduced novel natural and synthetic materials for controlling biological and mechanical properties, they are limited in their molecular characterization of tumor cells in their microenvironment. Moreover, there is minimal control over GF presentation and delivery, which is essential for optimal cell response.^18^ In contrast, hyaluronic acid and heparin-based hydrogels have proven to be potent scaffolds for primary cell culture and mimic the slow, controlled release of GFs *in vivo.*^18^–^27^ Epidermal growth factor (EGF) plays a vital role in controlling breast tumor cell growth and differentiation. Overexpression of the EGF receptor (EGFR) has been well documented in breast carcinogenesis.^28^ In order to test the capacity of the HP-B hydrogel system for cultivation of breast tumor cells, EGF was used to study the strength of heparin as a GF binding moiety. Furthermore, EGF stimulation was used as a model for studying the differences between localized stimulation using HP-B hydrogel *versus* aqueous stimulation in growth media. Identifying the genotypic and phenotypic differences between 2D cultures and 3D spheroid cultures are essential for studying tumor response to drugs and to further scrutinize underlying mechanisms conferring adaptive resistance to tumors *in vivo*.

**Fig. 1:**
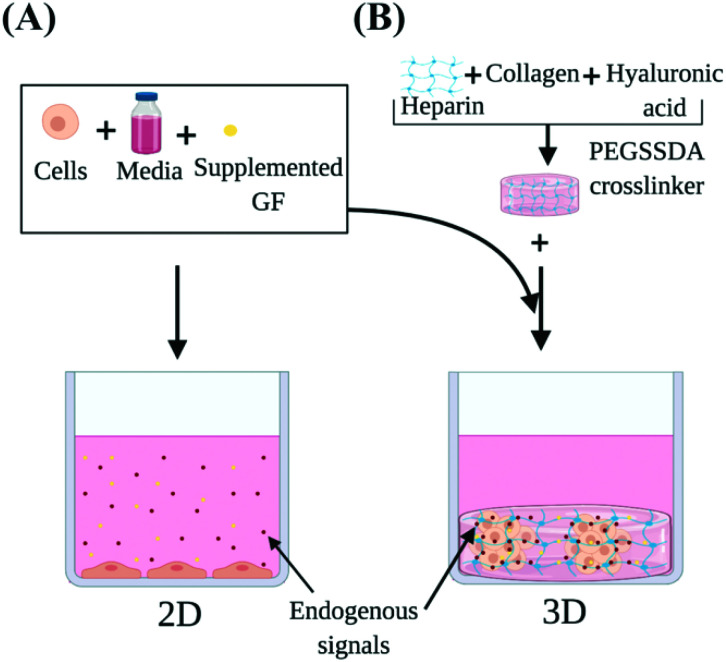
**Heparin-based (HP-B) hydrogel for 3D cell culture *vs.* standard 2D culture in a glass-bottom dish**. In this study, we demonstrate that HP-B hydrogel promotes spheroid formation in breast cancer cells and significantly alters the transcriptomic profile and phenotype of cells compared to standard culture conditions. (A) The presentation of growth factors and endogenous signals in aqueous media cannot be precisely controlled. The cells are adherent to the surface, have a flattened morphology and form a 2D monolayer. (B) Heparin-based hydrogel acts as a biomimetic tumor niche and promotes solid-phase presentation of growth factors and endogenous signals secreted by the cells, as depicted. The presence of extracellular matrix proteins encourages self-assembly into a spheroid morphology.

Our study investigated the use of HP-B hydrogel as a biomimetic scaffold for the solid-phase presentation of EGF as well as GFs and endogenous signaling molecules (Fig. 1). A triple-negative breast cancer (TNBC) cell line (MDA-MB-231) was used as a model. Due to a lack of targeted therapies for this aggressive breast cancer subtype, chemotherapy remains the standard source of treatment. Paclitaxel promotes mitotic arrest and cell death by binding to microtubules and has shown promising results in the triple-negative cohort.^29^ Therefore, Paclitaxel was used to study the chemoresistance in these cells. Cells were grown in four conditions; (1) control in a glass dish, (2) EGF supplemented in solution (EGF (aq)) in a glass dish, (3) HP-B hydrogel and (4) HP-B hydrogel pre-mixed with EGF in solid-phase (HP-B hydrogel + EGF (s)). Viability, proliferation and chemoresistance to paclitaxel were quantified. Additionally, to quantify the genotypic and phenotypic changes observed from sequestration and presentation of solid-phase GFs to breast tumor cells, RNA Sequencing was carried out on cells grown in the conditions mentioned above. This study shows that the microenvironment significantly alters the transcriptomic profile of MDA-MB-231 and increases chemoresistance to paclitaxel.

PI3K-AKT-mTOR signaling is one of the most commonly targeted pathways for cancer therapy. However, a study highlighted the aberrant changes in Wnt/β-catenin signaling in response to PI3K inhibitors and implies crosstalk between the two signaling pathways to confer resistance to PI3K inhibitors. Furthermore, dual inhibition of PI3K and Wnt signaling pathways *in vivo* and *in vitro* had a higher synergistic effect compared to inhibition of PI3K alone.^30^ Tumor Necrosis Factor Alpha (TNFA) signaling *via* NF-κB is another multifunctional pro-inflammatory pathway that has been known to negatively affect EGFR activation.^31^ Another important pathway, TGF-beta signaling, has been studied extensively in solid-tumors. It is known to function as a potent immunosuppressor, affecting normal lymphocyte proliferation and maturation in the tumor microenvironment.^32^,^33^ IL2-STAT5 signaling is also critical for T-cell development and IL2 has been approved for cancer immunotherapy in treating metastatic renal cell carcinoma and metastatic melanoma.^34^ IL2 suppression is also known to be mediated by TGF-beta.^35^ Our results highlight changes in these cancer hallmark pathways, observed in the different microenvironments. HP-B hydrogel is a promising biomimetic scaffold, critical for initial screening and successful translation of novel, targeted therapies to overcome resistance in triple-negative breast cancer.

## 2. Materials and methods

### 2.1. MDA-MB-231 cell culture

Triple-negative breast cancer cell line MDA-MB-231 (American Type Cell Culture HTB-26) was cultured in Gibco™ Dulbecco's Modified Eagle's Medium F-12 (DMEM/F-12) containing high glucose and GlutaMAX™, supplemented with 10% fetal bovine serum (FBS) and 100 units per ml penicillin and 0.1 mg ml^–1^ streptomycin. The cells were maintained in vented T-25 or T-75 flasks (corning) at 37 °C and 5% CO_2_. Media was changed every 48 hours. Using 0.05% Trypsin-EDTA for detachment, cells were harvested for experiments at 80–90% confluency.

### 2.2. Heparin hydrogel preparation

HyStem-HP Hydrogel Kit with PEGSSDA (ESI BIO GS315P) was used to prepare heparin hydrogel with a collagen I background. The kit is comprised of lyophilized solids of Heprasil® (thiol-modified sodium hyaluronate with thiol-modified heparin), Gelin-S® (thiol-modified gelatin), PEGSSDA™ (disulfide containing polyethylene glycol diacrylate), and degassed deionized water (DG Water). All vials are allowed to reach room temperature. Heprasil® and Gelin-S® are reconstituted in DG water to form 1% (w/v) solution, and PEGSSDA™ is reconstituted in DG water to form 2% (w/v) solution. Heprasil® and Gelin-S® vials are placed horizontally in a shaker for 40 minutes for complete dissolution. Solutions of Heprasil® and Gelin-S® are mixed in a 1 : 1 volume ratio. PEGSSDA™ works as a crosslinker and is mixed in a 1 : 4 volume ratio with the Heprasil® and Gelin-S® mixture to initiate gelation. Time for complete gelation is dependent on the volume and surface area occupied by the gel and is approximately 4 hours per ml.

### 2.3. Measurement of EGF retention in HP-B hydrogel

HP-B hydrogel was pre-mixed with 50 ng ml^–1^ EGF. 1.5 mL of this pre-mixed hydrogel was added to a 12-well plate (*n* = 3) and allowed to gelate for about 4 to 5 hours at room temperature. Following this, 1.5 mL of serum-free, EGF-free growth media was added on top of the hydrogel in each of the wells and incubated at 37 °C. The supernatant was collected, and the wells were replenished with 1.5 ml of fresh serum-free, EGF-free media every 24 hours for a total of 72 hours. The collected supernatants were analyzed for total EGF content, using EGF Human ELISA kit (Invitrogen, KHG0061). Previous studies have validated a normalization factor to account for the degradation of the protein in heparin-based hydrogel, that was used to post-process our data from ELISA.^36^

### 2.4. Simulation of EGF retention in HP-B hydrogel

COMSOL Multiphysics® 5.2 was used to simulate the diffusion of EGF through the heparin hydrogel porous media and determine heparin-EGF binding. To reflect the experimental setup, the dimensions of the 1.5 ml hydrogel and media were based on the 12-well plate. The diffusion coefficient of EGF in water (*D*_EW_) was calculated using the semiempirical eqn (1) of Polson^37^ shown below. The diffusion coefficient was corrected for heparin-EGF binding (*D*_EH_) using eqn (2) governing diffusion when protein binding is present.^37^ Fick's first law of diffusion, inbuilt in the software was used to run the simulation, taking into considering the porosity (69%) of the gel.1
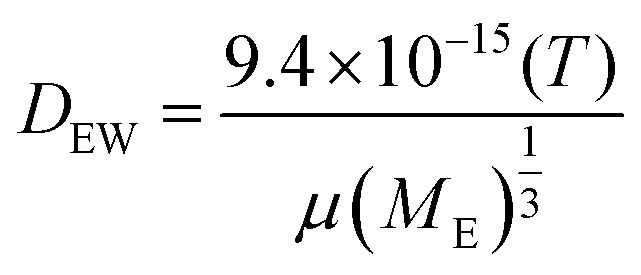
wherein *T* (37 °C) is the temperature, *μ*(6.913 × 10^–4^ Pa s) is the viscosity of water and *M*_E_ (6.2 kDa) is the molecular weight of EGF.2
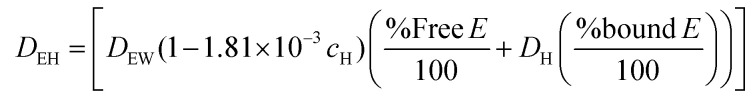
wherein *c*_H_ is the concentration of heparin and hyaluronic acid (4 kg m^–3^), *D*_H_ is the diffusion coefficient of heparin, assumed to be 0 in the hydrogel, due to the crosslinking of the matrix.

### 2.5. Characterization of viability and chemoresistance

Cells were grown in 96-well plates (Cellvis P96-1.5H-N) (*n* = 3) with a low seeding density of ∼1000 cells per well. Cells were grown in four different conditions: (1) 100 μl of complete growth media (control), (2) 100 μl of complete growth media supplemented with 50 ng ml^–1^ EGF (Sigma E9644) in solution (EGF (aq)), (3) 50 μl HP-B hydrogel topped with 100 μl of complete growth media (HP-B hydrogel), (4) 50 μl HP-B hydrogel pre-mixed with 50 ng ml^–1^ EGF (HP-B hydrogel + EGF(s)). Media (100 μl) was replenished every 24 hours. At the end of 72 hours, a live and dead cell assay (Abcam, ab115347) was performed to assess the viability of cells. In order to quantify chemoresistance, cells were allowed to grow for 48 hours prior to the addition of 100 μl of 100 nM paclitaxel. The cells were treated for 24 hours and images were taken at the 72-hour mark using the live and dead cell assay. Images of live (labeled green) and dead (labeled red) cells were taken on the Zeiss LSM 880 confocal microscope at 40× magnification. A manual cell counter on the open source software ImageJ/Fiji^38^ was used to count the live and dead cells in the image frame. ANOVA and Tukey's honest significant difference was used to compare the mean percentage of dead cells in all the groups.

### 2.6. Proliferation assay

MDA-MB-231 cells were cultured on cover slips (1.5, 0.16 mm) embedded in 12-well plates (MatTek) (*n* = 3) for immunohistochemistry experiments. The cells were grown in the same four aforementioned conditions. The coverslip was coated with 50 μl of the hydrogel for the two HP-B hydrogel samples. Cells were seeded at ∼1000 cells per well. Media was changed every 24 hours and cells were allowed to grow for 72 hours. Cells were fixed with 4% formaldehyde (Sigma 818708) and permeabilized using 0.5% Triton™ X-100 (Sigma ×100). 1% BSA was used as the blocking solution and the specimens were incubated for 30 min. Samples were then incubated overnight at 4 °C with primary rabbit monoclonal anti-Ki-67 (abcam ab16667). Cells were then washed with PBS before incubating with the secondary goat anti-rabbit IgG H&L conjugated with Alexa Fluor® 488 (abcam ab150077) for 1 hour in the dark. Images were taken using the Zeiss confocal microscope (Zeiss LSM 880) at 40× magnification. ANOVA and Tukey's honest significant difference was used to compare the mean percentage of Ki-67 stained cells in all the groups.

### 2.7. RNA sequencing

Cells were grown in 12-well plates (*n* = 2) with a density of 2.5 × 10^4^ cells per well in 1.5 ml of complete growth media. Cells were grown in the same aforementioned conditions listed in 2.5, except 1.5 ml of the hydrogel was used as a platform for the wells subjecting cells to HP-B hydrogel. Media was replenished every 24 hours. Total RNA was isolated from cells after allowing the cells to grow for 72 hours. For cells grown on the plate, cells were retrieved using trypsin whereas for cells encapsulated within the hydrogel, liquid nitrogen was used to flash freeze the gel and a mortar and pestle were used to powder the frozen gel. All samples were then processed with Trizol reagent (Life technologies 15596026). Chloroform (Fisher BP1145-1) was added for phase separation, which was carried out using centrifugation at 12 000*g* at 4 °C for 5 minutes. The aqueous phase was then processed using the RNeasy mini kit (Qiagen 74104) according to the manufacturer's instructions. Eluted RNA was stored in –80 °C. Quality of total RNA was checked on Agilent Tapestation 2200 (Agilent Technologies, Santa Clara CA). 100 ng of total RNA was processed with New England Biolabs (NEB) Next rRNA Depletion Kit (NEB E6310X) to produce rRNA depleted RNA for RNA sequencing. RNA Seq Library preparation was performed with NEBNext Ultra II Directional RNA Library Prep Kit for Illumina (NEB E7760L) and individual samples/libraries were indexed separately using the NEBNext Multiplex Oligos for Illumina (NEB E6609S) for sequencing on the Illumina NextSeq 500/550 High Output kit V2 (75 cycles) (P/N FC-404-2005) to 1 × 75 (400 million clusters). 13 cycles of PCR enrichment were used to amplify Adapter Ligated DNA. The 300 bp libraries generated are validated using Agilent 2200 Tapestation and quantitated using Quant-iT dsDNA HS Kit (Invitrogen, Q33120) and qPCR. The Illumina NextSeq Control Software v2.1.0.32 (; http://illumina.com) with Real-Time Analysis RTA v2.4.11.0 was used to provide the management and execution of the NextSeq 500 and to generate BCL files. The protocol is outlined in the schematic in ESIFig. S1.†

### 2.8. RNA sequencing data analysis

We obtained ∼21.3 million reads per sample. Raw RNA-Seq reads were trimmed to remove adaptors and demultiplexed using bcl2fastq Conversion Software v2.20 (http://illumina.com). Reads were aligned to the human reference genome Grch38/hg38 using Hisat2^39^ and Bowtie2.^40^ Average alignment rate was ∼95%. FeatureCounts^41^ was used to count reads aligned to genes using Gencode annotation v27.^42^ Raw read counts were used for subsequent TMM normalization and differential expression analysis using negative binomial model with edgeR v3.22,3.^43^ False discovery rate (FDR) was estimated to correct for multiple hypothesis test using Benjamini–Hochberg procedure. Normalized gene expression was used in gene set enrichment analysis (GSEA)^44^ to identify cancer hallmark pathways/gene signature altered in EGF (aq), HP-B hydrogel, and HP-B hydrogel + EGF (s) compared with the control group. All statistical analysis and visualization were performed using the R software package v3.5.1.^45^

## 3. Results

### 3.1. HP-B hydrogel for long-term, solid-phase EGF presentation

**Fig. 2:**
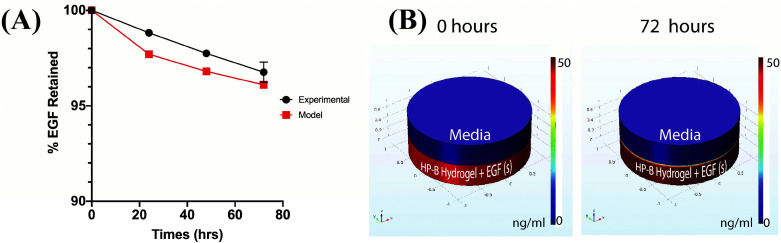
**Quantification of EGF release from HP-B hydrogel**. (A) Results from ELISA demonstrate the high retention of EGF in the HP-B hydrogel and this is confirmed by the protein-binding diffusion model. Over 95% of the initial EGF is retained at the end of 72 hours. (B) COMSOL multiphysics graphs of the diffusion model illustrate the minimal release of EGF observed after 72 hours.

The cumulative percentage release of EGF was less than 5% of the initial bound EGF over a period of 3 days. 96.73 ± 0.53% of the EGF was retained in the HP-B hydrogel over 72 hours (Fig. 2(A)). Furthermore, to calculate the heparin-EGF binding, a COMSOL model was used to simulate the system (refer to methods for COMSOL Simulation) (Fig. 2(B)). A 99.5% or higher binding rate corroborated the experimental data and resulted in 96% of the EGF being retained over the 72-hour period. The time-lapse simulation demonstrates the high retention of EGF from HP-B hydrogel (Video 1†).

### 3.2. HP-B hydrogel induces spheroid formation in breast cancer cells and keeps them more viable

**Fig. 3:**
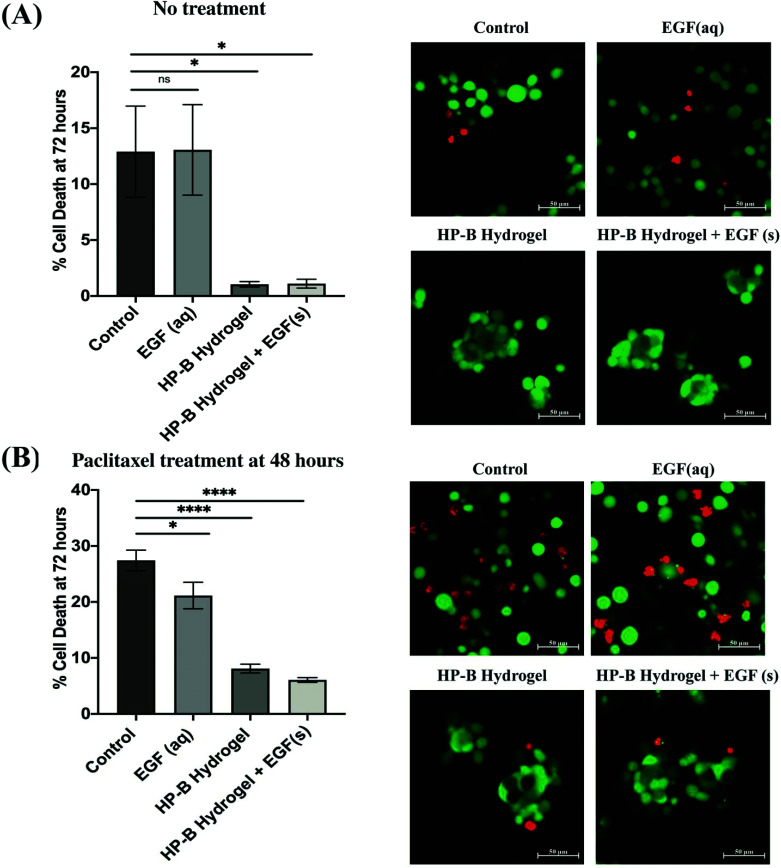
**Heparin-based (HP-B) hydrogel increases viability and chemoresistance**. (A) The percentage of dead cells after 72 hours was significantly (*p < 0.05) higher in both control and aqueous EGF conditions compared to HP-Bhydrogels, both with and without EGF. (B) A 24-hour treatment with paclitaxel prior to the live and dead cell assay at 72 hours increased the percentage of dead cells in all the conditions. However, the difference was significantly higher (*p < 0.05, ****p < 0.0001) in both 2D conditions compared to the HP-B hydrogels. Data is represented as mean ± standard error of mean (SEM).

MDA-MB-231 cells in the hydrogel formed multicellular (<100 cells) spheroids (∼100 μm) within 24 hours of initial seeding. The cell viability was measured at 72 hours using a live and dead cell assay (Fig. 3(A)). Cell viability in 2D showed a higher variability of viability. At 72 hours, cells in the control condition had 12.9 ± 4.074% dead cells whereas cells encapsulated in the HP-B hydrogel had only 1.048 ± 0.24% dead cells. Cells supplemented with aqueous EGF had 13.06 ± 4.046% dead cells unlike the cells grown in HP-B hydrogel with solid-phase EGF, which had a 1.107 ± 0.39%. Both hydrogel groups had a significant improvement in viability (*p < 0.05) compared to the control and supplemented EGF (aq) conditions.

### 3.3. 3D cell culture increases chemoresistance in breast cancer cells

MDA-MB-231 cells were treated with paclitaxel [100 nM] at 48 hours post initial seeding and viability was assessed at 72 hours (Fig. 3(B)). Cells in the control group had 27.40 ± 1.84% dead cells whereas EGF (aq) supplemented cells had 21.14 ± 2.38% dead cells. However, cells in the HP-B hydrogel had only 8.1 ± 0.79% dead cells and cells HP-B hydrogel + EGF(s) had 6.08 ± 0.42%. The percentage of dead cells in 3D morphology was significantly (****p < 0.0001) lower than in 2D.

### 3.4. HP-B hydrogel reduces cell proliferation

**Fig. 4:**
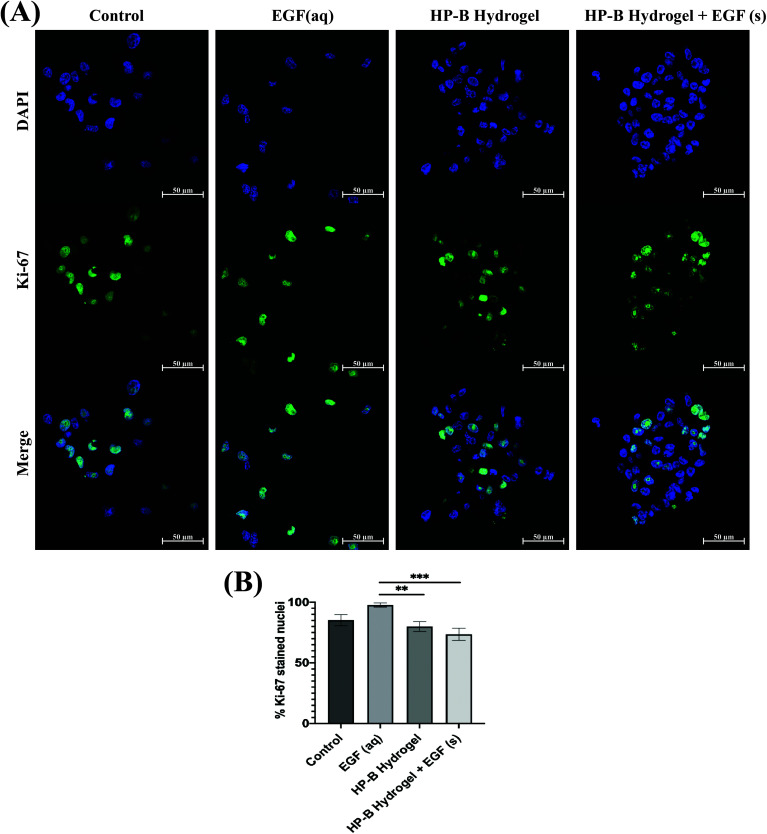
**Heparin-based (HP-B) hydrogel decreases breast cancer cell proliferation**. (A) Cells were stained for Ki-67 after 72 hours of cell culture. (B) Interestingly, the percentage of Ki-67 positive cells were fewer in 3D spheroids. Aqueous EGF stimulation significantly (**p < 0.005, ***p < 0.0005)" increased proliferation compared to the other conditions. Data is represented as mean ± SEM.

Proliferating MDA-MB-231 cells were determined by calculating the percentage of Ki-67 stained nuclei (Fig. 4). Cells in control had 85.17 ± 4.2% Ki-67 positive nuclei compared to 97.75 ± 1.54% with EGF (aq) stimulation. 3D cultures induced lower proliferation rates in MDA-MB-231 cells. HP-B hydrogel encapsulated cells had 79.97 ± 3.68% proliferating cells, significantly (**p < 0.005) lower than EGF (aq) stimulated cells. Similarly, HP-B hydrogel with EGF(s) was significantly (***p < 0.0005) lower than EGF (aq) stimulated cells with only 73.56 ± 4.31% proliferating cells.

### 3.5. Heparin hydrogel alters the transcriptomic profile of human breast cancer cells

**Fig. 5:**
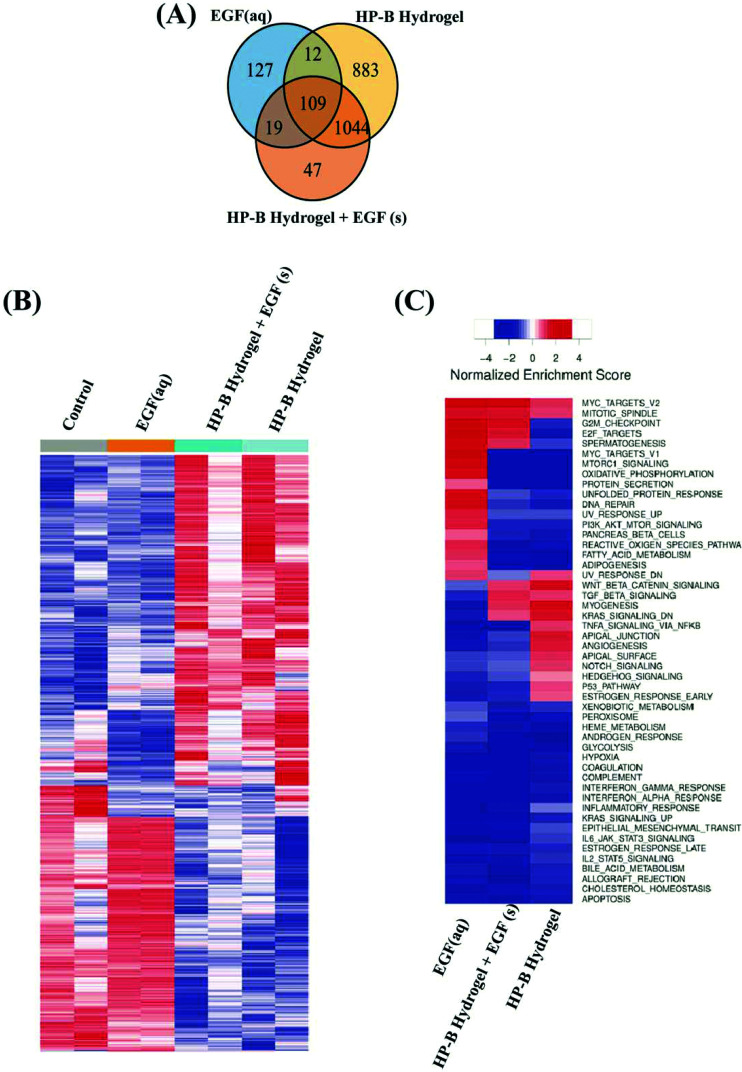
**Differential gene expression between 2D *versus* 3D heparin hydrogel cultivated breast cancer cells**. (A) The Venn diagram represents the differentially expressed genes in the culture conditions compared to the control group. The highest changes were observed in the 3D hydrogel environments. (B) Heat map shows gene expression in the four different groups. The control and EGF (aq), or the cells grown on a dish are aligned similarly with slightly stronger expression in the EGF (aq) samples. Likewise, cells grown on HP-B hydrogel with and without solid-phase EGF showed similar alignment reads. (C) Heatmap shows normalized enrichment scores for cancer hallmark gene sets. Critical pathways are significantly altered with aqueous and solid-phase presentation.

**Fig. 6:**
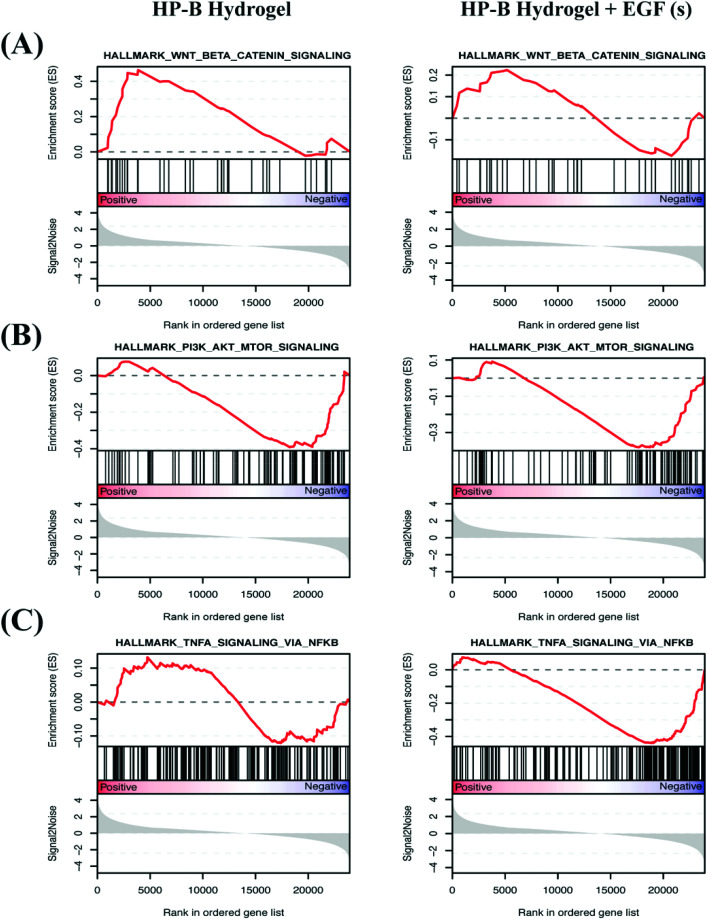
**Enrichment plots of differential expression of hallmark pathways**. Normalized gene expression was used in gene set enrichment analysis (GSEA) to identify cancer hallmark pathways/gene signature altered in EGF (aq), HP-B hydrogel, and HP-B hydrogel + EGF (s) compared with the control group. Vertical black lines on the central *x*-axis correlate with the genes from the pathway and is correspondingly mapped to the dataset. Genes that are positively correlated with the phenotype are on the left (indicated by red), whereas genes that are negatively correlated with the phenotype are on the right (indicated by blue). The degree of positive and negative correlation to the phenotype is determined by where it ranks on the ordered gene list. The enrichment score records hits from the sample to the specific phenotype and the more the number of genes encountered in the sample, the higher the enrichment score. (A) Wnt/β-catenin signaling is upregulated in HP-B hydrogel (*p < 0.05) as well as HP-B hydrogel with EGF(s). (B) PI3K-AKT-mTOR signaling is interestingly downregulated (*p < 0.05) in both HP-B hydrogel conditions. (C) TNFA signaling *via* NF-κB is influenced by the presence of EGF(s) stimulation and is downregulated (**p < 0.005) *versus* upregulated in HP-B hydrogel without supplemented EGF(s).

We identified differentially expressed genes in cells grown with EGF (aq) stimulation (267 genes), HP-B hydrogel (2048 genes) and HP-B hydrogel grown with pre-mixed EGF(s) stimulation (1219 genes) compared to the control group (Fig. 5(A)). Cells grown under the two hydrogel conditions showed significantly different gene expression profiles compared to cells grown under the EGF (aq) and control (Fig. 5(B)). To further investigate the signaling pathways affected by the growth conditions, we performed Gene Set Enrichment Analysis (GSEA) comparing all growth conditions with the control. GSEA was run using the Hallmark gene sets in the Molecular Signature Database (MSigDB). This particular gene set allows for a robust analysis of the most relevant gene expression changes and relates them to specific biological processes. This better facilitates follow-up analysis in the context of the disease phenotype.^46^ We identified prominent Hallmark pathways influenced by aqueous EGF stimulation and presence of a 3D microenvironment and solid-phase GF presentation (Fig. 5(C)). Some of these critical pathways are highlighted in Table 1. Interestingly, the PI3K-AKT-mTOR signaling pathway was downregulated in both the hydrogel conditions (Fig. 6(A)). The results corroborate previous studies reporting similar downregulation of the PI3K-AKT-mTOR pathways when cells are grown as 3D spheroids.^47^ However, AKT1, involved in several signaling pathways regulating cell proliferation, survival and metabolism was upregulated in the hydrogel and PTEN, known to function as a tumor suppressor and promote apoptosis had lower expression levels on both the hydrogel platforms (Fig. S2(A)†). Furthermore, canonical Wnt/β-catenin signaling was upregulated in the cells grown in HP-B hydrogel and HP-B hydrogel + EGF (s) (Fig. 6(B)). Transcription factors LEF1 and TCF7, downstream mediators of the canonical Wnt/β-catenin signaling pathway were upregulated in the cells grown in the 3D environment (Fig. S2(B)†).^48^,^49^ TNFA signaling *via* NF-κB, however, was downregulated with both aqueous and solid-phase EGF presentation but upregulated in HP-B hydrogel (Fig. 6(C)). NF-κB and TNF mRNA levels were also higher in the control and HP-B hydrogel cultures without EGF stimulation, both aqueous and solid (Fig. S2(C)†).

**Table 1:** **Enriched cancer hallmark gene signature in breast cancer cells grown with supplemented aqueous EGF, HP-B hydrogel and HP-B hydrogel with solid-phase EGF compared to control**. Pathways are significantly (*p < 0.05, **p < 0.005, ***p < 0.0005) altered with and without solid-phase GF presentation

Hallmark pathways	EGF (aq)	HP-B hydrogel	HP-B hydrogel + EGF (s)
PI3K-AKT-mTOR signaling	Upregulated	Downregulated*	Downregulated*
Wnt beta-catenin signaling	Downregulated	Upregulated*	Upregulated
TNFA signaling pathway *via* NF-κB	Downregulated**	Upregulated	Downregulated**
IL-2 STAT5 signaling	Downregulated	Downregulated***	Downregulated***
TGF-beta	Downregulated	Upregulated	Upregulated
Apoptosis	Downregulated**	Downregulated***	Downregulated***
Oxidative phosphorylation	Upregulated***	Downregulated***	Downregulated***

Both HP-B hydrogel environments also downregulated IL2-STAT5 signaling pathway (Fig. S3(A)†) and upregulated TGF-beta signaling (Fig. S3(B)†). The full GSEA for the datasets is available in the ESI (File S4†).

## 4. Discussion

HP-B hydrogel enables controlled presentation of GFs and endogenous signals and can serve as an effective biomimetic *in vitro* microenvironment for the culture of breast cancer cells. The EGF diffusion study in the HP-B hydrogel was able to confirm the association and dissociation between the GFs and heparin chains that limited the free diffusion of EGF in the pores of the hydrogel. This kind of a slow and sustained release of GFs from the HP-B hydrogel matrix bio-mimics the release of GFs from ECM *in vivo*,^50^ unlike traditional cell culture where the cells are directly exposed to a high initial concentration of the GFs in soluble form in the media. The spatiotemporal modulation of GFs not only keeps cells more viable but also helps differentiation (tissue-specific). Despite fewer proliferating cells in 3D spheroids, cells remained significantly more viable. Consistent with previous studies, drug resistance can be attributed to cells entering and exiting dormancy in 3D cultures to evade paclitaxel toxicity.^51^ Several of the transcriptomic changes influenced by microenvironmental stimulus could be responsible for this phenomenon and the high degree of chemoresistance observed in 3D spheroids.

EGF stimulation in solid-phase revealed a phenotypic profile that differs significantly from EGF stimulation in an aqueous phase. HP-B hydrogel with no EGF stimulation also revealed changes in cell behavior compared to the control group. The 3D environment specifically showed changes in critical pathways regulating cancer progression and metastasis. The gene expression changes seen in the key regulators of PI3K-AKT-mTOR and Wnt/β-catenin signaling pathways convey the importance of studying biomimetic platforms and their influence on the molecular biology of cells. A previous study demonstrated that inhibition of the PI3K-AKT-mTOR in MDA-MB-231 increased Wnt/β-catenin signaling.^30^ However, the molecular mechanisms conferring adaptive resistance to tumors *in vivo* have not been fully understood. Our results from RNA sequencing revealed similar trends, wherein PI3K-AKT-mTOR signaling was downregulated, and Wnt/β-catenin signaling was upregulated in the 3D environment. Although, cells grown on the dish with aqueous GF presentation showed the opposite trend. A low cell seeding density and interactions with the ECM environment may be responsible for the switch in the phenotype of the cells grown in the hydrogel environment in this short period of time. Despite the downregulation of the PI3K-AKT-mTOR signaling pathway, cells in the 3D environment had significantly higher viability and adaptive resistance to chemotherapy compared to cells grown in 2D, including the EGF (aq) stimulated cells.

Our study also highlights the importance of specific GF stimulations in the local tumor niche. EGF stimulation in both aqueous and solid-phase led to the downregulation of TNFA signaling *via* NF-κB. Recent studies suggest a cross-talk between EGFR and TNFA.^52^ Another study observed the inhibition of EGFR in lung cancer cells increased TNFA levels as an adaptive response.^53^ Further scrutinizing the crosstalk between signaling pathways, specifically in response to their environmental signals in essential to avoid failure of novel drug contenders in clinical testing. Immune-regulating pathways such as IL2-STAT5 signaling were downregulated in both 3D environments, whereas TGF-beta signaling was upregulated. TGF-beta signaling has been studied extensively in solid-tumors and is known to function as a potent immunosuppressor, affecting normal lymphocyte proliferation and maturation in the tumor microenvironment.^32^,^33^ As aforementioned, IL2-STAT5 signaling plays a crucial role in T-cell development and IL2 has been approved for cancer immunotherapy in treating metastatic renal cell carcinoma and metastatic melanoma.^34^ IL2 suppression is also known to be mediated by TGF-beta.^35^ Therefore, the mechanisms underlying immune evasion *in vivo* need to be studied thoroughly to avoid failure of promising immunotherapies. Cocktail therapies may be the key to treating tumors for successful remission.

Our results reaffirm that the rewiring of pathways during cancer proliferation, metastasis and colonization are highly dependent on the local niche.^54^ The use of HP-B hydrogel allows for manual fine-tuning of the microenvironment that can be modulated for tissue-specific cell responses.^20^ Our study emphasizes the need to use more sophisticated 3D platforms to study tumor dynamics *in vitro* to better represent the interactions between tumor cells and their microenvironment. In the future, HP-B hydrogel can serve as a platform for co-cultures based on the tissue of interest. Studies including organ specific stromal cells may be essential to further validate the need for heparin incorporation to control autocrine and paracrine signaling. This is especially important when evaluating the effects of chemotherapy and immunotherapy *in vitro* to avoid the variability observed between pre-clinical models and clinical trials.^55^ The scaffold has applications in the cultivation of low numbers of primary cells and CTCs, as demonstrated by relatively low seeding densities used in our experiments.

Additionally, heparin, in particular, is often administered as an anticoagulant to cancer patients receiving chemotherapy.^56^ Heparin-induced thrombocytopenia in cancer patients has been previously documented.^57^ The side-effects of heparin, in the context of changes in the transcriptomic profile, have not been well studied. Results from our study have highlighted that protein-binding moieties, such as heparin and hyaluronic acid can alter the phenotypic profile of cells, leading to increased chemoresistance. Conflicting reports currently exist on whether heparin affects overall survival of patients.^58^–^60^ Our study further stresses on the need for long-term studies on the influence of heparin content in the tumor microenvironment and its influence on chemoresistance.

## 5. Conclusions

Heparin-based hydrogel was a suitable scaffold for solid-phase epidermal growth factor presentation and to support the formation of breast cancer cell spheroids. Furthermore, MDA-MB-231 cells cultured in HP-B hydrogel were shown to proliferate less and exhibit a higher degree of chemoresistance compared to cells cultured in standard dishes. RNA Seq data illustrates the dramatic differences in transcriptomic profiles of human breast cancer cells cultured in HP-B hydrogel driving these phenotypic changes. This study shows that cell culture biomaterial designs are critical when running *in vitro* assays, including drug screening tests. Future studies are now required to investigate the effects of different growth factors presented *via* HP-B hydrogels on the survival, differentiation and proliferation of cells.

## Conflicts of interest

There are no conflicts to declare.

## Supplementary Material

Supplementary movie

Supplementary information

Supplementary information
