# Supplementary material for: Heparin-based hydrogel scaffolding alters the transcriptomic profile and increases the chemoresistance of MDA-MB-231 triple-negative breast cancer cells
Source: Biomater Sci. 2020 Feb 13;8(10):2786–96. doi: 10.1039/c9bm01481k (PMC7497406; doi:10.1039/c9bm01481k)
Supplement: Supplementary file 2 [file BM-008-C9BM01481K-s002.zip › Supplementary File 4/EGFvControl/Pathways/my_analysis.Gsea.1545200981068/HALLMARK_PI3K_AKT_MTOR_SIGNALING.html]

Details for gene set HALLMARK\_PI3K\_AKT\_MTOR\_SIGNALING[GSEA]

|  || Dataset | expr.class.cls#EGF\_versus\_CONTROL.class.cls#EGF\_versus\_CONTROL\_repos |
| Phenotype | class.cls#EGF\_versus\_CONTROL\_repos |
| Upregulated in class | EGF |
| GeneSet | HALLMARK\_PI3K\_AKT\_MTOR\_SIGNALING |
| Enrichment Score (ES) | 0.30627334 |
| Normalized Enrichment Score (NES) | 1.2537254 |
| Nominal p-value | 0.1266892 |
| FDR q-value | 0.15720987 |
| FWER p-Value | 0.962 |
Table: GSEA Results Summary

  

Fig 1: Enrichment plot: HALLMARK\_PI3K\_AKT\_MTOR\_SIGNALING      
 Profile of the Running ES Score & Positions of GeneSet Members on the Rank Ordered List

  

| PROBE | DESCRIPTION (from dataset) | GENE SYMBOL | GENE\_TITLE | RANK IN GENE LIST | RANK METRIC SCORE | RUNNING ES | CORE ENRICHMENT || 1 | MAP2K3 | na |  |  | 282 | 1.978 | 0.0190 | Yes |
| 2 | CAMK4 | na |  |  | 952 | 1.529 | 0.0100 | Yes |
| 3 | EGFR | na |  |  | 1241 | 1.429 | 0.0193 | Yes |
| 4 | E2F1 | na |  |  | 1340 | 1.395 | 0.0380 | Yes |
| 5 | PRKAG1 | na |  |  | 1436 | 1.368 | 0.0563 | Yes |
| 6 | PRKAR2A | na |  |  | 1476 | 1.353 | 0.0774 | Yes |
| 7 | UBE2N | na |  |  | 1878 | 1.251 | 0.0777 | Yes |
| 8 | SFN | na |  |  | 1900 | 1.246 | 0.0978 | Yes |
| 9 | CDK2 | na |  |  | 2311 | 1.153 | 0.0961 | Yes |
| 10 | RALB | na |  |  | 2730 | 1.071 | 0.0924 | Yes |
| 11 | PPP2R1B | na |  |  | 2761 | 1.067 | 0.1091 | Yes |
| 12 | ATF1 | na |  |  | 2879 | 1.047 | 0.1208 | Yes |
| 13 | MAPKAP1 | na |  |  | 2891 | 1.046 | 0.1381 | Yes |
| 14 | CDK1 | na |  |  | 2921 | 1.040 | 0.1543 | Yes |
| 15 | PFN1 | na |  |  | 3018 | 1.025 | 0.1667 | Yes |
| 16 | EIF4E | na |  |  | 3033 | 1.021 | 0.1834 | Yes |
| 17 | MAPK8 | na |  |  | 3070 | 1.012 | 0.1988 | Yes |
| 18 | ACTR2 | na |  |  | 3112 | 1.004 | 0.2137 | Yes |
| 19 | MAPK9 | na |  |  | 3209 | 0.985 | 0.2255 | Yes |
| 20 | IRAK4 | na |  |  | 3360 | 0.960 | 0.2340 | Yes |
| 21 | YWHAB | na |  |  | 3490 | 0.938 | 0.2433 | Yes |
| 22 | AP2M1 | na |  |  | 3681 | 0.907 | 0.2488 | Yes |
| 23 | RAF1 | na |  |  | 3823 | 0.880 | 0.2564 | Yes |
| 24 | PRKAA2 | na |  |  | 4005 | 0.850 | 0.2614 | Yes |
| 25 | CLTC | na |  |  | 4040 | 0.844 | 0.2741 | Yes |
| 26 | PTPN11 | na |  |  | 4074 | 0.840 | 0.2866 | Yes |
| 27 | CXCR4 | na |  |  | 4387 | 0.794 | 0.2839 | Yes |
| 28 | GRB2 | na |  |  | 4705 | 0.748 | 0.2800 | Yes |
| 29 | CDK4 | na |  |  | 4801 | 0.730 | 0.2875 | Yes |
| 30 | ACTR3 | na |  |  | 4821 | 0.726 | 0.2989 | Yes |
| 31 | NFKBIB | na |  |  | 4914 | 0.717 | 0.3063 | Yes |
| 32 | HRAS | na |  |  | 5618 | 0.623 | 0.2801 | No |
| 33 | ACACA | na |  |  | 6232 | 0.536 | 0.2572 | No |
| 34 | TBK1 | na |  |  | 6327 | 0.523 | 0.2612 | No |
| 35 | MAPK10 | na |  |  | 6331 | 0.523 | 0.2699 | No |
| 36 | NOD1 | na |  |  | 6548 | 0.496 | 0.2671 | No |
| 37 | GSK3B | na |  |  | 6707 | 0.479 | 0.2670 | No |
| 38 | DDIT3 | na |  |  | 6794 | 0.469 | 0.2705 | No |
| 39 | ITPR2 | na |  |  | 6897 | 0.456 | 0.2729 | No |
| 40 | TRAF2 | na |  |  | 7139 | 0.428 | 0.2676 | No |
| 41 | MAPK1 | na |  |  | 7252 | 0.414 | 0.2688 | No |
| 42 | MAP3K7 | na |  |  | 7759 | 0.355 | 0.2484 | No |
| 43 | RPTOR | na |  |  | 7901 | 0.336 | 0.2468 | No |
| 44 | ARHGDIA | na |  |  | 8010 | 0.324 | 0.2466 | No |
| 45 | UBE2D3 | na |  |  | 8015 | 0.323 | 0.2519 | No |
| 46 | PIK3R3 | na |  |  | 8228 | 0.301 | 0.2460 | No |
| 47 | MKNK1 | na |  |  | 8285 | 0.295 | 0.2481 | No |
| 48 | RPS6KA1 | na |  |  | 8486 | 0.273 | 0.2423 | No |
| 49 | AKT1 | na |  |  | 8668 | 0.251 | 0.2371 | No |
| 50 | NCK1 | na |  |  | 9082 | 0.204 | 0.2189 | No |
| 51 | RIT1 | na |  |  | 9242 | 0.189 | 0.2138 | No |
| 52 | RAC1 | na |  |  | 9267 | 0.186 | 0.2157 | No |
| 53 | PIN1 | na |  |  | 9500 | 0.156 | 0.2063 | No |
| 54 | PAK4 | na |  |  | 9515 | 0.155 | 0.2082 | No |
| 55 | PPP1CA | na |  |  | 11047 | -0.009 | 0.1282 | No |
| 56 | PLA2G12A | na |  |  | 11173 | -0.024 | 0.1221 | No |
| 57 | PIKFYVE | na |  |  | 11737 | -0.091 | 0.0942 | No |
| 58 | CAB39 | na |  |  | 11740 | -0.091 | 0.0956 | No |
| 59 | CFL1 | na |  |  | 11761 | -0.094 | 0.0962 | No |
| 60 | DUSP3 | na |  |  | 11833 | -0.104 | 0.0942 | No |
| 61 | SMAD2 | na |  |  | 11890 | -0.113 | 0.0932 | No |
| 62 | THEM4 | na |  |  | 12242 | -0.148 | 0.0774 | No |
| 63 | RPS6KA3 | na |  |  | 12300 | -0.154 | 0.0770 | No |
| 64 | ARF1 | na |  |  | 12303 | -0.155 | 0.0796 | No |
| 65 | SLC2A1 | na |  |  | 12606 | -0.200 | 0.0672 | No |
| 66 | PTEN | na |  |  | 12643 | -0.204 | 0.0688 | No |
| 67 | NGF | na |  |  | 12678 | -0.210 | 0.0706 | No |
| 68 | RIPK1 | na |  |  | 12747 | -0.221 | 0.0708 | No |
| 69 | AKT1S1 | na |  |  | 13477 | -0.312 | 0.0380 | No |
| 70 | PLCG1 | na |  |  | 13591 | -0.329 | 0.0377 | No |
| 71 | MYD88 | na |  |  | 13595 | -0.329 | 0.0431 | No |
| 72 | CDKN1B | na |  |  | 13864 | -0.360 | 0.0352 | No |
| 73 | ECSIT | na |  |  | 14028 | -0.382 | 0.0332 | No |
| 74 | FGF17 | na |  |  | 14458 | -0.438 | 0.0182 | No |
| 75 | ARPC3 | na |  |  | 14610 | -0.464 | 0.0183 | No |
| 76 | CALR | na |  |  | 15148 | -0.538 | -0.0007 | No |
| 77 | TSC2 | na |  |  | 15161 | -0.539 | 0.0079 | No |
| 78 | PITX2 | na |  |  | 15761 | -0.639 | -0.0126 | No |
| 79 | CDKN1A | na |  |  | 15950 | -0.678 | -0.0108 | No |
| 80 | PDK1 | na |  |  | 15998 | -0.686 | -0.0016 | No |
| 81 | HSP90B1 | na |  |  | 16095 | -0.702 | 0.0053 | No |
| 82 | TRIB3 | na |  |  | 16278 | -0.753 | 0.0086 | No |
| 83 | TNFRSF1A | na |  |  | 16404 | -0.783 | 0.0155 | No |
| 84 | SQSTM1 | na |  |  | 16663 | -0.848 | 0.0164 | No |
| 85 | CAB39L | na |  |  | 16808 | -0.889 | 0.0240 | No |
| 86 | CSNK2B | na |  |  | 17990 | -1.323 | -0.0152 | No |
| 87 | STAT2 | na |  |  | 18119 | -1.384 | 0.0017 | No |
| 88 | MKNK2 | na |  |  | 18310 | -1.504 | 0.0174 | No |
| 89 | TIAM1 | na |  |  | 18560 | -1.701 | 0.0334 | No |
Table: GSEA details [plain text format]

  

Fig 2: HALLMARK\_PI3K\_AKT\_MTOR\_SIGNALING      
 Blue-Pink O' Gram in the Space of the Analyzed GeneSet

  

Fig 3: HALLMARK\_PI3K\_AKT\_MTOR\_SIGNALING: Random ES distribution      
 Gene set null distribution of ES for **HALLMARK\_PI3K\_AKT\_MTOR\_SIGNALING**

  
