# Supplementary material for: Heparin-based hydrogel scaffolding alters the transcriptomic profile and increases the chemoresistance of MDA-MB-231 triple-negative breast cancer cells
Source: Biomater Sci. 2020 Feb 13;8(10):2786–96. doi: 10.1039/c9bm01481k (PMC7497406; doi:10.1039/c9bm01481k)
Supplement: Supplementary file 2 [file BM-008-C9BM01481K-s002.zip › Supplementary File 4/EGFvControl/Pathways/my_analysis.Gsea.1545200981068/HALLMARK_KRAS_SIGNALING_UP.html]

Details for gene set HALLMARK\_KRAS\_SIGNALING\_UP[GSEA]

|  || Dataset | expr.class.cls#EGF\_versus\_CONTROL.class.cls#EGF\_versus\_CONTROL\_repos |
| Phenotype | class.cls#EGF\_versus\_CONTROL\_repos |
| Upregulated in class | CONTROL |
| GeneSet | HALLMARK\_KRAS\_SIGNALING\_UP |
| Enrichment Score (ES) | -0.4787049 |
| Normalized Enrichment Score (NES) | -2.2446744 |
| Nominal p-value | 0.0 |
| FDR q-value | 0.0 |
| FWER p-Value | 0.0 |
Table: GSEA Results Summary

  

Fig 1: Enrichment plot: HALLMARK\_KRAS\_SIGNALING\_UP      
 Profile of the Running ES Score & Positions of GeneSet Members on the Rank Ordered List

  

| PROBE | DESCRIPTION (from dataset) | GENE SYMBOL | GENE\_TITLE | RANK IN GENE LIST | RANK METRIC SCORE | RUNNING ES | CORE ENRICHMENT || 1 | SCG5 | na |  |  | 24 | 2.939 | 0.0194 | No |
| 2 | PDCD1LG2 | na |  |  | 40 | 2.738 | 0.0379 | No |
| 3 | EPHB2 | na |  |  | 194 | 2.110 | 0.0447 | No |
| 4 | RELN | na |  |  | 304 | 1.947 | 0.0527 | No |
| 5 | PLAU | na |  |  | 387 | 1.854 | 0.0614 | No |
| 6 | DOCK2 | na |  |  | 1128 | 1.469 | 0.0329 | No |
| 7 | ETS1 | na |  |  | 1416 | 1.372 | 0.0275 | No |
| 8 | RBM4 | na |  |  | 1443 | 1.365 | 0.0357 | No |
| 9 | YRDC | na |  |  | 1520 | 1.340 | 0.0411 | No |
| 10 | IL7R | na |  |  | 1696 | 1.295 | 0.0411 | No |
| 11 | CSF2RA | na |  |  | 1888 | 1.248 | 0.0398 | No |
| 12 | PTCD2 | na |  |  | 2192 | 1.180 | 0.0322 | No |
| 13 | AMMECR1 | na |  |  | 2491 | 1.113 | 0.0244 | No |
| 14 | GALNT3 | na |  |  | 2717 | 1.073 | 0.0201 | No |
| 15 | CBL | na |  |  | 2825 | 1.057 | 0.0220 | No |
| 16 | CDADC1 | na |  |  | 2973 | 1.030 | 0.0215 | No |
| 17 | STRN | na |  |  | 3169 | 0.994 | 0.0183 | No |
| 18 | PTPRR | na |  |  | 3555 | 0.928 | 0.0046 | No |
| 19 | HBEGF | na |  |  | 3593 | 0.921 | 0.0091 | No |
| 20 | ZNF639 | na |  |  | 3850 | 0.876 | 0.0018 | No |
| 21 | F13A1 | na |  |  | 4259 | 0.814 | -0.0138 | No |
| 22 | WDR33 | na |  |  | 4284 | 0.810 | -0.0094 | No |
| 23 | CXCR4 | na |  |  | 4387 | 0.794 | -0.0092 | No |
| 24 | NIN | na |  |  | 4526 | 0.774 | -0.0110 | No |
| 25 | TPH1 | na |  |  | 4698 | 0.749 | -0.0147 | No |
| 26 | BTBD3 | na |  |  | 4769 | 0.736 | -0.0132 | No |
| 27 | EVI5 | na |  |  | 4841 | 0.723 | -0.0118 | No |
| 28 | ITGBL1 | na |  |  | 4957 | 0.713 | -0.0128 | No |
| 29 | SDCCAG8 | na |  |  | 5138 | 0.687 | -0.0175 | No |
| 30 | AVL9 | na |  |  | 5537 | 0.633 | -0.0339 | No |
| 31 | HKDC1 | na |  |  | 5899 | 0.583 | -0.0487 | No |
| 32 | HDAC9 | na |  |  | 6002 | 0.570 | -0.0501 | No |
| 33 | HIST1H2BB | na |  |  | 6168 | 0.546 | -0.0549 | No |
| 34 | SNAP25 | na |  |  | 6482 | 0.505 | -0.0678 | No |
| 35 | PTBP2 | na |  |  | 6612 | 0.490 | -0.0711 | No |
| 36 | ANGPTL4 | na |  |  | 6796 | 0.468 | -0.0774 | No |
| 37 | GNG11 | na |  |  | 7229 | 0.418 | -0.0971 | No |
| 38 | USP12 | na |  |  | 7275 | 0.411 | -0.0966 | No |
| 39 | CROT | na |  |  | 7490 | 0.386 | -0.1051 | No |
| 40 | AKAP12 | na |  |  | 7591 | 0.374 | -0.1077 | No |
| 41 | IL1RL2 | na |  |  | 7709 | 0.362 | -0.1113 | No |
| 42 | ATG10 | na |  |  | 7838 | 0.345 | -0.1156 | No |
| 43 | PPP1R15A | na |  |  | 8701 | 0.246 | -0.1591 | No |
| 44 | TOR1AIP2 | na |  |  | 9350 | 0.177 | -0.1919 | No |
| 45 | CD37 | na |  |  | 9722 | 0.134 | -0.2104 | No |
| 46 | MTMR10 | na |  |  | 10024 | 0.104 | -0.2255 | No |
| 47 | ENG | na |  |  | 10136 | 0.091 | -0.2307 | No |
| 48 | INHBA | na |  |  | 10150 | 0.088 | -0.2307 | No |
| 49 | CBX8 | na |  |  | 10252 | 0.075 | -0.2355 | No |
| 50 | ETV5 | na |  |  | 10263 | 0.074 | -0.2355 | No |
| 51 | MAP3K1 | na |  |  | 10453 | 0.059 | -0.2450 | No |
| 52 | FBXO4 | na |  |  | 10661 | 0.030 | -0.2557 | No |
| 53 | RABGAP1L | na |  |  | 10691 | 0.026 | -0.2570 | No |
| 54 | DNMBP | na |  |  | 10750 | 0.019 | -0.2599 | No |
| 55 | ADAM17 | na |  |  | 10944 | 0.003 | -0.2700 | No |
| 56 | ZNF277 | na |  |  | 11341 | -0.044 | -0.2905 | No |
| 57 | IGF2 | na |  |  | 11370 | -0.047 | -0.2916 | No |
| 58 | SPARCL1 | na |  |  | 11374 | -0.047 | -0.2915 | No |
| 59 | LAPTM5 | na |  |  | 11536 | -0.061 | -0.2995 | No |
| 60 | VWA5A | na |  |  | 11539 | -0.062 | -0.2991 | No |
| 61 | CFH | na |  |  | 11540 | -0.062 | -0.2987 | No |
| 62 | AKT2 | na |  |  | 11631 | -0.075 | -0.3029 | No |
| 63 | ID2 | na |  |  | 11940 | -0.118 | -0.3182 | No |
| 64 | PRKG2 | na |  |  | 12287 | -0.153 | -0.3353 | No |
| 65 | ALDH1A2 | na |  |  | 12313 | -0.157 | -0.3355 | No |
| 66 | G0S2 | na |  |  | 12437 | -0.176 | -0.3407 | No |
| 67 | BPGM | na |  |  | 12551 | -0.191 | -0.3453 | No |
| 68 | TLR8 | na |  |  | 12599 | -0.197 | -0.3464 | No |
| 69 | NGF | na |  |  | 12678 | -0.210 | -0.3490 | No |
| 70 | GPNMB | na |  |  | 12807 | -0.231 | -0.3541 | No |
| 71 | LIF | na |  |  | 12907 | -0.232 | -0.3577 | No |
| 72 | F2RL1 | na |  |  | 13357 | -0.296 | -0.3792 | No |
| 73 | RGS16 | na |  |  | 13422 | -0.305 | -0.3804 | No |
| 74 | SNAP91 | na |  |  | 13588 | -0.329 | -0.3867 | No |
| 75 | TNFRSF1B | na |  |  | 13603 | -0.331 | -0.3851 | No |
| 76 | CXCL10 | na |  |  | 13845 | -0.359 | -0.3953 | No |
| 77 | MMD | na |  |  | 14145 | -0.398 | -0.4082 | No |
| 78 | ANO1 | na |  |  | 14172 | -0.401 | -0.4067 | No |
| 79 | LAT2 | na |  |  | 14245 | -0.413 | -0.4076 | No |
| 80 | TSPAN13 | na |  |  | 14288 | -0.419 | -0.4068 | No |
| 81 | MAFB | na |  |  | 14481 | -0.442 | -0.4138 | No |
| 82 | MAP7 | na |  |  | 14501 | -0.444 | -0.4117 | No |
| 83 | DCBLD2 | na |  |  | 14704 | -0.481 | -0.4189 | No |
| 84 | GADD45G | na |  |  | 14936 | -0.505 | -0.4275 | No |
| 85 | MPZL2 | na |  |  | 15081 | -0.526 | -0.4313 | No |
| 86 | TSPAN1 | na |  |  | 15212 | -0.548 | -0.4343 | No |
| 87 | CBR4 | na |  |  | 15246 | -0.554 | -0.4321 | No |
| 88 | PLAUR | na |  |  | 15641 | -0.614 | -0.4485 | No |
| 89 | SOX9 | na |  |  | 15836 | -0.654 | -0.4541 | No |
| 90 | TSPAN7 | na |  |  | 15945 | -0.677 | -0.4550 | No |
| 91 | MALL | na |  |  | 15951 | -0.678 | -0.4505 | No |
| 92 | PLEK2 | na |  |  | 16161 | -0.722 | -0.4564 | No |
| 93 | KLF4 | na |  |  | 16438 | -0.793 | -0.4653 | No |
| 94 | EMP1 | na |  |  | 16599 | -0.834 | -0.4678 | No |
| 95 | CAB39L | na |  |  | 16808 | -0.889 | -0.4725 | Yes |
| 96 | ETV4 | na |  |  | 16819 | -0.894 | -0.4667 | Yes |
| 97 | EREG | na |  |  | 16952 | -0.936 | -0.4670 | Yes |
| 98 | PCSK1N | na |  |  | 17151 | -0.995 | -0.4704 | Yes |
| 99 | ANKH | na |  |  | 17223 | -1.014 | -0.4670 | Yes |
| 100 | PSMB8 | na |  |  | 17300 | -1.042 | -0.4637 | Yes |
| 101 | ITGB2 | na |  |  | 17342 | -1.055 | -0.4584 | Yes |
| 102 | BTC | na |  |  | 17357 | -1.062 | -0.4517 | Yes |
| 103 | CCND2 | na |  |  | 17385 | -1.070 | -0.4456 | Yes |
| 104 | TNFAIP3 | na |  |  | 17494 | -1.121 | -0.4434 | Yes |
| 105 | CSF2 | na |  |  | 17557 | -1.135 | -0.4386 | Yes |
| 106 | CFB | na |  |  | 17623 | -1.158 | -0.4339 | Yes |
| 107 | SCN1B | na |  |  | 17660 | -1.170 | -0.4276 | Yes |
| 108 | GPRC5B | na |  |  | 17828 | -1.241 | -0.4276 | Yes |
| 109 | MMP11 | na |  |  | 17836 | -1.248 | -0.4192 | Yes |
| 110 | JUP | na |  |  | 17913 | -1.281 | -0.4142 | Yes |
| 111 | PRDM1 | na |  |  | 17971 | -1.315 | -0.4079 | Yes |
| 112 | CPE | na |  |  | 18145 | -1.396 | -0.4072 | Yes |
| 113 | SEMA3B | na |  |  | 18153 | -1.402 | -0.3977 | Yes |
| 114 | GLRX | na |  |  | 18169 | -1.407 | -0.3886 | Yes |
| 115 | TMEM158 | na |  |  | 18209 | -1.430 | -0.3806 | Yes |
| 116 | IL1B | na |  |  | 18217 | -1.433 | -0.3708 | Yes |
| 117 | ADAM8 | na |  |  | 18240 | -1.452 | -0.3618 | Yes |
| 118 | HSD11B1 | na |  |  | 18258 | -1.461 | -0.3524 | Yes |
| 119 | SPRY2 | na |  |  | 18272 | -1.475 | -0.3427 | Yes |
| 120 | TRIB1 | na |  |  | 18278 | -1.478 | -0.3326 | Yes |
| 121 | GFPT2 | na |  |  | 18347 | -1.525 | -0.3254 | Yes |
| 122 | NRP1 | na |  |  | 18351 | -1.527 | -0.3148 | Yes |
| 123 | IGFBP3 | na |  |  | 18475 | -1.624 | -0.3099 | Yes |
| 124 | TRAF1 | na |  |  | 18477 | -1.625 | -0.2985 | Yes |
| 125 | TRIB2 | na |  |  | 18481 | -1.628 | -0.2872 | Yes |
| 126 | KCNN4 | na |  |  | 18501 | -1.644 | -0.2766 | Yes |
| 127 | DUSP6 | na |  |  | 18544 | -1.690 | -0.2670 | Yes |
| 128 | CCL20 | na |  |  | 18550 | -1.693 | -0.2553 | Yes |
| 129 | ALDH1A3 | na |  |  | 18625 | -1.776 | -0.2467 | Yes |
| 130 | PTGS2 | na |  |  | 18633 | -1.789 | -0.2345 | Yes |
| 131 | PLAT | na |  |  | 18660 | -1.832 | -0.2230 | Yes |
| 132 | ITGA2 | na |  |  | 18843 | -2.164 | -0.2173 | Yes |
| 133 | CMKLR1 | na |  |  | 18878 | -2.270 | -0.2031 | Yes |
| 134 | FUCA1 | na |  |  | 18883 | -2.288 | -0.1872 | Yes |
| 135 | ST6GAL1 | na |  |  | 18917 | -2.345 | -0.1725 | Yes |
| 136 | TFPI | na |  |  | 18951 | -2.436 | -0.1571 | Yes |
| 137 | CTSS | na |  |  | 18977 | -2.550 | -0.1405 | Yes |
| 138 | ANXA10 | na |  |  | 19021 | -2.746 | -0.1234 | Yes |
| 139 | ETV1 | na |  |  | 19025 | -2.766 | -0.1041 | Yes |
| 140 | BIRC3 | na |  |  | 19043 | -2.872 | -0.0848 | Yes |
| 141 | PRRX1 | na |  |  | 19045 | -2.882 | -0.0646 | Yes |
| 142 | SATB1 | na |  |  | 19064 | -2.931 | -0.0449 | Yes |
| 143 | BMP2 | na |  |  | 19119 | -3.339 | -0.0243 | Yes |
| 144 | PECAM1 | na |  |  | 19167 | -4.033 | 0.0016 | Yes |
Table: GSEA details [plain text format]

  

Fig 2: HALLMARK\_KRAS\_SIGNALING\_UP      
 Blue-Pink O' Gram in the Space of the Analyzed GeneSet

  

Fig 3: HALLMARK\_KRAS\_SIGNALING\_UP: Random ES distribution      
 Gene set null distribution of ES for **HALLMARK\_KRAS\_SIGNALING\_UP**

  
