# Supplementary material for: Heparin-based hydrogel scaffolding alters the transcriptomic profile and increases the chemoresistance of MDA-MB-231 triple-negative breast cancer cells
Source: Biomater Sci. 2020 Feb 13;8(10):2786–96. doi: 10.1039/c9bm01481k (PMC7497406; doi:10.1039/c9bm01481k)
Supplement: Supplementary file 2 [file BM-008-C9BM01481K-s002.zip › Supplementary File 4/EGFvControl/Pathways/my_analysis.Gsea.1545200981068/HALLMARK_ADIPOGENESIS.html]

Details for gene set HALLMARK\_ADIPOGENESIS[GSEA]

|  || Dataset | expr.class.cls#EGF\_versus\_CONTROL.class.cls#EGF\_versus\_CONTROL\_repos |
| Phenotype | class.cls#EGF\_versus\_CONTROL\_repos |
| Upregulated in class | EGF |
| GeneSet | HALLMARK\_ADIPOGENESIS |
| Enrichment Score (ES) | 0.18528193 |
| Normalized Enrichment Score (NES) | 0.8531466 |
| Nominal p-value | 0.82175225 |
| FDR q-value | 0.8808889 |
| FWER p-Value | 1.0 |
Table: GSEA Results Summary

  

Fig 1: Enrichment plot: HALLMARK\_ADIPOGENESIS      
 Profile of the Running ES Score & Positions of GeneSet Members on the Rank Ordered List

  

| PROBE | DESCRIPTION (from dataset) | GENE SYMBOL | GENE\_TITLE | RANK IN GENE LIST | RANK METRIC SCORE | RUNNING ES | CORE ENRICHMENT || 1 | SLC1A5 | na |  |  | 171 | 2.171 | 0.0084 | Yes |
| 2 | COQ3 | na |  |  | 222 | 2.060 | 0.0223 | Yes |
| 3 | SLC5A6 | na |  |  | 595 | 1.708 | 0.0164 | Yes |
| 4 | NKIRAS1 | na |  |  | 633 | 1.681 | 0.0279 | Yes |
| 5 | AIFM1 | na |  |  | 634 | 1.679 | 0.0414 | Yes |
| 6 | CDKN2C | na |  |  | 635 | 1.679 | 0.0548 | Yes |
| 7 | IDH3A | na |  |  | 787 | 1.595 | 0.0597 | Yes |
| 8 | SCARB1 | na |  |  | 794 | 1.593 | 0.0721 | Yes |
| 9 | CHUK | na |  |  | 1122 | 1.471 | 0.0667 | Yes |
| 10 | GPHN | na |  |  | 1213 | 1.436 | 0.0735 | Yes |
| 11 | STOM | na |  |  | 1214 | 1.436 | 0.0850 | Yes |
| 12 | ME1 | na |  |  | 1379 | 1.385 | 0.0875 | Yes |
| 13 | UCP2 | na |  |  | 1461 | 1.359 | 0.0941 | Yes |
| 14 | ATP1B3 | na |  |  | 1525 | 1.340 | 0.1016 | Yes |
| 15 | AK2 | na |  |  | 1577 | 1.326 | 0.1095 | Yes |
| 16 | COX8A | na |  |  | 1597 | 1.318 | 0.1191 | Yes |
| 17 | SAMM50 | na |  |  | 2324 | 1.151 | 0.0901 | Yes |
| 18 | GHITM | na |  |  | 2334 | 1.148 | 0.0989 | Yes |
| 19 | GPAM | na |  |  | 2341 | 1.147 | 0.1077 | Yes |
| 20 | SLC25A10 | na |  |  | 2509 | 1.109 | 0.1078 | Yes |
| 21 | PEMT | na |  |  | 2609 | 1.093 | 0.1114 | Yes |
| 22 | HSPB8 | na |  |  | 2666 | 1.081 | 0.1171 | Yes |
| 23 | CS | na |  |  | 2862 | 1.050 | 0.1153 | Yes |
| 24 | NDUFS3 | na |  |  | 2961 | 1.032 | 0.1184 | Yes |
| 25 | PTCD3 | na |  |  | 3177 | 0.992 | 0.1150 | Yes |
| 26 | MCCC1 | na |  |  | 3348 | 0.963 | 0.1138 | Yes |
| 27 | GRPEL1 | na |  |  | 3510 | 0.935 | 0.1128 | Yes |
| 28 | DBT | na |  |  | 3552 | 0.928 | 0.1181 | Yes |
| 29 | PPP1R15B | na |  |  | 3580 | 0.924 | 0.1241 | Yes |
| 30 | OMD | na |  |  | 3694 | 0.905 | 0.1254 | Yes |
| 31 | PRDX3 | na |  |  | 3704 | 0.903 | 0.1322 | Yes |
| 32 | DLD | na |  |  | 3868 | 0.873 | 0.1306 | Yes |
| 33 | PPARG | na |  |  | 3915 | 0.866 | 0.1351 | Yes |
| 34 | ADIPOR2 | na |  |  | 3953 | 0.860 | 0.1401 | Yes |
| 35 | UQCRQ | na |  |  | 4082 | 0.839 | 0.1401 | Yes |
| 36 | ARL4A | na |  |  | 4169 | 0.826 | 0.1422 | Yes |
| 37 | DLAT | na |  |  | 4235 | 0.817 | 0.1453 | Yes |
| 38 | ITGA7 | na |  |  | 4250 | 0.815 | 0.1511 | Yes |
| 39 | GPD2 | na |  |  | 4376 | 0.795 | 0.1509 | Yes |
| 40 | LIPE | na |  |  | 4401 | 0.791 | 0.1560 | Yes |
| 41 | ABCB8 | na |  |  | 4402 | 0.791 | 0.1623 | Yes |
| 42 | ITSN1 | na |  |  | 4520 | 0.774 | 0.1624 | Yes |
| 43 | BCL2L13 | na |  |  | 4585 | 0.765 | 0.1651 | Yes |
| 44 | IMMT | na |  |  | 4595 | 0.765 | 0.1708 | Yes |
| 45 | CPT2 | na |  |  | 4647 | 0.756 | 0.1742 | Yes |
| 46 | CMBL | na |  |  | 4758 | 0.738 | 0.1743 | Yes |
| 47 | CYC1 | na |  |  | 4803 | 0.729 | 0.1778 | Yes |
| 48 | NDUFA5 | na |  |  | 4854 | 0.722 | 0.1810 | Yes |
| 49 | QDPR | na |  |  | 4883 | 0.720 | 0.1853 | Yes |
| 50 | PHLDB1 | na |  |  | 5172 | 0.682 | 0.1756 | No |
| 51 | HIBCH | na |  |  | 5226 | 0.675 | 0.1782 | No |
| 52 | ATP5O | na |  |  | 5449 | 0.645 | 0.1717 | No |
| 53 | LTC4S | na |  |  | 5632 | 0.620 | 0.1671 | No |
| 54 | GADD45A | na |  |  | 6102 | 0.555 | 0.1469 | No |
| 55 | MGLL | na |  |  | 6147 | 0.549 | 0.1490 | No |
| 56 | PREB | na |  |  | 6354 | 0.520 | 0.1423 | No |
| 57 | UQCRC1 | na |  |  | 6365 | 0.518 | 0.1460 | No |
| 58 | ACLY | na |  |  | 6379 | 0.516 | 0.1494 | No |
| 59 | G3BP2 | na |  |  | 6456 | 0.508 | 0.1495 | No |
| 60 | DHRS7B | na |  |  | 6466 | 0.507 | 0.1531 | No |
| 61 | MTCH2 | na |  |  | 6495 | 0.503 | 0.1556 | No |
| 62 | ACADM | na |  |  | 6509 | 0.502 | 0.1590 | No |
| 63 | SORBS1 | na |  |  | 6590 | 0.493 | 0.1587 | No |
| 64 | ATL2 | na |  |  | 6768 | 0.472 | 0.1532 | No |
| 65 | UCK1 | na |  |  | 6771 | 0.472 | 0.1569 | No |
| 66 | ANGPTL4 | na |  |  | 6796 | 0.468 | 0.1594 | No |
| 67 | UBQLN1 | na |  |  | 7133 | 0.429 | 0.1451 | No |
| 68 | CD302 | na |  |  | 7174 | 0.425 | 0.1464 | No |
| 69 | ELOVL6 | na |  |  | 7276 | 0.411 | 0.1444 | No |
| 70 | FZD4 | na |  |  | 7603 | 0.373 | 0.1303 | No |
| 71 | ALDH2 | na |  |  | 7882 | 0.339 | 0.1184 | No |
| 72 | SOD1 | na |  |  | 7945 | 0.332 | 0.1178 | No |
| 73 | CMPK1 | na |  |  | 7956 | 0.330 | 0.1199 | No |
| 74 | NMT1 | na |  |  | 7979 | 0.328 | 0.1214 | No |
| 75 | SDHB | na |  |  | 8021 | 0.323 | 0.1218 | No |
| 76 | COX6A1 | na |  |  | 8064 | 0.319 | 0.1221 | No |
| 77 | RNF11 | na |  |  | 8066 | 0.318 | 0.1246 | No |
| 78 | SSPN | na |  |  | 8214 | 0.303 | 0.1194 | No |
| 79 | SUCLG1 | na |  |  | 8421 | 0.282 | 0.1108 | No |
| 80 | NDUFAB1 | na |  |  | 8463 | 0.277 | 0.1108 | No |
| 81 | ACADL | na |  |  | 8492 | 0.273 | 0.1116 | No |
| 82 | IDH1 | na |  |  | 8504 | 0.271 | 0.1132 | No |
| 83 | DECR1 | na |  |  | 8591 | 0.263 | 0.1107 | No |
| 84 | SDHC | na |  |  | 8704 | 0.246 | 0.1068 | No |
| 85 | ACAA2 | na |  |  | 8723 | 0.243 | 0.1078 | No |
| 86 | DHRS7 | na |  |  | 8780 | 0.239 | 0.1068 | No |
| 87 | UQCR11 | na |  |  | 8840 | 0.232 | 0.1056 | No |
| 88 | PQLC3 | na |  |  | 8980 | 0.215 | 0.1000 | No |
| 89 | LPL | na |  |  | 9088 | 0.203 | 0.0960 | No |
| 90 | CHCHD10 | na |  |  | 9141 | 0.197 | 0.0948 | No |
| 91 | TALDO1 | na |  |  | 9239 | 0.189 | 0.0912 | No |
| 92 | DRAM2 | na |  |  | 9276 | 0.183 | 0.0908 | No |
| 93 | MYLK | na |  |  | 9344 | 0.178 | 0.0887 | No |
| 94 | ESRRA | na |  |  | 9380 | 0.176 | 0.0883 | No |
| 95 | ACOX1 | na |  |  | 9429 | 0.166 | 0.0871 | No |
| 96 | MRPL15 | na |  |  | 9435 | 0.165 | 0.0882 | No |
| 97 | HADH | na |  |  | 9445 | 0.164 | 0.0890 | No |
| 98 | RTN3 | na |  |  | 9694 | 0.138 | 0.0771 | No |
| 99 | PPM1B | na |  |  | 9964 | 0.107 | 0.0638 | No |
| 100 | ENPP2 | na |  |  | 10133 | 0.091 | 0.0557 | No |
| 101 | AGPAT3 | na |  |  | 10176 | 0.084 | 0.0541 | No |
| 102 | DHCR7 | na |  |  | 10300 | 0.069 | 0.0482 | No |
| 103 | MDH2 | na |  |  | 10374 | 0.061 | 0.0449 | No |
| 104 | SCP2 | na |  |  | 10592 | 0.039 | 0.0338 | No |
| 105 | ELMOD3 | na |  |  | 10704 | 0.024 | 0.0281 | No |
| 106 | YWHAG | na |  |  | 10825 | 0.011 | 0.0219 | No |
| 107 | RREB1 | na |  |  | 10952 | 0.002 | 0.0153 | No |
| 108 | SLC19A1 | na |  |  | 11074 | -0.013 | 0.0091 | No |
| 109 | ECHS1 | na |  |  | 11160 | -0.022 | 0.0048 | No |
| 110 | SPARCL1 | na |  |  | 11374 | -0.047 | -0.0060 | No |
| 111 | ESYT1 | na |  |  | 11549 | -0.063 | -0.0147 | No |
| 112 | REEP5 | na |  |  | 11587 | -0.068 | -0.0161 | No |
| 113 | COL15A1 | na |  |  | 11659 | -0.080 | -0.0192 | No |
| 114 | RIOK3 | na |  |  | 11696 | -0.085 | -0.0204 | No |
| 115 | POR | na |  |  | 11704 | -0.087 | -0.0201 | No |
| 116 | RAB34 | na |  |  | 11820 | -0.102 | -0.0253 | No |
| 117 | COL4A1 | na |  |  | 11875 | -0.110 | -0.0272 | No |
| 118 | LIFR | na |  |  | 12211 | -0.145 | -0.0437 | No |
| 119 | RETSAT | na |  |  | 12351 | -0.162 | -0.0497 | No |
| 120 | TKT | na |  |  | 12361 | -0.164 | -0.0489 | No |
| 121 | SULT1A1 | na |  |  | 12401 | -0.170 | -0.0495 | No |
| 122 | TST | na |  |  | 12730 | -0.219 | -0.0650 | No |
| 123 | UQCR10 | na |  |  | 13007 | -0.244 | -0.0776 | No |
| 124 | LPCAT3 | na |  |  | 13112 | -0.260 | -0.0810 | No |
| 125 | SLC25A1 | na |  |  | 13138 | -0.264 | -0.0802 | No |
| 126 | PGM1 | na |  |  | 13543 | -0.322 | -0.0988 | No |
| 127 | DDT | na |  |  | 13546 | -0.322 | -0.0964 | No |
| 128 | PHYH | na |  |  | 13576 | -0.327 | -0.0953 | No |
| 129 | BAZ2A | na |  |  | 13615 | -0.333 | -0.0946 | No |
| 130 | FAH | na |  |  | 13686 | -0.343 | -0.0955 | No |
| 131 | ACO2 | na |  |  | 13859 | -0.360 | -0.1017 | No |
| 132 | DNAJC15 | na |  |  | 13960 | -0.371 | -0.1040 | No |
| 133 | ADCY6 | na |  |  | 14106 | -0.393 | -0.1084 | No |
| 134 | TOB1 | na |  |  | 14149 | -0.399 | -0.1074 | No |
| 135 | IDH3G | na |  |  | 14200 | -0.405 | -0.1068 | No |
| 136 | TANK | na |  |  | 14216 | -0.408 | -0.1043 | No |
| 137 | PIM3 | na |  |  | 14543 | -0.451 | -0.1179 | No |
| 138 | DGAT1 | na |  |  | 14826 | -0.502 | -0.1287 | No |
| 139 | ARAF | na |  |  | 15601 | -0.605 | -0.1645 | No |
| 140 | CAT | na |  |  | 15662 | -0.617 | -0.1627 | No |
| 141 | ETFB | na |  |  | 15698 | -0.626 | -0.1595 | No |
| 142 | NDUFB7 | na |  |  | 15915 | -0.668 | -0.1655 | No |
| 143 | JAGN1 | na |  |  | 16071 | -0.698 | -0.1681 | No |
| 144 | GPX3 | na |  |  | 16209 | -0.732 | -0.1694 | No |
| 145 | COQ9 | na |  |  | 16299 | -0.757 | -0.1680 | No |
| 146 | ECH1 | na |  |  | 16356 | -0.770 | -0.1648 | No |
| 147 | CD151 | na |  |  | 16373 | -0.774 | -0.1594 | No |
| 148 | ACADS | na |  |  | 16402 | -0.782 | -0.1546 | No |
| 149 | ALDOA | na |  |  | 16455 | -0.799 | -0.1510 | No |
| 150 | GPX4 | na |  |  | 16614 | -0.837 | -0.1526 | No |
| 151 | MAP4K3 | na |  |  | 16618 | -0.838 | -0.1460 | No |
| 152 | PFKL | na |  |  | 16708 | -0.859 | -0.1438 | No |
| 153 | REEP6 | na |  |  | 16765 | -0.874 | -0.1398 | No |
| 154 | COQ5 | na |  |  | 16867 | -0.911 | -0.1378 | No |
| 155 | PLIN2 | na |  |  | 16877 | -0.913 | -0.1309 | No |
| 156 | GBE1 | na |  |  | 16983 | -0.947 | -0.1288 | No |
| 157 | SNCG | na |  |  | 17064 | -0.967 | -0.1253 | No |
| 158 | APLP2 | na |  |  | 17252 | -1.022 | -0.1269 | No |
| 159 | APOE | na |  |  | 17293 | -1.040 | -0.1207 | No |
| 160 | IFNGR1 | na |  |  | 17299 | -1.042 | -0.1126 | No |
| 161 | VEGFB | na |  |  | 17563 | -1.137 | -0.1173 | No |
| 162 | STAT5A | na |  |  | 17598 | -1.148 | -0.1099 | No |
| 163 | COX7B | na |  |  | 17605 | -1.151 | -0.1010 | No |
| 164 | C3 | na |  |  | 17617 | -1.156 | -0.0923 | No |
| 165 | MGST3 | na |  |  | 17776 | -1.214 | -0.0909 | No |
| 166 | PEX14 | na |  |  | 17786 | -1.218 | -0.0816 | No |
| 167 | DNAJB9 | na |  |  | 17933 | -1.292 | -0.0790 | No |
| 168 | PFKFB3 | na |  |  | 18027 | -1.345 | -0.0731 | No |
| 169 | CRAT | na |  |  | 18281 | -1.480 | -0.0745 | No |
| 170 | SLC27A1 | na |  |  | 18306 | -1.501 | -0.0637 | No |
| 171 | ANGPT1 | na |  |  | 18334 | -1.518 | -0.0530 | No |
| 172 | BCL6 | na |  |  | 18551 | -1.694 | -0.0508 | No |
| 173 | LAMA4 | na |  |  | 18956 | -2.463 | -0.0523 | No |
| 174 | ABCA1 | na |  |  | 18984 | -2.587 | -0.0330 | No |
| 175 | PDCD4 | na |  |  | 18987 | -2.595 | -0.0123 | No |
| 176 | CCNG2 | na |  |  | 19058 | -2.911 | 0.0074 | No |
Table: GSEA details [plain text format]

  

Fig 2: HALLMARK\_ADIPOGENESIS      
 Blue-Pink O' Gram in the Space of the Analyzed GeneSet

  

Fig 3: HALLMARK\_ADIPOGENESIS: Random ES distribution      
 Gene set null distribution of ES for **HALLMARK\_ADIPOGENESIS**

  
