# Supplementary material for: Heparin-based hydrogel scaffolding alters the transcriptomic profile and increases the chemoresistance of MDA-MB-231 triple-negative breast cancer cells
Source: Biomater Sci. 2020 Feb 13;8(10):2786–96. doi: 10.1039/c9bm01481k (PMC7497406; doi:10.1039/c9bm01481k)
Supplement: Supplementary file 2 [file BM-008-C9BM01481K-s002.zip › Supplementary File 4/EGFvControl/Pathways/my_analysis.Gsea.1545200981068/HALLMARK_ANDROGEN_RESPONSE.html]

Details for gene set HALLMARK\_ANDROGEN\_RESPONSE[GSEA]

|  || Dataset | expr.class.cls#EGF\_versus\_CONTROL.class.cls#EGF\_versus\_CONTROL\_repos |
| Phenotype | class.cls#EGF\_versus\_CONTROL\_repos |
| Upregulated in class | CONTROL |
| GeneSet | HALLMARK\_ANDROGEN\_RESPONSE |
| Enrichment Score (ES) | -0.2543212 |
| Normalized Enrichment Score (NES) | -1.1102102 |
| Nominal p-value | 0.2539267 |
| FDR q-value | 0.28372526 |
| FWER p-Value | 0.994 |
Table: GSEA Results Summary

  

Fig 1: Enrichment plot: HALLMARK\_ANDROGEN\_RESPONSE      
 Profile of the Running ES Score & Positions of GeneSet Members on the Rank Ordered List

  

| PROBE | DESCRIPTION (from dataset) | GENE SYMBOL | GENE\_TITLE | RANK IN GENE LIST | RANK METRIC SCORE | RUNNING ES | CORE ENRICHMENT || 1 | HOMER2 | na |  |  | 95 | 2.356 | 0.0244 | Yes |
| 2 | CENPN | na |  |  | 252 | 2.028 | 0.0416 | Yes |
| 3 | SLC26A2 | na |  |  | 537 | 1.748 | 0.0486 | Yes |
| 4 | HERC3 | na |  |  | 566 | 1.727 | 0.0687 | Yes |
| 5 | RRP12 | na |  |  | 758 | 1.606 | 0.0787 | Yes |
| 6 | UAP1 | na |  |  | 781 | 1.597 | 0.0975 | Yes |
| 7 | PA2G4 | na |  |  | 838 | 1.574 | 0.1142 | Yes |
| 8 | FKBP5 | na |  |  | 1120 | 1.472 | 0.1179 | Yes |
| 9 | SMS | na |  |  | 1405 | 1.376 | 0.1202 | Yes |
| 10 | CDK6 | na |  |  | 1642 | 1.309 | 0.1242 | Yes |
| 11 | NGLY1 | na |  |  | 1735 | 1.284 | 0.1354 | Yes |
| 12 | ELOVL5 | na |  |  | 1901 | 1.246 | 0.1423 | Yes |
| 13 | XRCC6 | na |  |  | 1990 | 1.222 | 0.1529 | Yes |
| 14 | DHCR24 | na |  |  | 2137 | 1.190 | 0.1602 | Yes |
| 15 | PMEPA1 | na |  |  | 2174 | 1.184 | 0.1731 | Yes |
| 16 | CCND3 | na |  |  | 2241 | 1.169 | 0.1842 | Yes |
| 17 | HMGCR | na |  |  | 2377 | 1.137 | 0.1913 | Yes |
| 18 | ACTN1 | na |  |  | 2399 | 1.133 | 0.2044 | Yes |
| 19 | CCND1 | na |  |  | 2400 | 1.133 | 0.2185 | Yes |
| 20 | GNAI3 | na |  |  | 2417 | 1.129 | 0.2318 | Yes |
| 21 | SORD | na |  |  | 2578 | 1.097 | 0.2371 | Yes |
| 22 | INPP4B | na |  |  | 2707 | 1.075 | 0.2438 | Yes |
| 23 | PTPN21 | na |  |  | 3149 | 0.997 | 0.2332 | No |
| 24 | ABCC4 | na |  |  | 3645 | 0.914 | 0.2187 | No |
| 25 | ELL2 | na |  |  | 4025 | 0.847 | 0.2094 | No |
| 26 | SPCS3 | na |  |  | 4135 | 0.831 | 0.2141 | No |
| 27 | CDC14B | na |  |  | 4672 | 0.753 | 0.1955 | No |
| 28 | ELK4 | na |  |  | 4775 | 0.734 | 0.1993 | No |
| 29 | XRCC5 | na |  |  | 4788 | 0.733 | 0.2078 | No |
| 30 | RAB4A | na |  |  | 5005 | 0.708 | 0.2053 | No |
| 31 | ABHD2 | na |  |  | 5583 | 0.627 | 0.1830 | No |
| 32 | UBE2I | na |  |  | 6180 | 0.544 | 0.1586 | No |
| 33 | STK39 | na |  |  | 6840 | 0.463 | 0.1299 | No |
| 34 | GPD1L | na |  |  | 6916 | 0.454 | 0.1316 | No |
| 35 | TNFAIP8 | na |  |  | 7099 | 0.433 | 0.1275 | No |
| 36 | KRT8 | na |  |  | 7138 | 0.429 | 0.1309 | No |
| 37 | BMPR1B | na |  |  | 7163 | 0.426 | 0.1349 | No |
| 38 | PDLIM5 | na |  |  | 7457 | 0.391 | 0.1245 | No |
| 39 | SRF | na |  |  | 7554 | 0.379 | 0.1242 | No |
| 40 | NCOA4 | na |  |  | 7590 | 0.374 | 0.1270 | No |
| 41 | AKAP12 | na |  |  | 7591 | 0.374 | 0.1317 | No |
| 42 | TPD52 | na |  |  | 7821 | 0.347 | 0.1240 | No |
| 43 | HMGCS1 | na |  |  | 8453 | 0.278 | 0.0945 | No |
| 44 | ADRM1 | na |  |  | 8507 | 0.271 | 0.0951 | No |
| 45 | AKT1 | na |  |  | 8668 | 0.251 | 0.0899 | No |
| 46 | SRP19 | na |  |  | 8965 | 0.216 | 0.0771 | No |
| 47 | PTK2B | na |  |  | 9059 | 0.207 | 0.0748 | No |
| 48 | MAK | na |  |  | 9370 | 0.176 | 0.0608 | No |
| 49 | PGM3 | na |  |  | 9555 | 0.150 | 0.0530 | No |
| 50 | APPBP2 | na |  |  | 9852 | 0.121 | 0.0390 | No |
| 51 | IDI1 | na |  |  | 9872 | 0.119 | 0.0395 | No |
| 52 | NKX3-1 | na |  |  | 10658 | 0.030 | -0.0012 | No |
| 53 | FADS1 | na |  |  | 10812 | 0.012 | -0.0090 | No |
| 54 | VAPA | na |  |  | 10854 | 0.006 | -0.0111 | No |
| 55 | ADAMTS1 | na |  |  | 10968 | -0.000 | -0.0170 | No |
| 56 | PIAS1 | na |  |  | 11046 | -0.009 | -0.0209 | No |
| 57 | ZBTB10 | na |  |  | 11051 | -0.010 | -0.0210 | No |
| 58 | ACSL3 | na |  |  | 11105 | -0.017 | -0.0236 | No |
| 59 | LIFR | na |  |  | 12211 | -0.145 | -0.0796 | No |
| 60 | RPS6KA3 | na |  |  | 12300 | -0.154 | -0.0823 | No |
| 61 | LMAN1 | na |  |  | 12736 | -0.220 | -0.1023 | No |
| 62 | UBE2J1 | na |  |  | 12757 | -0.222 | -0.1006 | No |
| 63 | CAMKK2 | na |  |  | 13063 | -0.251 | -0.1134 | No |
| 64 | TMEM50A | na |  |  | 13807 | -0.350 | -0.1479 | No |
| 65 | SEC24D | na |  |  | 14410 | -0.434 | -0.1740 | No |
| 66 | MAP7 | na |  |  | 14501 | -0.444 | -0.1731 | No |
| 67 | GSR | na |  |  | 14564 | -0.456 | -0.1707 | No |
| 68 | STEAP4 | na |  |  | 14613 | -0.465 | -0.1674 | No |
| 69 | SGK1 | na |  |  | 14627 | -0.466 | -0.1623 | No |
| 70 | IQGAP2 | na |  |  | 14689 | -0.479 | -0.1595 | No |
| 71 | TMPRSS2 | na |  |  | 15893 | -0.665 | -0.2141 | No |
| 72 | MERTK | na |  |  | 16180 | -0.727 | -0.2200 | No |
| 73 | H1F0 | na |  |  | 16221 | -0.737 | -0.2129 | No |
| 74 | MYL12A | na |  |  | 16775 | -0.877 | -0.2309 | No |
| 75 | ANKH | na |  |  | 17223 | -1.014 | -0.2417 | No |
| 76 | ARID5B | na |  |  | 17386 | -1.071 | -0.2368 | No |
| 77 | ITGAV | na |  |  | 17601 | -1.149 | -0.2336 | No |
| 78 | ZMIZ1 | na |  |  | 17603 | -1.149 | -0.2193 | No |
| 79 | B4GALT1 | na |  |  | 17619 | -1.157 | -0.2057 | No |
| 80 | INSIG1 | na |  |  | 17727 | -1.193 | -0.1964 | No |
| 81 | DNAJB9 | na |  |  | 17933 | -1.292 | -0.1910 | No |
| 82 | SPDEF | na |  |  | 18117 | -1.383 | -0.1833 | No |
| 83 | KRT19 | na |  |  | 18142 | -1.395 | -0.1671 | No |
| 84 | SLC38A2 | na |  |  | 18461 | -1.614 | -0.1636 | No |
| 85 | TSC22D1 | na |  |  | 18536 | -1.680 | -0.1465 | No |
| 86 | DBI | na |  |  | 18547 | -1.692 | -0.1259 | No |
| 87 | B2M | na |  |  | 18622 | -1.775 | -0.1077 | No |
| 88 | ALDH1A3 | na |  |  | 18625 | -1.776 | -0.0856 | No |
| 89 | SCD | na |  |  | 19013 | -2.713 | -0.0720 | No |
| 90 | NDRG1 | na |  |  | 19083 | -3.028 | -0.0378 | No |
| 91 | SAT1 | na |  |  | 19139 | -3.506 | 0.0031 | No |
Table: GSEA details [plain text format]

  

Fig 2: HALLMARK\_ANDROGEN\_RESPONSE      
 Blue-Pink O' Gram in the Space of the Analyzed GeneSet

  

Fig 3: HALLMARK\_ANDROGEN\_RESPONSE: Random ES distribution      
 Gene set null distribution of ES for **HALLMARK\_ANDROGEN\_RESPONSE**

  
