# Supplementary material for: Heparin-based hydrogel scaffolding alters the transcriptomic profile and increases the chemoresistance of MDA-MB-231 triple-negative breast cancer cells
Source: Biomater Sci. 2020 Feb 13;8(10):2786–96. doi: 10.1039/c9bm01481k (PMC7497406; doi:10.1039/c9bm01481k)
Supplement: Supplementary file 2 [file BM-008-C9BM01481K-s002.zip › Supplementary File 4/EGFvControl/Pathways/my_analysis.Gsea.1545200981068/HALLMARK_MYOGENESIS.html]

Details for gene set HALLMARK\_MYOGENESIS[GSEA]

|  || Dataset | expr.class.cls#EGF\_versus\_CONTROL.class.cls#EGF\_versus\_CONTROL\_repos |
| Phenotype | class.cls#EGF\_versus\_CONTROL\_repos |
| Upregulated in class | CONTROL |
| GeneSet | HALLMARK\_MYOGENESIS |
| Enrichment Score (ES) | -0.38771492 |
| Normalized Enrichment Score (NES) | -1.7722665 |
| Nominal p-value | 0.0 |
| FDR q-value | 0.0026620578 |
| FWER p-Value | 0.02 |
Table: GSEA Results Summary

  

Fig 1: Enrichment plot: HALLMARK\_MYOGENESIS      
 Profile of the Running ES Score & Positions of GeneSet Members on the Rank Ordered List

  

| PROBE | DESCRIPTION (from dataset) | GENE SYMBOL | GENE\_TITLE | RANK IN GENE LIST | RANK METRIC SCORE | RUNNING ES | CORE ENRICHMENT || 1 | FST | na |  |  | 93 | 2.378 | 0.0141 | No |
| 2 | NQO1 | na |  |  | 141 | 2.223 | 0.0293 | No |
| 3 | ATP2A1 | na |  |  | 542 | 1.744 | 0.0222 | No |
| 4 | SPHK1 | na |  |  | 643 | 1.674 | 0.0303 | No |
| 5 | IFRD1 | na |  |  | 799 | 1.591 | 0.0349 | No |
| 6 | REEP1 | na |  |  | 1212 | 1.437 | 0.0247 | No |
| 7 | GADD45B | na |  |  | 1283 | 1.417 | 0.0323 | No |
| 8 | DTNA | na |  |  | 1386 | 1.383 | 0.0380 | No |
| 9 | COX7A1 | na |  |  | 1456 | 1.360 | 0.0452 | No |
| 10 | ABLIM1 | na |  |  | 1899 | 1.246 | 0.0319 | No |
| 11 | ADAM12 | na |  |  | 1937 | 1.238 | 0.0399 | No |
| 12 | FLII | na |  |  | 2197 | 1.178 | 0.0357 | No |
| 13 | LSP1 | na |  |  | 2389 | 1.134 | 0.0347 | No |
| 14 | PPP1R3C | na |  |  | 2401 | 1.133 | 0.0431 | No |
| 15 | PFKM | na |  |  | 2446 | 1.124 | 0.0498 | No |
| 16 | HSPB8 | na |  |  | 2666 | 1.081 | 0.0469 | No |
| 17 | SPTAN1 | na |  |  | 2725 | 1.072 | 0.0524 | No |
| 18 | KIFC3 | na |  |  | 2805 | 1.059 | 0.0567 | No |
| 19 | COL6A3 | na |  |  | 3117 | 1.003 | 0.0484 | No |
| 20 | CNN3 | na |  |  | 3370 | 0.959 | 0.0428 | No |
| 21 | HBEGF | na |  |  | 3593 | 0.921 | 0.0385 | No |
| 22 | PICK1 | na |  |  | 3898 | 0.868 | 0.0294 | No |
| 23 | EPHB3 | na |  |  | 3917 | 0.865 | 0.0354 | No |
| 24 | TEAD4 | na |  |  | 4111 | 0.835 | 0.0319 | No |
| 25 | TPM3 | na |  |  | 4207 | 0.821 | 0.0335 | No |
| 26 | ITGA7 | na |  |  | 4250 | 0.815 | 0.0378 | No |
| 27 | LAMA2 | na |  |  | 4257 | 0.815 | 0.0439 | No |
| 28 | EIF4A2 | na |  |  | 4370 | 0.798 | 0.0444 | No |
| 29 | PDE4DIP | na |  |  | 4571 | 0.768 | 0.0401 | No |
| 30 | SMTN | na |  |  | 4770 | 0.736 | 0.0355 | No |
| 31 | FDPS | na |  |  | 4955 | 0.713 | 0.0316 | No |
| 32 | PRNP | na |  |  | 4977 | 0.711 | 0.0361 | No |
| 33 | ACSL1 | na |  |  | 5124 | 0.689 | 0.0340 | No |
| 34 | RB1 | na |  |  | 5179 | 0.681 | 0.0365 | No |
| 35 | STC2 | na |  |  | 5348 | 0.658 | 0.0330 | No |
| 36 | CHRNB1 | na |  |  | 5556 | 0.632 | 0.0272 | No |
| 37 | KLF5 | na |  |  | 5811 | 0.596 | 0.0186 | No |
| 38 | MYH3 | na |  |  | 5934 | 0.579 | 0.0168 | No |
| 39 | SORBS1 | na |  |  | 6590 | 0.493 | -0.0136 | No |
| 40 | AGL | na |  |  | 7131 | 0.430 | -0.0385 | No |
| 41 | CKMT2 | na |  |  | 7230 | 0.418 | -0.0404 | No |
| 42 | SIRT2 | na |  |  | 7355 | 0.401 | -0.0437 | No |
| 43 | AEBP1 | na |  |  | 7388 | 0.398 | -0.0422 | No |
| 44 | MYO1C | na |  |  | 7509 | 0.385 | -0.0454 | No |
| 45 | SCHIP1 | na |  |  | 7720 | 0.360 | -0.0536 | No |
| 46 | MAPK12 | na |  |  | 7760 | 0.355 | -0.0528 | No |
| 47 | SGCA | na |  |  | 8124 | 0.315 | -0.0693 | No |
| 48 | SSPN | na |  |  | 8214 | 0.303 | -0.0716 | No |
| 49 | MB | na |  |  | 8834 | 0.233 | -0.1022 | No |
| 50 | TAGLN | na |  |  | 9033 | 0.211 | -0.1109 | No |
| 51 | PYGM | na |  |  | 9209 | 0.191 | -0.1185 | No |
| 52 | RIT1 | na |  |  | 9242 | 0.189 | -0.1187 | No |
| 53 | MYLK | na |  |  | 9344 | 0.178 | -0.1226 | No |
| 54 | IGFBP7 | na |  |  | 9375 | 0.176 | -0.1228 | No |
| 55 | TPD52L1 | na |  |  | 9466 | 0.161 | -0.1262 | No |
| 56 | ENO3 | na |  |  | 10445 | 0.059 | -0.1770 | No |
| 57 | FHL1 | na |  |  | 10688 | 0.027 | -0.1895 | No |
| 58 | PSEN2 | na |  |  | 11451 | -0.056 | -0.2290 | No |
| 59 | CAV3 | na |  |  | 11492 | -0.059 | -0.2307 | No |
| 60 | COL4A2 | na |  |  | 11618 | -0.072 | -0.2366 | No |
| 61 | AKT2 | na |  |  | 11631 | -0.075 | -0.2367 | No |
| 62 | COL15A1 | na |  |  | 11659 | -0.080 | -0.2374 | No |
| 63 | SPARC | na |  |  | 12049 | -0.132 | -0.2568 | No |
| 64 | ADCY9 | na |  |  | 12243 | -0.148 | -0.2657 | No |
| 65 | TNNC1 | na |  |  | 12621 | -0.201 | -0.2839 | No |
| 66 | COL1A1 | na |  |  | 12777 | -0.227 | -0.2902 | No |
| 67 | SORBS3 | na |  |  | 13029 | -0.248 | -0.3014 | No |
| 68 | TCAP | na |  |  | 13322 | -0.291 | -0.3144 | No |
| 69 | BIN1 | na |  |  | 13406 | -0.303 | -0.3164 | No |
| 70 | NOTCH1 | na |  |  | 13411 | -0.304 | -0.3141 | No |
| 71 | PDLIM7 | na |  |  | 13503 | -0.318 | -0.3164 | No |
| 72 | BAG1 | na |  |  | 13638 | -0.338 | -0.3207 | No |
| 73 | MYH11 | na |  |  | 13862 | -0.360 | -0.3296 | No |
| 74 | CACNA1H | na |  |  | 14194 | -0.405 | -0.3437 | No |
| 75 | NAV2 | na |  |  | 14526 | -0.448 | -0.3575 | No |
| 76 | SH2B1 | na |  |  | 14665 | -0.475 | -0.3609 | No |
| 77 | DMPK | na |  |  | 14694 | -0.479 | -0.3586 | No |
| 78 | LDB3 | na |  |  | 14891 | -0.502 | -0.3649 | No |
| 79 | GNAO1 | na |  |  | 15033 | -0.516 | -0.3682 | No |
| 80 | CTF1 | na |  |  | 15120 | -0.531 | -0.3684 | No |
| 81 | TSC2 | na |  |  | 15161 | -0.539 | -0.3662 | No |
| 82 | CHRNG | na |  |  | 15234 | -0.552 | -0.3656 | No |
| 83 | ST5 | na |  |  | 15369 | -0.577 | -0.3680 | No |
| 84 | SYNGR2 | na |  |  | 15687 | -0.624 | -0.3797 | No |
| 85 | TNNC2 | na |  |  | 15832 | -0.654 | -0.3820 | No |
| 86 | PTP4A3 | na |  |  | 15941 | -0.675 | -0.3823 | Yes |
| 87 | CDKN1A | na |  |  | 15950 | -0.678 | -0.3774 | Yes |
| 88 | CAMK2B | na |  |  | 16099 | -0.703 | -0.3795 | Yes |
| 89 | PLXNB2 | na |  |  | 16127 | -0.711 | -0.3753 | Yes |
| 90 | GPX3 | na |  |  | 16209 | -0.732 | -0.3737 | Yes |
| 91 | PKIA | na |  |  | 16270 | -0.750 | -0.3709 | Yes |
| 92 | MEF2D | na |  |  | 16275 | -0.751 | -0.3651 | Yes |
| 93 | ACHE | na |  |  | 16288 | -0.755 | -0.3597 | Yes |
| 94 | ERBB3 | na |  |  | 16345 | -0.768 | -0.3565 | Yes |
| 95 | MEF2A | na |  |  | 16383 | -0.777 | -0.3523 | Yes |
| 96 | CKB | na |  |  | 16470 | -0.804 | -0.3504 | Yes |
| 97 | AK1 | na |  |  | 16503 | -0.811 | -0.3456 | Yes |
| 98 | MRAS | na |  |  | 16513 | -0.815 | -0.3396 | Yes |
| 99 | ATP6AP1 | na |  |  | 16807 | -0.889 | -0.3479 | Yes |
| 100 | HDAC5 | na |  |  | 16964 | -0.940 | -0.3486 | Yes |
| 101 | MEF2C | na |  |  | 17019 | -0.957 | -0.3438 | Yes |
| 102 | GABARAPL2 | na |  |  | 17026 | -0.958 | -0.3364 | Yes |
| 103 | PC | na |  |  | 17387 | -1.072 | -0.3468 | Yes |
| 104 | COL6A2 | na |  |  | 17567 | -1.138 | -0.3471 | Yes |
| 105 | TGFB1 | na |  |  | 17599 | -1.148 | -0.3396 | Yes |
| 106 | OCEL1 | na |  |  | 17602 | -1.149 | -0.3305 | Yes |
| 107 | MYL6B | na |  |  | 17613 | -1.154 | -0.3219 | Yes |
| 108 | TNNT1 | na |  |  | 17637 | -1.163 | -0.3138 | Yes |
| 109 | VIPR1 | na |  |  | 17646 | -1.165 | -0.3050 | Yes |
| 110 | SLC6A8 | na |  |  | 17923 | -1.288 | -0.3092 | Yes |
| 111 | DMD | na |  |  | 17999 | -1.329 | -0.3025 | Yes |
| 112 | LPIN1 | na |  |  | 18020 | -1.341 | -0.2929 | Yes |
| 113 | SOD3 | na |  |  | 18026 | -1.343 | -0.2824 | Yes |
| 114 | GAA | na |  |  | 18088 | -1.375 | -0.2747 | Yes |
| 115 | SH3BGR | na |  |  | 18096 | -1.376 | -0.2641 | Yes |
| 116 | SPDEF | na |  |  | 18117 | -1.383 | -0.2541 | Yes |
| 117 | ITGB4 | na |  |  | 18146 | -1.397 | -0.2445 | Yes |
| 118 | ITGB5 | na |  |  | 18154 | -1.402 | -0.2337 | Yes |
| 119 | WWTR1 | na |  |  | 18194 | -1.422 | -0.2244 | Yes |
| 120 | GSN | na |  |  | 18275 | -1.477 | -0.2168 | Yes |
| 121 | CRAT | na |  |  | 18281 | -1.480 | -0.2053 | Yes |
| 122 | APP | na |  |  | 18352 | -1.528 | -0.1968 | Yes |
| 123 | TPM2 | na |  |  | 18358 | -1.533 | -0.1849 | Yes |
| 124 | BHLHE40 | na |  |  | 18387 | -1.556 | -0.1739 | Yes |
| 125 | SPEG | na |  |  | 18434 | -1.607 | -0.1635 | Yes |
| 126 | IGFBP3 | na |  |  | 18475 | -1.624 | -0.1527 | Yes |
| 127 | CLU | na |  |  | 18498 | -1.641 | -0.1408 | Yes |
| 128 | CFD | na |  |  | 18540 | -1.683 | -0.1295 | Yes |
| 129 | SVIL | na |  |  | 18566 | -1.704 | -0.1173 | Yes |
| 130 | MAPRE3 | na |  |  | 18700 | -1.894 | -0.1092 | Yes |
| 131 | SCD | na |  |  | 19013 | -2.713 | -0.1039 | Yes |
| 132 | FOXO4 | na |  |  | 19096 | -3.088 | -0.0836 | Yes |
| 133 | DAPK2 | na |  |  | 19121 | -3.348 | -0.0582 | Yes |
| 134 | FABP3 | na |  |  | 19147 | -3.680 | -0.0302 | Yes |
| 135 | PPFIA4 | na |  |  | 19170 | -4.119 | 0.0015 | Yes |
Table: GSEA details [plain text format]

  

Fig 2: HALLMARK\_MYOGENESIS      
 Blue-Pink O' Gram in the Space of the Analyzed GeneSet

  

Fig 3: HALLMARK\_MYOGENESIS: Random ES distribution      
 Gene set null distribution of ES for **HALLMARK\_MYOGENESIS**

  
