# Supplementary material for: Heparin-based hydrogel scaffolding alters the transcriptomic profile and increases the chemoresistance of MDA-MB-231 triple-negative breast cancer cells
Source: Biomater Sci. 2020 Feb 13;8(10):2786–96. doi: 10.1039/c9bm01481k (PMC7497406; doi:10.1039/c9bm01481k)
Supplement: Supplementary file 2 [file BM-008-C9BM01481K-s002.zip › Supplementary File 4/EGFvControl/Pathways/my_analysis.Gsea.1545200981068/HALLMARK_P53_PATHWAY.html]

Details for gene set HALLMARK\_P53\_PATHWAY[GSEA]

|  || Dataset | expr.class.cls#EGF\_versus\_CONTROL.class.cls#EGF\_versus\_CONTROL\_repos |
| Phenotype | class.cls#EGF\_versus\_CONTROL\_repos |
| Upregulated in class | CONTROL |
| GeneSet | HALLMARK\_P53\_PATHWAY |
| Enrichment Score (ES) | -0.30544576 |
| Normalized Enrichment Score (NES) | -1.4778296 |
| Nominal p-value | 0.0030864198 |
| FDR q-value | 0.021856036 |
| FWER p-Value | 0.265 |
Table: GSEA Results Summary

  

Fig 1: Enrichment plot: HALLMARK\_P53\_PATHWAY      
 Profile of the Running ES Score & Positions of GeneSet Members on the Rank Ordered List

  

| PROBE | DESCRIPTION (from dataset) | GENE SYMBOL | GENE\_TITLE | RANK IN GENE LIST | RANK METRIC SCORE | RUNNING ES | CORE ENRICHMENT || 1 | SLC3A2 | na |  |  | 191 | 2.122 | 0.0036 | No |
| 2 | NUDT15 | na |  |  | 250 | 2.029 | 0.0136 | No |
| 3 | RAD51C | na |  |  | 510 | 1.767 | 0.0113 | No |
| 4 | DEF6 | na |  |  | 561 | 1.729 | 0.0198 | No |
| 5 | LDHB | na |  |  | 598 | 1.705 | 0.0288 | No |
| 6 | SPHK1 | na |  |  | 643 | 1.674 | 0.0372 | No |
| 7 | IRAK1 | na |  |  | 985 | 1.518 | 0.0291 | No |
| 8 | HSPA4L | na |  |  | 1129 | 1.468 | 0.0310 | No |
| 9 | RCHY1 | na |  |  | 1185 | 1.446 | 0.0373 | No |
| 10 | STOM | na |  |  | 1214 | 1.436 | 0.0451 | No |
| 11 | SLC19A2 | na |  |  | 1323 | 1.405 | 0.0484 | No |
| 12 | PVT1 | na |  |  | 1325 | 1.405 | 0.0574 | No |
| 13 | AEN | na |  |  | 1410 | 1.373 | 0.0618 | No |
| 14 | SESN1 | na |  |  | 1467 | 1.357 | 0.0676 | No |
| 15 | SLC7A11 | na |  |  | 1471 | 1.356 | 0.0761 | No |
| 16 | TPRKB | na |  |  | 1534 | 1.338 | 0.0814 | No |
| 17 | NOL8 | na |  |  | 1538 | 1.336 | 0.0898 | No |
| 18 | PCNA | na |  |  | 1712 | 1.291 | 0.0890 | No |
| 19 | VDR | na |  |  | 1758 | 1.278 | 0.0949 | No |
| 20 | OSGIN1 | na |  |  | 1862 | 1.254 | 0.0975 | No |
| 21 | SFN | na |  |  | 1900 | 1.246 | 0.1036 | No |
| 22 | PTPN14 | na |  |  | 1962 | 1.232 | 0.1083 | No |
| 23 | CCND3 | na |  |  | 2241 | 1.169 | 0.1012 | No |
| 24 | CCP110 | na |  |  | 2292 | 1.159 | 0.1060 | No |
| 25 | DDB2 | na |  |  | 2488 | 1.113 | 0.1029 | No |
| 26 | TAX1BP3 | na |  |  | 2508 | 1.109 | 0.1090 | No |
| 27 | EI24 | na |  |  | 2556 | 1.100 | 0.1136 | No |
| 28 | UPP1 | na |  |  | 2597 | 1.095 | 0.1185 | No |
| 29 | FBXW7 | na |  |  | 2687 | 1.079 | 0.1207 | No |
| 30 | PDGFA | na |  |  | 2978 | 1.030 | 0.1121 | No |
| 31 | HMOX1 | na |  |  | 3011 | 1.026 | 0.1170 | No |
| 32 | SLC35D1 | na |  |  | 3115 | 1.004 | 0.1180 | No |
| 33 | POLH | na |  |  | 3221 | 0.982 | 0.1188 | No |
| 34 | MDM2 | na |  |  | 3313 | 0.968 | 0.1202 | No |
| 35 | HBEGF | na |  |  | 3593 | 0.921 | 0.1115 | No |
| 36 | TRAF4 | na |  |  | 3885 | 0.870 | 0.1018 | No |
| 37 | BLCAP | na |  |  | 3904 | 0.867 | 0.1064 | No |
| 38 | RPL18 | na |  |  | 4032 | 0.846 | 0.1052 | No |
| 39 | HINT1 | na |  |  | 4302 | 0.808 | 0.0962 | No |
| 40 | PLK2 | na |  |  | 4344 | 0.801 | 0.0992 | No |
| 41 | TRIAP1 | na |  |  | 4622 | 0.760 | 0.0895 | No |
| 42 | ZMAT3 | na |  |  | 4916 | 0.717 | 0.0787 | No |
| 43 | GPX2 | na |  |  | 4929 | 0.716 | 0.0827 | No |
| 44 | IER5 | na |  |  | 4968 | 0.712 | 0.0852 | No |
| 45 | ABCC5 | na |  |  | 5023 | 0.704 | 0.0869 | No |
| 46 | RB1 | na |  |  | 5179 | 0.681 | 0.0831 | No |
| 47 | HDAC3 | na |  |  | 5394 | 0.651 | 0.0761 | No |
| 48 | HRAS | na |  |  | 5618 | 0.623 | 0.0683 | No |
| 49 | CDK5R1 | na |  |  | 5732 | 0.605 | 0.0663 | No |
| 50 | CDKN2AIP | na |  |  | 5824 | 0.594 | 0.0653 | No |
| 51 | CCNK | na |  |  | 5907 | 0.582 | 0.0647 | No |
| 52 | BAIAP2 | na |  |  | 6000 | 0.570 | 0.0636 | No |
| 53 | PRKAB1 | na |  |  | 6061 | 0.563 | 0.0640 | No |
| 54 | GADD45A | na |  |  | 6102 | 0.555 | 0.0655 | No |
| 55 | WWP1 | na |  |  | 6265 | 0.532 | 0.0604 | No |
| 56 | FAS | na |  |  | 6266 | 0.532 | 0.0638 | No |
| 57 | PROCR | na |  |  | 6383 | 0.516 | 0.0610 | No |
| 58 | DNTTIP2 | na |  |  | 6419 | 0.512 | 0.0624 | No |
| 59 | TM7SF3 | na |  |  | 6479 | 0.506 | 0.0626 | No |
| 60 | ADA | na |  |  | 6574 | 0.493 | 0.0608 | No |
| 61 | RAP2B | na |  |  | 6651 | 0.485 | 0.0599 | No |
| 62 | POM121 | na |  |  | 6656 | 0.484 | 0.0628 | No |
| 63 | SEC61A1 | na |  |  | 6734 | 0.476 | 0.0618 | No |
| 64 | DDIT3 | na |  |  | 6794 | 0.469 | 0.0617 | No |
| 65 | CGRRF1 | na |  |  | 7022 | 0.444 | 0.0527 | No |
| 66 | WRAP73 | na |  |  | 7079 | 0.436 | 0.0525 | No |
| 67 | RRP8 | na |  |  | 7331 | 0.404 | 0.0419 | No |
| 68 | ATF3 | na |  |  | 7410 | 0.395 | 0.0403 | No |
| 69 | F2R | na |  |  | 7430 | 0.393 | 0.0419 | No |
| 70 | EPHA2 | na |  |  | 7485 | 0.387 | 0.0415 | No |
| 71 | FDXR | na |  |  | 7707 | 0.362 | 0.0322 | No |
| 72 | SDC1 | na |  |  | 7964 | 0.330 | 0.0209 | No |
| 73 | BAX | na |  |  | 8092 | 0.317 | 0.0162 | No |
| 74 | PLK3 | na |  |  | 8645 | 0.255 | -0.0112 | No |
| 75 | PPP1R15A | na |  |  | 8701 | 0.246 | -0.0125 | No |
| 76 | APAF1 | na |  |  | 9058 | 0.207 | -0.0299 | No |
| 77 | FOXO3 | na |  |  | 9106 | 0.201 | -0.0310 | No |
| 78 | TNFSF9 | na |  |  | 9187 | 0.193 | -0.0340 | No |
| 79 | EPS8L2 | na |  |  | 9238 | 0.189 | -0.0354 | No |
| 80 | TPD52L1 | na |  |  | 9466 | 0.161 | -0.0463 | No |
| 81 | MAPKAPK3 | na |  |  | 9583 | 0.147 | -0.0515 | No |
| 82 | SP1 | na |  |  | 9717 | 0.135 | -0.0576 | No |
| 83 | RPS27L | na |  |  | 10138 | 0.091 | -0.0791 | No |
| 84 | DCXR | na |  |  | 10151 | 0.088 | -0.0792 | No |
| 85 | ACVR1B | na |  |  | 10312 | 0.067 | -0.0871 | No |
| 86 | CASP1 | na |  |  | 10391 | 0.060 | -0.0909 | No |
| 87 | KRT17 | na |  |  | 10421 | 0.060 | -0.0920 | No |
| 88 | PPM1D | na |  |  | 10558 | 0.045 | -0.0989 | No |
| 89 | ERCC5 | na |  |  | 10584 | 0.040 | -0.0999 | No |
| 90 | CSRNP2 | na |  |  | 11009 | -0.004 | -0.1222 | No |
| 91 | RAD9A | na |  |  | 11166 | -0.022 | -0.1302 | No |
| 92 | RPS12 | na |  |  | 11442 | -0.055 | -0.1444 | No |
| 93 | INHBB | na |  |  | 11465 | -0.058 | -0.1451 | No |
| 94 | TCHH | na |  |  | 11472 | -0.059 | -0.1451 | No |
| 95 | VWA5A | na |  |  | 11539 | -0.062 | -0.1481 | No |
| 96 | BTG2 | na |  |  | 11938 | -0.118 | -0.1683 | No |
| 97 | TGFA | na |  |  | 12260 | -0.151 | -0.1842 | No |
| 98 | HIST1H1C | na |  |  | 12346 | -0.161 | -0.1877 | No |
| 99 | RETSAT | na |  |  | 12351 | -0.162 | -0.1868 | No |
| 100 | ST14 | na |  |  | 12353 | -0.163 | -0.1858 | No |
| 101 | JUN | na |  |  | 12605 | -0.199 | -0.1978 | No |
| 102 | IP6K2 | na |  |  | 12715 | -0.217 | -0.2021 | No |
| 103 | BAK1 | na |  |  | 12720 | -0.218 | -0.2009 | No |
| 104 | LIF | na |  |  | 12907 | -0.232 | -0.2092 | No |
| 105 | PMM1 | na |  |  | 12923 | -0.235 | -0.2085 | No |
| 106 | RXRA | na |  |  | 13249 | -0.281 | -0.2238 | No |
| 107 | STEAP3 | na |  |  | 13310 | -0.289 | -0.2251 | No |
| 108 | RAB40C | na |  |  | 13358 | -0.296 | -0.2256 | No |
| 109 | NOTCH1 | na |  |  | 13411 | -0.304 | -0.2264 | No |
| 110 | RGS16 | na |  |  | 13422 | -0.305 | -0.2250 | No |
| 111 | H2AFJ | na |  |  | 13564 | -0.325 | -0.2303 | No |
| 112 | IFI30 | na |  |  | 13832 | -0.356 | -0.2421 | No |
| 113 | ISCU | na |  |  | 13836 | -0.357 | -0.2399 | No |
| 114 | TOB1 | na |  |  | 14149 | -0.399 | -0.2538 | No |
| 115 | TP53 | na |  |  | 14267 | -0.417 | -0.2572 | No |
| 116 | CCNG1 | na |  |  | 14380 | -0.429 | -0.2604 | No |
| 117 | RNF19B | na |  |  | 14473 | -0.441 | -0.2624 | No |
| 118 | PRMT2 | na |  |  | 14973 | -0.510 | -0.2853 | No |
| 119 | RPL36 | na |  |  | 15275 | -0.559 | -0.2976 | No |
| 120 | JAG2 | na |  |  | 15291 | -0.563 | -0.2948 | No |
| 121 | MXD1 | na |  |  | 15373 | -0.577 | -0.2953 | No |
| 122 | ANKRA2 | na |  |  | 15465 | -0.593 | -0.2963 | No |
| 123 | KIF13B | na |  |  | 15539 | -0.601 | -0.2963 | No |
| 124 | DGKA | na |  |  | 15679 | -0.622 | -0.2996 | No |
| 125 | CYFIP2 | na |  |  | 15765 | -0.639 | -0.3000 | No |
| 126 | EPHX1 | na |  |  | 15777 | -0.644 | -0.2964 | No |
| 127 | CDKN1A | na |  |  | 15950 | -0.678 | -0.3011 | Yes |
| 128 | TRAFD1 | na |  |  | 15985 | -0.683 | -0.2985 | Yes |
| 129 | IL1A | na |  |  | 16094 | -0.702 | -0.2997 | Yes |
| 130 | PLXNB2 | na |  |  | 16127 | -0.711 | -0.2968 | Yes |
| 131 | CD81 | na |  |  | 16248 | -0.744 | -0.2983 | Yes |
| 132 | TRIB3 | na |  |  | 16278 | -0.753 | -0.2950 | Yes |
| 133 | DRAM1 | na |  |  | 16350 | -0.769 | -0.2938 | Yes |
| 134 | SOCS1 | na |  |  | 16397 | -0.781 | -0.2912 | Yes |
| 135 | KLF4 | na |  |  | 16438 | -0.793 | -0.2882 | Yes |
| 136 | ABHD4 | na |  |  | 16446 | -0.795 | -0.2835 | Yes |
| 137 | NINJ1 | na |  |  | 16489 | -0.808 | -0.2805 | Yes |
| 138 | SERTAD3 | na |  |  | 16496 | -0.810 | -0.2756 | Yes |
| 139 | AK1 | na |  |  | 16503 | -0.811 | -0.2707 | Yes |
| 140 | GM2A | na |  |  | 16868 | -0.911 | -0.2840 | Yes |
| 141 | CTSD | na |  |  | 16910 | -0.924 | -0.2803 | Yes |
| 142 | HEXIM1 | na |  |  | 16968 | -0.940 | -0.2772 | Yes |
| 143 | TM4SF1 | na |  |  | 17063 | -0.967 | -0.2759 | Yes |
| 144 | FAM162A | na |  |  | 17146 | -0.993 | -0.2739 | Yes |
| 145 | PERP | na |  |  | 17157 | -0.998 | -0.2680 | Yes |
| 146 | TCN2 | na |  |  | 17251 | -1.022 | -0.2663 | Yes |
| 147 | ZFP36L1 | na |  |  | 17278 | -1.033 | -0.2611 | Yes |
| 148 | CCND2 | na |  |  | 17385 | -1.070 | -0.2598 | Yes |
| 149 | TAP1 | na |  |  | 17413 | -1.084 | -0.2542 | Yes |
| 150 | RHBDF2 | na |  |  | 17423 | -1.090 | -0.2477 | Yes |
| 151 | TP63 | na |  |  | 17462 | -1.110 | -0.2426 | Yes |
| 152 | TGFB1 | na |  |  | 17599 | -1.148 | -0.2424 | Yes |
| 153 | FOS | na |  |  | 17698 | -1.185 | -0.2399 | Yes |
| 154 | DDIT4 | na |  |  | 17752 | -1.202 | -0.2350 | Yes |
| 155 | RRAD | na |  |  | 17756 | -1.204 | -0.2274 | Yes |
| 156 | PTPRE | na |  |  | 17804 | -1.225 | -0.2220 | Yes |
| 157 | CD82 | na |  |  | 17854 | -1.256 | -0.2165 | Yes |
| 158 | PHLDA3 | na |  |  | 17894 | -1.271 | -0.2104 | Yes |
| 159 | TSPYL2 | na |  |  | 17932 | -1.291 | -0.2041 | Yes |
| 160 | S100A10 | na |  |  | 18028 | -1.345 | -0.2004 | Yes |
| 161 | ITGB4 | na |  |  | 18146 | -1.397 | -0.1976 | Yes |
| 162 | MXD4 | na |  |  | 18207 | -1.429 | -0.1916 | Yes |
| 163 | MKNK2 | na |  |  | 18310 | -1.504 | -0.1873 | Yes |
| 164 | PITPNC1 | na |  |  | 18321 | -1.507 | -0.1782 | Yes |
| 165 | APP | na |  |  | 18352 | -1.528 | -0.1699 | Yes |
| 166 | XPC | na |  |  | 18373 | -1.542 | -0.1611 | Yes |
| 167 | RALGDS | na |  |  | 18413 | -1.583 | -0.1530 | Yes |
| 168 | NUPR1 | na |  |  | 18454 | -1.613 | -0.1447 | Yes |
| 169 | TSC22D1 | na |  |  | 18536 | -1.680 | -0.1382 | Yes |
| 170 | TXNIP | na |  |  | 18572 | -1.710 | -0.1291 | Yes |
| 171 | BTG1 | na |  |  | 18836 | -2.149 | -0.1291 | Yes |
| 172 | FUCA1 | na |  |  | 18883 | -2.288 | -0.1168 | Yes |
| 173 | ABAT | na |  |  | 18938 | -2.397 | -0.1043 | Yes |
| 174 | VAMP8 | na |  |  | 19018 | -2.739 | -0.0909 | Yes |
| 175 | CTSF | na |  |  | 19041 | -2.834 | -0.0738 | Yes |
| 176 | S100A4 | na |  |  | 19044 | -2.881 | -0.0554 | Yes |
| 177 | NDRG1 | na |  |  | 19083 | -3.028 | -0.0380 | Yes |
| 178 | BMP2 | na |  |  | 19119 | -3.339 | -0.0184 | Yes |
| 179 | SAT1 | na |  |  | 19139 | -3.506 | 0.0031 | Yes |
Table: GSEA details [plain text format]

  

Fig 2: HALLMARK\_P53\_PATHWAY      
 Blue-Pink O' Gram in the Space of the Analyzed GeneSet

  

Fig 3: HALLMARK\_P53\_PATHWAY: Random ES distribution      
 Gene set null distribution of ES for **HALLMARK\_P53\_PATHWAY**

  
