# Supplementary material for: Heparin-based hydrogel scaffolding alters the transcriptomic profile and increases the chemoresistance of MDA-MB-231 triple-negative breast cancer cells
Source: Biomater Sci. 2020 Feb 13;8(10):2786–96. doi: 10.1039/c9bm01481k (PMC7497406; doi:10.1039/c9bm01481k)
Supplement: Supplementary file 2 [file BM-008-C9BM01481K-s002.zip › Supplementary File 4/EGFvControl/Pathways/my_analysis.Gsea.1545200981068/HALLMARK_SPERMATOGENESIS.html]

Details for gene set HALLMARK\_SPERMATOGENESIS[GSEA]

|  || Dataset | expr.class.cls#EGF\_versus\_CONTROL.class.cls#EGF\_versus\_CONTROL\_repos |
| Phenotype | class.cls#EGF\_versus\_CONTROL\_repos |
| Upregulated in class | EGF |
| GeneSet | HALLMARK\_SPERMATOGENESIS |
| Enrichment Score (ES) | 0.44994524 |
| Normalized Enrichment Score (NES) | 1.7951754 |
| Nominal p-value | 0.0 |
| FDR q-value | 0.0014986225 |
| FWER p-Value | 0.013 |
Table: GSEA Results Summary

  

Fig 1: Enrichment plot: HALLMARK\_SPERMATOGENESIS      
 Profile of the Running ES Score & Positions of GeneSet Members on the Rank Ordered List

  

| PROBE | DESCRIPTION (from dataset) | GENE SYMBOL | GENE\_TITLE | RANK IN GENE LIST | RANK METRIC SCORE | RUNNING ES | CORE ENRICHMENT || 1 | SCG5 | na |  |  | 24 | 2.939 | 0.0425 | Yes |
| 2 | AURKA | na |  |  | 163 | 2.181 | 0.0677 | Yes |
| 3 | EZH2 | na |  |  | 227 | 2.053 | 0.0950 | Yes |
| 4 | CCNA1 | na |  |  | 344 | 1.897 | 0.1171 | Yes |
| 5 | BUB1 | na |  |  | 455 | 1.797 | 0.1381 | Yes |
| 6 | CDKN3 | na |  |  | 562 | 1.729 | 0.1583 | Yes |
| 7 | CAMK4 | na |  |  | 952 | 1.529 | 0.1607 | Yes |
| 8 | HSPA4L | na |  |  | 1129 | 1.468 | 0.1733 | Yes |
| 9 | PSMG1 | na |  |  | 1201 | 1.441 | 0.1911 | Yes |
| 10 | ZC3H14 | na |  |  | 1317 | 1.407 | 0.2060 | Yes |
| 11 | CSNK2A2 | na |  |  | 1406 | 1.376 | 0.2219 | Yes |
| 12 | RFC4 | na |  |  | 1417 | 1.372 | 0.2417 | Yes |
| 13 | PRKAR2A | na |  |  | 1476 | 1.353 | 0.2588 | Yes |
| 14 | PARP2 | na |  |  | 1635 | 1.310 | 0.2701 | Yes |
| 15 | TOPBP1 | na |  |  | 1682 | 1.299 | 0.2870 | Yes |
| 16 | SLC12A2 | na |  |  | 1686 | 1.297 | 0.3061 | Yes |
| 17 | NF2 | na |  |  | 1754 | 1.279 | 0.3217 | Yes |
| 18 | NCAPH | na |  |  | 1763 | 1.276 | 0.3402 | Yes |
| 19 | STRBP | na |  |  | 1911 | 1.244 | 0.3511 | Yes |
| 20 | KIF2C | na |  |  | 1975 | 1.227 | 0.3660 | Yes |
| 21 | TSN | na |  |  | 2099 | 1.196 | 0.3774 | Yes |
| 22 | DBF4 | na |  |  | 2587 | 1.096 | 0.3682 | Yes |
| 23 | CDK1 | na |  |  | 2921 | 1.040 | 0.3663 | Yes |
| 24 | CHFR | na |  |  | 2953 | 1.034 | 0.3801 | Yes |
| 25 | CLGN | na |  |  | 3109 | 1.005 | 0.3869 | Yes |
| 26 | NEK2 | na |  |  | 3222 | 0.981 | 0.3957 | Yes |
| 27 | TTK | na |  |  | 3293 | 0.971 | 0.4065 | Yes |
| 28 | VDAC3 | na |  |  | 3332 | 0.965 | 0.4188 | Yes |
| 29 | CLPB | na |  |  | 3576 | 0.924 | 0.4199 | Yes |
| 30 | MTOR | na |  |  | 3590 | 0.922 | 0.4329 | Yes |
| 31 | RAD17 | na |  |  | 3623 | 0.917 | 0.4449 | Yes |
| 32 | IFT88 | na |  |  | 3780 | 0.888 | 0.4499 | Yes |
| 33 | ARL4A | na |  |  | 4169 | 0.826 | 0.4419 | No |
| 34 | PIAS2 | na |  |  | 4288 | 0.810 | 0.4478 | No |
| 35 | MLF1 | na |  |  | 5002 | 0.708 | 0.4211 | No |
| 36 | CCNB2 | na |  |  | 5148 | 0.685 | 0.4237 | No |
| 37 | STAM2 | na |  |  | 5658 | 0.616 | 0.4062 | No |
| 38 | AGFG1 | na |  |  | 6017 | 0.568 | 0.3960 | No |
| 39 | PHKG2 | na |  |  | 6561 | 0.495 | 0.3749 | No |
| 40 | COIL | na |  |  | 6680 | 0.481 | 0.3759 | No |
| 41 | PGS1 | na |  |  | 6790 | 0.469 | 0.3772 | No |
| 42 | ACRV1 | na |  |  | 6969 | 0.449 | 0.3746 | No |
| 43 | GMCL1 | na |  |  | 7438 | 0.393 | 0.3559 | No |
| 44 | MLLT10 | na |  |  | 7939 | 0.333 | 0.3348 | No |
| 45 | GAD1 | na |  |  | 7981 | 0.328 | 0.3375 | No |
| 46 | BRAF | na |  |  | 8058 | 0.320 | 0.3383 | No |
| 47 | IDE | na |  |  | 8196 | 0.305 | 0.3357 | No |
| 48 | IP6K1 | na |  |  | 8456 | 0.278 | 0.3262 | No |
| 49 | SIRT1 | na |  |  | 8598 | 0.262 | 0.3228 | No |
| 50 | MAST2 | na |  |  | 8707 | 0.245 | 0.3208 | No |
| 51 | RPL39L | na |  |  | 8904 | 0.223 | 0.3138 | No |
| 52 | TCP11 | na |  |  | 9154 | 0.196 | 0.3037 | No |
| 53 | HSPA2 | na |  |  | 9174 | 0.194 | 0.3056 | No |
| 54 | TALDO1 | na |  |  | 9239 | 0.189 | 0.3051 | No |
| 55 | DDX25 | na |  |  | 9359 | 0.177 | 0.3015 | No |
| 56 | NEFH | na |  |  | 10044 | 0.100 | 0.2672 | No |
| 57 | PHF7 | na |  |  | 10190 | 0.083 | 0.2609 | No |
| 58 | JAM3 | na |  |  | 10852 | 0.007 | 0.2264 | No |
| 59 | PEBP1 | na |  |  | 11723 | -0.089 | 0.1823 | No |
| 60 | CCT6B | na |  |  | 12185 | -0.142 | 0.1603 | No |
| 61 | OAZ3 | na |  |  | 12838 | -0.231 | 0.1296 | No |
| 62 | SNAP91 | na |  |  | 13588 | -0.329 | 0.0953 | No |
| 63 | GFI1 | na |  |  | 13732 | -0.349 | 0.0930 | No |
| 64 | TLE4 | na |  |  | 14004 | -0.378 | 0.0845 | No |
| 65 | PAPOLB | na |  |  | 14081 | -0.390 | 0.0863 | No |
| 66 | GPR182 | na |  |  | 14169 | -0.401 | 0.0877 | No |
| 67 | MAP7 | na |  |  | 14501 | -0.444 | 0.0771 | No |
| 68 | PCSK4 | na |  |  | 14926 | -0.504 | 0.0624 | No |
| 69 | HSPA1L | na |  |  | 15666 | -0.618 | 0.0329 | No |
| 70 | GSTM3 | na |  |  | 16973 | -0.943 | -0.0213 | No |
| 71 | PCSK1N | na |  |  | 17151 | -0.995 | -0.0158 | No |
| 72 | SLC2A5 | na |  |  | 17292 | -1.039 | -0.0076 | No |
| 73 | NPHP1 | na |  |  | 17909 | -1.281 | -0.0208 | No |
| 74 | LPIN1 | na |  |  | 18020 | -1.341 | -0.0066 | No |
| 75 | CNIH2 | na |  |  | 18118 | -1.383 | 0.0089 | No |
| 76 | SEPT4 | na |  |  | 19104 | -3.193 | 0.0049 | No |
Table: GSEA details [plain text format]

  

Fig 2: HALLMARK\_SPERMATOGENESIS      
 Blue-Pink O' Gram in the Space of the Analyzed GeneSet

  

Fig 3: HALLMARK\_SPERMATOGENESIS: Random ES distribution      
 Gene set null distribution of ES for **HALLMARK\_SPERMATOGENESIS**

  
