# Supplementary material for: Heparin-based hydrogel scaffolding alters the transcriptomic profile and increases the chemoresistance of MDA-MB-231 triple-negative breast cancer cells
Source: Biomater Sci. 2020 Feb 13;8(10):2786–96. doi: 10.1039/c9bm01481k (PMC7497406; doi:10.1039/c9bm01481k)
Supplement: Supplementary file 2 [file BM-008-C9BM01481K-s002.zip › Supplementary File 4/EGFvControl/Pathways/my_analysis.Gsea.1545200981068/HALLMARK_PANCREAS_BETA_CELLS.html]

Details for gene set HALLMARK\_PANCREAS\_BETA\_CELLS[GSEA]

|  || Dataset | expr.class.cls#EGF\_versus\_CONTROL.class.cls#EGF\_versus\_CONTROL\_repos |
| Phenotype | class.cls#EGF\_versus\_CONTROL\_repos |
| Upregulated in class | EGF |
| GeneSet | HALLMARK\_PANCREAS\_BETA\_CELLS |
| Enrichment Score (ES) | 0.25334525 |
| Normalized Enrichment Score (NES) | 0.7498823 |
| Nominal p-value | 0.81895095 |
| FDR q-value | 0.9385138 |
| FWER p-Value | 1.0 |
Table: GSEA Results Summary

  

Fig 1: Enrichment plot: HALLMARK\_PANCREAS\_BETA\_CELLS      
 Profile of the Running ES Score & Positions of GeneSet Members on the Rank Ordered List

  

| PROBE | DESCRIPTION (from dataset) | GENE SYMBOL | GENE\_TITLE | RANK IN GENE LIST | RANK METRIC SCORE | RUNNING ES | CORE ENRICHMENT || 1 | SRPRB | na |  |  | 436 | 1.815 | 0.1142 | Yes |
| 2 | VDR | na |  |  | 1758 | 1.278 | 0.1418 | Yes |
| 3 | AKT3 | na |  |  | 3603 | 0.919 | 0.1150 | Yes |
| 4 | SRP9 | na |  |  | 3710 | 0.902 | 0.1776 | Yes |
| 5 | SEC11A | na |  |  | 3889 | 0.870 | 0.2339 | Yes |
| 6 | ELP4 | na |  |  | 4618 | 0.761 | 0.2533 | Yes |
| 7 | NKX6-1 | na |  |  | 7200 | 0.422 | 0.1506 | No |
| 8 | SPCS1 | na |  |  | 7349 | 0.402 | 0.1733 | No |
| 9 | CHGA | na |  |  | 8624 | 0.257 | 0.1263 | No |
| 10 | LMO2 | na |  |  | 9839 | 0.122 | 0.0722 | No |
| 11 | PCSK1 | na |  |  | 12341 | -0.161 | -0.0461 | No |
| 12 | STXBP1 | na |  |  | 12647 | -0.205 | -0.0465 | No |
| 13 | SYT13 | na |  |  | 13647 | -0.340 | -0.0729 | No |
| 14 | MAFB | na |  |  | 14481 | -0.442 | -0.0830 | No |
| 15 | DPP4 | na |  |  | 14594 | -0.462 | -0.0540 | No |
| 16 | FOXA2 | na |  |  | 17421 | -1.089 | -0.1191 | No |
| 17 | FOXO1 | na |  |  | 17953 | -1.306 | -0.0483 | No |
| 18 | SRP14 | na |  |  | 18305 | -1.500 | 0.0466 | No |
Table: GSEA details [plain text format]

  

Fig 2: HALLMARK\_PANCREAS\_BETA\_CELLS      
 Blue-Pink O' Gram in the Space of the Analyzed GeneSet

  

Fig 3: HALLMARK\_PANCREAS\_BETA\_CELLS: Random ES distribution      
 Gene set null distribution of ES for **HALLMARK\_PANCREAS\_BETA\_CELLS**

  
