# Supplementary material for: Heparin-based hydrogel scaffolding alters the transcriptomic profile and increases the chemoresistance of MDA-MB-231 triple-negative breast cancer cells
Source: Biomater Sci. 2020 Feb 13;8(10):2786–96. doi: 10.1039/c9bm01481k (PMC7497406; doi:10.1039/c9bm01481k)
Supplement: Supplementary file 2 [file BM-008-C9BM01481K-s002.zip › Supplementary File 4/EGFvControl/Pathways/my_analysis.Gsea.1545200981068/HALLMARK_OXIDATIVE_PHOSPHORYLATION.html]

Details for gene set HALLMARK\_OXIDATIVE\_PHOSPHORYLATION[GSEA]

|  || Dataset | expr.class.cls#EGF\_versus\_CONTROL.class.cls#EGF\_versus\_CONTROL\_repos |
| Phenotype | class.cls#EGF\_versus\_CONTROL\_repos |
| Upregulated in class | EGF |
| GeneSet | HALLMARK\_OXIDATIVE\_PHOSPHORYLATION |
| Enrichment Score (ES) | 0.34651607 |
| Normalized Enrichment Score (NES) | 1.6162214 |
| Nominal p-value | 0.0 |
| FDR q-value | 0.006645646 |
| FWER p-Value | 0.101 |
Table: GSEA Results Summary

  

Fig 1: Enrichment plot: HALLMARK\_OXIDATIVE\_PHOSPHORYLATION      
 Profile of the Running ES Score & Positions of GeneSet Members on the Rank Ordered List

  

| PROBE | DESCRIPTION (from dataset) | GENE SYMBOL | GENE\_TITLE | RANK IN GENE LIST | RANK METRIC SCORE | RUNNING ES | CORE ENRICHMENT || 1 | MTRR | na |  |  | 208 | 2.081 | 0.0035 | Yes |
| 2 | ATP5G1 | na |  |  | 281 | 1.978 | 0.0135 | Yes |
| 3 | NDUFA9 | na |  |  | 409 | 1.840 | 0.0196 | Yes |
| 4 | LDHB | na |  |  | 598 | 1.705 | 0.0215 | Yes |
| 5 | SLC25A5 | na |  |  | 601 | 1.704 | 0.0333 | Yes |
| 6 | AIFM1 | na |  |  | 634 | 1.679 | 0.0433 | Yes |
| 7 | FH | na |  |  | 638 | 1.677 | 0.0548 | Yes |
| 8 | TIMM8B | na |  |  | 683 | 1.654 | 0.0640 | Yes |
| 9 | IDH3A | na |  |  | 787 | 1.595 | 0.0696 | Yes |
| 10 | SLC25A20 | na |  |  | 788 | 1.595 | 0.0807 | Yes |
| 11 | TOMM22 | na |  |  | 984 | 1.518 | 0.0810 | Yes |
| 12 | COX10 | na |  |  | 1252 | 1.427 | 0.0769 | Yes |
| 13 | HSPA9 | na |  |  | 1253 | 1.427 | 0.0868 | Yes |
| 14 | MDH1 | na |  |  | 1318 | 1.406 | 0.0932 | Yes |
| 15 | MRPS15 | na |  |  | 1571 | 1.328 | 0.0892 | Yes |
| 16 | COX8A | na |  |  | 1597 | 1.318 | 0.0970 | Yes |
| 17 | GOT2 | na |  |  | 1607 | 1.316 | 0.1057 | Yes |
| 18 | NDUFB3 | na |  |  | 1632 | 1.311 | 0.1135 | Yes |
| 19 | TIMM9 | na |  |  | 1658 | 1.306 | 0.1213 | Yes |
| 20 | TIMM13 | na |  |  | 1730 | 1.287 | 0.1265 | Yes |
| 21 | ATP5A1 | na |  |  | 1816 | 1.263 | 0.1308 | Yes |
| 22 | SUCLA2 | na |  |  | 1863 | 1.254 | 0.1371 | Yes |
| 23 | SLC25A3 | na |  |  | 1903 | 1.246 | 0.1437 | Yes |
| 24 | TIMM17A | na |  |  | 1944 | 1.237 | 0.1502 | Yes |
| 25 | COX7A2L | na |  |  | 1988 | 1.223 | 0.1564 | Yes |
| 26 | COX15 | na |  |  | 2029 | 1.213 | 0.1628 | Yes |
| 27 | NDUFC2 | na |  |  | 2146 | 1.189 | 0.1649 | Yes |
| 28 | OAT | na |  |  | 2217 | 1.174 | 0.1694 | Yes |
| 29 | PMPCA | na |  |  | 2232 | 1.172 | 0.1768 | Yes |
| 30 | PDHA1 | na |  |  | 2270 | 1.162 | 0.1829 | Yes |
| 31 | AFG3L2 | na |  |  | 2288 | 1.159 | 0.1901 | Yes |
| 32 | ATP5G3 | na |  |  | 2433 | 1.126 | 0.1903 | Yes |
| 33 | OPA1 | na |  |  | 2447 | 1.124 | 0.1975 | Yes |
| 34 | ABCB7 | na |  |  | 2464 | 1.120 | 0.2044 | Yes |
| 35 | NDUFB2 | na |  |  | 2497 | 1.112 | 0.2105 | Yes |
| 36 | MRPL35 | na |  |  | 2575 | 1.098 | 0.2140 | Yes |
| 37 | PHB2 | na |  |  | 2720 | 1.073 | 0.2139 | Yes |
| 38 | FXN | na |  |  | 2809 | 1.059 | 0.2167 | Yes |
| 39 | ATP5B | na |  |  | 2824 | 1.057 | 0.2233 | Yes |
| 40 | CS | na |  |  | 2862 | 1.050 | 0.2286 | Yes |
| 41 | TIMM10 | na |  |  | 2871 | 1.048 | 0.2355 | Yes |
| 42 | MFN2 | na |  |  | 2931 | 1.037 | 0.2396 | Yes |
| 43 | ETFA | na |  |  | 2935 | 1.036 | 0.2466 | Yes |
| 44 | NDUFS3 | na |  |  | 2961 | 1.032 | 0.2525 | Yes |
| 45 | ATP5F1 | na |  |  | 2967 | 1.031 | 0.2594 | Yes |
| 46 | MRPS12 | na |  |  | 3065 | 1.013 | 0.2613 | Yes |
| 47 | LRPPRC | na |  |  | 3083 | 1.010 | 0.2675 | Yes |
| 48 | SLC25A11 | na |  |  | 3086 | 1.009 | 0.2744 | Yes |
| 49 | MRPL11 | na |  |  | 3216 | 0.983 | 0.2744 | Yes |
| 50 | ACAT1 | na |  |  | 3228 | 0.981 | 0.2806 | Yes |
| 51 | VDAC3 | na |  |  | 3332 | 0.965 | 0.2819 | Yes |
| 52 | GRPEL1 | na |  |  | 3510 | 0.935 | 0.2791 | Yes |
| 53 | FDX1 | na |  |  | 3610 | 0.918 | 0.2803 | Yes |
| 54 | HSD17B10 | na |  |  | 3686 | 0.907 | 0.2827 | Yes |
| 55 | PRDX3 | na |  |  | 3704 | 0.903 | 0.2880 | Yes |
| 56 | VDAC1 | na |  |  | 3760 | 0.892 | 0.2913 | Yes |
| 57 | NDUFV2 | na |  |  | 3802 | 0.883 | 0.2953 | Yes |
| 58 | DLD | na |  |  | 3868 | 0.873 | 0.2980 | Yes |
| 59 | ATP6V1H | na |  |  | 3874 | 0.872 | 0.3038 | Yes |
| 60 | NNT | na |  |  | 4026 | 0.847 | 0.3017 | Yes |
| 61 | SDHD | na |  |  | 4053 | 0.843 | 0.3062 | Yes |
| 62 | MRPS30 | na |  |  | 4076 | 0.839 | 0.3109 | Yes |
| 63 | TIMM50 | na |  |  | 4077 | 0.839 | 0.3167 | Yes |
| 64 | UQCRQ | na |  |  | 4082 | 0.839 | 0.3223 | Yes |
| 65 | DLAT | na |  |  | 4235 | 0.817 | 0.3200 | Yes |
| 66 | CYCS | na |  |  | 4292 | 0.810 | 0.3227 | Yes |
| 67 | ATP6V1D | na |  |  | 4550 | 0.771 | 0.3145 | Yes |
| 68 | SLC25A4 | na |  |  | 4552 | 0.770 | 0.3198 | Yes |
| 69 | IMMT | na |  |  | 4595 | 0.765 | 0.3229 | Yes |
| 70 | SUPV3L1 | na |  |  | 4635 | 0.758 | 0.3262 | Yes |
| 71 | PDHX | na |  |  | 4699 | 0.749 | 0.3280 | Yes |
| 72 | CYC1 | na |  |  | 4803 | 0.729 | 0.3277 | Yes |
| 73 | NDUFA5 | na |  |  | 4854 | 0.722 | 0.3301 | Yes |
| 74 | MRPS11 | na |  |  | 4872 | 0.720 | 0.3342 | Yes |
| 75 | ATP6V1F | na |  |  | 5085 | 0.696 | 0.3279 | Yes |
| 76 | ACAA1 | na |  |  | 5086 | 0.695 | 0.3327 | Yes |
| 77 | NDUFA4 | na |  |  | 5207 | 0.678 | 0.3311 | Yes |
| 78 | MTX2 | na |  |  | 5249 | 0.672 | 0.3336 | Yes |
| 79 | MRPS22 | na |  |  | 5280 | 0.668 | 0.3367 | Yes |
| 80 | ISCA1 | na |  |  | 5371 | 0.654 | 0.3365 | Yes |
| 81 | ATP5O | na |  |  | 5449 | 0.645 | 0.3369 | Yes |
| 82 | GLUD1 | na |  |  | 5455 | 0.643 | 0.3411 | Yes |
| 83 | NDUFB5 | na |  |  | 5460 | 0.643 | 0.3454 | Yes |
| 84 | SDHA | na |  |  | 5594 | 0.627 | 0.3428 | Yes |
| 85 | CASP7 | na |  |  | 5606 | 0.625 | 0.3465 | Yes |
| 86 | COX11 | na |  |  | 6029 | 0.567 | 0.3283 | No |
| 87 | ATP5L | na |  |  | 6182 | 0.544 | 0.3240 | No |
| 88 | UQCRC2 | na |  |  | 6295 | 0.528 | 0.3218 | No |
| 89 | HADHA | na |  |  | 6329 | 0.523 | 0.3237 | No |
| 90 | UQCRC1 | na |  |  | 6365 | 0.518 | 0.3255 | No |
| 91 | ACADM | na |  |  | 6509 | 0.502 | 0.3214 | No |
| 92 | ECI1 | na |  |  | 6530 | 0.498 | 0.3238 | No |
| 93 | SURF1 | na |  |  | 6535 | 0.498 | 0.3271 | No |
| 94 | UQCRFS1 | na |  |  | 6825 | 0.465 | 0.3151 | No |
| 95 | ATP5C1 | na |  |  | 6827 | 0.465 | 0.3183 | No |
| 96 | COX7A2 | na |  |  | 6896 | 0.457 | 0.3179 | No |
| 97 | NQO2 | na |  |  | 7499 | 0.385 | 0.2889 | No |
| 98 | IDH3B | na |  |  | 7605 | 0.373 | 0.2860 | No |
| 99 | HCCS | na |  |  | 7731 | 0.359 | 0.2819 | No |
| 100 | VDAC2 | na |  |  | 7830 | 0.346 | 0.2791 | No |
| 101 | NDUFS1 | na |  |  | 7854 | 0.343 | 0.2803 | No |
| 102 | COX4I1 | na |  |  | 7856 | 0.343 | 0.2826 | No |
| 103 | SDHB | na |  |  | 8021 | 0.323 | 0.2762 | No |
| 104 | COX6A1 | na |  |  | 8064 | 0.319 | 0.2762 | No |
| 105 | BAX | na |  |  | 8092 | 0.317 | 0.2770 | No |
| 106 | DLST | na |  |  | 8145 | 0.311 | 0.2765 | No |
| 107 | OGDH | na |  |  | 8191 | 0.306 | 0.2762 | No |
| 108 | ACADSB | na |  |  | 8349 | 0.288 | 0.2700 | No |
| 109 | SUCLG1 | na |  |  | 8421 | 0.282 | 0.2682 | No |
| 110 | ATP6V1C1 | na |  |  | 8435 | 0.280 | 0.2694 | No |
| 111 | NDUFAB1 | na |  |  | 8463 | 0.277 | 0.2700 | No |
| 112 | IDH1 | na |  |  | 8504 | 0.271 | 0.2697 | No |
| 113 | DECR1 | na |  |  | 8591 | 0.263 | 0.2670 | No |
| 114 | SDHC | na |  |  | 8704 | 0.246 | 0.2629 | No |
| 115 | ATP6V0E1 | na |  |  | 8705 | 0.246 | 0.2646 | No |
| 116 | ACAA2 | na |  |  | 8723 | 0.243 | 0.2654 | No |
| 117 | UQCR11 | na |  |  | 8840 | 0.232 | 0.2609 | No |
| 118 | NDUFB6 | na |  |  | 8934 | 0.220 | 0.2575 | No |
| 119 | NDUFA2 | na |  |  | 8935 | 0.220 | 0.2590 | No |
| 120 | NDUFV1 | na |  |  | 9281 | 0.183 | 0.2421 | No |
| 121 | MRPL15 | na |  |  | 9435 | 0.165 | 0.2352 | No |
| 122 | CPT1A | na |  |  | 9521 | 0.154 | 0.2318 | No |
| 123 | RHOT2 | na |  |  | 9543 | 0.152 | 0.2318 | No |
| 124 | OXA1L | na |  |  | 9734 | 0.133 | 0.2227 | No |
| 125 | PDHB | na |  |  | 9808 | 0.125 | 0.2198 | No |
| 126 | HTRA2 | na |  |  | 9952 | 0.108 | 0.2130 | No |
| 127 | MDH2 | na |  |  | 10374 | 0.061 | 0.1912 | No |
| 128 | MTRF1 | na |  |  | 10447 | 0.059 | 0.1879 | No |
| 129 | ATP6V0B | na |  |  | 11130 | -0.018 | 0.1521 | No |
| 130 | ALAS1 | na |  |  | 11139 | -0.018 | 0.1518 | No |
| 131 | ECHS1 | na |  |  | 11160 | -0.022 | 0.1509 | No |
| 132 | NDUFS7 | na |  |  | 11178 | -0.024 | 0.1502 | No |
| 133 | HADHB | na |  |  | 11243 | -0.033 | 0.1470 | No |
| 134 | ATP5J | na |  |  | 11323 | -0.042 | 0.1432 | No |
| 135 | NDUFS4 | na |  |  | 11568 | -0.065 | 0.1308 | No |
| 136 | POR | na |  |  | 11704 | -0.087 | 0.1243 | No |
| 137 | NDUFB8 | na |  |  | 11710 | -0.088 | 0.1246 | No |
| 138 | ATP5D | na |  |  | 12160 | -0.138 | 0.1020 | No |
| 139 | RETSAT | na |  |  | 12351 | -0.162 | 0.0931 | No |
| 140 | ATP5H | na |  |  | 12398 | -0.169 | 0.0919 | No |
| 141 | SLC25A12 | na |  |  | 12501 | -0.182 | 0.0878 | No |
| 142 | UQCRB | na |  |  | 12506 | -0.184 | 0.0888 | No |
| 143 | COX6C | na |  |  | 12625 | -0.202 | 0.0840 | No |
| 144 | CYB5A | na |  |  | 12910 | -0.233 | 0.0707 | No |
| 145 | UQCR10 | na |  |  | 13007 | -0.244 | 0.0673 | No |
| 146 | RHOT1 | na |  |  | 13022 | -0.246 | 0.0683 | No |
| 147 | COX5A | na |  |  | 13092 | -0.256 | 0.0665 | No |
| 148 | NDUFA8 | na |  |  | 13214 | -0.276 | 0.0620 | No |
| 149 | COX7C | na |  |  | 13396 | -0.302 | 0.0546 | No |
| 150 | ATP5J2 | na |  |  | 13397 | -0.302 | 0.0567 | No |
| 151 | PHYH | na |  |  | 13576 | -0.327 | 0.0496 | No |
| 152 | SLC25A6 | na |  |  | 13631 | -0.336 | 0.0491 | No |
| 153 | PDP1 | na |  |  | 13696 | -0.344 | 0.0481 | No |
| 154 | ISCU | na |  |  | 13836 | -0.357 | 0.0433 | No |
| 155 | NDUFS8 | na |  |  | 13842 | -0.359 | 0.0455 | No |
| 156 | ACO2 | na |  |  | 13859 | -0.360 | 0.0472 | No |
| 157 | MRPL34 | na |  |  | 13865 | -0.361 | 0.0494 | No |
| 158 | NDUFS2 | na |  |  | 13895 | -0.365 | 0.0504 | No |
| 159 | ETFDH | na |  |  | 14057 | -0.387 | 0.0446 | No |
| 160 | IDH3G | na |  |  | 14200 | -0.405 | 0.0400 | No |
| 161 | ACADVL | na |  |  | 14586 | -0.460 | 0.0229 | No |
| 162 | NDUFA6 | na |  |  | 14794 | -0.496 | 0.0155 | No |
| 163 | COX6B1 | na |  |  | 15036 | -0.517 | 0.0064 | No |
| 164 | ATP6V1E1 | na |  |  | 15109 | -0.529 | 0.0063 | No |
| 165 | ATP5G2 | na |  |  | 15288 | -0.562 | 0.0008 | No |
| 166 | ATP6V0C | na |  |  | 15346 | -0.573 | 0.0018 | No |
| 167 | CYB5R3 | na |  |  | 15382 | -0.579 | 0.0040 | No |
| 168 | UQCRH | na |  |  | 15421 | -0.585 | 0.0061 | No |
| 169 | ETFB | na |  |  | 15698 | -0.626 | -0.0041 | No |
| 170 | GPI | na |  |  | 15768 | -0.641 | -0.0033 | No |
| 171 | NDUFB7 | na |  |  | 15915 | -0.668 | -0.0063 | No |
| 172 | NDUFB1 | na |  |  | 16018 | -0.689 | -0.0069 | No |
| 173 | ECH1 | na |  |  | 16356 | -0.770 | -0.0193 | No |
| 174 | ATP5E | na |  |  | 16384 | -0.777 | -0.0153 | No |
| 175 | GPX4 | na |  |  | 16614 | -0.837 | -0.0215 | No |
| 176 | ATP6V1G1 | na |  |  | 16646 | -0.845 | -0.0173 | No |
| 177 | NDUFC1 | na |  |  | 16703 | -0.858 | -0.0143 | No |
| 178 | ATP6AP1 | na |  |  | 16807 | -0.889 | -0.0135 | No |
| 179 | COX5B | na |  |  | 17171 | -1.001 | -0.0257 | No |
| 180 | ALDH6A1 | na |  |  | 17199 | -1.008 | -0.0201 | No |
| 181 | NDUFA3 | na |  |  | 17580 | -1.142 | -0.0321 | No |
| 182 | COX7B | na |  |  | 17605 | -1.151 | -0.0254 | No |
| 183 | BDH2 | na |  |  | 17635 | -1.162 | -0.0188 | No |
| 184 | ATP1B1 | na |  |  | 17720 | -1.191 | -0.0150 | No |
| 185 | TCIRG1 | na |  |  | 17773 | -1.214 | -0.0093 | No |
| 186 | MGST3 | na |  |  | 17776 | -1.214 | -0.0009 | No |
| 187 | IDH2 | na |  |  | 17795 | -1.224 | 0.0066 | No |
| 188 | NDUFS6 | na |  |  | 17821 | -1.236 | 0.0139 | No |
| 189 | PDK4 | na |  |  | 17892 | -1.269 | 0.0190 | No |
| 190 | NDUFA1 | na |  |  | 17963 | -1.312 | 0.0245 | No |
| 191 | COX17 | na |  |  | 18071 | -1.366 | 0.0283 | No |
| 192 | MAOB | na |  |  | 18099 | -1.377 | 0.0365 | No |
| 193 | NDUFB4 | na |  |  | 18299 | -1.495 | 0.0364 | No |
| 194 | ATP5I | na |  |  | 18394 | -1.562 | 0.0423 | No |
Table: GSEA details [plain text format]

  

Fig 2: HALLMARK\_OXIDATIVE\_PHOSPHORYLATION      
 Blue-Pink O' Gram in the Space of the Analyzed GeneSet

  

Fig 3: HALLMARK\_OXIDATIVE\_PHOSPHORYLATION: Random ES distribution      
 Gene set null distribution of ES for **HALLMARK\_OXIDATIVE\_PHOSPHORYLATION**

  
