# Supplementary material for: Heparin-based hydrogel scaffolding alters the transcriptomic profile and increases the chemoresistance of MDA-MB-231 triple-negative breast cancer cells
Source: Biomater Sci. 2020 Feb 13;8(10):2786–96. doi: 10.1039/c9bm01481k (PMC7497406; doi:10.1039/c9bm01481k)
Supplement: Supplementary file 2 [file BM-008-C9BM01481K-s002.zip › Supplementary File 4/EGFvControl/Pathways/my_analysis.Gsea.1545200981068/HALLMARK_COMPLEMENT.html]

Details for gene set HALLMARK\_COMPLEMENT[GSEA]

|  || Dataset | expr.class.cls#EGF\_versus\_CONTROL.class.cls#EGF\_versus\_CONTROL\_repos |
| Phenotype | class.cls#EGF\_versus\_CONTROL\_repos |
| Upregulated in class | CONTROL |
| GeneSet | HALLMARK\_COMPLEMENT |
| Enrichment Score (ES) | -0.4187002 |
| Normalized Enrichment Score (NES) | -1.9665027 |
| Nominal p-value | 0.0 |
| FDR q-value | 3.5238094E-4 |
| FWER p-Value | 0.002 |
Table: GSEA Results Summary

  

Fig 1: Enrichment plot: HALLMARK\_COMPLEMENT      
 Profile of the Running ES Score & Positions of GeneSet Members on the Rank Ordered List

  

| PROBE | DESCRIPTION (from dataset) | GENE SYMBOL | GENE\_TITLE | RANK IN GENE LIST | RANK METRIC SCORE | RUNNING ES | CORE ENRICHMENT || 1 | C4BPB | na |  |  | 508 | 1.768 | -0.0115 | No |
| 2 | F3 | na |  |  | 545 | 1.742 | 0.0016 | No |
| 3 | KIF2A | na |  |  | 1070 | 1.489 | -0.0131 | No |
| 4 | CASP3 | na |  |  | 1155 | 1.458 | -0.0050 | No |
| 5 | CTSC | na |  |  | 1305 | 1.410 | -0.0007 | No |
| 6 | ME1 | na |  |  | 1379 | 1.385 | 0.0073 | No |
| 7 | RASGRP1 | na |  |  | 1769 | 1.276 | -0.0021 | No |
| 8 | DGKH | na |  |  | 1871 | 1.251 | 0.0033 | No |
| 9 | SERPINB2 | na |  |  | 2053 | 1.207 | 0.0042 | No |
| 10 | GNAI3 | na |  |  | 2417 | 1.129 | -0.0052 | No |
| 11 | GNB4 | na |  |  | 2614 | 1.092 | -0.0061 | No |
| 12 | KCNIP2 | na |  |  | 2801 | 1.060 | -0.0068 | No |
| 13 | USP14 | na |  |  | 2981 | 1.030 | -0.0073 | No |
| 14 | PFN1 | na |  |  | 3018 | 1.025 | -0.0004 | No |
| 15 | USP15 | na |  |  | 3028 | 1.022 | 0.0079 | No |
| 16 | RNF4 | na |  |  | 3328 | 0.966 | 0.0005 | No |
| 17 | USP16 | na |  |  | 3415 | 0.951 | 0.0041 | No |
| 18 | PPP2CB | na |  |  | 3484 | 0.939 | 0.0086 | No |
| 19 | FDX1 | na |  |  | 3610 | 0.918 | 0.0100 | No |
| 20 | RAF1 | na |  |  | 3823 | 0.880 | 0.0064 | No |
| 21 | PRCP | na |  |  | 4361 | 0.799 | -0.0149 | No |
| 22 | GPD2 | na |  |  | 4376 | 0.795 | -0.0088 | No |
| 23 | LAP3 | na |  |  | 4650 | 0.756 | -0.0167 | No |
| 24 | GRB2 | na |  |  | 4705 | 0.748 | -0.0131 | No |
| 25 | GMFB | na |  |  | 4986 | 0.710 | -0.0217 | No |
| 26 | CASP7 | na |  |  | 5606 | 0.625 | -0.0488 | No |
| 27 | CDK5R1 | na |  |  | 5732 | 0.605 | -0.0502 | No |
| 28 | ADAM9 | na |  |  | 5789 | 0.598 | -0.0480 | No |
| 29 | LIPA | na |  |  | 5938 | 0.579 | -0.0508 | No |
| 30 | CALM3 | na |  |  | 6126 | 0.551 | -0.0558 | No |
| 31 | DYRK2 | na |  |  | 6172 | 0.546 | -0.0535 | No |
| 32 | AKAP10 | na |  |  | 6293 | 0.528 | -0.0553 | No |
| 33 | JAK2 | na |  |  | 6438 | 0.510 | -0.0585 | No |
| 34 | GCA | na |  |  | 6687 | 0.481 | -0.0674 | No |
| 35 | KCNIP3 | na |  |  | 6903 | 0.456 | -0.0747 | No |
| 36 | EHD1 | na |  |  | 6990 | 0.448 | -0.0754 | No |
| 37 | ZFPM2 | na |  |  | 7136 | 0.429 | -0.0793 | No |
| 38 | USP8 | na |  |  | 7339 | 0.403 | -0.0865 | No |
| 39 | CDA | na |  |  | 7578 | 0.376 | -0.0957 | No |
| 40 | LYN | na |  |  | 7669 | 0.366 | -0.0973 | No |
| 41 | ERAP2 | na |  |  | 7797 | 0.349 | -0.1010 | No |
| 42 | CALM1 | na |  |  | 7845 | 0.344 | -0.1005 | No |
| 43 | PCLO | na |  |  | 7974 | 0.329 | -0.1044 | No |
| 44 | IRF2 | na |  |  | 8035 | 0.322 | -0.1048 | No |
| 45 | GATA3 | na |  |  | 8290 | 0.294 | -0.1156 | No |
| 46 | PLSCR1 | na |  |  | 8332 | 0.290 | -0.1152 | No |
| 47 | BRPF3 | na |  |  | 8511 | 0.271 | -0.1223 | No |
| 48 | RHOG | na |  |  | 9030 | 0.212 | -0.1476 | No |
| 49 | CTSH | na |  |  | 9066 | 0.206 | -0.1477 | No |
| 50 | F8 | na |  |  | 9146 | 0.197 | -0.1501 | No |
| 51 | PRDM4 | na |  |  | 9440 | 0.165 | -0.1641 | No |
| 52 | PREP | na |  |  | 10004 | 0.106 | -0.1927 | No |
| 53 | VCPIP1 | na |  |  | 10036 | 0.101 | -0.1935 | No |
| 54 | DOCK9 | na |  |  | 10213 | 0.079 | -0.2020 | No |
| 55 | LGMN | na |  |  | 10233 | 0.077 | -0.2024 | No |
| 56 | CASP1 | na |  |  | 10391 | 0.060 | -0.2101 | No |
| 57 | PIK3CG | na |  |  | 10407 | 0.060 | -0.2104 | No |
| 58 | PIK3CA | na |  |  | 10602 | 0.037 | -0.2202 | No |
| 59 | RCE1 | na |  |  | 10653 | 0.032 | -0.2226 | No |
| 60 | CASP4 | na |  |  | 10964 | 0.000 | -0.2389 | No |
| 61 | LAMP2 | na |  |  | 11032 | -0.007 | -0.2423 | No |
| 62 | RABIF | na |  |  | 11147 | -0.020 | -0.2481 | No |
| 63 | CFH | na |  |  | 11540 | -0.062 | -0.2682 | No |
| 64 | CSRP1 | na |  |  | 11580 | -0.067 | -0.2696 | No |
| 65 | COL4A2 | na |  |  | 11618 | -0.072 | -0.2710 | No |
| 66 | GNB2 | na |  |  | 11716 | -0.089 | -0.2753 | No |
| 67 | XPNPEP1 | na |  |  | 11807 | -0.100 | -0.2791 | No |
| 68 | SH2B3 | na |  |  | 11965 | -0.121 | -0.2863 | No |
| 69 | PLA2G4A | na |  |  | 12476 | -0.181 | -0.3116 | No |
| 70 | PRKCD | na |  |  | 12636 | -0.204 | -0.3181 | No |
| 71 | ZEB1 | na |  |  | 12970 | -0.242 | -0.3335 | No |
| 72 | ANXA5 | na |  |  | 13034 | -0.249 | -0.3347 | No |
| 73 | PRSS36 | na |  |  | 13300 | -0.287 | -0.3462 | No |
| 74 | TIMP2 | na |  |  | 13317 | -0.291 | -0.3445 | No |
| 75 | TMPRSS6 | na |  |  | 13328 | -0.292 | -0.3425 | No |
| 76 | PLG | na |  |  | 13548 | -0.322 | -0.3512 | No |
| 77 | LTA4H | na |  |  | 13613 | -0.333 | -0.3517 | No |
| 78 | PDP1 | na |  |  | 13696 | -0.344 | -0.3531 | No |
| 79 | CASP9 | na |  |  | 13830 | -0.355 | -0.3570 | No |
| 80 | MMP15 | na |  |  | 13999 | -0.378 | -0.3626 | No |
| 81 | PDGFB | na |  |  | 14224 | -0.409 | -0.3708 | No |
| 82 | DOCK10 | na |  |  | 14457 | -0.438 | -0.3793 | No |
| 83 | DPP4 | na |  |  | 14594 | -0.462 | -0.3824 | No |
| 84 | IRF7 | na |  |  | 14815 | -0.500 | -0.3897 | No |
| 85 | PSEN1 | na |  |  | 15031 | -0.515 | -0.3965 | No |
| 86 | CP | na |  |  | 15210 | -0.548 | -0.4012 | No |
| 87 | ANG | na |  |  | 15384 | -0.579 | -0.4053 | No |
| 88 | FYN | na |  |  | 15435 | -0.588 | -0.4029 | No |
| 89 | ATOX1 | na |  |  | 15487 | -0.595 | -0.4004 | No |
| 90 | SIRT6 | na |  |  | 15594 | -0.604 | -0.4008 | No |
| 91 | CPM | na |  |  | 15618 | -0.609 | -0.3968 | No |
| 92 | PLAUR | na |  |  | 15641 | -0.614 | -0.3927 | No |
| 93 | CEBPB | na |  |  | 15897 | -0.665 | -0.4003 | No |
| 94 | HSPA5 | na |  |  | 16140 | -0.715 | -0.4069 | No |
| 95 | APOC1 | na |  |  | 16366 | -0.773 | -0.4121 | Yes |
| 96 | DOCK4 | na |  |  | 16444 | -0.795 | -0.4093 | Yes |
| 97 | NOTCH4 | na |  |  | 16533 | -0.819 | -0.4069 | Yes |
| 98 | HSPA1A | na |  |  | 16690 | -0.854 | -0.4077 | Yes |
| 99 | MAFF | na |  |  | 16697 | -0.856 | -0.4007 | Yes |
| 100 | PPP4C | na |  |  | 16710 | -0.860 | -0.3939 | Yes |
| 101 | GP1BA | na |  |  | 16758 | -0.872 | -0.3889 | Yes |
| 102 | GNAI2 | na |  |  | 16842 | -0.901 | -0.3855 | Yes |
| 103 | CTSD | na |  |  | 16910 | -0.924 | -0.3811 | Yes |
| 104 | DUSP5 | na |  |  | 16942 | -0.934 | -0.3747 | Yes |
| 105 | SRC | na |  |  | 16943 | -0.934 | -0.3667 | Yes |
| 106 | PRSS3 | na |  |  | 17185 | -1.005 | -0.3707 | Yes |
| 107 | PIM1 | na |  |  | 17218 | -1.013 | -0.3637 | Yes |
| 108 | CXCL1 | na |  |  | 17379 | -1.069 | -0.3629 | Yes |
| 109 | C1R | na |  |  | 17490 | -1.119 | -0.3591 | Yes |
| 110 | TNFAIP3 | na |  |  | 17494 | -1.121 | -0.3496 | Yes |
| 111 | S100A13 | na |  |  | 17565 | -1.138 | -0.3435 | Yes |
| 112 | TIMP1 | na |  |  | 17600 | -1.148 | -0.3355 | Yes |
| 113 | GNG2 | na |  |  | 17610 | -1.152 | -0.3260 | Yes |
| 114 | C3 | na |  |  | 17617 | -1.156 | -0.3164 | Yes |
| 115 | CFB | na |  |  | 17623 | -1.158 | -0.3068 | Yes |
| 116 | CD46 | na |  |  | 17656 | -1.169 | -0.2984 | Yes |
| 117 | CD55 | na |  |  | 17692 | -1.182 | -0.2901 | Yes |
| 118 | PHEX | na |  |  | 17844 | -1.252 | -0.2873 | Yes |
| 119 | TFPI2 | na |  |  | 17846 | -1.252 | -0.2766 | Yes |
| 120 | CBLB | na |  |  | 17974 | -1.316 | -0.2719 | Yes |
| 121 | CD59 | na |  |  | 17994 | -1.325 | -0.2616 | Yes |
| 122 | STX4 | na |  |  | 18000 | -1.329 | -0.2504 | Yes |
| 123 | IRF1 | na |  |  | 18023 | -1.342 | -0.2400 | Yes |
| 124 | PSMB9 | na |  |  | 18166 | -1.407 | -0.2354 | Yes |
| 125 | PCSK9 | na |  |  | 18266 | -1.470 | -0.2280 | Yes |
| 126 | LRP1 | na |  |  | 18393 | -1.561 | -0.2212 | Yes |
| 127 | SERPINA1 | na |  |  | 18420 | -1.593 | -0.2089 | Yes |
| 128 | CLU | na |  |  | 18498 | -1.641 | -0.1988 | Yes |
| 129 | DUSP6 | na |  |  | 18544 | -1.690 | -0.1867 | Yes |
| 130 | APOBEC3G | na |  |  | 18546 | -1.692 | -0.1722 | Yes |
| 131 | CASP10 | na |  |  | 18607 | -1.760 | -0.1603 | Yes |
| 132 | PLAT | na |  |  | 18660 | -1.832 | -0.1473 | Yes |
| 133 | LGALS3 | na |  |  | 18663 | -1.837 | -0.1316 | Yes |
| 134 | CTSB | na |  |  | 18701 | -1.896 | -0.1173 | Yes |
| 135 | CTSO | na |  |  | 18747 | -1.958 | -0.1028 | Yes |
| 136 | IL6 | na |  |  | 18776 | -2.020 | -0.0870 | Yes |
| 137 | APOBEC3F | na |  |  | 18824 | -2.116 | -0.0713 | Yes |
| 138 | C1S | na |  |  | 18893 | -2.301 | -0.0551 | Yes |
| 139 | C2 | na |  |  | 18918 | -2.345 | -0.0362 | Yes |
| 140 | CTSS | na |  |  | 18977 | -2.550 | -0.0174 | Yes |
| 141 | KYNU | na |  |  | 19126 | -3.367 | 0.0038 | Yes |
Table: GSEA details [plain text format]

  

Fig 2: HALLMARK\_COMPLEMENT      
 Blue-Pink O' Gram in the Space of the Analyzed GeneSet

  

Fig 3: HALLMARK\_COMPLEMENT: Random ES distribution      
 Gene set null distribution of ES for **HALLMARK\_COMPLEMENT**

  
