# Supplementary material for: Heparin-based hydrogel scaffolding alters the transcriptomic profile and increases the chemoresistance of MDA-MB-231 triple-negative breast cancer cells
Source: Biomater Sci. 2020 Feb 13;8(10):2786–96. doi: 10.1039/c9bm01481k (PMC7497406; doi:10.1039/c9bm01481k)
Supplement: Supplementary file 2 [file BM-008-C9BM01481K-s002.zip › Supplementary File 4/EGFvControl/Pathways/my_analysis.Gsea.1545200981068/HALLMARK_TGF_BETA_SIGNALING.html]

Details for gene set HALLMARK\_TGF\_BETA\_SIGNALING[GSEA]

|  || Dataset | expr.class.cls#EGF\_versus\_CONTROL.class.cls#EGF\_versus\_CONTROL\_repos |
| Phenotype | class.cls#EGF\_versus\_CONTROL\_repos |
| Upregulated in class | CONTROL |
| GeneSet | HALLMARK\_TGF\_BETA\_SIGNALING |
| Enrichment Score (ES) | -0.32090157 |
| Normalized Enrichment Score (NES) | -1.2382749 |
| Nominal p-value | 0.14251782 |
| FDR q-value | 0.12317152 |
| FWER p-Value | 0.865 |
Table: GSEA Results Summary

  

Fig 1: Enrichment plot: HALLMARK\_TGF\_BETA\_SIGNALING      
 Profile of the Running ES Score & Positions of GeneSet Members on the Rank Ordered List

  

| PROBE | DESCRIPTION (from dataset) | GENE SYMBOL | GENE\_TITLE | RANK IN GENE LIST | RANK METRIC SCORE | RUNNING ES | CORE ENRICHMENT || 1 | SMAD7 | na |  |  | 195 | 2.108 | 0.0444 | No |
| 2 | ID3 | na |  |  | 417 | 1.833 | 0.0803 | No |
| 3 | NOG | na |  |  | 428 | 1.822 | 0.1270 | No |
| 4 | SMURF2 | na |  |  | 2130 | 1.192 | 0.0691 | No |
| 5 | PMEPA1 | na |  |  | 2174 | 1.184 | 0.0975 | No |
| 6 | HIPK2 | na |  |  | 2290 | 1.159 | 0.1215 | No |
| 7 | SMURF1 | na |  |  | 4075 | 0.840 | 0.0501 | No |
| 8 | FKBP1A | na |  |  | 4357 | 0.799 | 0.0561 | No |
| 9 | TJP1 | na |  |  | 4454 | 0.784 | 0.0714 | No |
| 10 | APC | na |  |  | 4827 | 0.725 | 0.0707 | No |
| 11 | SKIL | na |  |  | 5109 | 0.691 | 0.0740 | No |
| 12 | RHOA | na |  |  | 5369 | 0.655 | 0.0774 | No |
| 13 | BMPR1A | na |  |  | 5722 | 0.606 | 0.0747 | No |
| 14 | FURIN | na |  |  | 6470 | 0.506 | 0.0488 | No |
| 15 | FNTA | na |  |  | 7734 | 0.358 | -0.0079 | No |
| 16 | MAP3K7 | na |  |  | 7759 | 0.355 | 0.0001 | No |
| 17 | UBE2D3 | na |  |  | 8015 | 0.323 | -0.0049 | No |
| 18 | CDH1 | na |  |  | 8288 | 0.294 | -0.0115 | No |
| 19 | ACVR1 | na |  |  | 8375 | 0.286 | -0.0085 | No |
| 20 | PPM1A | na |  |  | 8495 | 0.272 | -0.0077 | No |
| 21 | PPP1R15A | na |  |  | 8701 | 0.246 | -0.0120 | No |
| 22 | SLC20A1 | na |  |  | 9338 | 0.179 | -0.0406 | No |
| 23 | HDAC1 | na |  |  | 9563 | 0.149 | -0.0484 | No |
| 24 | TRIM33 | na |  |  | 9723 | 0.134 | -0.0533 | No |
| 25 | CDK9 | na |  |  | 9741 | 0.132 | -0.0507 | No |
| 26 | BMPR2 | na |  |  | 9896 | 0.116 | -0.0558 | No |
| 27 | ENG | na |  |  | 10136 | 0.091 | -0.0659 | No |
| 28 | SKI | na |  |  | 10172 | 0.085 | -0.0655 | No |
| 29 | SMAD1 | na |  |  | 10541 | 0.047 | -0.0835 | No |
| 30 | PPP1CA | na |  |  | 11047 | -0.009 | -0.1096 | No |
| 31 | TGFBR1 | na |  |  | 11331 | -0.043 | -0.1233 | No |
| 32 | ARID4B | na |  |  | 11421 | -0.051 | -0.1266 | No |
| 33 | ID2 | na |  |  | 11940 | -0.118 | -0.1506 | No |
| 34 | CTNNB1 | na |  |  | 13218 | -0.277 | -0.2102 | No |
| 35 | SMAD6 | na |  |  | 14209 | -0.406 | -0.2513 | No |
| 36 | NCOR2 | na |  |  | 14701 | -0.480 | -0.2646 | No |
| 37 | XIAP | na |  |  | 15171 | -0.541 | -0.2750 | No |
| 38 | SMAD3 | na |  |  | 15810 | -0.650 | -0.2915 | No |
| 39 | BCAR3 | na |  |  | 15894 | -0.665 | -0.2786 | No |
| 40 | KLF10 | na |  |  | 16235 | -0.741 | -0.2772 | No |
| 41 | ID1 | na |  |  | 16701 | -0.857 | -0.2793 | No |
| 42 | JUNB | na |  |  | 17499 | -1.123 | -0.2918 | Yes |
| 43 | CDKN1C | na |  |  | 17571 | -1.139 | -0.2660 | Yes |
| 44 | TGFB1 | na |  |  | 17599 | -1.148 | -0.2377 | Yes |
| 45 | WWTR1 | na |  |  | 18194 | -1.422 | -0.2319 | Yes |
| 46 | TGIF1 | na |  |  | 18371 | -1.541 | -0.2012 | Yes |
| 47 | IFNGR2 | na |  |  | 18421 | -1.594 | -0.1625 | Yes |
| 48 | LTBP2 | na |  |  | 18800 | -2.073 | -0.1285 | Yes |
| 49 | RAB31 | na |  |  | 18945 | -2.421 | -0.0733 | Yes |
| 50 | BMP2 | na |  |  | 19119 | -3.339 | 0.0041 | Yes |
Table: GSEA details [plain text format]

  

Fig 2: HALLMARK\_TGF\_BETA\_SIGNALING      
 Blue-Pink O' Gram in the Space of the Analyzed GeneSet

  

Fig 3: HALLMARK\_TGF\_BETA\_SIGNALING: Random ES distribution      
 Gene set null distribution of ES for **HALLMARK\_TGF\_BETA\_SIGNALING**

  
