# Supplementary material for: Heparin-based hydrogel scaffolding alters the transcriptomic profile and increases the chemoresistance of MDA-MB-231 triple-negative breast cancer cells
Source: Biomater Sci. 2020 Feb 13;8(10):2786–96. doi: 10.1039/c9bm01481k (PMC7497406; doi:10.1039/c9bm01481k)
Supplement: Supplementary file 2 [file BM-008-C9BM01481K-s002.zip › Supplementary File 4/EGFvControl/Pathways/my_analysis.Gsea.1545200981068/HALLMARK_MYC_TARGETS_V2.html]

Details for gene set HALLMARK\_MYC\_TARGETS\_V2[GSEA]

|  || Dataset | expr.class.cls#EGF\_versus\_CONTROL.class.cls#EGF\_versus\_CONTROL\_repos |
| Phenotype | class.cls#EGF\_versus\_CONTROL\_repos |
| Upregulated in class | EGF |
| GeneSet | HALLMARK\_MYC\_TARGETS\_V2 |
| Enrichment Score (ES) | 0.72822225 |
| Normalized Enrichment Score (NES) | 2.7766533 |
| Nominal p-value | 0.0 |
| FDR q-value | 0.0 |
| FWER p-Value | 0.0 |
Table: GSEA Results Summary

  

Fig 1: Enrichment plot: HALLMARK\_MYC\_TARGETS\_V2      
 Profile of the Running ES Score & Positions of GeneSet Members on the Rank Ordered List

  

| PROBE | DESCRIPTION (from dataset) | GENE SYMBOL | GENE\_TITLE | RANK IN GENE LIST | RANK METRIC SCORE | RUNNING ES | CORE ENRICHMENT || 1 | IPO4 | na |  |  | 18 | 3.016 | 0.0409 | Yes |
| 2 | NOP16 | na |  |  | 164 | 2.177 | 0.0635 | Yes |
| 3 | NOP2 | na |  |  | 166 | 2.175 | 0.0936 | Yes |
| 4 | PRMT3 | na |  |  | 305 | 1.946 | 0.1133 | Yes |
| 5 | MYBBP1A | na |  |  | 337 | 1.904 | 0.1381 | Yes |
| 6 | UNG | na |  |  | 348 | 1.892 | 0.1638 | Yes |
| 7 | NOLC1 | na |  |  | 443 | 1.809 | 0.1839 | Yes |
| 8 | TFB2M | na |  |  | 478 | 1.782 | 0.2069 | Yes |
| 9 | RRP9 | na |  |  | 522 | 1.757 | 0.2290 | Yes |
| 10 | GRWD1 | na |  |  | 559 | 1.733 | 0.2511 | Yes |
| 11 | UTP20 | na |  |  | 612 | 1.696 | 0.2719 | Yes |
| 12 | HSPD1 | na |  |  | 619 | 1.691 | 0.2950 | Yes |
| 13 | TCOF1 | na |  |  | 629 | 1.686 | 0.3179 | Yes |
| 14 | WDR43 | na |  |  | 648 | 1.672 | 0.3402 | Yes |
| 15 | PLK4 | na |  |  | 650 | 1.670 | 0.3633 | Yes |
| 16 | PPRC1 | na |  |  | 713 | 1.635 | 0.3827 | Yes |
| 17 | RRP12 | na |  |  | 758 | 1.606 | 0.4026 | Yes |
| 18 | TMEM97 | na |  |  | 776 | 1.599 | 0.4239 | Yes |
| 19 | AIMP2 | na |  |  | 810 | 1.586 | 0.4442 | Yes |
| 20 | RCL1 | na |  |  | 820 | 1.581 | 0.4656 | Yes |
| 21 | MRTO4 | na |  |  | 822 | 1.579 | 0.4874 | Yes |
| 22 | PA2G4 | na |  |  | 838 | 1.574 | 0.5085 | Yes |
| 23 | SRM | na |  |  | 940 | 1.535 | 0.5245 | Yes |
| 24 | NDUFAF4 | na |  |  | 957 | 1.526 | 0.5448 | Yes |
| 25 | PLK1 | na |  |  | 1074 | 1.488 | 0.5593 | Yes |
| 26 | PHB | na |  |  | 1075 | 1.488 | 0.5800 | Yes |
| 27 | DCTPP1 | na |  |  | 1111 | 1.474 | 0.5986 | Yes |
| 28 | NOP56 | na |  |  | 1153 | 1.459 | 0.6166 | Yes |
| 29 | NIP7 | na |  |  | 1338 | 1.396 | 0.6264 | Yes |
| 30 | MCM4 | na |  |  | 1617 | 1.313 | 0.6300 | Yes |
| 31 | IMP4 | na |  |  | 1791 | 1.271 | 0.6386 | Yes |
| 32 | RABEPK | na |  |  | 1794 | 1.270 | 0.6561 | Yes |
| 33 | GNL3 | na |  |  | 1932 | 1.239 | 0.6661 | Yes |
| 34 | NOC4L | na |  |  | 1961 | 1.233 | 0.6818 | Yes |
| 35 | BYSL | na |  |  | 2144 | 1.189 | 0.6887 | Yes |
| 36 | DDX18 | na |  |  | 2362 | 1.142 | 0.6932 | Yes |
| 37 | TBRG4 | na |  |  | 2442 | 1.125 | 0.7047 | Yes |
| 38 | PUS1 | na |  |  | 2524 | 1.107 | 0.7158 | Yes |
| 39 | SORD | na |  |  | 2578 | 1.097 | 0.7282 | Yes |
| 40 | MCM5 | na |  |  | 3202 | 0.986 | 0.7093 | No |
| 41 | MYC | na |  |  | 3907 | 0.866 | 0.6846 | No |
| 42 | CBX3 | na |  |  | 4321 | 0.805 | 0.6742 | No |
| 43 | PES1 | na |  |  | 4588 | 0.765 | 0.6709 | No |
| 44 | SUPV3L1 | na |  |  | 4635 | 0.758 | 0.6790 | No |
| 45 | LAS1L | na |  |  | 4710 | 0.746 | 0.6854 | No |
| 46 | MPHOSPH10 | na |  |  | 4785 | 0.733 | 0.6917 | No |
| 47 | CDK4 | na |  |  | 4801 | 0.730 | 0.7011 | No |
| 48 | EXOSC5 | na |  |  | 5294 | 0.666 | 0.6846 | No |
| 49 | HSPE1 | na |  |  | 5965 | 0.574 | 0.6575 | No |
| 50 | FARSA | na |  |  | 8226 | 0.301 | 0.5437 | No |
| 51 | WDR74 | na |  |  | 10585 | 0.040 | 0.4210 | No |
| 52 | SLC19A1 | na |  |  | 11074 | -0.013 | 0.3957 | No |
| 53 | MAP3K6 | na |  |  | 13096 | -0.256 | 0.2937 | No |
| 54 | PPAN | na |  |  | 13394 | -0.302 | 0.2824 | No |
| 55 | HK2 | na |  |  | 14409 | -0.434 | 0.2354 | No |
| 56 | SLC29A2 | na |  |  | 17352 | -1.060 | 0.0964 | No |
Table: GSEA details [plain text format]

  

Fig 2: HALLMARK\_MYC\_TARGETS\_V2      
 Blue-Pink O' Gram in the Space of the Analyzed GeneSet

  

Fig 3: HALLMARK\_MYC\_TARGETS\_V2: Random ES distribution      
 Gene set null distribution of ES for **HALLMARK\_MYC\_TARGETS\_V2**

  
