# Supplementary material for: Heparin-based hydrogel scaffolding alters the transcriptomic profile and increases the chemoresistance of MDA-MB-231 triple-negative breast cancer cells
Source: Biomater Sci. 2020 Feb 13;8(10):2786–96. doi: 10.1039/c9bm01481k (PMC7497406; doi:10.1039/c9bm01481k)
Supplement: Supplementary file 2 [file BM-008-C9BM01481K-s002.zip › Supplementary File 4/EGFvControl/Pathways/my_analysis.Gsea.1545200981068/HALLMARK_ANGIOGENESIS.html]

Details for gene set HALLMARK\_ANGIOGENESIS[GSEA]

|  || Dataset | expr.class.cls#EGF\_versus\_CONTROL.class.cls#EGF\_versus\_CONTROL\_repos |
| Phenotype | class.cls#EGF\_versus\_CONTROL\_repos |
| Upregulated in class | CONTROL |
| GeneSet | HALLMARK\_ANGIOGENESIS |
| Enrichment Score (ES) | -0.5335993 |
| Normalized Enrichment Score (NES) | -1.6978166 |
| Nominal p-value | 0.015801355 |
| FDR q-value | 0.004756662 |
| FWER p-Value | 0.045 |
Table: GSEA Results Summary

  

Fig 1: Enrichment plot: HALLMARK\_ANGIOGENESIS      
 Profile of the Running ES Score & Positions of GeneSet Members on the Rank Ordered List

  

| PROBE | DESCRIPTION (from dataset) | GENE SYMBOL | GENE\_TITLE | RANK IN GENE LIST | RANK METRIC SCORE | RUNNING ES | CORE ENRICHMENT || 1 | COL5A2 | na |  |  | 273 | 1.994 | 0.0678 | No |
| 2 | FSTL1 | na |  |  | 1011 | 1.511 | 0.0914 | No |
| 3 | PDGFA | na |  |  | 2978 | 1.030 | 0.0312 | No |
| 4 | PTK2 | na |  |  | 4636 | 0.758 | -0.0240 | No |
| 5 | PGLYRP1 | na |  |  | 5532 | 0.634 | -0.0446 | No |
| 6 | VAV2 | na |  |  | 7580 | 0.375 | -0.1359 | No |
| 7 | VCAN | na |  |  | 8574 | 0.265 | -0.1768 | No |
| 8 | FGFR1 | na |  |  | 8893 | 0.225 | -0.1841 | No |
| 9 | LPL | na |  |  | 9088 | 0.203 | -0.1859 | No |
| 10 | LRPAP1 | na |  |  | 11161 | -0.022 | -0.2931 | No |
| 11 | JAG2 | na |  |  | 15291 | -0.563 | -0.4853 | No |
| 12 | JAG1 | na |  |  | 15596 | -0.604 | -0.4763 | No |
| 13 | THBD | na |  |  | 16696 | -0.856 | -0.4984 | Yes |
| 14 | TNFRSF21 | na |  |  | 17238 | -1.017 | -0.4848 | Yes |
| 15 | CCND2 | na |  |  | 17385 | -1.070 | -0.4485 | Yes |
| 16 | VTN | na |  |  | 17582 | -1.142 | -0.4117 | Yes |
| 17 | TIMP1 | na |  |  | 17600 | -1.148 | -0.3654 | Yes |
| 18 | ITGAV | na |  |  | 17601 | -1.149 | -0.3182 | Yes |
| 19 | SERPINA5 | na |  |  | 17678 | -1.177 | -0.2738 | Yes |
| 20 | NRP1 | na |  |  | 18351 | -1.527 | -0.2460 | Yes |
| 21 | APP | na |  |  | 18352 | -1.528 | -0.1832 | Yes |
| 22 | STC1 | na |  |  | 18997 | -2.647 | -0.1080 | Yes |
| 23 | S100A4 | na |  |  | 19044 | -2.881 | 0.0080 | Yes |
Table: GSEA details [plain text format]

  

Fig 2: HALLMARK\_ANGIOGENESIS      
 Blue-Pink O' Gram in the Space of the Analyzed GeneSet

  

Fig 3: HALLMARK\_ANGIOGENESIS: Random ES distribution      
 Gene set null distribution of ES for **HALLMARK\_ANGIOGENESIS**

  
