# Supplementary material for: Heparin-based hydrogel scaffolding alters the transcriptomic profile and increases the chemoresistance of MDA-MB-231 triple-negative breast cancer cells
Source: Biomater Sci. 2020 Feb 13;8(10):2786–96. doi: 10.1039/c9bm01481k (PMC7497406; doi:10.1039/c9bm01481k)
Supplement: Supplementary file 2 [file BM-008-C9BM01481K-s002.zip › Supplementary File 4/EGFvControl/Pathways/my_analysis.Gsea.1545200981068/HALLMARK_UV_RESPONSE_DN.html]

Details for gene set HALLMARK\_UV\_RESPONSE\_DN[GSEA]

|  || Dataset | expr.class.cls#EGF\_versus\_CONTROL.class.cls#EGF\_versus\_CONTROL\_repos |
| Phenotype | class.cls#EGF\_versus\_CONTROL\_repos |
| Upregulated in class | EGF |
| GeneSet | HALLMARK\_UV\_RESPONSE\_DN |
| Enrichment Score (ES) | 0.22070856 |
| Normalized Enrichment Score (NES) | 0.9669643 |
| Nominal p-value | 0.5344 |
| FDR q-value | 0.63309866 |
| FWER p-Value | 1.0 |
Table: GSEA Results Summary

  

Fig 1: Enrichment plot: HALLMARK\_UV\_RESPONSE\_DN      
 Profile of the Running ES Score & Positions of GeneSet Members on the Rank Ordered List

  

| PROBE | DESCRIPTION (from dataset) | GENE SYMBOL | GENE\_TITLE | RANK IN GENE LIST | RANK METRIC SCORE | RUNNING ES | CORE ENRICHMENT || 1 | BDNF | na |  |  | 126 | 2.261 | 0.0127 | Yes |
| 2 | RGS4 | na |  |  | 147 | 2.214 | 0.0306 | Yes |
| 3 | SMAD7 | na |  |  | 195 | 2.108 | 0.0461 | Yes |
| 4 | COL5A2 | na |  |  | 273 | 1.994 | 0.0591 | Yes |
| 5 | SLC7A1 | na |  |  | 311 | 1.936 | 0.0737 | Yes |
| 6 | RND3 | na |  |  | 431 | 1.819 | 0.0830 | Yes |
| 7 | ADORA2B | na |  |  | 492 | 1.775 | 0.0950 | Yes |
| 8 | DLC1 | na |  |  | 539 | 1.747 | 0.1076 | Yes |
| 9 | F3 | na |  |  | 545 | 1.742 | 0.1222 | Yes |
| 10 | RBPMS | na |  |  | 577 | 1.720 | 0.1353 | Yes |
| 11 | MT1E | na |  |  | 578 | 1.719 | 0.1499 | Yes |
| 12 | SNAI2 | na |  |  | 716 | 1.632 | 0.1567 | Yes |
| 13 | EFEMP1 | na |  |  | 850 | 1.569 | 0.1631 | Yes |
| 14 | PRKCE | na |  |  | 1041 | 1.497 | 0.1660 | Yes |
| 15 | DDAH1 | na |  |  | 1103 | 1.478 | 0.1754 | Yes |
| 16 | SYNE1 | na |  |  | 1466 | 1.357 | 0.1680 | Yes |
| 17 | CAV1 | na |  |  | 1542 | 1.335 | 0.1755 | Yes |
| 18 | NEK7 | na |  |  | 2286 | 1.159 | 0.1464 | Yes |
| 19 | ATP2B1 | na |  |  | 2361 | 1.142 | 0.1523 | Yes |
| 20 | ARHGEF9 | na |  |  | 2625 | 1.091 | 0.1478 | Yes |
| 21 | INPP4B | na |  |  | 2707 | 1.075 | 0.1528 | Yes |
| 22 | PTPN21 | na |  |  | 3149 | 0.997 | 0.1382 | Yes |
| 23 | MAPK14 | na |  |  | 3187 | 0.990 | 0.1447 | Yes |
| 24 | NR1D2 | na |  |  | 3282 | 0.972 | 0.1481 | Yes |
| 25 | NFIB | na |  |  | 3284 | 0.972 | 0.1563 | Yes |
| 26 | GCNT1 | na |  |  | 3320 | 0.967 | 0.1627 | Yes |
| 27 | FHL2 | na |  |  | 3426 | 0.948 | 0.1653 | Yes |
| 28 | MET | na |  |  | 3522 | 0.933 | 0.1683 | Yes |
| 29 | DLG1 | na |  |  | 3579 | 0.924 | 0.1733 | Yes |
| 30 | AKT3 | na |  |  | 3603 | 0.919 | 0.1799 | Yes |
| 31 | MYC | na |  |  | 3907 | 0.866 | 0.1714 | Yes |
| 32 | PPARG | na |  |  | 3915 | 0.866 | 0.1785 | Yes |
| 33 | SFMBT1 | na |  |  | 3983 | 0.853 | 0.1823 | Yes |
| 34 | LPAR1 | na |  |  | 4142 | 0.830 | 0.1811 | Yes |
| 35 | MIOS | na |  |  | 4214 | 0.820 | 0.1843 | Yes |
| 36 | SRI | na |  |  | 4299 | 0.808 | 0.1868 | Yes |
| 37 | PRKCA | na |  |  | 4313 | 0.806 | 0.1930 | Yes |
| 38 | TJP1 | na |  |  | 4454 | 0.784 | 0.1924 | Yes |
| 39 | RUNX1 | na |  |  | 4506 | 0.777 | 0.1964 | Yes |
| 40 | PAPD7 | na |  |  | 4532 | 0.773 | 0.2017 | Yes |
| 41 | YTHDC1 | na |  |  | 4581 | 0.766 | 0.2057 | Yes |
| 42 | PRKAR2B | na |  |  | 4603 | 0.763 | 0.2111 | Yes |
| 43 | CAP2 | na |  |  | 4663 | 0.755 | 0.2145 | Yes |
| 44 | ATP5S | na |  |  | 4701 | 0.748 | 0.2189 | Yes |
| 45 | FZD2 | na |  |  | 4787 | 0.733 | 0.2207 | Yes |
| 46 | NR3C1 | na |  |  | 5093 | 0.695 | 0.2106 | No |
| 47 | MAGI2 | na |  |  | 5237 | 0.674 | 0.2089 | No |
| 48 | SPOP | na |  |  | 5307 | 0.664 | 0.2110 | No |
| 49 | WDR37 | na |  |  | 5350 | 0.658 | 0.2144 | No |
| 50 | MAP1B | na |  |  | 5465 | 0.642 | 0.2139 | No |
| 51 | DYRK1A | na |  |  | 5581 | 0.628 | 0.2132 | No |
| 52 | NIPBL | na |  |  | 5678 | 0.613 | 0.2134 | No |
| 53 | BMPR1A | na |  |  | 5722 | 0.606 | 0.2163 | No |
| 54 | NOTCH2 | na |  |  | 6144 | 0.549 | 0.1990 | No |
| 55 | MGLL | na |  |  | 6147 | 0.549 | 0.2035 | No |
| 56 | ATP2C1 | na |  |  | 6254 | 0.533 | 0.2025 | No |
| 57 | HAS2 | na |  |  | 6692 | 0.480 | 0.1837 | No |
| 58 | KCNMA1 | na |  |  | 7209 | 0.421 | 0.1603 | No |
| 59 | MTA1 | na |  |  | 7219 | 0.420 | 0.1634 | No |
| 60 | PDLIM5 | na |  |  | 7457 | 0.391 | 0.1543 | No |
| 61 | VAV2 | na |  |  | 7580 | 0.375 | 0.1511 | No |
| 62 | SCHIP1 | na |  |  | 7720 | 0.360 | 0.1469 | No |
| 63 | RASA2 | na |  |  | 7751 | 0.356 | 0.1484 | No |
| 64 | KALRN | na |  |  | 7957 | 0.330 | 0.1404 | No |
| 65 | MRPS31 | na |  |  | 8136 | 0.313 | 0.1338 | No |
| 66 | BCKDHB | na |  |  | 8201 | 0.305 | 0.1330 | No |
| 67 | PIK3R3 | na |  |  | 8228 | 0.301 | 0.1342 | No |
| 68 | PRDM2 | na |  |  | 8426 | 0.282 | 0.1263 | No |
| 69 | CELF2 | na |  |  | 8461 | 0.277 | 0.1269 | No |
| 70 | CDK13 | na |  |  | 8791 | 0.237 | 0.1117 | No |
| 71 | SDC2 | na |  |  | 8869 | 0.228 | 0.1096 | No |
| 72 | TGFBR3 | na |  |  | 8951 | 0.217 | 0.1072 | No |
| 73 | CITED2 | na |  |  | 9844 | 0.121 | 0.0615 | No |
| 74 | CDC42BPA | na |  |  | 9923 | 0.113 | 0.0583 | No |
| 75 | SCAF8 | na |  |  | 10104 | 0.095 | 0.0497 | No |
| 76 | ADD3 | na |  |  | 10185 | 0.084 | 0.0462 | No |
| 77 | APBB2 | na |  |  | 10307 | 0.068 | 0.0405 | No |
| 78 | GJA1 | na |  |  | 10996 | -0.003 | 0.0044 | No |
| 79 | NFKB1 | na |  |  | 11078 | -0.014 | 0.0003 | No |
| 80 | ACVR2A | na |  |  | 11326 | -0.042 | -0.0123 | No |
| 81 | PTPRM | na |  |  | 11397 | -0.049 | -0.0156 | No |
| 82 | GRK5 | na |  |  | 11851 | -0.107 | -0.0384 | No |
| 83 | CACNA1A | na |  |  | 12532 | -0.189 | -0.0724 | No |
| 84 | PTEN | na |  |  | 12643 | -0.204 | -0.0765 | No |
| 85 | COL1A1 | na |  |  | 12777 | -0.227 | -0.0815 | No |
| 86 | RXRA | na |  |  | 13249 | -0.281 | -0.1038 | No |
| 87 | MAP2K5 | na |  |  | 13652 | -0.341 | -0.1220 | No |
| 88 | CDKN1B | na |  |  | 13864 | -0.360 | -0.1300 | No |
| 89 | PHF3 | na |  |  | 13935 | -0.368 | -0.1305 | No |
| 90 | ATRX | na |  |  | 13969 | -0.372 | -0.1290 | No |
| 91 | ABCC1 | na |  |  | 14177 | -0.402 | -0.1365 | No |
| 92 | IRS1 | na |  |  | 14327 | -0.421 | -0.1407 | No |
| 93 | DUSP1 | na |  |  | 14724 | -0.484 | -0.1573 | No |
| 94 | MMP16 | na |  |  | 14765 | -0.490 | -0.1552 | No |
| 95 | PMP22 | na |  |  | 15157 | -0.538 | -0.1711 | No |
| 96 | ERBB2 | na |  |  | 15220 | -0.550 | -0.1697 | No |
| 97 | FYN | na |  |  | 15435 | -0.588 | -0.1759 | No |
| 98 | SMAD3 | na |  |  | 15810 | -0.650 | -0.1899 | No |
| 99 | ATRN | na |  |  | 15977 | -0.682 | -0.1928 | No |
| 100 | PIK3CD | na |  |  | 15979 | -0.682 | -0.1870 | No |
| 101 | CDON | na |  |  | 16131 | -0.712 | -0.1889 | No |
| 102 | LAMC1 | na |  |  | 16327 | -0.763 | -0.1926 | No |
| 103 | AGGF1 | na |  |  | 16386 | -0.778 | -0.1890 | No |
| 104 | DAB2 | na |  |  | 16390 | -0.779 | -0.1825 | No |
| 105 | ID1 | na |  |  | 16701 | -0.857 | -0.1914 | No |
| 106 | DBP | na |  |  | 16887 | -0.915 | -0.1933 | No |
| 107 | ATP2B4 | na |  |  | 17125 | -0.987 | -0.1973 | No |
| 108 | IGF1R | na |  |  | 17220 | -1.013 | -0.1936 | No |
| 109 | PTGFR | na |  |  | 17255 | -1.023 | -0.1866 | No |
| 110 | ATXN1 | na |  |  | 17279 | -1.034 | -0.1790 | No |
| 111 | SYNJ2 | na |  |  | 17523 | -1.130 | -0.1821 | No |
| 112 | ZMIZ1 | na |  |  | 17603 | -1.149 | -0.1764 | No |
| 113 | LDLR | na |  |  | 17723 | -1.192 | -0.1724 | No |
| 114 | PIAS3 | na |  |  | 17726 | -1.193 | -0.1624 | No |
| 115 | INSIG1 | na |  |  | 17727 | -1.193 | -0.1522 | No |
| 116 | PEX14 | na |  |  | 17786 | -1.218 | -0.1448 | No |
| 117 | PDGFRB | na |  |  | 18059 | -1.357 | -0.1475 | No |
| 118 | ANXA4 | na |  |  | 18218 | -1.434 | -0.1435 | No |
| 119 | SIPA1L1 | na |  |  | 18229 | -1.444 | -0.1317 | No |
| 120 | NRP1 | na |  |  | 18351 | -1.527 | -0.1250 | No |
| 121 | BHLHE40 | na |  |  | 18387 | -1.556 | -0.1135 | No |
| 122 | SLC22A18 | na |  |  | 18521 | -1.664 | -0.1063 | No |
| 123 | TGFBR2 | na |  |  | 18636 | -1.798 | -0.0969 | No |
| 124 | COL1A2 | na |  |  | 18789 | -2.040 | -0.0874 | No |
| 125 | ICA1 | na |  |  | 18822 | -2.115 | -0.0711 | No |
| 126 | ITGB3 | na |  |  | 18931 | -2.380 | -0.0564 | No |
| 127 | VLDLR | na |  |  | 18950 | -2.433 | -0.0365 | No |
| 128 | TFPI | na |  |  | 18951 | -2.436 | -0.0157 | No |
| 129 | LTBP1 | na |  |  | 19122 | -3.351 | 0.0040 | No |
Table: GSEA details [plain text format]

  

Fig 2: HALLMARK\_UV\_RESPONSE\_DN      
 Blue-Pink O' Gram in the Space of the Analyzed GeneSet

  

Fig 3: HALLMARK\_UV\_RESPONSE\_DN: Random ES distribution      
 Gene set null distribution of ES for **HALLMARK\_UV\_RESPONSE\_DN**

  
