# Supplementary material for: Heparin-based hydrogel scaffolding alters the transcriptomic profile and increases the chemoresistance of MDA-MB-231 triple-negative breast cancer cells
Source: Biomater Sci. 2020 Feb 13;8(10):2786–96. doi: 10.1039/c9bm01481k (PMC7497406; doi:10.1039/c9bm01481k)
Supplement: Supplementary file 2 [file BM-008-C9BM01481K-s002.zip › Supplementary File 4/EGFvControl/Pathways/my_analysis.Gsea.1545200981068/HALLMARK_INTERFERON_ALPHA_RESPONSE.html]

Details for gene set HALLMARK\_INTERFERON\_ALPHA\_RESPONSE[GSEA]

|  || Dataset | expr.class.cls#EGF\_versus\_CONTROL.class.cls#EGF\_versus\_CONTROL\_repos |
| Phenotype | class.cls#EGF\_versus\_CONTROL\_repos |
| Upregulated in class | CONTROL |
| GeneSet | HALLMARK\_INTERFERON\_ALPHA\_RESPONSE |
| Enrichment Score (ES) | -0.50280875 |
| Normalized Enrichment Score (NES) | -2.2325604 |
| Nominal p-value | 0.0 |
| FDR q-value | 4.0000002E-4 |
| FWER p-Value | 0.001 |
Table: GSEA Results Summary

  

Fig 1: Enrichment plot: HALLMARK\_INTERFERON\_ALPHA\_RESPONSE      
 Profile of the Running ES Score & Positions of GeneSet Members on the Rank Ordered List

  

| PROBE | DESCRIPTION (from dataset) | GENE SYMBOL | GENE\_TITLE | RANK IN GENE LIST | RANK METRIC SCORE | RUNNING ES | CORE ENRICHMENT || 1 | PNPT1 | na |  |  | 320 | 1.926 | 0.0062 | No |
| 2 | TRIM14 | na |  |  | 363 | 1.877 | 0.0263 | No |
| 3 | ISG15 | na |  |  | 1964 | 1.231 | -0.0428 | No |
| 4 | USP18 | na |  |  | 2289 | 1.159 | -0.0459 | No |
| 5 | PARP12 | na |  |  | 2539 | 1.104 | -0.0458 | No |
| 6 | TRIM25 | na |  |  | 2663 | 1.082 | -0.0394 | No |
| 7 | RSAD2 | na |  |  | 3298 | 0.971 | -0.0610 | No |
| 8 | CASP8 | na |  |  | 3766 | 0.890 | -0.0749 | No |
| 9 | HERC6 | na |  |  | 4081 | 0.839 | -0.0813 | No |
| 10 | PSMA3 | na |  |  | 4449 | 0.785 | -0.0912 | No |
| 11 | LAP3 | na |  |  | 4650 | 0.756 | -0.0926 | No |
| 12 | IFI44L | na |  |  | 4958 | 0.713 | -0.1002 | No |
| 13 | SAMD9L | na |  |  | 5166 | 0.682 | -0.1029 | No |
| 14 | EIF2AK2 | na |  |  | 6165 | 0.547 | -0.1487 | No |
| 15 | RTP4 | na |  |  | 6227 | 0.536 | -0.1455 | No |
| 16 | GMPR | na |  |  | 6341 | 0.522 | -0.1452 | No |
| 17 | PROCR | na |  |  | 6383 | 0.516 | -0.1412 | No |
| 18 | NMI | na |  |  | 6667 | 0.482 | -0.1503 | No |
| 19 | SP110 | na |  |  | 6804 | 0.467 | -0.1518 | No |
| 20 | SAMD9 | na |  |  | 6901 | 0.456 | -0.1514 | No |
| 21 | TDRD7 | na |  |  | 6947 | 0.451 | -0.1484 | No |
| 22 | IFIT3 | na |  |  | 7001 | 0.447 | -0.1459 | No |
| 23 | LPAR6 | na |  |  | 7154 | 0.427 | -0.1487 | No |
| 24 | PSME2 | na |  |  | 7980 | 0.328 | -0.1880 | No |
| 25 | IRF2 | na |  |  | 8035 | 0.322 | -0.1870 | No |
| 26 | PLSCR1 | na |  |  | 8332 | 0.290 | -0.1991 | No |
| 27 | IFIT2 | na |  |  | 8545 | 0.266 | -0.2070 | No |
| 28 | TRIM26 | na |  |  | 8599 | 0.261 | -0.2066 | No |
| 29 | IFI44 | na |  |  | 8902 | 0.223 | -0.2198 | No |
| 30 | SLC25A28 | na |  |  | 9831 | 0.123 | -0.2669 | No |
| 31 | TRIM5 | na |  |  | 10199 | 0.082 | -0.2851 | No |
| 32 | CNP | na |  |  | 10235 | 0.077 | -0.2860 | No |
| 33 | CASP1 | na |  |  | 10391 | 0.060 | -0.2934 | No |
| 34 | CMPK2 | na |  |  | 10862 | 0.006 | -0.3180 | No |
| 35 | ADAR | na |  |  | 11005 | -0.004 | -0.3254 | No |
| 36 | DDX60 | na |  |  | 11988 | -0.124 | -0.3753 | No |
| 37 | WARS | na |  |  | 12021 | -0.127 | -0.3754 | No |
| 38 | ELF1 | na |  |  | 12748 | -0.221 | -0.4108 | No |
| 39 | LAMP3 | na |  |  | 13043 | -0.250 | -0.4232 | No |
| 40 | TRIM21 | na |  |  | 13344 | -0.295 | -0.4354 | No |
| 41 | IL7 | na |  |  | 13349 | -0.295 | -0.4321 | No |
| 42 | MX1 | na |  |  | 13486 | -0.314 | -0.4355 | No |
| 43 | OAS1 | na |  |  | 13532 | -0.321 | -0.4340 | No |
| 44 | RNF31 | na |  |  | 13607 | -0.331 | -0.4339 | No |
| 45 | IFI30 | na |  |  | 13832 | -0.356 | -0.4414 | No |
| 46 | CXCL10 | na |  |  | 13845 | -0.359 | -0.4378 | No |
| 47 | OASL | na |  |  | 14184 | -0.403 | -0.4507 | No |
| 48 | MOV10 | na |  |  | 14651 | -0.472 | -0.4694 | No |
| 49 | IRF7 | na |  |  | 14815 | -0.500 | -0.4720 | No |
| 50 | NUB1 | na |  |  | 15209 | -0.548 | -0.4861 | No |
| 51 | NCOA7 | na |  |  | 15338 | -0.572 | -0.4860 | No |
| 52 | RIPK2 | na |  |  | 15343 | -0.573 | -0.4794 | No |
| 53 | IFIH1 | na |  |  | 15792 | -0.647 | -0.4951 | Yes |
| 54 | IL15 | na |  |  | 15840 | -0.654 | -0.4898 | Yes |
| 55 | TRAFD1 | na |  |  | 15985 | -0.683 | -0.4892 | Yes |
| 56 | CCRL2 | na |  |  | 16115 | -0.706 | -0.4875 | Yes |
| 57 | IRF9 | na |  |  | 16304 | -0.759 | -0.4883 | Yes |
| 58 | IFI35 | na |  |  | 16370 | -0.774 | -0.4825 | Yes |
| 59 | UBE2L6 | na |  |  | 16481 | -0.806 | -0.4787 | Yes |
| 60 | OGFR | na |  |  | 16562 | -0.826 | -0.4731 | Yes |
| 61 | LY6E | na |  |  | 16861 | -0.908 | -0.4778 | Yes |
| 62 | PSME1 | na |  |  | 16865 | -0.910 | -0.4672 | Yes |
| 63 | IFITM1 | na |  |  | 17072 | -0.969 | -0.4664 | Yes |
| 64 | TMEM140 | na |  |  | 17094 | -0.975 | -0.4559 | Yes |
| 65 | PSMB8 | na |  |  | 17300 | -1.042 | -0.4543 | Yes |
| 66 | IFITM3 | na |  |  | 17388 | -1.072 | -0.4460 | Yes |
| 67 | BATF2 | na |  |  | 17393 | -1.073 | -0.4335 | Yes |
| 68 | TAP1 | na |  |  | 17413 | -1.084 | -0.4216 | Yes |
| 69 | IFITM2 | na |  |  | 17476 | -1.113 | -0.4116 | Yes |
| 70 | FAM46A | na |  |  | 17839 | -1.249 | -0.4157 | Yes |
| 71 | LGALS3BP | na |  |  | 17867 | -1.260 | -0.4021 | Yes |
| 72 | IRF1 | na |  |  | 18023 | -1.342 | -0.3942 | Yes |
| 73 | IL4R | na |  |  | 18107 | -1.380 | -0.3822 | Yes |
| 74 | STAT2 | na |  |  | 18119 | -1.384 | -0.3663 | Yes |
| 75 | PSMB9 | na |  |  | 18166 | -1.407 | -0.3519 | Yes |
| 76 | PARP14 | na |  |  | 18354 | -1.529 | -0.3435 | Yes |
| 77 | CD74 | na |  |  | 18418 | -1.591 | -0.3279 | Yes |
| 78 | CD47 | na |  |  | 18478 | -1.626 | -0.3116 | Yes |
| 79 | ISG20 | na |  |  | 18505 | -1.647 | -0.2934 | Yes |
| 80 | HLA-C | na |  |  | 18568 | -1.706 | -0.2763 | Yes |
| 81 | TXNIP | na |  |  | 18572 | -1.710 | -0.2561 | Yes |
| 82 | B2M | na |  |  | 18622 | -1.775 | -0.2376 | Yes |
| 83 | PARP9 | na |  |  | 18679 | -1.860 | -0.2184 | Yes |
| 84 | BST2 | na |  |  | 18856 | -2.210 | -0.2013 | Yes |
| 85 | C1S | na |  |  | 18893 | -2.301 | -0.1758 | Yes |
| 86 | DHX58 | na |  |  | 18900 | -2.322 | -0.1485 | Yes |
| 87 | GBP4 | na |  |  | 18924 | -2.365 | -0.1215 | Yes |
| 88 | UBA7 | na |  |  | 18962 | -2.492 | -0.0938 | Yes |
| 89 | GBP2 | na |  |  | 18974 | -2.541 | -0.0641 | Yes |
| 90 | IFI27 | na |  |  | 19088 | -3.055 | -0.0337 | Yes |
| 91 | CXCL11 | na |  |  | 19118 | -3.311 | 0.0042 | Yes |
Table: GSEA details [plain text format]

  

Fig 2: HALLMARK\_INTERFERON\_ALPHA\_RESPONSE      
 Blue-Pink O' Gram in the Space of the Analyzed GeneSet

  

Fig 3: HALLMARK\_INTERFERON\_ALPHA\_RESPONSE: Random ES distribution      
 Gene set null distribution of ES for **HALLMARK\_INTERFERON\_ALPHA\_RESPONSE**

  
