# Supplementary material for: Heparin-based hydrogel scaffolding alters the transcriptomic profile and increases the chemoresistance of MDA-MB-231 triple-negative breast cancer cells
Source: Biomater Sci. 2020 Feb 13;8(10):2786–96. doi: 10.1039/c9bm01481k (PMC7497406; doi:10.1039/c9bm01481k)
Supplement: Supplementary file 2 [file BM-008-C9BM01481K-s002.zip › Supplementary File 4/EGFvControl/Pathways/my_analysis.Gsea.1545200981068/HALLMARK_DNA_REPAIR.html]

Details for gene set HALLMARK\_DNA\_REPAIR[GSEA]

|  || Dataset | expr.class.cls#EGF\_versus\_CONTROL.class.cls#EGF\_versus\_CONTROL\_repos |
| Phenotype | class.cls#EGF\_versus\_CONTROL\_repos |
| Upregulated in class | EGF |
| GeneSet | HALLMARK\_DNA\_REPAIR |
| Enrichment Score (ES) | 0.36843103 |
| Normalized Enrichment Score (NES) | 1.6403146 |
| Nominal p-value | 0.001572327 |
| FDR q-value | 0.005391839 |
| FWER p-Value | 0.073 |
Table: GSEA Results Summary

  

Fig 1: Enrichment plot: HALLMARK\_DNA\_REPAIR      
 Profile of the Running ES Score & Positions of GeneSet Members on the Rank Ordered List

  

| PROBE | DESCRIPTION (from dataset) | GENE SYMBOL | GENE\_TITLE | RANK IN GENE LIST | RANK METRIC SCORE | RUNNING ES | CORE ENRICHMENT || 1 | FEN1 | na |  |  | 251 | 2.028 | 0.0046 | Yes |
| 2 | ZNRD1 | na |  |  | 524 | 1.756 | 0.0058 | Yes |
| 3 | UMPS | na |  |  | 526 | 1.756 | 0.0212 | Yes |
| 4 | PNP | na |  |  | 546 | 1.742 | 0.0355 | Yes |
| 5 | ERCC2 | na |  |  | 651 | 1.670 | 0.0447 | Yes |
| 6 | PRIM1 | na |  |  | 671 | 1.660 | 0.0582 | Yes |
| 7 | NME1 | na |  |  | 764 | 1.603 | 0.0675 | Yes |
| 8 | ZWINT | na |  |  | 817 | 1.582 | 0.0787 | Yes |
| 9 | RFC2 | na |  |  | 818 | 1.582 | 0.0925 | Yes |
| 10 | GTF2A2 | na |  |  | 977 | 1.520 | 0.0976 | Yes |
| 11 | RAE1 | na |  |  | 1065 | 1.490 | 0.1061 | Yes |
| 12 | TYMS | na |  |  | 1066 | 1.490 | 0.1192 | Yes |
| 13 | GTF2H1 | na |  |  | 1094 | 1.481 | 0.1308 | Yes |
| 14 | STX3 | na |  |  | 1133 | 1.466 | 0.1417 | Yes |
| 15 | POLA1 | na |  |  | 1137 | 1.465 | 0.1544 | Yes |
| 16 | NUDT21 | na |  |  | 1211 | 1.437 | 0.1632 | Yes |
| 17 | SNAPC5 | na |  |  | 1244 | 1.428 | 0.1740 | Yes |
| 18 | RFC3 | na |  |  | 1349 | 1.393 | 0.1808 | Yes |
| 19 | RFC4 | na |  |  | 1417 | 1.372 | 0.1893 | Yes |
| 20 | POLR2A | na |  |  | 1451 | 1.363 | 0.1996 | Yes |
| 21 | RAD52 | na |  |  | 1575 | 1.327 | 0.2048 | Yes |
| 22 | PCNA | na |  |  | 1712 | 1.291 | 0.2090 | Yes |
| 23 | POLR1C | na |  |  | 1728 | 1.289 | 0.2195 | Yes |
| 24 | GMPR2 | na |  |  | 1797 | 1.269 | 0.2271 | Yes |
| 25 | IMPDH2 | na |  |  | 1989 | 1.223 | 0.2278 | Yes |
| 26 | CLP1 | na |  |  | 2105 | 1.195 | 0.2323 | Yes |
| 27 | SF3A3 | na |  |  | 2170 | 1.185 | 0.2393 | Yes |
| 28 | SSRP1 | na |  |  | 2378 | 1.137 | 0.2384 | Yes |
| 29 | ARL6IP1 | na |  |  | 2385 | 1.136 | 0.2481 | Yes |
| 30 | DDB2 | na |  |  | 2488 | 1.113 | 0.2525 | Yes |
| 31 | POLA2 | na |  |  | 2529 | 1.105 | 0.2601 | Yes |
| 32 | POLE4 | na |  |  | 2559 | 1.100 | 0.2683 | Yes |
| 33 | POLD1 | na |  |  | 2630 | 1.089 | 0.2742 | Yes |
| 34 | TAF13 | na |  |  | 2636 | 1.088 | 0.2834 | Yes |
| 35 | TAF1C | na |  |  | 2653 | 1.084 | 0.2921 | Yes |
| 36 | HPRT1 | na |  |  | 2703 | 1.076 | 0.2990 | Yes |
| 37 | TAF6 | na |  |  | 2711 | 1.075 | 0.3081 | Yes |
| 38 | GTF3C5 | na |  |  | 2734 | 1.071 | 0.3163 | Yes |
| 39 | DGUOK | na |  |  | 2767 | 1.066 | 0.3240 | Yes |
| 40 | RFC5 | na |  |  | 2814 | 1.058 | 0.3309 | Yes |
| 41 | ERCC3 | na |  |  | 2881 | 1.047 | 0.3366 | Yes |
| 42 | POLR1D | na |  |  | 3041 | 1.018 | 0.3372 | Yes |
| 43 | RPA2 | na |  |  | 3128 | 1.001 | 0.3415 | Yes |
| 44 | POLH | na |  |  | 3221 | 0.982 | 0.3453 | Yes |
| 45 | POLD3 | na |  |  | 3244 | 0.979 | 0.3527 | Yes |
| 46 | TAF9 | na |  |  | 3290 | 0.971 | 0.3589 | Yes |
| 47 | RAD51 | na |  |  | 3425 | 0.948 | 0.3602 | Yes |
| 48 | GTF2H3 | na |  |  | 3656 | 0.913 | 0.3561 | Yes |
| 49 | SMAD5 | na |  |  | 3680 | 0.908 | 0.3629 | Yes |
| 50 | DUT | na |  |  | 3726 | 0.900 | 0.3684 | Yes |
| 51 | DGCR8 | na |  |  | 4265 | 0.813 | 0.3473 | No |
| 52 | NT5C | na |  |  | 4483 | 0.780 | 0.3428 | No |
| 53 | SAC3D1 | na |  |  | 4537 | 0.773 | 0.3468 | No |
| 54 | POLR2K | na |  |  | 4696 | 0.750 | 0.3451 | No |
| 55 | REV3L | na |  |  | 4765 | 0.736 | 0.3480 | No |
| 56 | POLR3C | na |  |  | 4779 | 0.734 | 0.3538 | No |
| 57 | POLR2G | na |  |  | 4797 | 0.731 | 0.3593 | No |
| 58 | LIG1 | na |  |  | 5038 | 0.702 | 0.3529 | No |
| 59 | RRM2B | na |  |  | 5044 | 0.701 | 0.3588 | No |
| 60 | ITPA | na |  |  | 5114 | 0.691 | 0.3612 | No |
| 61 | POLL | na |  |  | 5119 | 0.691 | 0.3671 | No |
| 62 | POLR2D | na |  |  | 5397 | 0.650 | 0.3582 | No |
| 63 | POLR2C | na |  |  | 5723 | 0.606 | 0.3465 | No |
| 64 | EIF1B | na |  |  | 5735 | 0.605 | 0.3512 | No |
| 65 | CSTF3 | na |  |  | 5922 | 0.581 | 0.3466 | No |
| 66 | NPR2 | na |  |  | 6034 | 0.567 | 0.3457 | No |
| 67 | NCBP2 | na |  |  | 6120 | 0.552 | 0.3461 | No |
| 68 | GTF2B | na |  |  | 6148 | 0.548 | 0.3495 | No |
| 69 | RPA3 | na |  |  | 6282 | 0.530 | 0.3472 | No |
| 70 | RALA | na |  |  | 6371 | 0.517 | 0.3471 | No |
| 71 | POLR2E | na |  |  | 6401 | 0.513 | 0.3501 | No |
| 72 | SURF1 | na |  |  | 6535 | 0.498 | 0.3475 | No |
| 73 | ADA | na |  |  | 6574 | 0.493 | 0.3498 | No |
| 74 | POM121 | na |  |  | 6656 | 0.484 | 0.3498 | No |
| 75 | TMED2 | na |  |  | 6719 | 0.478 | 0.3508 | No |
| 76 | SEC61A1 | na |  |  | 6734 | 0.476 | 0.3542 | No |
| 77 | RBX1 | na |  |  | 6955 | 0.450 | 0.3466 | No |
| 78 | DDB1 | na |  |  | 7041 | 0.441 | 0.3460 | No |
| 79 | NFX1 | na |  |  | 7087 | 0.435 | 0.3475 | No |
| 80 | BCAP31 | na |  |  | 7157 | 0.427 | 0.3476 | No |
| 81 | DCTN4 | na |  |  | 7195 | 0.423 | 0.3494 | No |
| 82 | CDA | na |  |  | 7578 | 0.376 | 0.3327 | No |
| 83 | SRSF6 | na |  |  | 7878 | 0.339 | 0.3200 | No |
| 84 | BOLA2 | na |  |  | 7966 | 0.329 | 0.3183 | No |
| 85 | MRPL40 | na |  |  | 8036 | 0.322 | 0.3175 | No |
| 86 | SNAPC4 | na |  |  | 8158 | 0.310 | 0.3139 | No |
| 87 | GTF2H5 | na |  |  | 8176 | 0.308 | 0.3157 | No |
| 88 | ADRM1 | na |  |  | 8507 | 0.271 | 0.3007 | No |
| 89 | CANT1 | na |  |  | 8515 | 0.269 | 0.3027 | No |
| 90 | TSG101 | na |  |  | 8709 | 0.245 | 0.2948 | No |
| 91 | DAD1 | na |  |  | 8884 | 0.226 | 0.2876 | No |
| 92 | ZNF707 | na |  |  | 9015 | 0.213 | 0.2827 | No |
| 93 | ELL | na |  |  | 9137 | 0.198 | 0.2781 | No |
| 94 | POLR2H | na |  |  | 9188 | 0.193 | 0.2771 | No |
| 95 | NUDT9 | na |  |  | 9266 | 0.186 | 0.2747 | No |
| 96 | TAF12 | na |  |  | 9372 | 0.176 | 0.2708 | No |
| 97 | TARBP2 | na |  |  | 9396 | 0.172 | 0.2711 | No |
| 98 | GUK1 | na |  |  | 9412 | 0.170 | 0.2718 | No |
| 99 | CCNO | na |  |  | 9632 | 0.146 | 0.2616 | No |
| 100 | ERCC1 | na |  |  | 9755 | 0.129 | 0.2563 | No |
| 101 | ERCC5 | na |  |  | 10584 | 0.040 | 0.2132 | No |
| 102 | ERCC4 | na |  |  | 10714 | 0.023 | 0.2066 | No |
| 103 | UPF3B | na |  |  | 10721 | 0.022 | 0.2065 | No |
| 104 | APRT | na |  |  | 10789 | 0.015 | 0.2031 | No |
| 105 | CMPK2 | na |  |  | 10862 | 0.006 | 0.1994 | No |
| 106 | POLB | na |  |  | 11738 | -0.091 | 0.1543 | No |
| 107 | ERCC8 | na |  |  | 11985 | -0.124 | 0.1425 | No |
| 108 | RNMT | na |  |  | 12192 | -0.143 | 0.1329 | No |
| 109 | POLR2J | na |  |  | 12320 | -0.157 | 0.1276 | No |
| 110 | BRF2 | na |  |  | 12359 | -0.164 | 0.1271 | No |
| 111 | SUPT5H | na |  |  | 12576 | -0.195 | 0.1175 | No |
| 112 | TAF10 | na |  |  | 12584 | -0.196 | 0.1188 | No |
| 113 | HCLS1 | na |  |  | 13062 | -0.251 | 0.0960 | No |
| 114 | VPS37B | na |  |  | 13365 | -0.297 | 0.0828 | No |
| 115 | AAAS | na |  |  | 13989 | -0.376 | 0.0534 | No |
| 116 | ADCY6 | na |  |  | 14106 | -0.393 | 0.0507 | No |
| 117 | GTF2F1 | na |  |  | 14192 | -0.405 | 0.0498 | No |
| 118 | TP53 | na |  |  | 14267 | -0.417 | 0.0496 | No |
| 119 | SDCBP | na |  |  | 14589 | -0.461 | 0.0368 | No |
| 120 | NME4 | na |  |  | 14599 | -0.463 | 0.0404 | No |
| 121 | CETN2 | na |  |  | 14630 | -0.467 | 0.0429 | No |
| 122 | USP11 | na |  |  | 16082 | -0.700 | -0.0271 | No |
| 123 | VPS28 | na |  |  | 16240 | -0.743 | -0.0288 | No |
| 124 | MPG | na |  |  | 16357 | -0.770 | -0.0281 | No |
| 125 | AK1 | na |  |  | 16503 | -0.811 | -0.0286 | No |
| 126 | GPX4 | na |  |  | 16614 | -0.837 | -0.0270 | No |
| 127 | SUPT4H1 | na |  |  | 16698 | -0.856 | -0.0238 | No |
| 128 | POLR2I | na |  |  | 16800 | -0.886 | -0.0213 | No |
| 129 | AK3 | na |  |  | 17069 | -0.968 | -0.0269 | No |
| 130 | POLR3GL | na |  |  | 17071 | -0.969 | -0.0185 | No |
| 131 | NME3 | na |  |  | 17119 | -0.983 | -0.0123 | No |
| 132 | EDF1 | na |  |  | 17699 | -1.186 | -0.0323 | No |
| 133 | COX17 | na |  |  | 18071 | -1.366 | -0.0397 | No |
| 134 | PDE4B | na |  |  | 18287 | -1.482 | -0.0380 | No |
| 135 | XPC | na |  |  | 18373 | -1.542 | -0.0289 | No |
| 136 | TK2 | na |  |  | 18425 | -1.599 | -0.0175 | No |
| 137 | VPS37D | na |  |  | 18643 | -1.806 | -0.0131 | No |
| 138 | BCAM | na |  |  | 18802 | -2.079 | -0.0031 | No |
| 139 | POLD4 | na |  |  | 19015 | -2.715 | 0.0096 | No |
Table: GSEA details [plain text format]

  

Fig 2: HALLMARK\_DNA\_REPAIR      
 Blue-Pink O' Gram in the Space of the Analyzed GeneSet

  

Fig 3: HALLMARK\_DNA\_REPAIR: Random ES distribution      
 Gene set null distribution of ES for **HALLMARK\_DNA\_REPAIR**

  
