# Supplementary material for: Heparin-based hydrogel scaffolding alters the transcriptomic profile and increases the chemoresistance of MDA-MB-231 triple-negative breast cancer cells
Source: Biomater Sci. 2020 Feb 13;8(10):2786–96. doi: 10.1039/c9bm01481k (PMC7497406; doi:10.1039/c9bm01481k)
Supplement: Supplementary file 2 [file BM-008-C9BM01481K-s002.zip › Supplementary File 4/EGFvControl/Pathways/my_analysis.Gsea.1545200981068/HALLMARK_APOPTOSIS.html]

Details for gene set HALLMARK\_APOPTOSIS[GSEA]

|  || Dataset | expr.class.cls#EGF\_versus\_CONTROL.class.cls#EGF\_versus\_CONTROL\_repos |
| Phenotype | class.cls#EGF\_versus\_CONTROL\_repos |
| Upregulated in class | CONTROL |
| GeneSet | HALLMARK\_APOPTOSIS |
| Enrichment Score (ES) | -0.37425458 |
| Normalized Enrichment Score (NES) | -1.7650607 |
| Nominal p-value | 0.0 |
| FDR q-value | 0.002575807 |
| FWER p-Value | 0.021 |
Table: GSEA Results Summary

  

Fig 1: Enrichment plot: HALLMARK\_APOPTOSIS      
 Profile of the Running ES Score & Positions of GeneSet Members on the Rank Ordered List

  

| PROBE | DESCRIPTION (from dataset) | GENE SYMBOL | GENE\_TITLE | RANK IN GENE LIST | RANK METRIC SCORE | RUNNING ES | CORE ENRICHMENT || 1 | IL18 | na |  |  | 152 | 2.202 | 0.0088 | No |
| 2 | SMAD7 | na |  |  | 195 | 2.108 | 0.0226 | No |
| 3 | CCNA1 | na |  |  | 344 | 1.897 | 0.0293 | No |
| 4 | PPT1 | na |  |  | 379 | 1.863 | 0.0417 | No |
| 5 | DFFA | na |  |  | 756 | 1.606 | 0.0342 | No |
| 6 | PEA15 | na |  |  | 840 | 1.573 | 0.0419 | No |
| 7 | DAP3 | na |  |  | 954 | 1.527 | 0.0476 | No |
| 8 | CASP3 | na |  |  | 1155 | 1.458 | 0.0482 | No |
| 9 | HMGB2 | na |  |  | 1183 | 1.446 | 0.0578 | No |
| 10 | GADD45B | na |  |  | 1283 | 1.417 | 0.0634 | No |
| 11 | CAV1 | na |  |  | 1542 | 1.335 | 0.0600 | No |
| 12 | BRCA1 | na |  |  | 1620 | 1.312 | 0.0660 | No |
| 13 | ETF1 | na |  |  | 1652 | 1.307 | 0.0743 | No |
| 14 | LEF1 | na |  |  | 1666 | 1.304 | 0.0835 | No |
| 15 | ROCK1 | na |  |  | 1702 | 1.294 | 0.0916 | No |
| 16 | CASP2 | na |  |  | 2078 | 1.201 | 0.0810 | No |
| 17 | TOP2A | na |  |  | 2151 | 1.189 | 0.0863 | No |
| 18 | CDK2 | na |  |  | 2311 | 1.153 | 0.0868 | No |
| 19 | CCND1 | na |  |  | 2400 | 1.133 | 0.0908 | No |
| 20 | DIABLO | na |  |  | 2583 | 1.097 | 0.0896 | No |
| 21 | SPTAN1 | na |  |  | 2725 | 1.072 | 0.0903 | No |
| 22 | HMOX1 | na |  |  | 3011 | 1.026 | 0.0832 | No |
| 23 | TNFRSF12A | na |  |  | 3094 | 1.008 | 0.0866 | No |
| 24 | KRT18 | na |  |  | 3236 | 0.980 | 0.0866 | No |
| 25 | CASP8 | na |  |  | 3766 | 0.890 | 0.0657 | No |
| 26 | DNAJA1 | na |  |  | 3884 | 0.871 | 0.0662 | No |
| 27 | CD44 | na |  |  | 3954 | 0.860 | 0.0691 | No |
| 28 | BTG3 | na |  |  | 3985 | 0.853 | 0.0740 | No |
| 29 | WEE1 | na |  |  | 3991 | 0.851 | 0.0802 | No |
| 30 | EBP | na |  |  | 4090 | 0.838 | 0.0815 | No |
| 31 | PTK2 | na |  |  | 4636 | 0.758 | 0.0586 | No |
| 32 | DPYD | na |  |  | 4744 | 0.739 | 0.0587 | No |
| 33 | CYLD | na |  |  | 4937 | 0.715 | 0.0540 | No |
| 34 | ANXA1 | na |  |  | 5268 | 0.669 | 0.0418 | No |
| 35 | DNM1L | na |  |  | 5311 | 0.663 | 0.0447 | No |
| 36 | CASP7 | na |  |  | 5606 | 0.625 | 0.0340 | No |
| 37 | PMAIP1 | na |  |  | 6092 | 0.558 | 0.0128 | No |
| 38 | GADD45A | na |  |  | 6102 | 0.555 | 0.0166 | No |
| 39 | PLCB2 | na |  |  | 6129 | 0.551 | 0.0194 | No |
| 40 | FAS | na |  |  | 6266 | 0.532 | 0.0163 | No |
| 41 | DDIT3 | na |  |  | 6794 | 0.469 | -0.0078 | No |
| 42 | GCH1 | na |  |  | 6881 | 0.459 | -0.0088 | No |
| 43 | BCAP31 | na |  |  | 7157 | 0.427 | -0.0200 | No |
| 44 | ATF3 | na |  |  | 7410 | 0.395 | -0.0302 | No |
| 45 | F2R | na |  |  | 7430 | 0.393 | -0.0282 | No |
| 46 | FDXR | na |  |  | 7707 | 0.362 | -0.0399 | No |
| 47 | VDAC2 | na |  |  | 7830 | 0.346 | -0.0437 | No |
| 48 | SOD1 | na |  |  | 7945 | 0.332 | -0.0471 | No |
| 49 | CTH | na |  |  | 7970 | 0.329 | -0.0459 | No |
| 50 | BAX | na |  |  | 8092 | 0.317 | -0.0498 | No |
| 51 | TGFB2 | na |  |  | 8117 | 0.315 | -0.0487 | No |
| 52 | TGFBR3 | na |  |  | 8951 | 0.217 | -0.0907 | No |
| 53 | BCL2L2 | na |  |  | 9105 | 0.201 | -0.0972 | No |
| 54 | FEZ1 | na |  |  | 9229 | 0.190 | -0.1022 | No |
| 55 | SLC20A1 | na |  |  | 9338 | 0.179 | -0.1065 | No |
| 56 | RHOT2 | na |  |  | 9543 | 0.152 | -0.1161 | No |
| 57 | GSTM1 | na |  |  | 9772 | 0.128 | -0.1271 | No |
| 58 | CREBBP | na |  |  | 9790 | 0.126 | -0.1270 | No |
| 59 | NEFH | na |  |  | 10044 | 0.100 | -0.1395 | No |
| 60 | CASP1 | na |  |  | 10391 | 0.060 | -0.1572 | No |
| 61 | CASP4 | na |  |  | 10964 | 0.000 | -0.1872 | No |
| 62 | PSEN2 | na |  |  | 11451 | -0.056 | -0.2123 | No |
| 63 | MADD | na |  |  | 11651 | -0.078 | -0.2221 | No |
| 64 | BTG2 | na |  |  | 11938 | -0.118 | -0.2363 | No |
| 65 | CDC25B | na |  |  | 12061 | -0.134 | -0.2416 | No |
| 66 | PPP3R1 | na |  |  | 12294 | -0.154 | -0.2526 | No |
| 67 | BCL2L1 | na |  |  | 12327 | -0.159 | -0.2531 | No |
| 68 | RETSAT | na |  |  | 12351 | -0.162 | -0.2531 | No |
| 69 | MCL1 | na |  |  | 12451 | -0.179 | -0.2569 | No |
| 70 | ADD1 | na |  |  | 12475 | -0.181 | -0.2567 | No |
| 71 | JUN | na |  |  | 12605 | -0.199 | -0.2620 | No |
| 72 | PPP2R5B | na |  |  | 12652 | -0.205 | -0.2628 | No |
| 73 | CTNNB1 | na |  |  | 13218 | -0.277 | -0.2904 | No |
| 74 | TIMP2 | na |  |  | 13317 | -0.291 | -0.2933 | No |
| 75 | TSPO | na |  |  | 13324 | -0.292 | -0.2914 | No |
| 76 | PAK1 | na |  |  | 13679 | -0.342 | -0.3074 | No |
| 77 | BID | na |  |  | 13709 | -0.347 | -0.3063 | No |
| 78 | CASP9 | na |  |  | 13830 | -0.355 | -0.3098 | No |
| 79 | CDKN1B | na |  |  | 13864 | -0.360 | -0.3088 | No |
| 80 | CASP6 | na |  |  | 13966 | -0.372 | -0.3113 | No |
| 81 | DNAJC3 | na |  |  | 14042 | -0.384 | -0.3123 | No |
| 82 | GPX1 | na |  |  | 14229 | -0.410 | -0.3189 | No |
| 83 | HSPB1 | na |  |  | 14244 | -0.413 | -0.3165 | No |
| 84 | GSR | na |  |  | 14564 | -0.456 | -0.3298 | No |
| 85 | RELA | na |  |  | 14752 | -0.488 | -0.3359 | No |
| 86 | PSEN1 | na |  |  | 15031 | -0.515 | -0.3466 | No |
| 87 | XIAP | na |  |  | 15171 | -0.541 | -0.3497 | No |
| 88 | ERBB2 | na |  |  | 15220 | -0.550 | -0.3481 | No |
| 89 | IGF2R | na |  |  | 15552 | -0.602 | -0.3608 | No |
| 90 | GNA15 | na |  |  | 15638 | -0.613 | -0.3606 | No |
| 91 | TNF | na |  |  | 15826 | -0.653 | -0.3655 | No |
| 92 | CDKN1A | na |  |  | 15950 | -0.678 | -0.3668 | No |
| 93 | IL1A | na |  |  | 16094 | -0.702 | -0.3689 | Yes |
| 94 | DAP | na |  |  | 16189 | -0.729 | -0.3683 | Yes |
| 95 | GPX3 | na |  |  | 16209 | -0.732 | -0.3637 | Yes |
| 96 | H1F0 | na |  |  | 16221 | -0.737 | -0.3587 | Yes |
| 97 | ERBB3 | na |  |  | 16345 | -0.768 | -0.3593 | Yes |
| 98 | RARA | na |  |  | 16355 | -0.770 | -0.3539 | Yes |
| 99 | RHOB | na |  |  | 16399 | -0.781 | -0.3502 | Yes |
| 100 | BCL2L11 | na |  |  | 16498 | -0.810 | -0.3492 | Yes |
| 101 | CD14 | na |  |  | 16595 | -0.833 | -0.3479 | Yes |
| 102 | EMP1 | na |  |  | 16599 | -0.834 | -0.3417 | Yes |
| 103 | GPX4 | na |  |  | 16614 | -0.837 | -0.3360 | Yes |
| 104 | BCL10 | na |  |  | 16617 | -0.838 | -0.3297 | Yes |
| 105 | SQSTM1 | na |  |  | 16663 | -0.848 | -0.3256 | Yes |
| 106 | EGR3 | na |  |  | 16779 | -0.878 | -0.3250 | Yes |
| 107 | EREG | na |  |  | 16952 | -0.936 | -0.3269 | Yes |
| 108 | ANKH | na |  |  | 17223 | -1.014 | -0.3333 | Yes |
| 109 | IFNGR1 | na |  |  | 17299 | -1.042 | -0.3293 | Yes |
| 110 | BIK | na |  |  | 17323 | -1.050 | -0.3225 | Yes |
| 111 | CCND2 | na |  |  | 17385 | -1.070 | -0.3176 | Yes |
| 112 | IFITM3 | na |  |  | 17388 | -1.072 | -0.3095 | Yes |
| 113 | TAP1 | na |  |  | 17413 | -1.084 | -0.3025 | Yes |
| 114 | BNIP3L | na |  |  | 17581 | -1.142 | -0.3026 | Yes |
| 115 | TIMP1 | na |  |  | 17600 | -1.148 | -0.2948 | Yes |
| 116 | IRF1 | na |  |  | 18023 | -1.342 | -0.3067 | Yes |
| 117 | PDGFRB | na |  |  | 18059 | -1.357 | -0.2982 | Yes |
| 118 | BMF | na |  |  | 18174 | -1.411 | -0.2935 | Yes |
| 119 | IL1B | na |  |  | 18217 | -1.433 | -0.2847 | Yes |
| 120 | GSN | na |  |  | 18275 | -1.477 | -0.2765 | Yes |
| 121 | ENO2 | na |  |  | 18286 | -1.481 | -0.2657 | Yes |
| 122 | RNASEL | na |  |  | 18309 | -1.502 | -0.2554 | Yes |
| 123 | APP | na |  |  | 18352 | -1.528 | -0.2460 | Yes |
| 124 | NEDD9 | na |  |  | 18380 | -1.552 | -0.2356 | Yes |
| 125 | CFLAR | na |  |  | 18417 | -1.589 | -0.2254 | Yes |
| 126 | TNFSF10 | na |  |  | 18488 | -1.633 | -0.2166 | Yes |
| 127 | CLU | na |  |  | 18498 | -1.641 | -0.2046 | Yes |
| 128 | ISG20 | na |  |  | 18505 | -1.647 | -0.1924 | Yes |
| 129 | TXNIP | na |  |  | 18572 | -1.710 | -0.1828 | Yes |
| 130 | PLAT | na |  |  | 18660 | -1.832 | -0.1734 | Yes |
| 131 | LGALS3 | na |  |  | 18663 | -1.837 | -0.1595 | Yes |
| 132 | IL6 | na |  |  | 18776 | -2.020 | -0.1500 | Yes |
| 133 | TIMP3 | na |  |  | 18862 | -2.233 | -0.1375 | Yes |
| 134 | SOD2 | na |  |  | 18970 | -2.521 | -0.1239 | Yes |
| 135 | IGFBP6 | na |  |  | 18978 | -2.554 | -0.1048 | Yes |
| 136 | PDCD4 | na |  |  | 18987 | -2.595 | -0.0855 | Yes |
| 137 | BIRC3 | na |  |  | 19043 | -2.872 | -0.0665 | Yes |
| 138 | SATB1 | na |  |  | 19064 | -2.931 | -0.0452 | Yes |
| 139 | BMP2 | na |  |  | 19119 | -3.339 | -0.0226 | Yes |
| 140 | SAT1 | na |  |  | 19139 | -3.506 | 0.0031 | Yes |
Table: GSEA details [plain text format]

  

Fig 2: HALLMARK\_APOPTOSIS      
 Blue-Pink O' Gram in the Space of the Analyzed GeneSet

  

Fig 3: HALLMARK\_APOPTOSIS: Random ES distribution      
 Gene set null distribution of ES for **HALLMARK\_APOPTOSIS**

  
