# Supplementary material for: Heparin-based hydrogel scaffolding alters the transcriptomic profile and increases the chemoresistance of MDA-MB-231 triple-negative breast cancer cells
Source: Biomater Sci. 2020 Feb 13;8(10):2786–96. doi: 10.1039/c9bm01481k (PMC7497406; doi:10.1039/c9bm01481k)
Supplement: Supplementary file 2 [file BM-008-C9BM01481K-s002.zip › Supplementary File 4/EGFvControl/Pathways/my_analysis.Gsea.1545200981068/HALLMARK_APICAL_JUNCTION.html]

Details for gene set HALLMARK\_APICAL\_JUNCTION[GSEA]

|  || Dataset | expr.class.cls#EGF\_versus\_CONTROL.class.cls#EGF\_versus\_CONTROL\_repos |
| Phenotype | class.cls#EGF\_versus\_CONTROL\_repos |
| Upregulated in class | CONTROL |
| GeneSet | HALLMARK\_APICAL\_JUNCTION |
| Enrichment Score (ES) | -0.37161815 |
| Normalized Enrichment Score (NES) | -1.7262362 |
| Nominal p-value | 0.0 |
| FDR q-value | 0.003731225 |
| FWER p-Value | 0.032 |
Table: GSEA Results Summary

  

Fig 1: Enrichment plot: HALLMARK\_APICAL\_JUNCTION      
 Profile of the Running ES Score & Positions of GeneSet Members on the Rank Ordered List

  

| PROBE | DESCRIPTION (from dataset) | GENE SYMBOL | GENE\_TITLE | RANK IN GENE LIST | RANK METRIC SCORE | RUNNING ES | CORE ENRICHMENT || 1 | CDH4 | na |  |  | 61 | 2.564 | 0.0160 | No |
| 2 | NEGR1 | na |  |  | 167 | 2.174 | 0.0267 | No |
| 3 | CDK8 | na |  |  | 432 | 1.818 | 0.0265 | No |
| 4 | AMIGO2 | na |  |  | 521 | 1.757 | 0.0350 | No |
| 5 | PARD6G | na |  |  | 604 | 1.702 | 0.0434 | No |
| 6 | CD274 | na |  |  | 836 | 1.574 | 0.0431 | No |
| 7 | LIMA1 | na |  |  | 928 | 1.541 | 0.0499 | No |
| 8 | NEXN | na |  |  | 1227 | 1.434 | 0.0449 | No |
| 9 | EGFR | na |  |  | 1241 | 1.429 | 0.0549 | No |
| 10 | FBN1 | na |  |  | 1368 | 1.388 | 0.0587 | No |
| 11 | NF2 | na |  |  | 1754 | 1.279 | 0.0481 | No |
| 12 | MYH10 | na |  |  | 1933 | 1.239 | 0.0480 | No |
| 13 | COL16A1 | na |  |  | 2030 | 1.212 | 0.0520 | No |
| 14 | COL17A1 | na |  |  | 2111 | 1.194 | 0.0568 | No |
| 15 | MPP5 | na |  |  | 2200 | 1.178 | 0.0610 | No |
| 16 | ACTN1 | na |  |  | 2399 | 1.133 | 0.0591 | No |
| 17 | ICAM2 | na |  |  | 2686 | 1.079 | 0.0521 | No |
| 18 | TIAL1 | na |  |  | 2910 | 1.042 | 0.0482 | No |
| 19 | PFN1 | na |  |  | 3018 | 1.025 | 0.0503 | No |
| 20 | MAPK14 | na |  |  | 3187 | 0.990 | 0.0489 | No |
| 21 | TNFRSF11B | na |  |  | 3241 | 0.979 | 0.0534 | No |
| 22 | GNAI1 | na |  |  | 3405 | 0.953 | 0.0520 | No |
| 23 | LAYN | na |  |  | 3577 | 0.924 | 0.0499 | No |
| 24 | DLG1 | na |  |  | 3579 | 0.924 | 0.0568 | No |
| 25 | AKT3 | na |  |  | 3603 | 0.919 | 0.0624 | No |
| 26 | YWHAH | na |  |  | 3864 | 0.875 | 0.0553 | No |
| 27 | TJP1 | na |  |  | 4454 | 0.784 | 0.0303 | No |
| 28 | RASA1 | na |  |  | 4463 | 0.783 | 0.0357 | No |
| 29 | EPB41L2 | na |  |  | 4626 | 0.759 | 0.0329 | No |
| 30 | PTK2 | na |  |  | 4636 | 0.758 | 0.0381 | No |
| 31 | VCL | na |  |  | 4660 | 0.755 | 0.0426 | No |
| 32 | LDLRAP1 | na |  |  | 4817 | 0.727 | 0.0398 | No |
| 33 | ZYX | na |  |  | 5112 | 0.691 | 0.0295 | No |
| 34 | CNN2 | na |  |  | 5140 | 0.686 | 0.0333 | No |
| 35 | ARPC2 | na |  |  | 5229 | 0.674 | 0.0337 | No |
| 36 | SHROOM2 | na |  |  | 5400 | 0.650 | 0.0296 | No |
| 37 | CAP1 | na |  |  | 5536 | 0.634 | 0.0273 | No |
| 38 | HRAS | na |  |  | 5618 | 0.623 | 0.0277 | No |
| 39 | AMH | na |  |  | 5712 | 0.607 | 0.0273 | No |
| 40 | ADAM9 | na |  |  | 5789 | 0.598 | 0.0278 | No |
| 41 | NF1 | na |  |  | 5879 | 0.587 | 0.0276 | No |
| 42 | MYL12B | na |  |  | 5961 | 0.575 | 0.0276 | No |
| 43 | ADAM23 | na |  |  | 5986 | 0.571 | 0.0306 | No |
| 44 | BAIAP2 | na |  |  | 6000 | 0.570 | 0.0342 | No |
| 45 | AMIGO1 | na |  |  | 6082 | 0.559 | 0.0341 | No |
| 46 | CD34 | na |  |  | 6203 | 0.541 | 0.0319 | No |
| 47 | VASP | na |  |  | 6458 | 0.508 | 0.0224 | No |
| 48 | PCDH1 | na |  |  | 6708 | 0.479 | 0.0129 | No |
| 49 | SHC1 | na |  |  | 6751 | 0.474 | 0.0142 | No |
| 50 | ATP1A3 | na |  |  | 7402 | 0.396 | -0.0169 | No |
| 51 | VAV2 | na |  |  | 7580 | 0.375 | -0.0234 | No |
| 52 | TSPAN4 | na |  |  | 7663 | 0.367 | -0.0250 | No |
| 53 | IKBKG | na |  |  | 7781 | 0.352 | -0.0285 | No |
| 54 | RSU1 | na |  |  | 7812 | 0.348 | -0.0275 | No |
| 55 | PARVA | na |  |  | 7900 | 0.336 | -0.0295 | No |
| 56 | CLDN11 | na |  |  | 8126 | 0.314 | -0.0390 | No |
| 57 | PIK3R3 | na |  |  | 8228 | 0.301 | -0.0420 | No |
| 58 | CDH1 | na |  |  | 8288 | 0.294 | -0.0429 | No |
| 59 | EXOC4 | na |  |  | 8296 | 0.293 | -0.0411 | No |
| 60 | VCAN | na |  |  | 8574 | 0.265 | -0.0537 | No |
| 61 | TUBG1 | na |  |  | 8708 | 0.245 | -0.0588 | No |
| 62 | WNK4 | na |  |  | 8822 | 0.235 | -0.0630 | No |
| 63 | EVL | na |  |  | 9365 | 0.177 | -0.0901 | No |
| 64 | HADH | na |  |  | 9445 | 0.164 | -0.0930 | No |
| 65 | MYL9 | na |  |  | 9571 | 0.148 | -0.0985 | No |
| 66 | CLDN9 | na |  |  | 9823 | 0.125 | -0.1107 | No |
| 67 | MAPK11 | na |  |  | 9840 | 0.122 | -0.1106 | No |
| 68 | MPZL1 | na |  |  | 10173 | 0.085 | -0.1274 | No |
| 69 | GAMT | na |  |  | 10799 | 0.014 | -0.1601 | No |
| 70 | JAM3 | na |  |  | 10852 | 0.007 | -0.1628 | No |
| 71 | PKD1 | na |  |  | 11013 | -0.005 | -0.1712 | No |
| 72 | DHX16 | na |  |  | 11131 | -0.018 | -0.1772 | No |
| 73 | CDSN | na |  |  | 11376 | -0.047 | -0.1896 | No |
| 74 | AKT2 | na |  |  | 11631 | -0.075 | -0.2024 | No |
| 75 | CLDN18 | na |  |  | 11680 | -0.082 | -0.2043 | No |
| 76 | PIK3CB | na |  |  | 11960 | -0.120 | -0.2181 | No |
| 77 | CLDN14 | na |  |  | 12149 | -0.136 | -0.2269 | No |
| 78 | MADCAM1 | na |  |  | 12377 | -0.166 | -0.2376 | No |
| 79 | CLDN15 | na |  |  | 12531 | -0.189 | -0.2442 | No |
| 80 | CTNNA1 | na |  |  | 12562 | -0.192 | -0.2443 | No |
| 81 | PTEN | na |  |  | 12643 | -0.204 | -0.2470 | No |
| 82 | MAP4K2 | na |  |  | 12685 | -0.210 | -0.2476 | No |
| 83 | TSC1 | na |  |  | 12942 | -0.238 | -0.2592 | No |
| 84 | CDH3 | na |  |  | 12977 | -0.242 | -0.2592 | No |
| 85 | SORBS3 | na |  |  | 13029 | -0.248 | -0.2600 | No |
| 86 | SYMPK | na |  |  | 13202 | -0.274 | -0.2670 | No |
| 87 | SIRPA | na |  |  | 13307 | -0.288 | -0.2703 | No |
| 88 | PLCG1 | na |  |  | 13591 | -0.329 | -0.2827 | No |
| 89 | TAOK2 | na |  |  | 13961 | -0.371 | -0.2993 | No |
| 90 | CDH15 | na |  |  | 14007 | -0.379 | -0.2988 | No |
| 91 | GTF2F1 | na |  |  | 14192 | -0.405 | -0.3055 | No |
| 92 | IRS1 | na |  |  | 14327 | -0.421 | -0.3093 | No |
| 93 | CD276 | na |  |  | 14475 | -0.441 | -0.3138 | No |
| 94 | WASL | na |  |  | 14807 | -0.498 | -0.3274 | No |
| 95 | CALB2 | na |  |  | 14948 | -0.506 | -0.3310 | No |
| 96 | VCAM1 | na |  |  | 15075 | -0.525 | -0.3336 | No |
| 97 | MPZL2 | na |  |  | 15081 | -0.526 | -0.3300 | No |
| 98 | LAMB3 | na |  |  | 15267 | -0.557 | -0.3355 | No |
| 99 | MVD | na |  |  | 15274 | -0.559 | -0.3316 | No |
| 100 | ADAM15 | na |  |  | 15285 | -0.561 | -0.3280 | No |
| 101 | SGCE | na |  |  | 15791 | -0.646 | -0.3496 | No |
| 102 | INPPL1 | na |  |  | 15860 | -0.659 | -0.3483 | No |
| 103 | NLGN2 | na |  |  | 16112 | -0.706 | -0.3562 | No |
| 104 | FSCN1 | na |  |  | 16186 | -0.728 | -0.3546 | No |
| 105 | GRB7 | na |  |  | 16192 | -0.730 | -0.3494 | No |
| 106 | NRTN | na |  |  | 16321 | -0.762 | -0.3504 | No |
| 107 | RAC2 | na |  |  | 16465 | -0.801 | -0.3519 | No |
| 108 | GNAI2 | na |  |  | 16842 | -0.901 | -0.3649 | Yes |
| 109 | ADRA1B | na |  |  | 16846 | -0.904 | -0.3583 | Yes |
| 110 | ARHGEF6 | na |  |  | 16873 | -0.912 | -0.3528 | Yes |
| 111 | PBX2 | na |  |  | 16902 | -0.921 | -0.3474 | Yes |
| 112 | SRC | na |  |  | 16943 | -0.934 | -0.3425 | Yes |
| 113 | CTNND1 | na |  |  | 16974 | -0.944 | -0.3370 | Yes |
| 114 | CLDN4 | na |  |  | 17113 | -0.982 | -0.3369 | Yes |
| 115 | SLIT2 | na |  |  | 17135 | -0.990 | -0.3306 | Yes |
| 116 | CD99 | na |  |  | 17216 | -1.012 | -0.3272 | Yes |
| 117 | CERCAM | na |  |  | 17237 | -1.017 | -0.3207 | Yes |
| 118 | ICAM5 | na |  |  | 17264 | -1.026 | -0.3144 | Yes |
| 119 | SKAP2 | na |  |  | 17486 | -1.118 | -0.3176 | Yes |
| 120 | B4GALT1 | na |  |  | 17619 | -1.157 | -0.3159 | Yes |
| 121 | TMEM8B | na |  |  | 17632 | -1.161 | -0.3078 | Yes |
| 122 | INSIG1 | na |  |  | 17727 | -1.193 | -0.3038 | Yes |
| 123 | SDC3 | na |  |  | 17812 | -1.229 | -0.2990 | Yes |
| 124 | TGFBI | na |  |  | 17865 | -1.259 | -0.2923 | Yes |
| 125 | JUP | na |  |  | 17913 | -1.281 | -0.2852 | Yes |
| 126 | ICAM1 | na |  |  | 17954 | -1.306 | -0.2775 | Yes |
| 127 | MAPK13 | na |  |  | 17998 | -1.329 | -0.2699 | Yes |
| 128 | STX4 | na |  |  | 18000 | -1.329 | -0.2600 | Yes |
| 129 | RHOF | na |  |  | 18083 | -1.373 | -0.2540 | Yes |
| 130 | ITGB4 | na |  |  | 18146 | -1.397 | -0.2468 | Yes |
| 131 | RRAS | na |  |  | 18220 | -1.437 | -0.2399 | Yes |
| 132 | CRAT | na |  |  | 18281 | -1.480 | -0.2320 | Yes |
| 133 | SPEG | na |  |  | 18434 | -1.607 | -0.2279 | Yes |
| 134 | TRAF1 | na |  |  | 18477 | -1.625 | -0.2180 | Yes |
| 135 | THBS3 | na |  |  | 18592 | -1.742 | -0.2109 | Yes |
| 136 | CX3CL1 | na |  |  | 18661 | -1.834 | -0.2008 | Yes |
| 137 | CLDN7 | na |  |  | 18704 | -1.903 | -0.1887 | Yes |
| 138 | ITGA2 | na |  |  | 18843 | -2.164 | -0.1798 | Yes |
| 139 | CDH11 | na |  |  | 18848 | -2.179 | -0.1637 | Yes |
| 140 | BMP1 | na |  |  | 18897 | -2.311 | -0.1489 | Yes |
| 141 | LAMC2 | na |  |  | 18985 | -2.588 | -0.1341 | Yes |
| 142 | PPP2R2C | na |  |  | 19009 | -2.704 | -0.1151 | Yes |
| 143 | LAMA3 | na |  |  | 19037 | -2.819 | -0.0954 | Yes |
| 144 | NLGN3 | na |  |  | 19091 | -3.077 | -0.0752 | Yes |
| 145 | MDK | na |  |  | 19103 | -3.166 | -0.0521 | Yes |
| 146 | ITGA10 | na |  |  | 19142 | -3.579 | -0.0273 | Yes |
| 147 | PECAM1 | na |  |  | 19167 | -4.033 | 0.0016 | Yes |
Table: GSEA details [plain text format]

  

Fig 2: HALLMARK\_APICAL\_JUNCTION      
 Blue-Pink O' Gram in the Space of the Analyzed GeneSet

  

Fig 3: HALLMARK\_APICAL\_JUNCTION: Random ES distribution      
 Gene set null distribution of ES for **HALLMARK\_APICAL\_JUNCTION**

  
