# Supplementary material for: Heparin-based hydrogel scaffolding alters the transcriptomic profile and increases the chemoresistance of MDA-MB-231 triple-negative breast cancer cells
Source: Biomater Sci. 2020 Feb 13;8(10):2786–96. doi: 10.1039/c9bm01481k (PMC7497406; doi:10.1039/c9bm01481k)
Supplement: Supplementary file 2 [file BM-008-C9BM01481K-s002.zip › Supplementary File 4/EGFvControl/Pathways/my_analysis.Gsea.1545200981068/HALLMARK_INFLAMMATORY_RESPONSE.html]

Details for gene set HALLMARK\_INFLAMMATORY\_RESPONSE[GSEA]

|  || Dataset | expr.class.cls#EGF\_versus\_CONTROL.class.cls#EGF\_versus\_CONTROL\_repos |
| Phenotype | class.cls#EGF\_versus\_CONTROL\_repos |
| Upregulated in class | CONTROL |
| GeneSet | HALLMARK\_INFLAMMATORY\_RESPONSE |
| Enrichment Score (ES) | -0.42986503 |
| Normalized Enrichment Score (NES) | -2.0012493 |
| Nominal p-value | 0.0 |
| FDR q-value | 3.9153438E-4 |
| FWER p-Value | 0.002 |
Table: GSEA Results Summary

  

Fig 1: Enrichment plot: HALLMARK\_INFLAMMATORY\_RESPONSE      
 Profile of the Running ES Score & Positions of GeneSet Members on the Rank Ordered List

  

| PROBE | DESCRIPTION (from dataset) | GENE SYMBOL | GENE\_TITLE | RANK IN GENE LIST | RANK METRIC SCORE | RUNNING ES | CORE ENRICHMENT || 1 | EDN1 | na |  |  | 77 | 2.466 | 0.0156 | No |
| 2 | IL18 | na |  |  | 152 | 2.202 | 0.0293 | No |
| 3 | SLC7A1 | na |  |  | 311 | 1.936 | 0.0365 | No |
| 4 | ADORA2B | na |  |  | 492 | 1.775 | 0.0412 | No |
| 5 | F3 | na |  |  | 545 | 1.742 | 0.0524 | No |
| 6 | SPHK1 | na |  |  | 643 | 1.674 | 0.0607 | No |
| 7 | NLRP3 | na |  |  | 1376 | 1.386 | 0.0334 | No |
| 8 | IL7R | na |  |  | 1696 | 1.295 | 0.0270 | No |
| 9 | RASGRP1 | na |  |  | 1769 | 1.276 | 0.0334 | No |
| 10 | SLC31A1 | na |  |  | 2028 | 1.213 | 0.0295 | No |
| 11 | PTGER4 | na |  |  | 2110 | 1.194 | 0.0348 | No |
| 12 | SLC7A2 | na |  |  | 2354 | 1.144 | 0.0312 | No |
| 13 | ATP2B1 | na |  |  | 2361 | 1.142 | 0.0400 | No |
| 14 | GNAI3 | na |  |  | 2417 | 1.129 | 0.0461 | No |
| 15 | PVR | na |  |  | 2784 | 1.062 | 0.0354 | No |
| 16 | SGMS2 | na |  |  | 3190 | 0.990 | 0.0221 | No |
| 17 | MET | na |  |  | 3522 | 0.933 | 0.0122 | No |
| 18 | HBEGF | na |  |  | 3593 | 0.921 | 0.0158 | No |
| 19 | RAF1 | na |  |  | 3823 | 0.880 | 0.0109 | No |
| 20 | MYC | na |  |  | 3907 | 0.866 | 0.0134 | No |
| 21 | LPAR1 | na |  |  | 4142 | 0.830 | 0.0078 | No |
| 22 | ATP2A2 | na |  |  | 4160 | 0.828 | 0.0135 | No |
| 23 | PCDH7 | na |  |  | 4195 | 0.823 | 0.0183 | No |
| 24 | SRI | na |  |  | 4299 | 0.808 | 0.0193 | No |
| 25 | FZD5 | na |  |  | 4494 | 0.779 | 0.0154 | No |
| 26 | HRH1 | na |  |  | 4651 | 0.756 | 0.0132 | No |
| 27 | SLC4A4 | na |  |  | 5176 | 0.681 | -0.0088 | No |
| 28 | EIF2AK2 | na |  |  | 6165 | 0.547 | -0.0563 | No |
| 29 | RTP4 | na |  |  | 6227 | 0.536 | -0.0552 | No |
| 30 | ATP2C1 | na |  |  | 6254 | 0.533 | -0.0523 | No |
| 31 | P2RY2 | na |  |  | 6271 | 0.531 | -0.0489 | No |
| 32 | NMI | na |  |  | 6667 | 0.482 | -0.0658 | No |
| 33 | HAS2 | na |  |  | 6692 | 0.480 | -0.0632 | No |
| 34 | GCH1 | na |  |  | 6881 | 0.459 | -0.0694 | No |
| 35 | LYN | na |  |  | 7669 | 0.366 | -0.1077 | No |
| 36 | KIF1B | na |  |  | 8020 | 0.323 | -0.1235 | No |
| 37 | CXCR6 | na |  |  | 8188 | 0.307 | -0.1298 | No |
| 38 | ABI1 | na |  |  | 8474 | 0.275 | -0.1426 | No |
| 39 | ADRM1 | na |  |  | 8507 | 0.271 | -0.1421 | No |
| 40 | TLR1 | na |  |  | 8781 | 0.239 | -0.1545 | No |
| 41 | RHOG | na |  |  | 9030 | 0.212 | -0.1658 | No |
| 42 | TNFSF9 | na |  |  | 9187 | 0.193 | -0.1725 | No |
| 43 | CHST2 | na |  |  | 9739 | 0.132 | -0.2003 | No |
| 44 | INHBA | na |  |  | 10150 | 0.088 | -0.2211 | No |
| 45 | TLR3 | na |  |  | 10160 | 0.087 | -0.2209 | No |
| 46 | ACVR1B | na |  |  | 10312 | 0.067 | -0.2283 | No |
| 47 | NFKB1 | na |  |  | 11078 | -0.014 | -0.2683 | No |
| 48 | HIF1A | na |  |  | 11136 | -0.018 | -0.2711 | No |
| 49 | IFNAR1 | na |  |  | 11221 | -0.030 | -0.2753 | No |
| 50 | RNF144B | na |  |  | 11324 | -0.042 | -0.2803 | No |
| 51 | ACVR2A | na |  |  | 11326 | -0.042 | -0.2800 | No |
| 52 | BTG2 | na |  |  | 11938 | -0.118 | -0.3111 | No |
| 53 | CD40 | na |  |  | 11978 | -0.123 | -0.3122 | No |
| 54 | SCARF1 | na |  |  | 12369 | -0.165 | -0.3313 | No |
| 55 | LIF | na |  |  | 12907 | -0.232 | -0.3576 | No |
| 56 | LAMP3 | na |  |  | 13043 | -0.250 | -0.3627 | No |
| 57 | PTGER2 | na |  |  | 13046 | -0.250 | -0.3608 | No |
| 58 | RGS16 | na |  |  | 13422 | -0.305 | -0.3780 | No |
| 59 | TNFRSF1B | na |  |  | 13603 | -0.331 | -0.3848 | No |
| 60 | SLC11A2 | na |  |  | 13608 | -0.331 | -0.3824 | No |
| 61 | CXCL10 | na |  |  | 13845 | -0.359 | -0.3919 | No |
| 62 | CCR7 | na |  |  | 13929 | -0.368 | -0.3933 | No |
| 63 | IL15RA | na |  |  | 14050 | -0.385 | -0.3965 | No |
| 64 | KLF6 | na |  |  | 14086 | -0.390 | -0.3953 | No |
| 65 | CYBB | na |  |  | 14390 | -0.431 | -0.4077 | No |
| 66 | DCBLD2 | na |  |  | 14704 | -0.481 | -0.4203 | No |
| 67 | SLC31A2 | na |  |  | 14710 | -0.482 | -0.4167 | No |
| 68 | RELA | na |  |  | 14752 | -0.488 | -0.4150 | No |
| 69 | IRF7 | na |  |  | 14815 | -0.500 | -0.4142 | No |
| 70 | PSEN1 | na |  |  | 15031 | -0.515 | -0.4214 | No |
| 71 | TLR2 | na |  |  | 15128 | -0.535 | -0.4222 | No |
| 72 | CCL2 | na |  |  | 15276 | -0.560 | -0.4254 | Yes |
| 73 | BEST1 | na |  |  | 15305 | -0.565 | -0.4224 | Yes |
| 74 | RIPK2 | na |  |  | 15343 | -0.573 | -0.4197 | Yes |
| 75 | MXD1 | na |  |  | 15373 | -0.577 | -0.4166 | Yes |
| 76 | GPR132 | na |  |  | 15495 | -0.597 | -0.4182 | Yes |
| 77 | EBI3 | na |  |  | 15602 | -0.605 | -0.4190 | Yes |
| 78 | GNA15 | na |  |  | 15638 | -0.613 | -0.4159 | Yes |
| 79 | PLAUR | na |  |  | 15641 | -0.614 | -0.4111 | Yes |
| 80 | IL15 | na |  |  | 15840 | -0.654 | -0.4163 | Yes |
| 81 | CDKN1A | na |  |  | 15950 | -0.678 | -0.4166 | Yes |
| 82 | IL1A | na |  |  | 16094 | -0.702 | -0.4185 | Yes |
| 83 | CCRL2 | na |  |  | 16115 | -0.706 | -0.4139 | Yes |
| 84 | EMP3 | na |  |  | 16130 | -0.712 | -0.4089 | Yes |
| 85 | P2RX4 | na |  |  | 16172 | -0.726 | -0.4053 | Yes |
| 86 | CD14 | na |  |  | 16595 | -0.833 | -0.4208 | Yes |
| 87 | GP1BA | na |  |  | 16758 | -0.872 | -0.4223 | Yes |
| 88 | ADM | na |  |  | 16860 | -0.908 | -0.4204 | Yes |
| 89 | LY6E | na |  |  | 16861 | -0.908 | -0.4131 | Yes |
| 90 | EREG | na |  |  | 16952 | -0.936 | -0.4104 | Yes |
| 91 | SEMA4D | na |  |  | 16971 | -0.942 | -0.4038 | Yes |
| 92 | IFITM1 | na |  |  | 17072 | -0.969 | -0.4013 | Yes |
| 93 | ICOSLG | na |  |  | 17131 | -0.989 | -0.3964 | Yes |
| 94 | GABBR1 | na |  |  | 17370 | -1.066 | -0.4004 | Yes |
| 95 | NFKBIA | na |  |  | 17437 | -1.096 | -0.3951 | Yes |
| 96 | TIMP1 | na |  |  | 17600 | -1.148 | -0.3945 | Yes |
| 97 | NDP | na |  |  | 17628 | -1.161 | -0.3866 | Yes |
| 98 | TAPBP | na |  |  | 17642 | -1.164 | -0.3780 | Yes |
| 99 | CSF3 | na |  |  | 17647 | -1.166 | -0.3689 | Yes |
| 100 | SCN1B | na |  |  | 17660 | -1.170 | -0.3602 | Yes |
| 101 | CD55 | na |  |  | 17692 | -1.182 | -0.3524 | Yes |
| 102 | LDLR | na |  |  | 17723 | -1.192 | -0.3444 | Yes |
| 103 | PTPRE | na |  |  | 17804 | -1.225 | -0.3389 | Yes |
| 104 | CD82 | na |  |  | 17854 | -1.256 | -0.3314 | Yes |
| 105 | ICAM1 | na |  |  | 17954 | -1.306 | -0.3262 | Yes |
| 106 | ITGA5 | na |  |  | 18016 | -1.340 | -0.3187 | Yes |
| 107 | IRF1 | na |  |  | 18023 | -1.342 | -0.3083 | Yes |
| 108 | IL4R | na |  |  | 18107 | -1.380 | -0.3016 | Yes |
| 109 | IL1B | na |  |  | 18217 | -1.433 | -0.2959 | Yes |
| 110 | IRAK2 | na |  |  | 18256 | -1.460 | -0.2862 | Yes |
| 111 | FPR1 | na |  |  | 18271 | -1.474 | -0.2752 | Yes |
| 112 | IL1R1 | na |  |  | 18283 | -1.480 | -0.2640 | Yes |
| 113 | PDE4B | na |  |  | 18287 | -1.482 | -0.2523 | Yes |
| 114 | IFNGR2 | na |  |  | 18421 | -1.594 | -0.2466 | Yes |
| 115 | NAMPT | na |  |  | 18422 | -1.594 | -0.2338 | Yes |
| 116 | TNFSF10 | na |  |  | 18488 | -1.633 | -0.2242 | Yes |
| 117 | CCL20 | na |  |  | 18550 | -1.693 | -0.2139 | Yes |
| 118 | OSMR | na |  |  | 18646 | -1.810 | -0.2044 | Yes |
| 119 | CX3CL1 | na |  |  | 18661 | -1.834 | -0.1905 | Yes |
| 120 | AHR | na |  |  | 18744 | -1.954 | -0.1792 | Yes |
| 121 | IL6 | na |  |  | 18776 | -2.020 | -0.1648 | Yes |
| 122 | TPBG | na |  |  | 18842 | -2.163 | -0.1509 | Yes |
| 123 | BST2 | na |  |  | 18856 | -2.210 | -0.1339 | Yes |
| 124 | CMKLR1 | na |  |  | 18878 | -2.270 | -0.1169 | Yes |
| 125 | TNFSF15 | na |  |  | 18899 | -2.318 | -0.0995 | Yes |
| 126 | ITGB3 | na |  |  | 18931 | -2.380 | -0.0821 | Yes |
| 127 | ABCA1 | na |  |  | 18984 | -2.587 | -0.0642 | Yes |
| 128 | TNFRSF9 | na |  |  | 19048 | -2.893 | -0.0444 | Yes |
| 129 | ITGB8 | na |  |  | 19107 | -3.224 | -0.0217 | Yes |
| 130 | CXCL11 | na |  |  | 19118 | -3.311 | 0.0042 | Yes |
Table: GSEA details [plain text format]

  

Fig 2: HALLMARK\_INFLAMMATORY\_RESPONSE      
 Blue-Pink O' Gram in the Space of the Analyzed GeneSet

  

Fig 3: HALLMARK\_INFLAMMATORY\_RESPONSE: Random ES distribution      
 Gene set null distribution of ES for **HALLMARK\_INFLAMMATORY\_RESPONSE**

  
