# Supplementary material for: Heparin-based hydrogel scaffolding alters the transcriptomic profile and increases the chemoresistance of MDA-MB-231 triple-negative breast cancer cells
Source: Biomater Sci. 2020 Feb 13;8(10):2786–96. doi: 10.1039/c9bm01481k (PMC7497406; doi:10.1039/c9bm01481k)
Supplement: Supplementary file 2 [file BM-008-C9BM01481K-s002.zip › Supplementary File 4/EGFvControl/Pathways/my_analysis.Gsea.1545200981068/HALLMARK_GLYCOLYSIS.html]

Details for gene set HALLMARK\_GLYCOLYSIS[GSEA]

|  || Dataset | expr.class.cls#EGF\_versus\_CONTROL.class.cls#EGF\_versus\_CONTROL\_repos |
| Phenotype | class.cls#EGF\_versus\_CONTROL\_repos |
| Upregulated in class | CONTROL |
| GeneSet | HALLMARK\_GLYCOLYSIS |
| Enrichment Score (ES) | -0.32389128 |
| Normalized Enrichment Score (NES) | -1.5497401 |
| Nominal p-value | 0.0 |
| FDR q-value | 0.014346696 |
| FWER p-Value | 0.154 |
Table: GSEA Results Summary

  

Fig 1: Enrichment plot: HALLMARK\_GLYCOLYSIS      
 Profile of the Running ES Score & Positions of GeneSet Members on the Rank Ordered List

  

| PROBE | DESCRIPTION (from dataset) | GENE SYMBOL | GENE\_TITLE | RANK IN GENE LIST | RANK METRIC SCORE | RUNNING ES | CORE ENRICHMENT || 1 | AURKA | na |  |  | 163 | 2.181 | 0.0056 | No |
| 2 | GOT1 | na |  |  | 200 | 2.096 | 0.0174 | No |
| 3 | PRPS1 | na |  |  | 267 | 2.008 | 0.0270 | No |
| 4 | ABCB6 | na |  |  | 292 | 1.964 | 0.0385 | No |
| 5 | PGM2 | na |  |  | 395 | 1.849 | 0.0452 | No |
| 6 | POLR3K | na |  |  | 466 | 1.789 | 0.0531 | No |
| 7 | ADORA2B | na |  |  | 492 | 1.775 | 0.0634 | No |
| 8 | FKBP4 | na |  |  | 865 | 1.565 | 0.0540 | No |
| 9 | NANP | na |  |  | 953 | 1.528 | 0.0594 | No |
| 10 | KIF2A | na |  |  | 1070 | 1.489 | 0.0630 | No |
| 11 | CLN6 | na |  |  | 1082 | 1.485 | 0.0720 | No |
| 12 | EGFR | na |  |  | 1241 | 1.429 | 0.0730 | No |
| 13 | RARS | na |  |  | 1261 | 1.424 | 0.0813 | No |
| 14 | MDH1 | na |  |  | 1318 | 1.406 | 0.0875 | No |
| 15 | KIF20A | na |  |  | 1348 | 1.393 | 0.0951 | No |
| 16 | HMMR | na |  |  | 1351 | 1.393 | 0.1040 | No |
| 17 | ME1 | na |  |  | 1379 | 1.385 | 0.1116 | No |
| 18 | GOT2 | na |  |  | 1607 | 1.316 | 0.1082 | No |
| 19 | PYGL | na |  |  | 1905 | 1.245 | 0.1007 | No |
| 20 | PAXIP1 | na |  |  | 2191 | 1.180 | 0.0934 | No |
| 21 | TXN | na |  |  | 2234 | 1.172 | 0.0989 | No |
| 22 | SLC25A10 | na |  |  | 2509 | 1.109 | 0.0917 | No |
| 23 | DEPDC1 | na |  |  | 2541 | 1.104 | 0.0972 | No |
| 24 | PSMC4 | na |  |  | 2550 | 1.101 | 0.1040 | No |
| 25 | PKP2 | na |  |  | 2914 | 1.041 | 0.0917 | No |
| 26 | CDK1 | na |  |  | 2921 | 1.040 | 0.0981 | No |
| 27 | ECD | na |  |  | 3053 | 1.015 | 0.0978 | No |
| 28 | COG2 | na |  |  | 3364 | 0.959 | 0.0878 | No |
| 29 | PPP2CB | na |  |  | 3484 | 0.939 | 0.0877 | No |
| 30 | ME2 | na |  |  | 3508 | 0.936 | 0.0925 | No |
| 31 | MET | na |  |  | 3522 | 0.933 | 0.0979 | No |
| 32 | NASP | na |  |  | 3598 | 0.921 | 0.1000 | No |
| 33 | GMPPB | na |  |  | 3627 | 0.917 | 0.1045 | No |
| 34 | DLD | na |  |  | 3868 | 0.873 | 0.0975 | No |
| 35 | CD44 | na |  |  | 3954 | 0.860 | 0.0987 | No |
| 36 | HAX1 | na |  |  | 4113 | 0.835 | 0.0958 | No |
| 37 | ALG1 | na |  |  | 4297 | 0.809 | 0.0914 | No |
| 38 | CXCR4 | na |  |  | 4387 | 0.794 | 0.0919 | No |
| 39 | CENPA | na |  |  | 4451 | 0.785 | 0.0937 | No |
| 40 | B4GALT4 | na |  |  | 4452 | 0.785 | 0.0988 | No |
| 41 | RPE | na |  |  | 4539 | 0.772 | 0.0993 | No |
| 42 | STMN1 | na |  |  | 4739 | 0.740 | 0.0937 | No |
| 43 | ALDH7A1 | na |  |  | 4850 | 0.722 | 0.0926 | No |
| 44 | NSDHL | na |  |  | 4974 | 0.712 | 0.0908 | No |
| 45 | EXT1 | na |  |  | 5006 | 0.707 | 0.0938 | No |
| 46 | ARPP19 | na |  |  | 5182 | 0.681 | 0.0890 | No |
| 47 | STC2 | na |  |  | 5348 | 0.658 | 0.0846 | No |
| 48 | NDST3 | na |  |  | 5577 | 0.628 | 0.0767 | No |
| 49 | ALDH9A1 | na |  |  | 5781 | 0.599 | 0.0699 | No |
| 50 | GNPDA1 | na |  |  | 6032 | 0.567 | 0.0605 | No |
| 51 | HOMER1 | na |  |  | 6039 | 0.565 | 0.0638 | No |
| 52 | GFPT1 | na |  |  | 6073 | 0.561 | 0.0658 | No |
| 53 | BPNT1 | na |  |  | 6110 | 0.553 | 0.0675 | No |
| 54 | SLC25A13 | na |  |  | 6198 | 0.542 | 0.0664 | No |
| 55 | PMM2 | na |  |  | 6238 | 0.535 | 0.0679 | No |
| 56 | HS2ST1 | na |  |  | 6340 | 0.522 | 0.0660 | No |
| 57 | SLC35A3 | na |  |  | 6538 | 0.497 | 0.0588 | No |
| 58 | TSTA3 | na |  |  | 6733 | 0.476 | 0.0517 | No |
| 59 | GCLC | na |  |  | 6772 | 0.472 | 0.0528 | No |
| 60 | ANGPTL4 | na |  |  | 6796 | 0.468 | 0.0546 | No |
| 61 | PHKA2 | na |  |  | 6905 | 0.455 | 0.0519 | No |
| 62 | SLC37A4 | na |  |  | 6924 | 0.453 | 0.0539 | No |
| 63 | AGL | na |  |  | 7131 | 0.430 | 0.0459 | No |
| 64 | NDUFV3 | na |  |  | 7737 | 0.358 | 0.0164 | No |
| 65 | LHX9 | na |  |  | 7889 | 0.338 | 0.0107 | No |
| 66 | SOD1 | na |  |  | 7945 | 0.332 | 0.0100 | No |
| 67 | SDC1 | na |  |  | 7964 | 0.330 | 0.0112 | No |
| 68 | CTH | na |  |  | 7970 | 0.329 | 0.0130 | No |
| 69 | TPST1 | na |  |  | 8204 | 0.304 | 0.0028 | No |
| 70 | IDH1 | na |  |  | 8504 | 0.271 | -0.0112 | No |
| 71 | COPB2 | na |  |  | 8520 | 0.268 | -0.0102 | No |
| 72 | VCAN | na |  |  | 8574 | 0.265 | -0.0113 | No |
| 73 | SDHC | na |  |  | 8704 | 0.246 | -0.0164 | No |
| 74 | SDC2 | na |  |  | 8869 | 0.228 | -0.0236 | No |
| 75 | IRS2 | na |  |  | 9101 | 0.201 | -0.0344 | No |
| 76 | HDLBP | na |  |  | 9190 | 0.193 | -0.0378 | No |
| 77 | TALDO1 | na |  |  | 9239 | 0.189 | -0.0391 | No |
| 78 | CHST2 | na |  |  | 9739 | 0.132 | -0.0644 | No |
| 79 | CLDN9 | na |  |  | 9823 | 0.125 | -0.0680 | No |
| 80 | CITED2 | na |  |  | 9844 | 0.121 | -0.0683 | No |
| 81 | UGP2 | na |  |  | 9929 | 0.111 | -0.0719 | No |
| 82 | QSOX1 | na |  |  | 10103 | 0.095 | -0.0804 | No |
| 83 | CHST12 | na |  |  | 10207 | 0.081 | -0.0853 | No |
| 84 | GPC1 | na |  |  | 10254 | 0.075 | -0.0872 | No |
| 85 | MDH2 | na |  |  | 10374 | 0.061 | -0.0931 | No |
| 86 | LCT | na |  |  | 10508 | 0.053 | -0.0997 | No |
| 87 | NT5E | na |  |  | 10569 | 0.043 | -0.1026 | No |
| 88 | B4GALT7 | na |  |  | 11026 | -0.006 | -0.1265 | No |
| 89 | GALK1 | na |  |  | 11038 | -0.007 | -0.1271 | No |
| 90 | PFKFB1 | na |  |  | 11369 | -0.047 | -0.1441 | No |
| 91 | GLCE | na |  |  | 11811 | -0.101 | -0.1666 | No |
| 92 | EXT2 | na |  |  | 11911 | -0.115 | -0.1711 | No |
| 93 | GPR87 | na |  |  | 12084 | -0.136 | -0.1792 | No |
| 94 | B3GALT6 | na |  |  | 12175 | -0.140 | -0.1830 | No |
| 95 | TGFA | na |  |  | 12260 | -0.151 | -0.1865 | No |
| 96 | GUSB | na |  |  | 12358 | -0.164 | -0.1905 | No |
| 97 | B4GALT2 | na |  |  | 12690 | -0.212 | -0.2065 | No |
| 98 | SAP30 | na |  |  | 12691 | -0.212 | -0.2052 | No |
| 99 | RBCK1 | na |  |  | 12706 | -0.214 | -0.2045 | No |
| 100 | CYB5A | na |  |  | 12910 | -0.233 | -0.2136 | No |
| 101 | GALE | na |  |  | 12953 | -0.240 | -0.2143 | No |
| 102 | G6PD | na |  |  | 13071 | -0.252 | -0.2188 | No |
| 103 | RRAGD | na |  |  | 13146 | -0.265 | -0.2210 | No |
| 104 | GMPPA | na |  |  | 13150 | -0.266 | -0.2194 | No |
| 105 | XYLT2 | na |  |  | 13359 | -0.297 | -0.2284 | No |
| 106 | IL13RA1 | na |  |  | 13405 | -0.303 | -0.2288 | No |
| 107 | MPI | na |  |  | 13561 | -0.325 | -0.2348 | No |
| 108 | GALK2 | na |  |  | 13811 | -0.351 | -0.2456 | No |
| 109 | CASP6 | na |  |  | 13966 | -0.372 | -0.2513 | No |
| 110 | PGLS | na |  |  | 13995 | -0.377 | -0.2503 | No |
| 111 | ARTN | na |  |  | 14174 | -0.401 | -0.2571 | No |
| 112 | CACNA1H | na |  |  | 14194 | -0.405 | -0.2554 | No |
| 113 | CHPF2 | na |  |  | 14242 | -0.412 | -0.2552 | No |
| 114 | MED24 | na |  |  | 14377 | -0.429 | -0.2595 | No |
| 115 | HK2 | na |  |  | 14409 | -0.434 | -0.2583 | No |
| 116 | PGAM1 | na |  |  | 14534 | -0.450 | -0.2619 | No |
| 117 | GNE | na |  |  | 14635 | -0.468 | -0.2641 | No |
| 118 | AKR1A1 | na |  |  | 14707 | -0.481 | -0.2647 | No |
| 119 | ANG | na |  |  | 15384 | -0.579 | -0.2964 | No |
| 120 | PFKP | na |  |  | 15615 | -0.609 | -0.3046 | No |
| 121 | SOX9 | na |  |  | 15836 | -0.654 | -0.3119 | No |
| 122 | B3GAT3 | na |  |  | 15855 | -0.658 | -0.3085 | No |
| 123 | EFNA3 | na |  |  | 16136 | -0.713 | -0.3186 | No |
| 124 | HSPA5 | na |  |  | 16140 | -0.715 | -0.3141 | No |
| 125 | MERTK | na |  |  | 16180 | -0.727 | -0.3114 | No |
| 126 | IDUA | na |  |  | 16415 | -0.787 | -0.3186 | Yes |
| 127 | ZNF292 | na |  |  | 16428 | -0.791 | -0.3141 | Yes |
| 128 | ALDOA | na |  |  | 16455 | -0.799 | -0.3103 | Yes |
| 129 | TPI1 | na |  |  | 16603 | -0.835 | -0.3125 | Yes |
| 130 | COL5A1 | na |  |  | 16811 | -0.890 | -0.3176 | Yes |
| 131 | GYS1 | na |  |  | 16931 | -0.931 | -0.3178 | Yes |
| 132 | KDELR3 | na |  |  | 17046 | -0.961 | -0.3176 | Yes |
| 133 | CHPF | na |  |  | 17062 | -0.966 | -0.3121 | Yes |
| 134 | AK3 | na |  |  | 17069 | -0.968 | -0.3061 | Yes |
| 135 | FUT8 | na |  |  | 17111 | -0.981 | -0.3019 | Yes |
| 136 | FAM162A | na |  |  | 17146 | -0.993 | -0.2972 | Yes |
| 137 | BIK | na |  |  | 17323 | -1.050 | -0.2996 | Yes |
| 138 | PC | na |  |  | 17387 | -1.072 | -0.2959 | Yes |
| 139 | LHPP | na |  |  | 17402 | -1.079 | -0.2896 | Yes |
| 140 | SLC16A3 | na |  |  | 17416 | -1.087 | -0.2833 | Yes |
| 141 | SRD5A3 | na |  |  | 17520 | -1.129 | -0.2813 | Yes |
| 142 | B4GALT1 | na |  |  | 17619 | -1.157 | -0.2789 | Yes |
| 143 | PLOD1 | na |  |  | 17650 | -1.167 | -0.2729 | Yes |
| 144 | PLOD2 | na |  |  | 17729 | -1.194 | -0.2693 | Yes |
| 145 | B3GNT3 | na |  |  | 17746 | -1.200 | -0.2623 | Yes |
| 146 | DDIT4 | na |  |  | 17752 | -1.202 | -0.2547 | Yes |
| 147 | SDC3 | na |  |  | 17812 | -1.229 | -0.2498 | Yes |
| 148 | TGFBI | na |  |  | 17865 | -1.259 | -0.2444 | Yes |
| 149 | ANKZF1 | na |  |  | 17879 | -1.263 | -0.2368 | Yes |
| 150 | PYGB | na |  |  | 17907 | -1.278 | -0.2299 | Yes |
| 151 | P4HA2 | na |  |  | 17972 | -1.316 | -0.2247 | Yes |
| 152 | P4HA1 | na |  |  | 18001 | -1.330 | -0.2176 | Yes |
| 153 | ELF3 | na |  |  | 18029 | -1.345 | -0.2102 | Yes |
| 154 | AK4 | na |  |  | 18050 | -1.351 | -0.2025 | Yes |
| 155 | GLRX | na |  |  | 18169 | -1.407 | -0.1995 | Yes |
| 156 | CAPN5 | na |  |  | 18247 | -1.455 | -0.1941 | Yes |
| 157 | ENO2 | na |  |  | 18286 | -1.481 | -0.1865 | Yes |
| 158 | PDK3 | na |  |  | 18298 | -1.493 | -0.1773 | Yes |
| 159 | IGFBP3 | na |  |  | 18475 | -1.624 | -0.1760 | Yes |
| 160 | ISG20 | na |  |  | 18505 | -1.647 | -0.1668 | Yes |
| 161 | PAM | na |  |  | 18737 | -1.945 | -0.1663 | Yes |
| 162 | PGK1 | na |  |  | 18818 | -2.107 | -0.1568 | Yes |
| 163 | TPBG | na |  |  | 18842 | -2.163 | -0.1439 | Yes |
| 164 | NOL3 | na |  |  | 18930 | -2.376 | -0.1330 | Yes |
| 165 | VLDLR | na |  |  | 18950 | -2.433 | -0.1182 | Yes |
| 166 | STC1 | na |  |  | 18997 | -2.647 | -0.1034 | Yes |
| 167 | MXI1 | na |  |  | 19008 | -2.689 | -0.0864 | Yes |
| 168 | SPAG4 | na |  |  | 19098 | -3.128 | -0.0707 | Yes |
| 169 | DSC2 | na |  |  | 19106 | -3.207 | -0.0502 | Yes |
| 170 | PPFIA4 | na |  |  | 19170 | -4.119 | -0.0267 | Yes |
| 171 | EGLN3 | na |  |  | 19178 | -4.329 | 0.0011 | Yes |
Table: GSEA details [plain text format]

  

Fig 2: HALLMARK\_GLYCOLYSIS      
 Blue-Pink O' Gram in the Space of the Analyzed GeneSet

  

Fig 3: HALLMARK\_GLYCOLYSIS: Random ES distribution      
 Gene set null distribution of ES for **HALLMARK\_GLYCOLYSIS**

  
