# Supplementary material for: Heparin-based hydrogel scaffolding alters the transcriptomic profile and increases the chemoresistance of MDA-MB-231 triple-negative breast cancer cells
Source: Biomater Sci. 2020 Feb 13;8(10):2786–96. doi: 10.1039/c9bm01481k (PMC7497406; doi:10.1039/c9bm01481k)
Supplement: Supplementary file 2 [file BM-008-C9BM01481K-s002.zip › Supplementary File 4/EGFvControl/Pathways/my_analysis.Gsea.1545200981068/HALLMARK_TNFA_SIGNALING_VIA_NFKB.html]

Details for gene set HALLMARK\_TNFA\_SIGNALING\_VIA\_NFKB[GSEA]

|  || Dataset | expr.class.cls#EGF\_versus\_CONTROL.class.cls#EGF\_versus\_CONTROL\_repos |
| Phenotype | class.cls#EGF\_versus\_CONTROL\_repos |
| Upregulated in class | CONTROL |
| GeneSet | HALLMARK\_TNFA\_SIGNALING\_VIA\_NFKB |
| Enrichment Score (ES) | -0.5156901 |
| Normalized Enrichment Score (NES) | -2.4871068 |
| Nominal p-value | 0.0 |
| FDR q-value | 0.0 |
| FWER p-Value | 0.0 |
Table: GSEA Results Summary

  

Fig 1: Enrichment plot: HALLMARK\_TNFA\_SIGNALING\_VIA\_NFKB      
 Profile of the Running ES Score & Positions of GeneSet Members on the Rank Ordered List

  

| PROBE | DESCRIPTION (from dataset) | GENE SYMBOL | GENE\_TITLE | RANK IN GENE LIST | RANK METRIC SCORE | RUNNING ES | CORE ENRICHMENT || 1 | FJX1 | na |  |  | 47 | 2.688 | 0.0123 | No |
| 2 | EDN1 | na |  |  | 77 | 2.466 | 0.0244 | No |
| 3 | IL18 | na |  |  | 152 | 2.202 | 0.0326 | No |
| 4 | MAP2K3 | na |  |  | 282 | 1.978 | 0.0367 | No |
| 5 | PLAU | na |  |  | 387 | 1.854 | 0.0415 | No |
| 6 | F3 | na |  |  | 545 | 1.742 | 0.0428 | No |
| 7 | SPHK1 | na |  |  | 643 | 1.674 | 0.0469 | No |
| 8 | DNAJB4 | na |  |  | 809 | 1.587 | 0.0470 | No |
| 9 | GADD45B | na |  |  | 1283 | 1.417 | 0.0299 | No |
| 10 | YRDC | na |  |  | 1520 | 1.340 | 0.0249 | No |
| 11 | IL7R | na |  |  | 1696 | 1.295 | 0.0228 | No |
| 12 | BIRC2 | na |  |  | 1711 | 1.291 | 0.0292 | No |
| 13 | ETS2 | na |  |  | 1829 | 1.261 | 0.0300 | No |
| 14 | SERPINB2 | na |  |  | 2053 | 1.207 | 0.0249 | No |
| 15 | PTGER4 | na |  |  | 2110 | 1.194 | 0.0285 | No |
| 16 | PMEPA1 | na |  |  | 2174 | 1.184 | 0.0317 | No |
| 17 | RCAN1 | na |  |  | 2291 | 1.159 | 0.0320 | No |
| 18 | ATP2B1 | na |  |  | 2361 | 1.142 | 0.0347 | No |
| 19 | CCND1 | na |  |  | 2400 | 1.133 | 0.0389 | No |
| 20 | HBEGF | na |  |  | 3593 | 0.921 | -0.0187 | No |
| 21 | MYC | na |  |  | 3907 | 0.866 | -0.0304 | No |
| 22 | CD44 | na |  |  | 3954 | 0.860 | -0.0281 | No |
| 23 | BTG3 | na |  |  | 3985 | 0.853 | -0.0249 | No |
| 24 | PLK2 | na |  |  | 4344 | 0.801 | -0.0394 | No |
| 25 | IER5 | na |  |  | 4968 | 0.712 | -0.0682 | No |
| 26 | FOSB | na |  |  | 5209 | 0.678 | -0.0771 | No |
| 27 | NFAT5 | na |  |  | 5539 | 0.633 | -0.0909 | No |
| 28 | FOSL1 | na |  |  | 5713 | 0.607 | -0.0966 | No |
| 29 | GADD45A | na |  |  | 6102 | 0.555 | -0.1140 | No |
| 30 | GCH1 | na |  |  | 6881 | 0.459 | -0.1524 | No |
| 31 | EHD1 | na |  |  | 6990 | 0.448 | -0.1556 | No |
| 32 | TNFAIP8 | na |  |  | 7099 | 0.433 | -0.1589 | No |
| 33 | ATF3 | na |  |  | 7410 | 0.395 | -0.1730 | No |
| 34 | PDLIM5 | na |  |  | 7457 | 0.391 | -0.1733 | No |
| 35 | FUT4 | na |  |  | 7683 | 0.365 | -0.1831 | No |
| 36 | CD83 | na |  |  | 7756 | 0.356 | -0.1849 | No |
| 37 | IFIT2 | na |  |  | 8545 | 0.266 | -0.2249 | No |
| 38 | TIPARP | na |  |  | 8676 | 0.250 | -0.2303 | No |
| 39 | PPP1R15A | na |  |  | 8701 | 0.246 | -0.2302 | No |
| 40 | TUBB2A | na |  |  | 8831 | 0.234 | -0.2357 | No |
| 41 | TNIP2 | na |  |  | 9075 | 0.205 | -0.2474 | No |
| 42 | IRS2 | na |  |  | 9101 | 0.201 | -0.2476 | No |
| 43 | TNFSF9 | na |  |  | 9187 | 0.193 | -0.2510 | No |
| 44 | INHBA | na |  |  | 10150 | 0.088 | -0.3011 | No |
| 45 | NR4A3 | na |  |  | 10179 | 0.084 | -0.3021 | No |
| 46 | DENND5A | na |  |  | 10268 | 0.073 | -0.3063 | No |
| 47 | ZBTB10 | na |  |  | 11051 | -0.010 | -0.3474 | No |
| 48 | NFKB1 | na |  |  | 11078 | -0.014 | -0.3486 | No |
| 49 | EIF1 | na |  |  | 11186 | -0.025 | -0.3541 | No |
| 50 | HES1 | na |  |  | 11615 | -0.072 | -0.3762 | No |
| 51 | IL6ST | na |  |  | 11649 | -0.077 | -0.3775 | No |
| 52 | BTG2 | na |  |  | 11938 | -0.118 | -0.3920 | No |
| 53 | ID2 | na |  |  | 11940 | -0.118 | -0.3914 | No |
| 54 | CLCF1 | na |  |  | 12277 | -0.152 | -0.4083 | No |
| 55 | NFE2L2 | na |  |  | 12430 | -0.175 | -0.4153 | No |
| 56 | G0S2 | na |  |  | 12437 | -0.176 | -0.4146 | No |
| 57 | MCL1 | na |  |  | 12451 | -0.179 | -0.4143 | No |
| 58 | JUN | na |  |  | 12605 | -0.199 | -0.4213 | No |
| 59 | LIF | na |  |  | 12907 | -0.232 | -0.4358 | No |
| 60 | F2RL1 | na |  |  | 13357 | -0.296 | -0.4578 | No |
| 61 | REL | na |  |  | 13430 | -0.305 | -0.4599 | No |
| 62 | CCNL1 | na |  |  | 13683 | -0.343 | -0.4713 | No |
| 63 | TRIP10 | na |  |  | 13701 | -0.345 | -0.4703 | No |
| 64 | CXCL10 | na |  |  | 13845 | -0.359 | -0.4758 | No |
| 65 | PTX3 | na |  |  | 13978 | -0.374 | -0.4807 | No |
| 66 | IL15RA | na |  |  | 14050 | -0.385 | -0.4823 | No |
| 67 | LITAF | na |  |  | 14053 | -0.386 | -0.4803 | No |
| 68 | KLF6 | na |  |  | 14086 | -0.390 | -0.4798 | No |
| 69 | TANK | na |  |  | 14216 | -0.408 | -0.4844 | No |
| 70 | RNF19B | na |  |  | 14473 | -0.441 | -0.4954 | No |
| 71 | SGK1 | na |  |  | 14627 | -0.466 | -0.5009 | No |
| 72 | DUSP1 | na |  |  | 14724 | -0.484 | -0.5033 | No |
| 73 | RELA | na |  |  | 14752 | -0.488 | -0.5020 | No |
| 74 | GEM | na |  |  | 14920 | -0.503 | -0.5080 | No |
| 75 | KLF2 | na |  |  | 14929 | -0.504 | -0.5056 | No |
| 76 | SLC2A6 | na |  |  | 15085 | -0.526 | -0.5109 | No |
| 77 | TLR2 | na |  |  | 15128 | -0.535 | -0.5102 | No |
| 78 | EGR2 | na |  |  | 15131 | -0.536 | -0.5073 | No |
| 79 | LAMB3 | na |  |  | 15267 | -0.557 | -0.5113 | No |
| 80 | CCL2 | na |  |  | 15276 | -0.560 | -0.5087 | No |
| 81 | RIPK2 | na |  |  | 15343 | -0.573 | -0.5090 | No |
| 82 | MXD1 | na |  |  | 15373 | -0.577 | -0.5073 | No |
| 83 | NR4A1 | na |  |  | 15488 | -0.595 | -0.5101 | No |
| 84 | JAG1 | na |  |  | 15596 | -0.604 | -0.5124 | Yes |
| 85 | SIK1 | na |  |  | 15609 | -0.607 | -0.5097 | Yes |
| 86 | PLAUR | na |  |  | 15641 | -0.614 | -0.5079 | Yes |
| 87 | PER1 | na |  |  | 15649 | -0.615 | -0.5049 | Yes |
| 88 | PHLDA1 | na |  |  | 15776 | -0.644 | -0.5080 | Yes |
| 89 | IFIH1 | na |  |  | 15792 | -0.647 | -0.5052 | Yes |
| 90 | SMAD3 | na |  |  | 15810 | -0.650 | -0.5025 | Yes |
| 91 | TNF | na |  |  | 15826 | -0.653 | -0.4997 | Yes |
| 92 | CEBPB | na |  |  | 15897 | -0.665 | -0.4997 | Yes |
| 93 | CDKN1A | na |  |  | 15950 | -0.678 | -0.4987 | Yes |
| 94 | SOCS3 | na |  |  | 16002 | -0.686 | -0.4976 | Yes |
| 95 | IL1A | na |  |  | 16094 | -0.702 | -0.4985 | Yes |
| 96 | CCRL2 | na |  |  | 16115 | -0.706 | -0.4957 | Yes |
| 97 | FOSL2 | na |  |  | 16125 | -0.710 | -0.4923 | Yes |
| 98 | NFKBIE | na |  |  | 16129 | -0.711 | -0.4885 | Yes |
| 99 | IER2 | na |  |  | 16166 | -0.724 | -0.4864 | Yes |
| 100 | DDX58 | na |  |  | 16198 | -0.731 | -0.4840 | Yes |
| 101 | PHLDA2 | na |  |  | 16201 | -0.731 | -0.4801 | Yes |
| 102 | KLF10 | na |  |  | 16235 | -0.741 | -0.4778 | Yes |
| 103 | DRAM1 | na |  |  | 16350 | -0.769 | -0.4795 | Yes |
| 104 | PANX1 | na |  |  | 16359 | -0.771 | -0.4757 | Yes |
| 105 | RHOB | na |  |  | 16399 | -0.781 | -0.4735 | Yes |
| 106 | KLF4 | na |  |  | 16438 | -0.793 | -0.4711 | Yes |
| 107 | NINJ1 | na |  |  | 16489 | -0.808 | -0.4693 | Yes |
| 108 | TNC | na |  |  | 16616 | -0.837 | -0.4713 | Yes |
| 109 | SNN | na |  |  | 16639 | -0.843 | -0.4678 | Yes |
| 110 | SQSTM1 | na |  |  | 16663 | -0.848 | -0.4643 | Yes |
| 111 | MAFF | na |  |  | 16697 | -0.856 | -0.4614 | Yes |
| 112 | EGR3 | na |  |  | 16779 | -0.878 | -0.4608 | Yes |
| 113 | SERPINB8 | na |  |  | 16830 | -0.899 | -0.4585 | Yes |
| 114 | MAP3K8 | na |  |  | 16847 | -0.904 | -0.4543 | Yes |
| 115 | NFKB2 | na |  |  | 16862 | -0.909 | -0.4501 | Yes |
| 116 | DUSP5 | na |  |  | 16942 | -0.934 | -0.4491 | Yes |
| 117 | ICOSLG | na |  |  | 17131 | -0.989 | -0.4535 | Yes |
| 118 | CEBPD | na |  |  | 17150 | -0.995 | -0.4490 | Yes |
| 119 | SPSB1 | na |  |  | 17236 | -1.017 | -0.4479 | Yes |
| 120 | CXCL1 | na |  |  | 17379 | -1.069 | -0.4494 | Yes |
| 121 | TAP1 | na |  |  | 17413 | -1.084 | -0.4452 | Yes |
| 122 | NFKBIA | na |  |  | 17437 | -1.096 | -0.4404 | Yes |
| 123 | CXCL3 | na |  |  | 17464 | -1.110 | -0.4356 | Yes |
| 124 | TNFAIP3 | na |  |  | 17494 | -1.121 | -0.4310 | Yes |
| 125 | JUNB | na |  |  | 17499 | -1.123 | -0.4250 | Yes |
| 126 | CXCL2 | na |  |  | 17501 | -1.124 | -0.4189 | Yes |
| 127 | RELB | na |  |  | 17504 | -1.124 | -0.4128 | Yes |
| 128 | TNIP1 | na |  |  | 17556 | -1.134 | -0.4092 | Yes |
| 129 | CSF2 | na |  |  | 17557 | -1.135 | -0.4030 | Yes |
| 130 | STAT5A | na |  |  | 17598 | -1.148 | -0.3988 | Yes |
| 131 | B4GALT1 | na |  |  | 17619 | -1.157 | -0.3935 | Yes |
| 132 | FOS | na |  |  | 17698 | -1.185 | -0.3910 | Yes |
| 133 | SDC4 | na |  |  | 17717 | -1.190 | -0.3854 | Yes |
| 134 | LDLR | na |  |  | 17723 | -1.192 | -0.3791 | Yes |
| 135 | EFNA1 | na |  |  | 17738 | -1.198 | -0.3733 | Yes |
| 136 | PTPRE | na |  |  | 17804 | -1.225 | -0.3699 | Yes |
| 137 | MARCKS | na |  |  | 17808 | -1.228 | -0.3633 | Yes |
| 138 | B4GALT5 | na |  |  | 17866 | -1.259 | -0.3594 | Yes |
| 139 | ZC3H12A | na |  |  | 17934 | -1.292 | -0.3558 | Yes |
| 140 | ICAM1 | na |  |  | 17954 | -1.306 | -0.3496 | Yes |
| 141 | IRF1 | na |  |  | 18023 | -1.342 | -0.3458 | Yes |
| 142 | PFKFB3 | na |  |  | 18027 | -1.345 | -0.3386 | Yes |
| 143 | IL23A | na |  |  | 18033 | -1.345 | -0.3314 | Yes |
| 144 | EGR1 | na |  |  | 18053 | -1.352 | -0.3250 | Yes |
| 145 | BCL3 | na |  |  | 18068 | -1.364 | -0.3182 | Yes |
| 146 | ZFP36 | na |  |  | 18138 | -1.393 | -0.3142 | Yes |
| 147 | KDM6B | na |  |  | 18155 | -1.402 | -0.3073 | Yes |
| 148 | IL1B | na |  |  | 18217 | -1.433 | -0.3026 | Yes |
| 149 | NFIL3 | na |  |  | 18225 | -1.442 | -0.2950 | Yes |
| 150 | TRIB1 | na |  |  | 18278 | -1.478 | -0.2896 | Yes |
| 151 | PDE4B | na |  |  | 18287 | -1.482 | -0.2819 | Yes |
| 152 | GFPT2 | na |  |  | 18347 | -1.525 | -0.2766 | Yes |
| 153 | TGIF1 | na |  |  | 18371 | -1.541 | -0.2693 | Yes |
| 154 | BHLHE40 | na |  |  | 18387 | -1.556 | -0.2616 | Yes |
| 155 | CFLAR | na |  |  | 18417 | -1.589 | -0.2543 | Yes |
| 156 | IFNGR2 | na |  |  | 18421 | -1.594 | -0.2457 | Yes |
| 157 | NAMPT | na |  |  | 18422 | -1.594 | -0.2369 | Yes |
| 158 | TRAF1 | na |  |  | 18477 | -1.625 | -0.2308 | Yes |
| 159 | NR4A2 | na |  |  | 18503 | -1.645 | -0.2231 | Yes |
| 160 | PNRC1 | na |  |  | 18528 | -1.672 | -0.2152 | Yes |
| 161 | TSC22D1 | na |  |  | 18536 | -1.680 | -0.2063 | Yes |
| 162 | CCL20 | na |  |  | 18550 | -1.693 | -0.1976 | Yes |
| 163 | BCL6 | na |  |  | 18551 | -1.694 | -0.1883 | Yes |
| 164 | DUSP4 | na |  |  | 18589 | -1.738 | -0.1807 | Yes |
| 165 | PTGS2 | na |  |  | 18633 | -1.789 | -0.1731 | Yes |
| 166 | IL6 | na |  |  | 18776 | -2.020 | -0.1694 | Yes |
| 167 | BTG1 | na |  |  | 18836 | -2.149 | -0.1607 | Yes |
| 168 | SOD2 | na |  |  | 18970 | -2.521 | -0.1538 | Yes |
| 169 | AREG | na |  |  | 18983 | -2.586 | -0.1402 | Yes |
| 170 | ABCA1 | na |  |  | 18984 | -2.587 | -0.1260 | Yes |
| 171 | KLF9 | na |  |  | 19024 | -2.760 | -0.1128 | Yes |
| 172 | SLC2A3 | na |  |  | 19034 | -2.803 | -0.0979 | Yes |
| 173 | BIRC3 | na |  |  | 19043 | -2.872 | -0.0825 | Yes |
| 174 | TNFRSF9 | na |  |  | 19048 | -2.893 | -0.0668 | Yes |
| 175 | CXCL11 | na |  |  | 19118 | -3.311 | -0.0522 | Yes |
| 176 | BMP2 | na |  |  | 19119 | -3.339 | -0.0338 | Yes |
| 177 | KYNU | na |  |  | 19126 | -3.367 | -0.0156 | Yes |
| 178 | SAT1 | na |  |  | 19139 | -3.506 | 0.0031 | Yes |
Table: GSEA details [plain text format]

  

Fig 2: HALLMARK\_TNFA\_SIGNALING\_VIA\_NFKB      
 Blue-Pink O' Gram in the Space of the Analyzed GeneSet

  

Fig 3: HALLMARK\_TNFA\_SIGNALING\_VIA\_NFKB: Random ES distribution      
 Gene set null distribution of ES for **HALLMARK\_TNFA\_SIGNALING\_VIA\_NFKB**

  
