# Supplementary material for: Heparin-based hydrogel scaffolding alters the transcriptomic profile and increases the chemoresistance of MDA-MB-231 triple-negative breast cancer cells
Source: Biomater Sci. 2020 Feb 13;8(10):2786–96. doi: 10.1039/c9bm01481k (PMC7497406; doi:10.1039/c9bm01481k)
Supplement: Supplementary file 2 [file BM-008-C9BM01481K-s002.zip › Supplementary File 4/EGFvControl/Pathways/my_analysis.Gsea.1545200981068/HALLMARK_CHOLESTEROL_HOMEOSTASIS.html]

Details for gene set HALLMARK\_CHOLESTEROL\_HOMEOSTASIS[GSEA]

|  || Dataset | expr.class.cls#EGF\_versus\_CONTROL.class.cls#EGF\_versus\_CONTROL\_repos |
| Phenotype | class.cls#EGF\_versus\_CONTROL\_repos |
| Upregulated in class | CONTROL |
| GeneSet | HALLMARK\_CHOLESTEROL\_HOMEOSTASIS |
| Enrichment Score (ES) | -0.44398177 |
| Normalized Enrichment Score (NES) | -1.8223083 |
| Nominal p-value | 0.0 |
| FDR q-value | 0.0016962896 |
| FWER p-Value | 0.011 |
Table: GSEA Results Summary

  

Fig 1: Enrichment plot: HALLMARK\_CHOLESTEROL\_HOMEOSTASIS      
 Profile of the Running ES Score & Positions of GeneSet Members on the Rank Ordered List

  

| PROBE | DESCRIPTION (from dataset) | GENE SYMBOL | GENE\_TITLE | RANK IN GENE LIST | RANK METRIC SCORE | RUNNING ES | CORE ENRICHMENT || 1 | TMEM97 | na |  |  | 776 | 1.599 | -0.0143 | No |
| 2 | ALCAM | na |  |  | 834 | 1.575 | 0.0086 | No |
| 3 | GPX8 | na |  |  | 1044 | 1.496 | 0.0223 | No |
| 4 | FABP5 | na |  |  | 1404 | 1.376 | 0.0262 | No |
| 5 | CHKA | na |  |  | 2251 | 1.166 | 0.0011 | No |
| 6 | HMGCR | na |  |  | 2377 | 1.137 | 0.0133 | No |
| 7 | TNFRSF12A | na |  |  | 3094 | 1.008 | -0.0076 | No |
| 8 | FDFT1 | na |  |  | 3116 | 1.004 | 0.0078 | No |
| 9 | ACAT2 | na |  |  | 3122 | 1.002 | 0.0240 | No |
| 10 | GNAI1 | na |  |  | 3405 | 0.953 | 0.0250 | No |
| 11 | PPARG | na |  |  | 3915 | 0.866 | 0.0126 | No |
| 12 | EBP | na |  |  | 4090 | 0.838 | 0.0173 | No |
| 13 | FDPS | na |  |  | 4955 | 0.713 | -0.0162 | No |
| 14 | NSDHL | na |  |  | 4974 | 0.712 | -0.0054 | No |
| 15 | PCYT2 | na |  |  | 5484 | 0.640 | -0.0215 | No |
| 16 | ATF5 | na |  |  | 6791 | 0.469 | -0.0820 | No |
| 17 | MVK | na |  |  | 7063 | 0.439 | -0.0890 | No |
| 18 | ATF3 | na |  |  | 7410 | 0.395 | -0.1006 | No |
| 19 | PLSCR1 | na |  |  | 8332 | 0.290 | -0.1439 | No |
| 20 | HMGCS1 | na |  |  | 8453 | 0.278 | -0.1456 | No |
| 21 | STARD4 | na |  |  | 8985 | 0.214 | -0.1699 | No |
| 22 | LPL | na |  |  | 9088 | 0.203 | -0.1719 | No |
| 23 | ATXN2 | na |  |  | 9275 | 0.183 | -0.1786 | No |
| 24 | FAM129A | na |  |  | 9465 | 0.161 | -0.1858 | No |
| 25 | IDI1 | na |  |  | 9872 | 0.119 | -0.2051 | No |
| 26 | LGMN | na |  |  | 10233 | 0.077 | -0.2226 | No |
| 27 | DHCR7 | na |  |  | 10300 | 0.069 | -0.2249 | No |
| 28 | FBXO6 | na |  |  | 10965 | 0.000 | -0.2596 | No |
| 29 | ETHE1 | na |  |  | 11035 | -0.007 | -0.2631 | No |
| 30 | SQLE | na |  |  | 11156 | -0.021 | -0.2691 | No |
| 31 | GUSB | na |  |  | 12358 | -0.164 | -0.3291 | No |
| 32 | ANXA5 | na |  |  | 13034 | -0.249 | -0.3603 | No |
| 33 | LSS | na |  |  | 13109 | -0.259 | -0.3599 | No |
| 34 | CTNNB1 | na |  |  | 13218 | -0.277 | -0.3610 | No |
| 35 | FASN | na |  |  | 13229 | -0.278 | -0.3570 | No |
| 36 | HSD17B7 | na |  |  | 13436 | -0.306 | -0.3627 | No |
| 37 | PMVK | na |  |  | 14256 | -0.415 | -0.3987 | No |
| 38 | STX5 | na |  |  | 14354 | -0.426 | -0.3968 | No |
| 39 | ACSS2 | na |  |  | 14962 | -0.508 | -0.4201 | No |
| 40 | MVD | na |  |  | 15274 | -0.559 | -0.4272 | No |
| 41 | JAG1 | na |  |  | 15596 | -0.604 | -0.4340 | Yes |
| 42 | PLAUR | na |  |  | 15641 | -0.614 | -0.4263 | Yes |
| 43 | GSTM2 | na |  |  | 15706 | -0.628 | -0.4193 | Yes |
| 44 | S100A11 | na |  |  | 15994 | -0.685 | -0.4230 | Yes |
| 45 | TRIB3 | na |  |  | 16278 | -0.753 | -0.4254 | Yes |
| 46 | ECH1 | na |  |  | 16356 | -0.770 | -0.4168 | Yes |
| 47 | MAL2 | na |  |  | 16472 | -0.805 | -0.4096 | Yes |
| 48 | CYP51A1 | na |  |  | 16545 | -0.823 | -0.3998 | Yes |
| 49 | SREBF2 | na |  |  | 16748 | -0.869 | -0.3961 | Yes |
| 50 | ANTXR2 | na |  |  | 17244 | -1.019 | -0.4052 | Yes |
| 51 | LDLR | na |  |  | 17723 | -1.192 | -0.4106 | Yes |
| 52 | CXCL16 | na |  |  | 17758 | -1.205 | -0.3926 | Yes |
| 53 | FADS2 | na |  |  | 17957 | -1.308 | -0.3814 | Yes |
| 54 | ABCA2 | na |  |  | 18102 | -1.378 | -0.3663 | Yes |
| 55 | SEMA3B | na |  |  | 18153 | -1.402 | -0.3458 | Yes |
| 56 | NFIL3 | na |  |  | 18225 | -1.442 | -0.3258 | Yes |
| 57 | PDK3 | na |  |  | 18298 | -1.493 | -0.3051 | Yes |
| 58 | CPEB2 | na |  |  | 18410 | -1.581 | -0.2849 | Yes |
| 59 | CD9 | na |  |  | 18486 | -1.630 | -0.2620 | Yes |
| 60 | CLU | na |  |  | 18498 | -1.641 | -0.2356 | Yes |
| 61 | PNRC1 | na |  |  | 18528 | -1.672 | -0.2096 | Yes |
| 62 | TM7SF2 | na |  |  | 18655 | -1.822 | -0.1862 | Yes |
| 63 | LGALS3 | na |  |  | 18663 | -1.837 | -0.1564 | Yes |
| 64 | TP53INP1 | na |  |  | 18721 | -1.930 | -0.1277 | Yes |
| 65 | ERRFI1 | na |  |  | 18935 | -2.395 | -0.0994 | Yes |
| 66 | SCD | na |  |  | 19013 | -2.713 | -0.0588 | Yes |
| 67 | ALDOC | na |  |  | 19173 | -4.164 | 0.0013 | Yes |
Table: GSEA details [plain text format]

  

Fig 2: HALLMARK\_CHOLESTEROL\_HOMEOSTASIS      
 Blue-Pink O' Gram in the Space of the Analyzed GeneSet

  

Fig 3: HALLMARK\_CHOLESTEROL\_HOMEOSTASIS: Random ES distribution      
 Gene set null distribution of ES for **HALLMARK\_CHOLESTEROL\_HOMEOSTASIS**

  
