# Supplementary material for: Heparin-based hydrogel scaffolding alters the transcriptomic profile and increases the chemoresistance of MDA-MB-231 triple-negative breast cancer cells
Source: Biomater Sci. 2020 Feb 13;8(10):2786–96. doi: 10.1039/c9bm01481k (PMC7497406; doi:10.1039/c9bm01481k)
Supplement: Supplementary file 2 [file BM-008-C9BM01481K-s002.zip › Supplementary File 4/EGFvControl/Pathways/my_analysis.Gsea.1545200981068/HALLMARK_BILE_ACID_METABOLISM.html]

Details for gene set HALLMARK\_BILE\_ACID\_METABOLISM[GSEA]

|  || Dataset | expr.class.cls#EGF\_versus\_CONTROL.class.cls#EGF\_versus\_CONTROL\_repos |
| Phenotype | class.cls#EGF\_versus\_CONTROL\_repos |
| Upregulated in class | CONTROL |
| GeneSet | HALLMARK\_BILE\_ACID\_METABOLISM |
| Enrichment Score (ES) | -0.35453144 |
| Normalized Enrichment Score (NES) | -1.5133816 |
| Nominal p-value | 0.017412934 |
| FDR q-value | 0.017629612 |
| FWER p-Value | 0.191 |
Table: GSEA Results Summary

  

Fig 1: Enrichment plot: HALLMARK\_BILE\_ACID\_METABOLISM      
 Profile of the Running ES Score & Positions of GeneSet Members on the Rank Ordered List

  

| PROBE | DESCRIPTION (from dataset) | GENE SYMBOL | GENE\_TITLE | RANK IN GENE LIST | RANK METRIC SCORE | RUNNING ES | CORE ENRICHMENT || 1 | DIO2 | na |  |  | 253 | 2.028 | 0.0196 | No |
| 2 | NEDD4 | na |  |  | 553 | 1.739 | 0.0321 | No |
| 3 | HSD17B6 | na |  |  | 720 | 1.630 | 0.0498 | No |
| 4 | GCLM | na |  |  | 1109 | 1.475 | 0.0534 | No |
| 5 | GNPAT | na |  |  | 1828 | 1.261 | 0.0362 | No |
| 6 | DHCR24 | na |  |  | 2137 | 1.190 | 0.0394 | No |
| 7 | ABCA5 | na |  |  | 2202 | 1.177 | 0.0551 | No |
| 8 | PFKM | na |  |  | 2446 | 1.124 | 0.0605 | No |
| 9 | ABCD3 | na |  |  | 2448 | 1.124 | 0.0787 | No |
| 10 | SLC23A2 | na |  |  | 2916 | 1.041 | 0.0711 | No |
| 11 | HACL1 | na |  |  | 3804 | 0.883 | 0.0390 | No |
| 12 | ISOC1 | na |  |  | 4105 | 0.836 | 0.0368 | No |
| 13 | TFCP2L1 | na |  |  | 4124 | 0.832 | 0.0494 | No |
| 14 | LIPE | na |  |  | 4401 | 0.791 | 0.0477 | No |
| 15 | ACSL1 | na |  |  | 5124 | 0.689 | 0.0211 | No |
| 16 | EFHC1 | na |  |  | 5637 | 0.620 | 0.0044 | No |
| 17 | ALDH9A1 | na |  |  | 5781 | 0.599 | 0.0066 | No |
| 18 | SLC29A1 | na |  |  | 6979 | 0.449 | -0.0488 | No |
| 19 | NR3C2 | na |  |  | 7084 | 0.435 | -0.0472 | No |
| 20 | GSTK1 | na |  |  | 7100 | 0.433 | -0.0409 | No |
| 21 | CROT | na |  |  | 7490 | 0.386 | -0.0550 | No |
| 22 | MLYCD | na |  |  | 7518 | 0.383 | -0.0502 | No |
| 23 | FDXR | na |  |  | 7707 | 0.362 | -0.0542 | No |
| 24 | SOD1 | na |  |  | 7945 | 0.332 | -0.0612 | No |
| 25 | PEX1 | na |  |  | 8218 | 0.303 | -0.0706 | No |
| 26 | PXMP2 | na |  |  | 8239 | 0.300 | -0.0668 | No |
| 27 | SLCO1A2 | na |  |  | 8281 | 0.296 | -0.0641 | No |
| 28 | PEX16 | na |  |  | 8284 | 0.295 | -0.0595 | No |
| 29 | LONP2 | na |  |  | 8419 | 0.282 | -0.0619 | No |
| 30 | IDH1 | na |  |  | 8504 | 0.271 | -0.0619 | No |
| 31 | NR1I2 | na |  |  | 8778 | 0.239 | -0.0723 | No |
| 32 | PEX13 | na |  |  | 8966 | 0.216 | -0.0786 | No |
| 33 | SLC27A5 | na |  |  | 9057 | 0.207 | -0.0799 | No |
| 34 | CYP46A1 | na |  |  | 9233 | 0.190 | -0.0860 | No |
| 35 | IDI1 | na |  |  | 9872 | 0.119 | -0.1175 | No |
| 36 | HSD17B11 | na |  |  | 10030 | 0.103 | -0.1240 | No |
| 37 | PEX12 | na |  |  | 10066 | 0.098 | -0.1242 | No |
| 38 | SCP2 | na |  |  | 10592 | 0.039 | -0.1511 | No |
| 39 | FADS1 | na |  |  | 10812 | 0.012 | -0.1623 | No |
| 40 | PEX6 | na |  |  | 11097 | -0.017 | -0.1769 | No |
| 41 | PEX19 | na |  |  | 11399 | -0.049 | -0.1919 | No |
| 42 | ABCG4 | na |  |  | 11727 | -0.090 | -0.2075 | No |
| 43 | SLC23A1 | na |  |  | 12332 | -0.160 | -0.2365 | No |
| 44 | RETSAT | na |  |  | 12351 | -0.162 | -0.2348 | No |
| 45 | NUDT12 | na |  |  | 12741 | -0.221 | -0.2516 | No |
| 46 | PNPLA8 | na |  |  | 12940 | -0.238 | -0.2581 | No |
| 47 | PEX26 | na |  |  | 13168 | -0.268 | -0.2657 | No |
| 48 | RXRA | na |  |  | 13249 | -0.281 | -0.2653 | No |
| 49 | BMP6 | na |  |  | 13435 | -0.306 | -0.2700 | No |
| 50 | PHYH | na |  |  | 13576 | -0.327 | -0.2720 | No |
| 51 | ABCA6 | na |  |  | 13577 | -0.327 | -0.2668 | No |
| 52 | GNMT | na |  |  | 13953 | -0.370 | -0.2804 | No |
| 53 | PEX11A | na |  |  | 14006 | -0.379 | -0.2770 | No |
| 54 | ACSL5 | na |  |  | 14462 | -0.439 | -0.2937 | No |
| 55 | HSD17B4 | na |  |  | 14809 | -0.499 | -0.3037 | No |
| 56 | PEX11G | na |  |  | 15319 | -0.569 | -0.3211 | No |
| 57 | HSD3B7 | na |  |  | 15423 | -0.585 | -0.3170 | No |
| 58 | SLC35B2 | na |  |  | 15492 | -0.595 | -0.3110 | No |
| 59 | CAT | na |  |  | 15662 | -0.617 | -0.3098 | No |
| 60 | AMACR | na |  |  | 15886 | -0.663 | -0.3107 | No |
| 61 | BCAR3 | na |  |  | 15894 | -0.665 | -0.3003 | No |
| 62 | NPC1 | na |  |  | 16451 | -0.798 | -0.3165 | No |
| 63 | ABCD1 | na |  |  | 17179 | -1.003 | -0.3383 | Yes |
| 64 | ATXN1 | na |  |  | 17279 | -1.034 | -0.3267 | Yes |
| 65 | ABCA3 | na |  |  | 17487 | -1.118 | -0.3195 | Yes |
| 66 | PAOX | na |  |  | 17492 | -1.120 | -0.3016 | Yes |
| 67 | APOA1 | na |  |  | 17673 | -1.176 | -0.2919 | Yes |
| 68 | PIPOX | na |  |  | 17675 | -1.176 | -0.2730 | Yes |
| 69 | IDH2 | na |  |  | 17795 | -1.224 | -0.2594 | Yes |
| 70 | OPTN | na |  |  | 17950 | -1.301 | -0.2464 | Yes |
| 71 | FADS2 | na |  |  | 17957 | -1.308 | -0.2255 | Yes |
| 72 | ABCA2 | na |  |  | 18102 | -1.378 | -0.2107 | Yes |
| 73 | AR | na |  |  | 18268 | -1.471 | -0.1956 | Yes |
| 74 | PECR | na |  |  | 18512 | -1.654 | -0.1815 | Yes |
| 75 | SLC22A18 | na |  |  | 18521 | -1.664 | -0.1550 | Yes |
| 76 | CYP27A1 | na |  |  | 18645 | -1.808 | -0.1322 | Yes |
| 77 | PEX7 | na |  |  | 18832 | -2.138 | -0.1073 | Yes |
| 78 | PRDX5 | na |  |  | 18957 | -2.467 | -0.0739 | Yes |
| 79 | ABCA1 | na |  |  | 18984 | -2.587 | -0.0334 | Yes |
| 80 | CYP39A1 | na |  |  | 19023 | -2.750 | 0.0092 | Yes |
Table: GSEA details [plain text format]

  

Fig 2: HALLMARK\_BILE\_ACID\_METABOLISM      
 Blue-Pink O' Gram in the Space of the Analyzed GeneSet

  

Fig 3: HALLMARK\_BILE\_ACID\_METABOLISM: Random ES distribution      
 Gene set null distribution of ES for **HALLMARK\_BILE\_ACID\_METABOLISM**

  
