# Supplementary material for: Heparin-based hydrogel scaffolding alters the transcriptomic profile and increases the chemoresistance of MDA-MB-231 triple-negative breast cancer cells
Source: Biomater Sci. 2020 Feb 13;8(10):2786–96. doi: 10.1039/c9bm01481k (PMC7497406; doi:10.1039/c9bm01481k)
Supplement: Supplementary file 2 [file BM-008-C9BM01481K-s002.zip › Supplementary File 4/EGFvControl/Pathways/my_analysis.Gsea.1545200981068/HALLMARK_MTORC1_SIGNALING.html]

Details for gene set HALLMARK\_MTORC1\_SIGNALING[GSEA]

|  || Dataset | expr.class.cls#EGF\_versus\_CONTROL.class.cls#EGF\_versus\_CONTROL\_repos |
| Phenotype | class.cls#EGF\_versus\_CONTROL\_repos |
| Upregulated in class | EGF |
| GeneSet | HALLMARK\_MTORC1\_SIGNALING |
| Enrichment Score (ES) | 0.3653556 |
| Normalized Enrichment Score (NES) | 1.701037 |
| Nominal p-value | 0.0 |
| FDR q-value | 0.0036738797 |
| FWER p-Value | 0.039 |
Table: GSEA Results Summary

  

Fig 1: Enrichment plot: HALLMARK\_MTORC1\_SIGNALING      
 Profile of the Running ES Score & Positions of GeneSet Members on the Rank Ordered List

  

| PROBE | DESCRIPTION (from dataset) | GENE SYMBOL | GENE\_TITLE | RANK IN GENE LIST | RANK METRIC SCORE | RUNNING ES | CORE ENRICHMENT || 1 | CDC25A | na |  |  | 107 | 2.312 | 0.0066 | Yes |
| 2 | PSAT1 | na |  |  | 142 | 2.222 | 0.0166 | Yes |
| 3 | AURKA | na |  |  | 163 | 2.181 | 0.0270 | Yes |
| 4 | SLC1A5 | na |  |  | 171 | 2.171 | 0.0382 | Yes |
| 5 | MTHFD2 | na |  |  | 172 | 2.163 | 0.0496 | Yes |
| 6 | GOT1 | na |  |  | 200 | 2.096 | 0.0593 | Yes |
| 7 | SLC7A5 | na |  |  | 213 | 2.074 | 0.0696 | Yes |
| 8 | ATP5G1 | na |  |  | 281 | 1.978 | 0.0765 | Yes |
| 9 | MAP2K3 | na |  |  | 282 | 1.978 | 0.0870 | Yes |
| 10 | CYB5B | na |  |  | 290 | 1.967 | 0.0970 | Yes |
| 11 | POLR3G | na |  |  | 323 | 1.923 | 0.1055 | Yes |
| 12 | UNG | na |  |  | 348 | 1.892 | 0.1143 | Yes |
| 13 | TFRC | na |  |  | 359 | 1.882 | 0.1237 | Yes |
| 14 | TOMM40 | na |  |  | 454 | 1.800 | 0.1283 | Yes |
| 15 | BUB1 | na |  |  | 455 | 1.797 | 0.1378 | Yes |
| 16 | RRP9 | na |  |  | 522 | 1.757 | 0.1436 | Yes |
| 17 | PNP | na |  |  | 546 | 1.742 | 0.1516 | Yes |
| 18 | HSPD1 | na |  |  | 619 | 1.691 | 0.1567 | Yes |
| 19 | TUBA4A | na |  |  | 624 | 1.689 | 0.1655 | Yes |
| 20 | DDX39A | na |  |  | 731 | 1.621 | 0.1685 | Yes |
| 21 | TMEM97 | na |  |  | 776 | 1.599 | 0.1746 | Yes |
| 22 | IFRD1 | na |  |  | 799 | 1.591 | 0.1819 | Yes |
| 23 | PSMD14 | na |  |  | 825 | 1.577 | 0.1889 | Yes |
| 24 | RRM2 | na |  |  | 890 | 1.557 | 0.1938 | Yes |
| 25 | MCM2 | na |  |  | 906 | 1.550 | 0.2012 | Yes |
| 26 | PSME3 | na |  |  | 924 | 1.543 | 0.2084 | Yes |
| 27 | CACYBP | na |  |  | 1010 | 1.511 | 0.2119 | Yes |
| 28 | PNO1 | na |  |  | 1035 | 1.501 | 0.2186 | Yes |
| 29 | PLK1 | na |  |  | 1074 | 1.488 | 0.2245 | Yes |
| 30 | NUP205 | na |  |  | 1076 | 1.487 | 0.2323 | Yes |
| 31 | GTF2H1 | na |  |  | 1094 | 1.481 | 0.2392 | Yes |
| 32 | ABCF2 | na |  |  | 1187 | 1.445 | 0.2420 | Yes |
| 33 | PSMG1 | na |  |  | 1201 | 1.441 | 0.2490 | Yes |
| 34 | HSPA9 | na |  |  | 1253 | 1.427 | 0.2538 | Yes |
| 35 | CTSC | na |  |  | 1305 | 1.410 | 0.2586 | Yes |
| 36 | CCNF | na |  |  | 1350 | 1.393 | 0.2637 | Yes |
| 37 | SRD5A1 | na |  |  | 1363 | 1.390 | 0.2704 | Yes |
| 38 | EPRS | na |  |  | 1365 | 1.389 | 0.2777 | Yes |
| 39 | ME1 | na |  |  | 1379 | 1.385 | 0.2843 | Yes |
| 40 | RPA1 | na |  |  | 1382 | 1.384 | 0.2915 | Yes |
| 41 | PRDX1 | na |  |  | 1452 | 1.363 | 0.2951 | Yes |
| 42 | SLC7A11 | na |  |  | 1471 | 1.356 | 0.3013 | Yes |
| 43 | MCM4 | na |  |  | 1617 | 1.313 | 0.3007 | Yes |
| 44 | ETF1 | na |  |  | 1652 | 1.307 | 0.3058 | Yes |
| 45 | PPA1 | na |  |  | 1704 | 1.294 | 0.3099 | Yes |
| 46 | PSMC2 | na |  |  | 1722 | 1.290 | 0.3159 | Yes |
| 47 | SDF2L1 | na |  |  | 1751 | 1.279 | 0.3212 | Yes |
| 48 | CCT6A | na |  |  | 1864 | 1.254 | 0.3219 | Yes |
| 49 | ELOVL5 | na |  |  | 1901 | 1.246 | 0.3266 | Yes |
| 50 | DHCR24 | na |  |  | 2137 | 1.190 | 0.3205 | Yes |
| 51 | GLA | na |  |  | 2226 | 1.173 | 0.3221 | Yes |
| 52 | STIP1 | na |  |  | 2297 | 1.157 | 0.3245 | Yes |
| 53 | GMPS | na |  |  | 2348 | 1.145 | 0.3280 | Yes |
| 54 | HMGCR | na |  |  | 2377 | 1.137 | 0.3325 | Yes |
| 55 | HSPA4 | na |  |  | 2383 | 1.136 | 0.3382 | Yes |
| 56 | DHFR | na |  |  | 2437 | 1.126 | 0.3414 | Yes |
| 57 | PSMC4 | na |  |  | 2550 | 1.101 | 0.3413 | Yes |
| 58 | SORD | na |  |  | 2578 | 1.097 | 0.3457 | Yes |
| 59 | COPS5 | na |  |  | 2665 | 1.082 | 0.3469 | Yes |
| 60 | PSMC6 | na |  |  | 2673 | 1.081 | 0.3523 | Yes |
| 61 | PSMA4 | na |  |  | 2701 | 1.076 | 0.3565 | Yes |
| 62 | HPRT1 | na |  |  | 2703 | 1.076 | 0.3622 | Yes |
| 63 | UCHL5 | na |  |  | 2852 | 1.052 | 0.3600 | Yes |
| 64 | TXNRD1 | na |  |  | 2856 | 1.051 | 0.3654 | Yes |
| 65 | ACTR2 | na |  |  | 3112 | 1.004 | 0.3573 | No |
| 66 | PSPH | na |  |  | 3292 | 0.971 | 0.3530 | No |
| 67 | PSMB5 | na |  |  | 3883 | 0.871 | 0.3265 | No |
| 68 | SEC11A | na |  |  | 3889 | 0.870 | 0.3309 | No |
| 69 | ADIPOR2 | na |  |  | 3953 | 0.860 | 0.3321 | No |
| 70 | PITPNB | na |  |  | 4060 | 0.842 | 0.3310 | No |
| 71 | EBP | na |  |  | 4090 | 0.838 | 0.3339 | No |
| 72 | ATP2A2 | na |  |  | 4160 | 0.828 | 0.3346 | No |
| 73 | PSMD12 | na |  |  | 4180 | 0.825 | 0.3380 | No |
| 74 | CXCR4 | na |  |  | 4387 | 0.794 | 0.3314 | No |
| 75 | HMBS | na |  |  | 4409 | 0.790 | 0.3344 | No |
| 76 | PSMA3 | na |  |  | 4449 | 0.785 | 0.3365 | No |
| 77 | ATP6V1D | na |  |  | 4550 | 0.771 | 0.3353 | No |
| 78 | IMMT | na |  |  | 4595 | 0.765 | 0.3371 | No |
| 79 | UFM1 | na |  |  | 4634 | 0.759 | 0.3391 | No |
| 80 | YKT6 | na |  |  | 4749 | 0.739 | 0.3370 | No |
| 81 | ACTR3 | na |  |  | 4821 | 0.726 | 0.3371 | No |
| 82 | TCEA1 | na |  |  | 4826 | 0.725 | 0.3407 | No |
| 83 | QDPR | na |  |  | 4883 | 0.720 | 0.3416 | No |
| 84 | NFKBIB | na |  |  | 4914 | 0.717 | 0.3438 | No |
| 85 | SERP1 | na |  |  | 4992 | 0.709 | 0.3435 | No |
| 86 | SYTL2 | na |  |  | 5076 | 0.697 | 0.3428 | No |
| 87 | EEF1E1 | na |  |  | 5155 | 0.684 | 0.3423 | No |
| 88 | PSMD13 | na |  |  | 5199 | 0.679 | 0.3437 | No |
| 89 | MTHFD2L | na |  |  | 5232 | 0.674 | 0.3455 | No |
| 90 | CORO1A | na |  |  | 5554 | 0.632 | 0.3320 | No |
| 91 | BCAT1 | na |  |  | 5614 | 0.624 | 0.3322 | No |
| 92 | NFYC | na |  |  | 5796 | 0.597 | 0.3258 | No |
| 93 | ARPC5L | na |  |  | 5822 | 0.595 | 0.3276 | No |
| 94 | HSPE1 | na |  |  | 5965 | 0.574 | 0.3232 | No |
| 95 | M6PR | na |  |  | 6185 | 0.544 | 0.3146 | No |
| 96 | ACACA | na |  |  | 6232 | 0.536 | 0.3150 | No |
| 97 | TBK1 | na |  |  | 6327 | 0.523 | 0.3128 | No |
| 98 | ACLY | na |  |  | 6379 | 0.516 | 0.3129 | No |
| 99 | EIF2S2 | na |  |  | 6459 | 0.507 | 0.3114 | No |
| 100 | GSK3B | na |  |  | 6707 | 0.479 | 0.3009 | No |
| 101 | SHMT2 | na |  |  | 6749 | 0.474 | 0.3013 | No |
| 102 | GCLC | na |  |  | 6772 | 0.472 | 0.3026 | No |
| 103 | DDIT3 | na |  |  | 6794 | 0.469 | 0.3040 | No |
| 104 | TES | na |  |  | 6856 | 0.461 | 0.3032 | No |
| 105 | SLC37A4 | na |  |  | 6924 | 0.453 | 0.3021 | No |
| 106 | ELOVL6 | na |  |  | 7276 | 0.411 | 0.2858 | No |
| 107 | USO1 | na |  |  | 7386 | 0.398 | 0.2822 | No |
| 108 | NUFIP1 | na |  |  | 7648 | 0.368 | 0.2704 | No |
| 109 | FDXR | na |  |  | 7707 | 0.362 | 0.2692 | No |
| 110 | CTH | na |  |  | 7970 | 0.329 | 0.2572 | No |
| 111 | NMT1 | na |  |  | 7979 | 0.328 | 0.2585 | No |
| 112 | UBE2D3 | na |  |  | 8015 | 0.323 | 0.2584 | No |
| 113 | PIK3R3 | na |  |  | 8228 | 0.301 | 0.2488 | No |
| 114 | SLC1A4 | na |  |  | 8321 | 0.291 | 0.2455 | No |
| 115 | HMGCS1 | na |  |  | 8453 | 0.278 | 0.2401 | No |
| 116 | IDH1 | na |  |  | 8504 | 0.271 | 0.2389 | No |
| 117 | PPP1R15A | na |  |  | 8701 | 0.246 | 0.2299 | No |
| 118 | TUBG1 | na |  |  | 8708 | 0.245 | 0.2309 | No |
| 119 | STARD4 | na |  |  | 8985 | 0.214 | 0.2175 | No |
| 120 | RIT1 | na |  |  | 9242 | 0.189 | 0.2050 | No |
| 121 | RPN1 | na |  |  | 9273 | 0.183 | 0.2044 | No |
| 122 | FAM129A | na |  |  | 9465 | 0.161 | 0.1952 | No |
| 123 | IDI1 | na |  |  | 9872 | 0.119 | 0.1745 | No |
| 124 | SSR1 | na |  |  | 10012 | 0.105 | 0.1677 | No |
| 125 | ADD3 | na |  |  | 10185 | 0.084 | 0.1591 | No |
| 126 | LGMN | na |  |  | 10233 | 0.077 | 0.1571 | No |
| 127 | DHCR7 | na |  |  | 10300 | 0.069 | 0.1540 | No |
| 128 | FGL2 | na |  |  | 10347 | 0.062 | 0.1519 | No |
| 129 | FADS1 | na |  |  | 10812 | 0.012 | 0.1275 | No |
| 130 | RDH11 | na |  |  | 10948 | 0.003 | 0.1204 | No |
| 131 | ACSL3 | na |  |  | 11105 | -0.017 | 0.1123 | No |
| 132 | SQLE | na |  |  | 11156 | -0.021 | 0.1098 | No |
| 133 | GGA2 | na |  |  | 11204 | -0.027 | 0.1075 | No |
| 134 | BTG2 | na |  |  | 11938 | -0.118 | 0.0695 | No |
| 135 | WARS | na |  |  | 12021 | -0.127 | 0.0659 | No |
| 136 | ASNS | na |  |  | 12529 | -0.188 | 0.0402 | No |
| 137 | SLC6A6 | na |  |  | 12600 | -0.197 | 0.0376 | No |
| 138 | SLC2A1 | na |  |  | 12606 | -0.200 | 0.0384 | No |
| 139 | G6PD | na |  |  | 13071 | -0.252 | 0.0153 | No |
| 140 | PDAP1 | na |  |  | 13491 | -0.315 | -0.0051 | No |
| 141 | PHGDH | na |  |  | 13506 | -0.318 | -0.0041 | No |
| 142 | PGM1 | na |  |  | 13543 | -0.322 | -0.0043 | No |
| 143 | LTA4H | na |  |  | 13613 | -0.333 | -0.0062 | No |
| 144 | RAB1A | na |  |  | 13692 | -0.344 | -0.0085 | No |
| 145 | IFI30 | na |  |  | 13832 | -0.356 | -0.0139 | No |
| 146 | SERPINH1 | na |  |  | 13922 | -0.366 | -0.0167 | No |
| 147 | MLLT11 | na |  |  | 14250 | -0.414 | -0.0317 | No |
| 148 | CCNG1 | na |  |  | 14380 | -0.429 | -0.0362 | No |
| 149 | HK2 | na |  |  | 14409 | -0.434 | -0.0354 | No |
| 150 | GSR | na |  |  | 14564 | -0.456 | -0.0410 | No |
| 151 | SLC9A3R1 | na |  |  | 14781 | -0.492 | -0.0498 | No |
| 152 | CALR | na |  |  | 15148 | -0.538 | -0.0662 | No |
| 153 | GPI | na |  |  | 15768 | -0.641 | -0.0954 | No |
| 154 | CDKN1A | na |  |  | 15950 | -0.678 | -0.1013 | No |
| 155 | PDK1 | na |  |  | 15998 | -0.686 | -0.1002 | No |
| 156 | XBP1 | na |  |  | 16003 | -0.687 | -0.0968 | No |
| 157 | HSP90B1 | na |  |  | 16095 | -0.702 | -0.0978 | No |
| 158 | HSPA5 | na |  |  | 16140 | -0.715 | -0.0964 | No |
| 159 | TRIB3 | na |  |  | 16278 | -0.753 | -0.0996 | No |
| 160 | EDEM1 | na |  |  | 16338 | -0.765 | -0.0986 | No |
| 161 | ALDOA | na |  |  | 16455 | -0.799 | -0.1005 | No |
| 162 | CYP51A1 | na |  |  | 16545 | -0.823 | -0.1008 | No |
| 163 | TPI1 | na |  |  | 16603 | -0.835 | -0.0994 | No |
| 164 | SQSTM1 | na |  |  | 16663 | -0.848 | -0.0980 | No |
| 165 | PFKL | na |  |  | 16708 | -0.859 | -0.0958 | No |
| 166 | GBE1 | na |  |  | 16983 | -0.947 | -0.1052 | No |
| 167 | ITGB2 | na |  |  | 17342 | -1.055 | -0.1185 | No |
| 168 | SKAP2 | na |  |  | 17486 | -1.118 | -0.1201 | No |
| 169 | LDLR | na |  |  | 17723 | -1.192 | -0.1262 | No |
| 170 | INSIG1 | na |  |  | 17727 | -1.193 | -0.1200 | No |
| 171 | PLOD2 | na |  |  | 17729 | -1.194 | -0.1138 | No |
| 172 | DDIT4 | na |  |  | 17752 | -1.202 | -0.1086 | No |
| 173 | CFP | na |  |  | 17862 | -1.258 | -0.1077 | No |
| 174 | FADS2 | na |  |  | 17957 | -1.308 | -0.1057 | No |
| 175 | P4HA1 | na |  |  | 18001 | -1.330 | -0.1009 | No |
| 176 | AK4 | na |  |  | 18050 | -1.351 | -0.0963 | No |
| 177 | FKBP2 | na |  |  | 18140 | -1.394 | -0.0936 | No |
| 178 | GLRX | na |  |  | 18169 | -1.407 | -0.0876 | No |
| 179 | NFIL3 | na |  |  | 18225 | -1.442 | -0.0829 | No |
| 180 | BHLHE40 | na |  |  | 18387 | -1.556 | -0.0832 | No |
| 181 | NAMPT | na |  |  | 18422 | -1.594 | -0.0765 | No |
| 182 | NUPR1 | na |  |  | 18454 | -1.613 | -0.0696 | No |
| 183 | CD9 | na |  |  | 18486 | -1.630 | -0.0626 | No |
| 184 | TM7SF2 | na |  |  | 18655 | -1.822 | -0.0618 | No |
| 185 | PGK1 | na |  |  | 18818 | -2.107 | -0.0592 | No |
| 186 | VLDLR | na |  |  | 18950 | -2.433 | -0.0532 | No |
| 187 | STC1 | na |  |  | 18997 | -2.647 | -0.0416 | No |
| 188 | SCD | na |  |  | 19013 | -2.713 | -0.0281 | No |
| 189 | SLC2A3 | na |  |  | 19034 | -2.803 | -0.0143 | No |
| 190 | EGLN3 | na |  |  | 19178 | -4.329 | 0.0011 | No |
Table: GSEA details [plain text format]

  

Fig 2: HALLMARK\_MTORC1\_SIGNALING      
 Blue-Pink O' Gram in the Space of the Analyzed GeneSet

  

Fig 3: HALLMARK\_MTORC1\_SIGNALING: Random ES distribution      
 Gene set null distribution of ES for **HALLMARK\_MTORC1\_SIGNALING**

  
