# Supplementary material for: Heparin-based hydrogel scaffolding alters the transcriptomic profile and increases the chemoresistance of MDA-MB-231 triple-negative breast cancer cells
Source: Biomater Sci. 2020 Feb 13;8(10):2786–96. doi: 10.1039/c9bm01481k (PMC7497406; doi:10.1039/c9bm01481k)
Supplement: Supplementary file 2 [file BM-008-C9BM01481K-s002.zip › Supplementary File 4/EGFvControl/Pathways/my_analysis.Gsea.1545200981068/HALLMARK_ESTROGEN_RESPONSE_EARLY.html]

Details for gene set HALLMARK\_ESTROGEN\_RESPONSE\_EARLY[GSEA]

|  || Dataset | expr.class.cls#EGF\_versus\_CONTROL.class.cls#EGF\_versus\_CONTROL\_repos |
| Phenotype | class.cls#EGF\_versus\_CONTROL\_repos |
| Upregulated in class | CONTROL |
| GeneSet | HALLMARK\_ESTROGEN\_RESPONSE\_EARLY |
| Enrichment Score (ES) | -0.3116096 |
| Normalized Enrichment Score (NES) | -1.5050149 |
| Nominal p-value | 0.0027247956 |
| FDR q-value | 0.017544074 |
| FWER p-Value | 0.206 |
Table: GSEA Results Summary

  

Fig 1: Enrichment plot: HALLMARK\_ESTROGEN\_RESPONSE\_EARLY      
 Profile of the Running ES Score & Positions of GeneSet Members on the Rank Ordered List

  

| PROBE | DESCRIPTION (from dataset) | GENE SYMBOL | GENE\_TITLE | RANK IN GENE LIST | RANK METRIC SCORE | RUNNING ES | CORE ENRICHMENT || 1 | SLC7A5 | na |  |  | 213 | 2.074 | 0.0021 | No |
| 2 | MYBBP1A | na |  |  | 337 | 1.904 | 0.0078 | No |
| 3 | SNX24 | na |  |  | 371 | 1.872 | 0.0180 | No |
| 4 | SLC26A2 | na |  |  | 537 | 1.748 | 0.0205 | No |
| 5 | DLC1 | na |  |  | 539 | 1.747 | 0.0317 | No |
| 6 | ISG20L2 | na |  |  | 681 | 1.657 | 0.0348 | No |
| 7 | RRP12 | na |  |  | 758 | 1.606 | 0.0411 | No |
| 8 | WWC1 | na |  |  | 786 | 1.595 | 0.0499 | No |
| 9 | SCARB1 | na |  |  | 794 | 1.593 | 0.0597 | No |
| 10 | SLC22A5 | na |  |  | 839 | 1.573 | 0.0675 | No |
| 11 | FKBP4 | na |  |  | 865 | 1.565 | 0.0762 | No |
| 12 | RBBP8 | na |  |  | 927 | 1.541 | 0.0828 | No |
| 13 | FKBP5 | na |  |  | 1120 | 1.472 | 0.0822 | No |
| 14 | BCL2 | na |  |  | 1163 | 1.454 | 0.0892 | No |
| 15 | REEP1 | na |  |  | 1212 | 1.437 | 0.0959 | No |
| 16 | SLC19A2 | na |  |  | 1323 | 1.405 | 0.0991 | No |
| 17 | PODXL | na |  |  | 1428 | 1.369 | 0.1024 | No |
| 18 | PPIF | na |  |  | 1496 | 1.347 | 0.1075 | No |
| 19 | MYBL1 | na |  |  | 1564 | 1.330 | 0.1125 | No |
| 20 | DHRS2 | na |  |  | 1688 | 1.297 | 0.1143 | No |
| 21 | PAPSS2 | na |  |  | 1742 | 1.283 | 0.1197 | No |
| 22 | RASGRP1 | na |  |  | 1769 | 1.276 | 0.1265 | No |
| 23 | ABLIM1 | na |  |  | 1899 | 1.246 | 0.1277 | No |
| 24 | SFN | na |  |  | 1900 | 1.246 | 0.1357 | No |
| 25 | ELOVL5 | na |  |  | 1901 | 1.246 | 0.1437 | No |
| 26 | SIAH2 | na |  |  | 2015 | 1.216 | 0.1455 | No |
| 27 | SH3BP5 | na |  |  | 2169 | 1.185 | 0.1451 | No |
| 28 | GLA | na |  |  | 2226 | 1.173 | 0.1496 | No |
| 29 | SLC7A2 | na |  |  | 2354 | 1.144 | 0.1503 | No |
| 30 | CCND1 | na |  |  | 2400 | 1.133 | 0.1551 | No |
| 31 | HSPB8 | na |  |  | 2666 | 1.081 | 0.1481 | No |
| 32 | FDFT1 | na |  |  | 3116 | 1.004 | 0.1310 | No |
| 33 | MED13L | na |  |  | 3219 | 0.982 | 0.1319 | No |
| 34 | KRT18 | na |  |  | 3236 | 0.980 | 0.1373 | No |
| 35 | SYNGR1 | na |  |  | 3254 | 0.977 | 0.1427 | No |
| 36 | MICB | na |  |  | 3420 | 0.950 | 0.1401 | No |
| 37 | FHL2 | na |  |  | 3426 | 0.948 | 0.1459 | No |
| 38 | CHPT1 | na |  |  | 3549 | 0.928 | 0.1454 | No |
| 39 | MYOF | na |  |  | 3905 | 0.867 | 0.1323 | No |
| 40 | MYC | na |  |  | 3907 | 0.866 | 0.1378 | No |
| 41 | CD44 | na |  |  | 3954 | 0.860 | 0.1408 | No |
| 42 | TBC1D30 | na |  |  | 3965 | 0.857 | 0.1458 | No |
| 43 | MYB | na |  |  | 3976 | 0.854 | 0.1507 | No |
| 44 | SYBU | na |  |  | 4043 | 0.844 | 0.1527 | No |
| 45 | FARP1 | na |  |  | 4232 | 0.817 | 0.1480 | No |
| 46 | RHOBTB3 | na |  |  | 4638 | 0.758 | 0.1316 | No |
| 47 | LRIG1 | na |  |  | 4830 | 0.725 | 0.1262 | No |
| 48 | TTC39A | na |  |  | 4891 | 0.719 | 0.1276 | No |
| 49 | MREG | na |  |  | 4938 | 0.715 | 0.1298 | No |
| 50 | NXT1 | na |  |  | 5060 | 0.699 | 0.1279 | No |
| 51 | STC2 | na |  |  | 5348 | 0.658 | 0.1170 | No |
| 52 | ABHD2 | na |  |  | 5583 | 0.627 | 0.1087 | No |
| 53 | ITPK1 | na |  |  | 5790 | 0.598 | 0.1017 | No |
| 54 | ASB13 | na |  |  | 5924 | 0.581 | 0.0985 | No |
| 55 | PMAIP1 | na |  |  | 6092 | 0.558 | 0.0932 | No |
| 56 | AKAP1 | na |  |  | 6184 | 0.544 | 0.0919 | No |
| 57 | ESRP2 | na |  |  | 6194 | 0.543 | 0.0949 | No |
| 58 | P2RY2 | na |  |  | 6271 | 0.531 | 0.0943 | No |
| 59 | JAK2 | na |  |  | 6438 | 0.510 | 0.0889 | No |
| 60 | TGM2 | na |  |  | 6481 | 0.505 | 0.0899 | No |
| 61 | ZNF185 | na |  |  | 6507 | 0.502 | 0.0918 | No |
| 62 | CYP26B1 | na |  |  | 6716 | 0.478 | 0.0839 | No |
| 63 | FRK | na |  |  | 6801 | 0.467 | 0.0825 | No |
| 64 | PDZK1 | na |  |  | 6915 | 0.454 | 0.0795 | No |
| 65 | KRT8 | na |  |  | 7138 | 0.429 | 0.0706 | No |
| 66 | ARL3 | na |  |  | 7166 | 0.426 | 0.0719 | No |
| 67 | TFAP2C | na |  |  | 7746 | 0.357 | 0.0437 | No |
| 68 | SLC39A6 | na |  |  | 7923 | 0.335 | 0.0366 | No |
| 69 | SLC1A4 | na |  |  | 8321 | 0.291 | 0.0176 | No |
| 70 | PDLIM3 | na |  |  | 8401 | 0.284 | 0.0153 | No |
| 71 | CANT1 | na |  |  | 8515 | 0.269 | 0.0111 | No |
| 72 | TUBB2B | na |  |  | 8518 | 0.269 | 0.0127 | No |
| 73 | TIPARP | na |  |  | 8676 | 0.250 | 0.0060 | No |
| 74 | INPP5F | na |  |  | 9017 | 0.213 | -0.0105 | No |
| 75 | RAPGEFL1 | na |  |  | 9332 | 0.180 | -0.0258 | No |
| 76 | TPD52L1 | na |  |  | 9466 | 0.161 | -0.0318 | No |
| 77 | UNC119 | na |  |  | 9932 | 0.111 | -0.0555 | No |
| 78 | DEPTOR | na |  |  | 10001 | 0.106 | -0.0584 | No |
| 79 | AMFR | na |  |  | 10146 | 0.089 | -0.0654 | No |
| 80 | ADD3 | na |  |  | 10185 | 0.084 | -0.0669 | No |
| 81 | ENDOD1 | na |  |  | 10248 | 0.075 | -0.0697 | No |
| 82 | DHCR7 | na |  |  | 10300 | 0.069 | -0.0719 | No |
| 83 | TGIF2 | na |  |  | 10444 | 0.059 | -0.0790 | No |
| 84 | OPN3 | na |  |  | 10587 | 0.039 | -0.0862 | No |
| 85 | IL17RB | na |  |  | 10623 | 0.035 | -0.0879 | No |
| 86 | KAZN | na |  |  | 10686 | 0.027 | -0.0909 | No |
| 87 | OLFML3 | na |  |  | 10701 | 0.024 | -0.0915 | No |
| 88 | GJA1 | na |  |  | 10996 | -0.003 | -0.1069 | No |
| 89 | RHOD | na |  |  | 11280 | -0.037 | -0.1216 | No |
| 90 | MAPT | na |  |  | 11463 | -0.058 | -0.1308 | No |
| 91 | INHBB | na |  |  | 11465 | -0.058 | -0.1305 | No |
| 92 | HES1 | na |  |  | 11615 | -0.072 | -0.1378 | No |
| 93 | AFF1 | na |  |  | 11645 | -0.077 | -0.1389 | No |
| 94 | IL6ST | na |  |  | 11649 | -0.077 | -0.1385 | No |
| 95 | WFS1 | na |  |  | 11974 | -0.122 | -0.1548 | No |
| 96 | TMEM164 | na |  |  | 12022 | -0.127 | -0.1564 | No |
| 97 | ADCY9 | na |  |  | 12243 | -0.148 | -0.1670 | No |
| 98 | PRSS23 | na |  |  | 12321 | -0.157 | -0.1701 | No |
| 99 | SLC2A1 | na |  |  | 12606 | -0.200 | -0.1837 | No |
| 100 | BCL11B | na |  |  | 12709 | -0.215 | -0.1877 | No |
| 101 | ELF1 | na |  |  | 12748 | -0.221 | -0.1883 | No |
| 102 | CLIC3 | na |  |  | 12943 | -0.238 | -0.1970 | No |
| 103 | FASN | na |  |  | 13229 | -0.278 | -0.2102 | No |
| 104 | TJP3 | na |  |  | 13413 | -0.305 | -0.2178 | No |
| 105 | BAG1 | na |  |  | 13638 | -0.338 | -0.2274 | No |
| 106 | THSD4 | na |  |  | 13834 | -0.356 | -0.2354 | No |
| 107 | PEX11A | na |  |  | 14006 | -0.379 | -0.2420 | No |
| 108 | TOB1 | na |  |  | 14149 | -0.399 | -0.2469 | No |
| 109 | BLVRB | na |  |  | 14274 | -0.418 | -0.2507 | No |
| 110 | MED24 | na |  |  | 14377 | -0.429 | -0.2533 | No |
| 111 | NAV2 | na |  |  | 14526 | -0.448 | -0.2583 | No |
| 112 | FOXC1 | na |  |  | 14625 | -0.465 | -0.2604 | No |
| 113 | NCOR2 | na |  |  | 14701 | -0.480 | -0.2613 | No |
| 114 | SLC9A3R1 | na |  |  | 14781 | -0.492 | -0.2623 | No |
| 115 | CALB2 | na |  |  | 14948 | -0.506 | -0.2678 | No |
| 116 | ADCY1 | na |  |  | 15509 | -0.597 | -0.2934 | No |
| 117 | RAB17 | na |  |  | 15558 | -0.603 | -0.2921 | No |
| 118 | NADSYN1 | na |  |  | 15819 | -0.652 | -0.3015 | No |
| 119 | XBP1 | na |  |  | 16003 | -0.687 | -0.3068 | No |
| 120 | SLC1A1 | na |  |  | 16096 | -0.702 | -0.3071 | Yes |
| 121 | SYT12 | na |  |  | 16171 | -0.726 | -0.3064 | Yes |
| 122 | TSKU | na |  |  | 16207 | -0.732 | -0.3035 | Yes |
| 123 | KLF10 | na |  |  | 16235 | -0.741 | -0.3002 | Yes |
| 124 | CELSR2 | na |  |  | 16281 | -0.754 | -0.2977 | Yes |
| 125 | FAM102A | na |  |  | 16306 | -0.759 | -0.2942 | Yes |
| 126 | RARA | na |  |  | 16355 | -0.770 | -0.2917 | Yes |
| 127 | KLF4 | na |  |  | 16438 | -0.793 | -0.2910 | Yes |
| 128 | RPS6KA2 | na |  |  | 16764 | -0.874 | -0.3025 | Yes |
| 129 | EGR3 | na |  |  | 16779 | -0.878 | -0.2976 | Yes |
| 130 | UGCG | na |  |  | 16903 | -0.922 | -0.2982 | Yes |
| 131 | GAB2 | na |  |  | 16926 | -0.930 | -0.2934 | Yes |
| 132 | KRT15 | na |  |  | 17048 | -0.962 | -0.2936 | Yes |
| 133 | DYNLT3 | na |  |  | 17081 | -0.972 | -0.2890 | Yes |
| 134 | ALDH3B1 | na |  |  | 17123 | -0.985 | -0.2849 | Yes |
| 135 | IGF1R | na |  |  | 17220 | -1.013 | -0.2835 | Yes |
| 136 | MLPH | na |  |  | 17221 | -1.013 | -0.2770 | Yes |
| 137 | ABCA3 | na |  |  | 17487 | -1.118 | -0.2838 | Yes |
| 138 | NRIP1 | na |  |  | 17528 | -1.131 | -0.2786 | Yes |
| 139 | PLA2G16 | na |  |  | 17607 | -1.151 | -0.2754 | Yes |
| 140 | B4GALT1 | na |  |  | 17619 | -1.157 | -0.2685 | Yes |
| 141 | MAST4 | na |  |  | 17653 | -1.168 | -0.2628 | Yes |
| 142 | KDM4B | na |  |  | 17655 | -1.169 | -0.2554 | Yes |
| 143 | FOS | na |  |  | 17698 | -1.185 | -0.2500 | Yes |
| 144 | AQP3 | na |  |  | 17703 | -1.187 | -0.2426 | Yes |
| 145 | NBL1 | na |  |  | 17771 | -1.213 | -0.2384 | Yes |
| 146 | CELSR1 | na |  |  | 17918 | -1.286 | -0.2378 | Yes |
| 147 | PTGES | na |  |  | 17960 | -1.311 | -0.2316 | Yes |
| 148 | SLC37A1 | na |  |  | 17988 | -1.323 | -0.2246 | Yes |
| 149 | ELF3 | na |  |  | 18029 | -1.345 | -0.2181 | Yes |
| 150 | KRT19 | na |  |  | 18142 | -1.395 | -0.2150 | Yes |
| 151 | SEMA3B | na |  |  | 18153 | -1.402 | -0.2066 | Yes |
| 152 | CISH | na |  |  | 18183 | -1.416 | -0.1990 | Yes |
| 153 | TMPRSS3 | na |  |  | 18244 | -1.453 | -0.1929 | Yes |
| 154 | AR | na |  |  | 18268 | -1.471 | -0.1847 | Yes |
| 155 | BHLHE40 | na |  |  | 18387 | -1.556 | -0.1810 | Yes |
| 156 | HR | na |  |  | 18450 | -1.613 | -0.1739 | Yes |
| 157 | TFF1 | na |  |  | 18525 | -1.668 | -0.1671 | Yes |
| 158 | TIAM1 | na |  |  | 18560 | -1.701 | -0.1580 | Yes |
| 159 | SVIL | na |  |  | 18566 | -1.704 | -0.1474 | Yes |
| 160 | CLDN7 | na |  |  | 18704 | -1.903 | -0.1424 | Yes |
| 161 | TPBG | na |  |  | 18842 | -2.163 | -0.1358 | Yes |
| 162 | ABAT | na |  |  | 18938 | -2.397 | -0.1254 | Yes |
| 163 | SCNN1A | na |  |  | 18942 | -2.414 | -0.1101 | Yes |
| 164 | RAB31 | na |  |  | 18945 | -2.421 | -0.0948 | Yes |
| 165 | DHRS3 | na |  |  | 18963 | -2.502 | -0.0797 | Yes |
| 166 | SEC14L2 | na |  |  | 18982 | -2.584 | -0.0641 | Yes |
| 167 | AREG | na |  |  | 18983 | -2.586 | -0.0475 | Yes |
| 168 | WISP2 | na |  |  | 19038 | -2.829 | -0.0323 | Yes |
| 169 | CA12 | na |  |  | 19077 | -2.973 | -0.0153 | Yes |
| 170 | ANXA9 | na |  |  | 19127 | -3.371 | 0.0037 | Yes |
Table: GSEA details [plain text format]

  

Fig 2: HALLMARK\_ESTROGEN\_RESPONSE\_EARLY      
 Blue-Pink O' Gram in the Space of the Analyzed GeneSet

  

Fig 3: HALLMARK\_ESTROGEN\_RESPONSE\_EARLY: Random ES distribution      
 Gene set null distribution of ES for **HALLMARK\_ESTROGEN\_RESPONSE\_EARLY**

  
