# Supplementary material for: Heparin-based hydrogel scaffolding alters the transcriptomic profile and increases the chemoresistance of MDA-MB-231 triple-negative breast cancer cells
Source: Biomater Sci. 2020 Feb 13;8(10):2786–96. doi: 10.1039/c9bm01481k (PMC7497406; doi:10.1039/c9bm01481k)
Supplement: Supplementary file 2 [file BM-008-C9BM01481K-s002.zip › Supplementary File 4/EGFvControl/Pathways/my_analysis.Gsea.1545200981068/HALLMARK_REACTIVE_OXIGEN_SPECIES_PATHWAY.html]

Details for gene set HALLMARK\_REACTIVE\_OXIGEN\_SPECIES\_PATHWAY[GSEA]

|  || Dataset | expr.class.cls#EGF\_versus\_CONTROL.class.cls#EGF\_versus\_CONTROL\_repos |
| Phenotype | class.cls#EGF\_versus\_CONTROL\_repos |
| Upregulated in class | EGF |
| GeneSet | HALLMARK\_REACTIVE\_OXIGEN\_SPECIES\_PATHWAY |
| Enrichment Score (ES) | 0.3110383 |
| Normalized Enrichment Score (NES) | 1.1129789 |
| Nominal p-value | 0.2818792 |
| FDR q-value | 0.34386906 |
| FWER p-Value | 0.999 |
Table: GSEA Results Summary

  

Fig 1: Enrichment plot: HALLMARK\_REACTIVE\_OXIGEN\_SPECIES\_PATHWAY      
 Profile of the Running ES Score & Positions of GeneSet Members on the Rank Ordered List

  

| PROBE | DESCRIPTION (from dataset) | GENE SYMBOL | GENE\_TITLE | RANK IN GENE LIST | RANK METRIC SCORE | RUNNING ES | CORE ENRICHMENT || 1 | HHEX | na |  |  | 139 | 2.223 | 0.0501 | Yes |
| 2 | NQO1 | na |  |  | 141 | 2.223 | 0.1074 | Yes |
| 3 | ERCC2 | na |  |  | 651 | 1.670 | 0.1240 | Yes |
| 4 | MGST1 | na |  |  | 1050 | 1.494 | 0.1417 | Yes |
| 5 | GCLM | na |  |  | 1109 | 1.475 | 0.1768 | Yes |
| 6 | TXNRD2 | na |  |  | 1251 | 1.427 | 0.2063 | Yes |
| 7 | PRDX1 | na |  |  | 1452 | 1.363 | 0.2310 | Yes |
| 8 | GLRX2 | na |  |  | 1582 | 1.324 | 0.2584 | Yes |
| 9 | TXN | na |  |  | 2234 | 1.172 | 0.2547 | Yes |
| 10 | LSP1 | na |  |  | 2389 | 1.134 | 0.2759 | Yes |
| 11 | TXNRD1 | na |  |  | 2856 | 1.051 | 0.2787 | Yes |
| 12 | HMOX2 | na |  |  | 3015 | 1.025 | 0.2969 | Yes |
| 13 | OXSR1 | na |  |  | 3614 | 0.918 | 0.2894 | Yes |
| 14 | SCAF4 | na |  |  | 3652 | 0.913 | 0.3110 | Yes |
| 15 | PRNP | na |  |  | 4977 | 0.711 | 0.2603 | No |
| 16 | PRDX4 | na |  |  | 6214 | 0.538 | 0.2096 | No |
| 17 | GCLC | na |  |  | 6772 | 0.472 | 0.1927 | No |
| 18 | SOD1 | na |  |  | 7945 | 0.332 | 0.1401 | No |
| 19 | PRDX6 | na |  |  | 8480 | 0.274 | 0.1193 | No |
| 20 | EGLN2 | na |  |  | 9324 | 0.180 | 0.0800 | No |
| 21 | MSRA | na |  |  | 9405 | 0.171 | 0.0802 | No |
| 22 | FTL | na |  |  | 12549 | -0.190 | -0.0790 | No |
| 23 | FES | na |  |  | 12587 | -0.197 | -0.0758 | No |
| 24 | G6PD | na |  |  | 13071 | -0.252 | -0.0945 | No |
| 25 | SRXN1 | na |  |  | 13814 | -0.351 | -0.1242 | No |
| 26 | NDUFS2 | na |  |  | 13895 | -0.365 | -0.1190 | No |
| 27 | CDKN2D | na |  |  | 14162 | -0.400 | -0.1226 | No |
| 28 | ABCC1 | na |  |  | 14177 | -0.402 | -0.1129 | No |
| 29 | GSR | na |  |  | 14564 | -0.456 | -0.1213 | No |
| 30 | NDUFA6 | na |  |  | 14794 | -0.496 | -0.1204 | No |
| 31 | ATOX1 | na |  |  | 15487 | -0.595 | -0.1412 | No |
| 32 | PFKP | na |  |  | 15615 | -0.609 | -0.1321 | No |
| 33 | CAT | na |  |  | 15662 | -0.617 | -0.1186 | No |
| 34 | PDLIM1 | na |  |  | 15891 | -0.664 | -0.1133 | No |
| 35 | STK25 | na |  |  | 15908 | -0.667 | -0.0970 | No |
| 36 | MBP | na |  |  | 15934 | -0.673 | -0.0809 | No |
| 37 | GPX3 | na |  |  | 16209 | -0.732 | -0.0763 | No |
| 38 | PRDX2 | na |  |  | 16340 | -0.766 | -0.0633 | No |
| 39 | GPX4 | na |  |  | 16614 | -0.837 | -0.0560 | No |
| 40 | SBNO2 | na |  |  | 16620 | -0.839 | -0.0346 | No |
| 41 | JUNB | na |  |  | 17499 | -1.123 | -0.0514 | No |
| 42 | GLRX | na |  |  | 18169 | -1.407 | -0.0500 | No |
| 43 | NDUFB4 | na |  |  | 18299 | -1.495 | -0.0182 | No |
| 44 | SOD2 | na |  |  | 18970 | -2.521 | 0.0119 | No |
Table: GSEA details [plain text format]

  

Fig 2: HALLMARK\_REACTIVE\_OXIGEN\_SPECIES\_PATHWAY      
 Blue-Pink O' Gram in the Space of the Analyzed GeneSet

  

Fig 3: HALLMARK\_REACTIVE\_OXIGEN\_SPECIES\_PATHWAY: Random ES distribution      
 Gene set null distribution of ES for **HALLMARK\_REACTIVE\_OXIGEN\_SPECIES\_PATHWAY**

  
