# Supplementary material for: Heparin-based hydrogel scaffolding alters the transcriptomic profile and increases the chemoresistance of MDA-MB-231 triple-negative breast cancer cells
Source: Biomater Sci. 2020 Feb 13;8(10):2786–96. doi: 10.1039/c9bm01481k (PMC7497406; doi:10.1039/c9bm01481k)
Supplement: Supplementary file 2 [file BM-008-C9BM01481K-s002.zip › Supplementary File 4/EGFvControl/Pathways/my_analysis.Gsea.1545200981068/HALLMARK_PROTEIN_SECRETION.html]

Details for gene set HALLMARK\_PROTEIN\_SECRETION[GSEA]

|  || Dataset | expr.class.cls#EGF\_versus\_CONTROL.class.cls#EGF\_versus\_CONTROL\_repos |
| Phenotype | class.cls#EGF\_versus\_CONTROL\_repos |
| Upregulated in class | EGF |
| GeneSet | HALLMARK\_PROTEIN\_SECRETION |
| Enrichment Score (ES) | 0.18341976 |
| Normalized Enrichment Score (NES) | 0.7668792 |
| Nominal p-value | 0.9230769 |
| FDR q-value | 0.9758501 |
| FWER p-Value | 1.0 |
Table: GSEA Results Summary

  

Fig 1: Enrichment plot: HALLMARK\_PROTEIN\_SECRETION      
 Profile of the Running ES Score & Positions of GeneSet Members on the Rank Ordered List

  

| PROBE | DESCRIPTION (from dataset) | GENE SYMBOL | GENE\_TITLE | RANK IN GENE LIST | RANK METRIC SCORE | RUNNING ES | CORE ENRICHMENT || 1 | PPT1 | na |  |  | 379 | 1.863 | 0.0111 | Yes |
| 2 | EGFR | na |  |  | 1241 | 1.429 | -0.0102 | Yes |
| 3 | CTSC | na |  |  | 1305 | 1.410 | 0.0100 | Yes |
| 4 | ZW10 | na |  |  | 1550 | 1.334 | 0.0194 | Yes |
| 5 | SCRN1 | na |  |  | 2223 | 1.173 | 0.0037 | Yes |
| 6 | GLA | na |  |  | 2226 | 1.173 | 0.0231 | Yes |
| 7 | GOSR2 | na |  |  | 2229 | 1.173 | 0.0425 | Yes |
| 8 | ANP32E | na |  |  | 2263 | 1.164 | 0.0601 | Yes |
| 9 | CLTA | na |  |  | 2787 | 1.062 | 0.0504 | Yes |
| 10 | RAB14 | na |  |  | 2938 | 1.036 | 0.0597 | Yes |
| 11 | KRT18 | na |  |  | 3236 | 0.980 | 0.0605 | Yes |
| 12 | AP3S1 | na |  |  | 3272 | 0.974 | 0.0748 | Yes |
| 13 | COG2 | na |  |  | 3364 | 0.959 | 0.0860 | Yes |
| 14 | RER1 | na |  |  | 3520 | 0.934 | 0.0934 | Yes |
| 15 | TMX1 | na |  |  | 3636 | 0.916 | 0.1026 | Yes |
| 16 | AP2M1 | na |  |  | 3681 | 0.907 | 0.1154 | Yes |
| 17 | AP2S1 | na |  |  | 3824 | 0.880 | 0.1226 | Yes |
| 18 | ATP6V1H | na |  |  | 3874 | 0.872 | 0.1345 | Yes |
| 19 | ARFGEF2 | na |  |  | 3956 | 0.860 | 0.1446 | Yes |
| 20 | CLTC | na |  |  | 4040 | 0.844 | 0.1543 | Yes |
| 21 | VAMP7 | na |  |  | 4319 | 0.805 | 0.1531 | Yes |
| 22 | COPB1 | na |  |  | 4407 | 0.790 | 0.1617 | Yes |
| 23 | DOPEY1 | na |  |  | 4481 | 0.780 | 0.1708 | Yes |
| 24 | YKT6 | na |  |  | 4749 | 0.739 | 0.1691 | Yes |
| 25 | VPS45 | na |  |  | 5142 | 0.685 | 0.1600 | Yes |
| 26 | DNM1L | na |  |  | 5311 | 0.663 | 0.1622 | Yes |
| 27 | SEC22B | na |  |  | 5325 | 0.661 | 0.1725 | Yes |
| 28 | NAPG | na |  |  | 5519 | 0.635 | 0.1730 | Yes |
| 29 | OCRL | na |  |  | 5522 | 0.635 | 0.1834 | Yes |
| 30 | AP2B1 | na |  |  | 5945 | 0.578 | 0.1709 | No |
| 31 | M6PR | na |  |  | 6185 | 0.544 | 0.1675 | No |
| 32 | AP1G1 | na |  |  | 6344 | 0.522 | 0.1679 | No |
| 33 | TMED2 | na |  |  | 6719 | 0.478 | 0.1562 | No |
| 34 | AP3B1 | na |  |  | 6887 | 0.458 | 0.1551 | No |
| 35 | GBF1 | na |  |  | 7249 | 0.415 | 0.1431 | No |
| 36 | MAPK1 | na |  |  | 7252 | 0.414 | 0.1499 | No |
| 37 | SNX2 | na |  |  | 7277 | 0.410 | 0.1554 | No |
| 38 | USO1 | na |  |  | 7386 | 0.398 | 0.1564 | No |
| 39 | TOM1L1 | na |  |  | 7508 | 0.385 | 0.1565 | No |
| 40 | MON2 | na |  |  | 7817 | 0.347 | 0.1461 | No |
| 41 | TPD52 | na |  |  | 7821 | 0.347 | 0.1517 | No |
| 42 | SOD1 | na |  |  | 7945 | 0.332 | 0.1508 | No |
| 43 | ARCN1 | na |  |  | 7982 | 0.327 | 0.1544 | No |
| 44 | KIF1B | na |  |  | 8020 | 0.323 | 0.1578 | No |
| 45 | STAM | na |  |  | 8152 | 0.310 | 0.1561 | No |
| 46 | SSPN | na |  |  | 8214 | 0.303 | 0.1579 | No |
| 47 | ARFIP1 | na |  |  | 8334 | 0.290 | 0.1565 | No |
| 48 | VAMP4 | na |  |  | 8408 | 0.283 | 0.1574 | No |
| 49 | CLN5 | na |  |  | 8487 | 0.273 | 0.1579 | No |
| 50 | COPB2 | na |  |  | 8520 | 0.268 | 0.1607 | No |
| 51 | YIPF6 | na |  |  | 8694 | 0.247 | 0.1557 | No |
| 52 | TSG101 | na |  |  | 8709 | 0.245 | 0.1590 | No |
| 53 | ARFGEF1 | na |  |  | 8721 | 0.244 | 0.1625 | No |
| 54 | GOLGA4 | na |  |  | 8877 | 0.227 | 0.1582 | No |
| 55 | BET1 | na |  |  | 8937 | 0.220 | 0.1587 | No |
| 56 | CAV2 | na |  |  | 9217 | 0.191 | 0.1473 | No |
| 57 | STX7 | na |  |  | 9326 | 0.180 | 0.1447 | No |
| 58 | SGMS1 | na |  |  | 9826 | 0.124 | 0.1206 | No |
| 59 | ARFGAP3 | na |  |  | 10060 | 0.098 | 0.1100 | No |
| 60 | VPS4B | na |  |  | 10468 | 0.057 | 0.0897 | No |
| 61 | NAPA | na |  |  | 10958 | 0.002 | 0.0641 | No |
| 62 | LAMP2 | na |  |  | 11032 | -0.007 | 0.0604 | No |
| 63 | RAB2A | na |  |  | 11148 | -0.020 | 0.0547 | No |
| 64 | SNAP23 | na |  |  | 11694 | -0.084 | 0.0276 | No |
| 65 | GALC | na |  |  | 11877 | -0.110 | 0.0199 | No |
| 66 | CD63 | na |  |  | 12213 | -0.145 | 0.0048 | No |
| 67 | RPS6KA3 | na |  |  | 12300 | -0.154 | 0.0028 | No |
| 68 | ARF1 | na |  |  | 12303 | -0.155 | 0.0053 | No |
| 69 | RAB22A | na |  |  | 12339 | -0.161 | 0.0061 | No |
| 70 | SEC31A | na |  |  | 12524 | -0.187 | -0.0004 | No |
| 71 | STX16 | na |  |  | 12684 | -0.210 | -0.0052 | No |
| 72 | LMAN1 | na |  |  | 12736 | -0.220 | -0.0042 | No |
| 73 | VAMP3 | na |  |  | 13008 | -0.244 | -0.0143 | No |
| 74 | RAB5A | na |  |  | 13370 | -0.299 | -0.0283 | No |
| 75 | ADAM10 | na |  |  | 14023 | -0.381 | -0.0561 | No |
| 76 | ATP6V1B1 | na |  |  | 14371 | -0.428 | -0.0671 | No |
| 77 | SEC24D | na |  |  | 14410 | -0.434 | -0.0619 | No |
| 78 | TMED10 | na |  |  | 14453 | -0.437 | -0.0568 | No |
| 79 | TSPAN8 | na |  |  | 14522 | -0.447 | -0.0529 | No |
| 80 | SCAMP3 | na |  |  | 14598 | -0.463 | -0.0492 | No |
| 81 | ERGIC3 | na |  |  | 14744 | -0.487 | -0.0487 | No |
| 82 | COPE | na |  |  | 15122 | -0.532 | -0.0596 | No |
| 83 | SCAMP1 | na |  |  | 15486 | -0.595 | -0.0687 | No |
| 84 | IGF2R | na |  |  | 15552 | -0.602 | -0.0621 | No |
| 85 | CLCN3 | na |  |  | 15758 | -0.639 | -0.0622 | No |
| 86 | GNAS | na |  |  | 16158 | -0.720 | -0.0711 | No |
| 87 | STX12 | na |  |  | 16686 | -0.853 | -0.0845 | No |
| 88 | RAB9A | na |  |  | 16798 | -0.885 | -0.0756 | No |
| 89 | ATP1A1 | na |  |  | 17961 | -1.311 | -0.1146 | No |
| 90 | ATP7A | na |  |  | 18638 | -1.802 | -0.1201 | No |
| 91 | PAM | na |  |  | 18737 | -1.945 | -0.0928 | No |
| 92 | ICA1 | na |  |  | 18822 | -2.115 | -0.0621 | No |
| 93 | BNIP3 | na |  |  | 18904 | -2.327 | -0.0277 | No |
| 94 | ABCA1 | na |  |  | 18984 | -2.587 | 0.0112 | No |
Table: GSEA details [plain text format]

  

Fig 2: HALLMARK\_PROTEIN\_SECRETION      
 Blue-Pink O' Gram in the Space of the Analyzed GeneSet

  

Fig 3: HALLMARK\_PROTEIN\_SECRETION: Random ES distribution      
 Gene set null distribution of ES for **HALLMARK\_PROTEIN\_SECRETION**

  
