# Supplementary material for: Heparin-based hydrogel scaffolding alters the transcriptomic profile and increases the chemoresistance of MDA-MB-231 triple-negative breast cancer cells
Source: Biomater Sci. 2020 Feb 13;8(10):2786–96. doi: 10.1039/c9bm01481k (PMC7497406; doi:10.1039/c9bm01481k)
Supplement: Supplementary file 2 [file BM-008-C9BM01481K-s002.zip › Supplementary File 4/EGFvControl/Pathways/my_analysis.Gsea.1545200981068/HALLMARK_APICAL_SURFACE.html]

Details for gene set HALLMARK\_APICAL\_SURFACE[GSEA]

|  || Dataset | expr.class.cls#EGF\_versus\_CONTROL.class.cls#EGF\_versus\_CONTROL\_repos |
| Phenotype | class.cls#EGF\_versus\_CONTROL\_repos |
| Upregulated in class | CONTROL |
| GeneSet | HALLMARK\_APICAL\_SURFACE |
| Enrichment Score (ES) | -0.3219462 |
| Normalized Enrichment Score (NES) | -1.0814459 |
| Nominal p-value | 0.32126698 |
| FDR q-value | 0.31464246 |
| FWER p-Value | 0.997 |
Table: GSEA Results Summary

  

Fig 1: Enrichment plot: HALLMARK\_APICAL\_SURFACE      
 Profile of the Running ES Score & Positions of GeneSet Members on the Rank Ordered List

  

| PROBE | DESCRIPTION (from dataset) | GENE SYMBOL | GENE\_TITLE | RANK IN GENE LIST | RANK METRIC SCORE | RUNNING ES | CORE ENRICHMENT || 1 | ATP8B1 | na |  |  | 945 | 1.532 | 0.0219 | No |
| 2 | ATP6V0A4 | na |  |  | 1005 | 1.512 | 0.0890 | No |
| 3 | BRCA1 | na |  |  | 1620 | 1.312 | 0.1179 | No |
| 4 | ADIPOR2 | na |  |  | 3953 | 0.860 | 0.0362 | No |
| 5 | SCUBE1 | na |  |  | 3979 | 0.854 | 0.0746 | No |
| 6 | SHROOM2 | na |  |  | 5400 | 0.650 | 0.0307 | No |
| 7 | SLC34A3 | na |  |  | 5683 | 0.612 | 0.0444 | No |
| 8 | LYN | na |  |  | 7669 | 0.366 | -0.0422 | No |
| 9 | GATA3 | na |  |  | 8290 | 0.294 | -0.0609 | No |
| 10 | CD160 | na |  |  | 8457 | 0.278 | -0.0566 | No |
| 11 | AKAP7 | na |  |  | 8483 | 0.274 | -0.0452 | No |
| 12 | AFAP1L2 | na |  |  | 8848 | 0.231 | -0.0535 | No |
| 13 | MDGA1 | na |  |  | 9785 | 0.127 | -0.0964 | No |
| 14 | CROCC | na |  |  | 10805 | 0.013 | -0.1489 | No |
| 15 | NCOA6 | na |  |  | 12158 | -0.137 | -0.2131 | No |
| 16 | SRPX | na |  |  | 13308 | -0.288 | -0.2596 | No |
| 17 | ADAM10 | na |  |  | 14023 | -0.381 | -0.2792 | No |
| 18 | HSPB1 | na |  |  | 14244 | -0.413 | -0.2715 | No |
| 19 | DCBLD2 | na |  |  | 14704 | -0.481 | -0.2731 | No |
| 20 | PLAUR | na |  |  | 15641 | -0.614 | -0.2934 | Yes |
| 21 | EPHB4 | na |  |  | 15913 | -0.668 | -0.2766 | Yes |
| 22 | FLOT2 | na |  |  | 16089 | -0.702 | -0.2531 | Yes |
| 23 | EFNA5 | na |  |  | 16633 | -0.841 | -0.2424 | Yes |
| 24 | GSTM3 | na |  |  | 16973 | -0.943 | -0.2162 | Yes |
| 25 | B4GALT1 | na |  |  | 17619 | -1.157 | -0.1961 | Yes |
| 26 | TMEM8B | na |  |  | 17632 | -1.161 | -0.1428 | Yes |
| 27 | PCSK9 | na |  |  | 18266 | -1.470 | -0.1076 | Yes |
| 28 | APP | na |  |  | 18352 | -1.528 | -0.0411 | Yes |
| 29 | CX3CL1 | na |  |  | 18661 | -1.834 | 0.0280 | Yes |
Table: GSEA details [plain text format]

  

Fig 2: HALLMARK\_APICAL\_SURFACE      
 Blue-Pink O' Gram in the Space of the Analyzed GeneSet

  

Fig 3: HALLMARK\_APICAL\_SURFACE: Random ES distribution      
 Gene set null distribution of ES for **HALLMARK\_APICAL\_SURFACE**

  
