# Supplementary material for: Heparin-based hydrogel scaffolding alters the transcriptomic profile and increases the chemoresistance of MDA-MB-231 triple-negative breast cancer cells
Source: Biomater Sci. 2020 Feb 13;8(10):2786–96. doi: 10.1039/c9bm01481k (PMC7497406; doi:10.1039/c9bm01481k)
Supplement: Supplementary file 2 [file BM-008-C9BM01481K-s002.zip › Supplementary File 4/EGFvControl/Pathways/my_analysis.Gsea.1545200981068/HALLMARK_EPITHELIAL_MESENCHYMAL_TRANSITION.html]

Details for gene set HALLMARK\_EPITHELIAL\_MESENCHYMAL\_TRANSITION[GSEA]

|  || Dataset | expr.class.cls#EGF\_versus\_CONTROL.class.cls#EGF\_versus\_CONTROL\_repos |
| Phenotype | class.cls#EGF\_versus\_CONTROL\_repos |
| Upregulated in class | CONTROL |
| GeneSet | HALLMARK\_EPITHELIAL\_MESENCHYMAL\_TRANSITION |
| Enrichment Score (ES) | -0.4666636 |
| Normalized Enrichment Score (NES) | -2.2168403 |
| Nominal p-value | 0.0 |
| FDR q-value | 3.3333336E-4 |
| FWER p-Value | 0.001 |
Table: GSEA Results Summary

  

Fig 1: Enrichment plot: HALLMARK\_EPITHELIAL\_MESENCHYMAL\_TRANSITION      
 Profile of the Running ES Score & Positions of GeneSet Members on the Rank Ordered List

  

| PROBE | DESCRIPTION (from dataset) | GENE SYMBOL | GENE\_TITLE | RANK IN GENE LIST | RANK METRIC SCORE | RUNNING ES | CORE ENRICHMENT || 1 | OXTR | na |  |  | 34 | 2.794 | 0.0162 | No |
| 2 | BDNF | na |  |  | 126 | 2.261 | 0.0260 | No |
| 3 | RGS4 | na |  |  | 147 | 2.214 | 0.0392 | No |
| 4 | COL5A2 | na |  |  | 273 | 1.994 | 0.0455 | No |
| 5 | DKK1 | na |  |  | 418 | 1.831 | 0.0497 | No |
| 6 | TPM1 | na |  |  | 616 | 1.693 | 0.0503 | No |
| 7 | SNAI2 | na |  |  | 716 | 1.632 | 0.0556 | No |
| 8 | COL12A1 | na |  |  | 763 | 1.603 | 0.0635 | No |
| 9 | FSTL1 | na |  |  | 1011 | 1.511 | 0.0603 | No |
| 10 | CTGF | na |  |  | 1080 | 1.486 | 0.0663 | No |
| 11 | MCM7 | na |  |  | 1099 | 1.479 | 0.0749 | No |
| 12 | GADD45B | na |  |  | 1283 | 1.417 | 0.0744 | No |
| 13 | FBN1 | na |  |  | 1368 | 1.388 | 0.0789 | No |
| 14 | ABI3BP | na |  |  | 1931 | 1.240 | 0.0574 | No |
| 15 | ADAM12 | na |  |  | 1937 | 1.238 | 0.0651 | No |
| 16 | COL16A1 | na |  |  | 2030 | 1.212 | 0.0681 | No |
| 17 | ECM2 | na |  |  | 2064 | 1.205 | 0.0741 | No |
| 18 | PMEPA1 | na |  |  | 2174 | 1.184 | 0.0761 | No |
| 19 | LRRC15 | na |  |  | 2182 | 1.182 | 0.0833 | No |
| 20 | FSTL3 | na |  |  | 2430 | 1.127 | 0.0776 | No |
| 21 | PVR | na |  |  | 2784 | 1.062 | 0.0659 | No |
| 22 | TNFRSF12A | na |  |  | 3094 | 1.008 | 0.0562 | No |
| 23 | COL6A3 | na |  |  | 3117 | 1.003 | 0.0615 | No |
| 24 | TNFRSF11B | na |  |  | 3241 | 0.979 | 0.0613 | No |
| 25 | GLIPR1 | na |  |  | 3949 | 0.861 | 0.0298 | No |
| 26 | CD44 | na |  |  | 3954 | 0.860 | 0.0351 | No |
| 27 | LAMA2 | na |  |  | 4257 | 0.815 | 0.0245 | No |
| 28 | PCOLCE2 | na |  |  | 4341 | 0.802 | 0.0253 | No |
| 29 | CAP2 | na |  |  | 4663 | 0.755 | 0.0133 | No |
| 30 | GPX7 | na |  |  | 5482 | 0.641 | -0.0255 | No |
| 31 | FERMT2 | na |  |  | 5807 | 0.596 | -0.0387 | No |
| 32 | CALU | na |  |  | 5998 | 0.571 | -0.0450 | No |
| 33 | FBLN1 | na |  |  | 6025 | 0.567 | -0.0427 | No |
| 34 | GADD45A | na |  |  | 6102 | 0.555 | -0.0431 | No |
| 35 | NOTCH2 | na |  |  | 6144 | 0.549 | -0.0417 | No |
| 36 | FAS | na |  |  | 6266 | 0.532 | -0.0446 | No |
| 37 | BASP1 | na |  |  | 6423 | 0.511 | -0.0495 | No |
| 38 | TGM2 | na |  |  | 6481 | 0.505 | -0.0493 | No |
| 39 | ACTA2 | na |  |  | 6747 | 0.474 | -0.0601 | No |
| 40 | PPIB | na |  |  | 6935 | 0.452 | -0.0670 | No |
| 41 | VIM | na |  |  | 6989 | 0.448 | -0.0669 | No |
| 42 | COL7A1 | na |  |  | 7127 | 0.430 | -0.0714 | No |
| 43 | PFN2 | na |  |  | 7627 | 0.371 | -0.0952 | No |
| 44 | MMP3 | na |  |  | 7932 | 0.334 | -0.1090 | No |
| 45 | SDC1 | na |  |  | 7964 | 0.330 | -0.1085 | No |
| 46 | VEGFC | na |  |  | 8088 | 0.317 | -0.1129 | No |
| 47 | VCAN | na |  |  | 8574 | 0.265 | -0.1366 | No |
| 48 | COPA | na |  |  | 8623 | 0.257 | -0.1375 | No |
| 49 | MAGEE1 | na |  |  | 8901 | 0.223 | -0.1506 | No |
| 50 | TGFBR3 | na |  |  | 8951 | 0.217 | -0.1518 | No |
| 51 | TAGLN | na |  |  | 9033 | 0.211 | -0.1547 | No |
| 52 | ELN | na |  |  | 9228 | 0.190 | -0.1636 | No |
| 53 | MYLK | na |  |  | 9344 | 0.178 | -0.1685 | No |
| 54 | MYL9 | na |  |  | 9571 | 0.148 | -0.1794 | No |
| 55 | MATN3 | na |  |  | 9671 | 0.142 | -0.1837 | No |
| 56 | SGCB | na |  |  | 9740 | 0.132 | -0.1864 | No |
| 57 | QSOX1 | na |  |  | 10103 | 0.095 | -0.2048 | No |
| 58 | MEST | na |  |  | 10122 | 0.093 | -0.2052 | No |
| 59 | INHBA | na |  |  | 10150 | 0.088 | -0.2060 | No |
| 60 | GPC1 | na |  |  | 10254 | 0.075 | -0.2109 | No |
| 61 | NT5E | na |  |  | 10569 | 0.043 | -0.2272 | No |
| 62 | GJA1 | na |  |  | 10996 | -0.003 | -0.2495 | No |
| 63 | EFEMP2 | na |  |  | 11064 | -0.012 | -0.2529 | No |
| 64 | COL4A2 | na |  |  | 11618 | -0.072 | -0.2815 | No |
| 65 | NID2 | na |  |  | 11674 | -0.081 | -0.2839 | No |
| 66 | COL4A1 | na |  |  | 11875 | -0.110 | -0.2937 | No |
| 67 | ID2 | na |  |  | 11940 | -0.118 | -0.2963 | No |
| 68 | SPARC | na |  |  | 12049 | -0.132 | -0.3011 | No |
| 69 | THBS2 | na |  |  | 12176 | -0.140 | -0.3068 | No |
| 70 | CALD1 | na |  |  | 12366 | -0.165 | -0.3156 | No |
| 71 | PLOD3 | na |  |  | 12442 | -0.177 | -0.3184 | No |
| 72 | JUN | na |  |  | 12605 | -0.199 | -0.3257 | No |
| 73 | COL1A1 | na |  |  | 12777 | -0.227 | -0.3332 | No |
| 74 | APLP1 | na |  |  | 13196 | -0.273 | -0.3534 | No |
| 75 | COL8A2 | na |  |  | 13401 | -0.302 | -0.3621 | No |
| 76 | WIPF1 | na |  |  | 13594 | -0.329 | -0.3701 | No |
| 77 | TPM4 | na |  |  | 13876 | -0.362 | -0.3825 | No |
| 78 | SERPINH1 | na |  |  | 13922 | -0.366 | -0.3825 | No |
| 79 | PTX3 | na |  |  | 13978 | -0.374 | -0.3830 | No |
| 80 | SNTB1 | na |  |  | 14027 | -0.382 | -0.3830 | No |
| 81 | SPOCK1 | na |  |  | 14141 | -0.397 | -0.3864 | No |
| 82 | CAPG | na |  |  | 14567 | -0.458 | -0.4058 | No |
| 83 | GEM | na |  |  | 14920 | -0.503 | -0.4210 | No |
| 84 | VCAM1 | na |  |  | 15075 | -0.525 | -0.4257 | No |
| 85 | PMP22 | na |  |  | 15157 | -0.538 | -0.4265 | No |
| 86 | CADM1 | na |  |  | 15381 | -0.579 | -0.4345 | No |
| 87 | PLAUR | na |  |  | 15641 | -0.614 | -0.4441 | No |
| 88 | IL15 | na |  |  | 15840 | -0.654 | -0.4503 | No |
| 89 | MATN2 | na |  |  | 16030 | -0.692 | -0.4558 | No |
| 90 | EMP3 | na |  |  | 16130 | -0.712 | -0.4564 | No |
| 91 | LAMC1 | na |  |  | 16327 | -0.763 | -0.4617 | Yes |
| 92 | DAB2 | na |  |  | 16390 | -0.779 | -0.4600 | Yes |
| 93 | RHOB | na |  |  | 16399 | -0.781 | -0.4554 | Yes |
| 94 | LGALS1 | na |  |  | 16602 | -0.835 | -0.4606 | Yes |
| 95 | TNC | na |  |  | 16616 | -0.837 | -0.4559 | Yes |
| 96 | COL5A1 | na |  |  | 16811 | -0.890 | -0.4603 | Yes |
| 97 | MMP1 | na |  |  | 16900 | -0.921 | -0.4590 | Yes |
| 98 | PDLIM4 | na |  |  | 16928 | -0.930 | -0.4545 | Yes |
| 99 | ECM1 | na |  |  | 16989 | -0.949 | -0.4515 | Yes |
| 100 | SLIT2 | na |  |  | 17135 | -0.990 | -0.4527 | Yes |
| 101 | CXCL1 | na |  |  | 17379 | -1.069 | -0.4586 | Yes |
| 102 | SERPINE2 | na |  |  | 17404 | -1.080 | -0.4529 | Yes |
| 103 | TNFAIP3 | na |  |  | 17494 | -1.121 | -0.4504 | Yes |
| 104 | FZD8 | na |  |  | 17554 | -1.133 | -0.4461 | Yes |
| 105 | HTRA1 | na |  |  | 17558 | -1.135 | -0.4390 | Yes |
| 106 | COL6A2 | na |  |  | 17567 | -1.138 | -0.4321 | Yes |
| 107 | TGFB1 | na |  |  | 17599 | -1.148 | -0.4263 | Yes |
| 108 | TIMP1 | na |  |  | 17600 | -1.148 | -0.4189 | Yes |
| 109 | ITGAV | na |  |  | 17601 | -1.149 | -0.4115 | Yes |
| 110 | PLOD1 | na |  |  | 17650 | -1.167 | -0.4065 | Yes |
| 111 | LOXL1 | na |  |  | 17682 | -1.178 | -0.4006 | Yes |
| 112 | SDC4 | na |  |  | 17717 | -1.190 | -0.3947 | Yes |
| 113 | PLOD2 | na |  |  | 17729 | -1.194 | -0.3876 | Yes |
| 114 | PTHLH | na |  |  | 17793 | -1.223 | -0.3830 | Yes |
| 115 | LOXL2 | na |  |  | 17814 | -1.231 | -0.3761 | Yes |
| 116 | TFPI2 | na |  |  | 17846 | -1.252 | -0.3697 | Yes |
| 117 | TGFBI | na |  |  | 17865 | -1.259 | -0.3625 | Yes |
| 118 | SLC6A8 | na |  |  | 17923 | -1.288 | -0.3572 | Yes |
| 119 | PCOLCE | na |  |  | 17987 | -1.322 | -0.3520 | Yes |
| 120 | CD59 | na |  |  | 17994 | -1.325 | -0.3438 | Yes |
| 121 | ITGA5 | na |  |  | 18016 | -1.340 | -0.3362 | Yes |
| 122 | WNT5A | na |  |  | 18031 | -1.345 | -0.3283 | Yes |
| 123 | PDGFRB | na |  |  | 18059 | -1.357 | -0.3210 | Yes |
| 124 | CTHRC1 | na |  |  | 18114 | -1.382 | -0.3149 | Yes |
| 125 | ITGB5 | na |  |  | 18154 | -1.402 | -0.3079 | Yes |
| 126 | LOX | na |  |  | 18158 | -1.403 | -0.2991 | Yes |
| 127 | SCG2 | na |  |  | 18189 | -1.419 | -0.2915 | Yes |
| 128 | ENO2 | na |  |  | 18286 | -1.481 | -0.2870 | Yes |
| 129 | TPM2 | na |  |  | 18358 | -1.533 | -0.2808 | Yes |
| 130 | IL32 | na |  |  | 18390 | -1.558 | -0.2724 | Yes |
| 131 | LRP1 | na |  |  | 18393 | -1.561 | -0.2625 | Yes |
| 132 | IGFBP3 | na |  |  | 18475 | -1.624 | -0.2563 | Yes |
| 133 | SFRP1 | na |  |  | 18535 | -1.679 | -0.2486 | Yes |
| 134 | EDIL3 | na |  |  | 18687 | -1.874 | -0.2444 | Yes |
| 135 | IL6 | na |  |  | 18776 | -2.020 | -0.2360 | Yes |
| 136 | COL1A2 | na |  |  | 18789 | -2.040 | -0.2235 | Yes |
| 137 | ITGA2 | na |  |  | 18843 | -2.164 | -0.2124 | Yes |
| 138 | NNMT | na |  |  | 18846 | -2.171 | -0.1985 | Yes |
| 139 | CDH11 | na |  |  | 18848 | -2.179 | -0.1845 | Yes |
| 140 | TIMP3 | na |  |  | 18862 | -2.233 | -0.1708 | Yes |
| 141 | FUCA1 | na |  |  | 18883 | -2.288 | -0.1571 | Yes |
| 142 | BMP1 | na |  |  | 18897 | -2.311 | -0.1429 | Yes |
| 143 | ITGB3 | na |  |  | 18931 | -2.380 | -0.1293 | Yes |
| 144 | AREG | na |  |  | 18983 | -2.586 | -0.1153 | Yes |
| 145 | LAMC2 | na |  |  | 18985 | -2.588 | -0.0987 | Yes |
| 146 | LAMA3 | na |  |  | 19037 | -2.819 | -0.0832 | Yes |
| 147 | PRRX1 | na |  |  | 19045 | -2.882 | -0.0650 | Yes |
| 148 | DPYSL3 | na |  |  | 19076 | -2.970 | -0.0475 | Yes |
| 149 | SAT1 | na |  |  | 19139 | -3.506 | -0.0281 | Yes |
| 150 | PRSS2 | na |  |  | 19188 | -4.840 | 0.0005 | Yes |
Table: GSEA details [plain text format]

  

Fig 2: HALLMARK\_EPITHELIAL\_MESENCHYMAL\_TRANSITION      
 Blue-Pink O' Gram in the Space of the Analyzed GeneSet

  

Fig 3: HALLMARK\_EPITHELIAL\_MESENCHYMAL\_TRANSITION: Random ES distribution      
 Gene set null distribution of ES for **HALLMARK\_EPITHELIAL\_MESENCHYMAL\_TRANSITION**

  
