# Supplementary material for: Heparin-based hydrogel scaffolding alters the transcriptomic profile and increases the chemoresistance of MDA-MB-231 triple-negative breast cancer cells
Source: Biomater Sci. 2020 Feb 13;8(10):2786–96. doi: 10.1039/c9bm01481k (PMC7497406; doi:10.1039/c9bm01481k)
Supplement: Supplementary file 2 [file BM-008-C9BM01481K-s002.zip › Supplementary File 4/EGFvControl/Pathways/my_analysis.Gsea.1545200981068/HALLMARK_NOTCH_SIGNALING.html]

Details for gene set HALLMARK\_NOTCH\_SIGNALING[GSEA]

|  || Dataset | expr.class.cls#EGF\_versus\_CONTROL.class.cls#EGF\_versus\_CONTROL\_repos |
| Phenotype | class.cls#EGF\_versus\_CONTROL\_repos |
| Upregulated in class | CONTROL |
| GeneSet | HALLMARK\_NOTCH\_SIGNALING |
| Enrichment Score (ES) | -0.3219933 |
| Normalized Enrichment Score (NES) | -1.0870751 |
| Nominal p-value | 0.323185 |
| FDR q-value | 0.3152848 |
| FWER p-Value | 0.996 |
Table: GSEA Results Summary

  

Fig 1: Enrichment plot: HALLMARK\_NOTCH\_SIGNALING      
 Profile of the Running ES Score & Positions of GeneSet Members on the Rank Ordered List

  

| PROBE | DESCRIPTION (from dataset) | GENE SYMBOL | GENE\_TITLE | RANK IN GENE LIST | RANK METRIC SCORE | RUNNING ES | CORE ENRICHMENT || 1 | CUL1 | na |  |  | 1288 | 1.415 | 0.0020 | No |
| 2 | CCND1 | na |  |  | 2400 | 1.133 | -0.0006 | No |
| 3 | FBXW11 | na |  |  | 4266 | 0.813 | -0.0582 | No |
| 4 | PRKCA | na |  |  | 4313 | 0.806 | -0.0212 | No |
| 5 | FZD7 | na |  |  | 4446 | 0.786 | 0.0103 | No |
| 6 | FZD5 | na |  |  | 4494 | 0.779 | 0.0459 | No |
| 7 | PSENEN | na |  |  | 5065 | 0.698 | 0.0503 | No |
| 8 | SKP1 | na |  |  | 5694 | 0.610 | 0.0473 | No |
| 9 | NOTCH2 | na |  |  | 6144 | 0.549 | 0.0507 | No |
| 10 | RBX1 | na |  |  | 6955 | 0.450 | 0.0305 | No |
| 11 | APH1A | na |  |  | 7583 | 0.375 | 0.0161 | No |
| 12 | DTX4 | na |  |  | 7646 | 0.369 | 0.0308 | No |
| 13 | ST3GAL6 | na |  |  | 11301 | -0.040 | -0.1578 | No |
| 14 | PSEN2 | na |  |  | 11451 | -0.056 | -0.1628 | No |
| 15 | HES1 | na |  |  | 11615 | -0.072 | -0.1678 | No |
| 16 | DTX2 | na |  |  | 12301 | -0.155 | -0.1959 | No |
| 17 | SAP30 | na |  |  | 12691 | -0.212 | -0.2059 | No |
| 18 | PPARD | na |  |  | 12731 | -0.219 | -0.1972 | No |
| 19 | NOTCH1 | na |  |  | 13411 | -0.304 | -0.2178 | No |
| 20 | KAT2A | na |  |  | 13640 | -0.339 | -0.2132 | No |
| 21 | JAG1 | na |  |  | 15596 | -0.604 | -0.2856 | Yes |
| 22 | FZD1 | na |  |  | 15971 | -0.681 | -0.2719 | Yes |
| 23 | MAML2 | na |  |  | 16933 | -0.932 | -0.2765 | Yes |
| 24 | TCF7L2 | na |  |  | 17148 | -0.994 | -0.2391 | Yes |
| 25 | ARRB1 | na |  |  | 17629 | -1.161 | -0.2074 | Yes |
| 26 | WNT5A | na |  |  | 18031 | -1.345 | -0.1626 | Yes |
| 27 | LFNG | na |  |  | 18494 | -1.637 | -0.1067 | Yes |
| 28 | NOTCH3 | na |  |  | 19065 | -2.934 | 0.0069 | Yes |
Table: GSEA details [plain text format]

  

Fig 2: HALLMARK\_NOTCH\_SIGNALING      
 Blue-Pink O' Gram in the Space of the Analyzed GeneSet

  

Fig 3: HALLMARK\_NOTCH\_SIGNALING: Random ES distribution      
 Gene set null distribution of ES for **HALLMARK\_NOTCH\_SIGNALING**

  
