# Supplementary material for: Heparin-based hydrogel scaffolding alters the transcriptomic profile and increases the chemoresistance of MDA-MB-231 triple-negative breast cancer cells
Source: Biomater Sci. 2020 Feb 13;8(10):2786–96. doi: 10.1039/c9bm01481k (PMC7497406; doi:10.1039/c9bm01481k)
Supplement: Supplementary file 2 [file BM-008-C9BM01481K-s002.zip › Supplementary File 4/EGFvControl/Pathways/my_analysis.Gsea.1545200981068/HALLMARK_WNT_BETA_CATENIN_SIGNALING.html]

Details for gene set HALLMARK\_WNT\_BETA\_CATENIN\_SIGNALING[GSEA]

|  || Dataset | expr.class.cls#EGF\_versus\_CONTROL.class.cls#EGF\_versus\_CONTROL\_repos |
| Phenotype | class.cls#EGF\_versus\_CONTROL\_repos |
| Upregulated in class | CONTROL |
| GeneSet | HALLMARK\_WNT\_BETA\_CATENIN\_SIGNALING |
| Enrichment Score (ES) | -0.2229465 |
| Normalized Enrichment Score (NES) | -0.80150336 |
| Nominal p-value | 0.7790974 |
| FDR q-value | 0.8915818 |
| FWER p-Value | 1.0 |
Table: GSEA Results Summary

  

Fig 1: Enrichment plot: HALLMARK\_WNT\_BETA\_CATENIN\_SIGNALING      
 Profile of the Running ES Score & Positions of GeneSet Members on the Rank Ordered List

  

| PROBE | DESCRIPTION (from dataset) | GENE SYMBOL | GENE\_TITLE | RANK IN GENE LIST | RANK METRIC SCORE | RUNNING ES | CORE ENRICHMENT || 1 | DKK1 | na |  |  | 418 | 1.831 | 0.0470 | Yes |
| 2 | WNT5B | na |  |  | 675 | 1.658 | 0.0959 | Yes |
| 3 | CUL1 | na |  |  | 1288 | 1.415 | 0.1171 | Yes |
| 4 | SKP2 | na |  |  | 1581 | 1.324 | 0.1515 | Yes |
| 5 | LEF1 | na |  |  | 1666 | 1.304 | 0.1961 | Yes |
| 6 | HDAC2 | na |  |  | 2599 | 1.094 | 0.1886 | Yes |
| 7 | GNAI1 | na |  |  | 3405 | 0.953 | 0.1823 | Yes |
| 8 | DVL2 | na |  |  | 3725 | 0.900 | 0.1995 | Yes |
| 9 | MYC | na |  |  | 3907 | 0.866 | 0.2226 | Yes |
| 10 | MAML1 | na |  |  | 5610 | 0.624 | 0.1572 | No |
| 11 | AXIN1 | na |  |  | 8255 | 0.298 | 0.0304 | No |
| 12 | RBPJ | na |  |  | 8652 | 0.254 | 0.0193 | No |
| 13 | PTCH1 | na |  |  | 10340 | 0.063 | -0.0664 | No |
| 14 | ADAM17 | na |  |  | 10944 | 0.003 | -0.0978 | No |
| 15 | PSEN2 | na |  |  | 11451 | -0.056 | -0.1221 | No |
| 16 | TCF7 | na |  |  | 12167 | -0.139 | -0.1542 | No |
| 17 | PPARD | na |  |  | 12731 | -0.219 | -0.1753 | No |
| 18 | CTNNB1 | na |  |  | 13218 | -0.277 | -0.1903 | No |
| 19 | NOTCH1 | na |  |  | 13411 | -0.304 | -0.1889 | No |
| 20 | KAT2A | na |  |  | 13640 | -0.339 | -0.1881 | No |
| 21 | TP53 | na |  |  | 14267 | -0.417 | -0.2051 | No |
| 22 | HDAC11 | na |  |  | 14604 | -0.464 | -0.2052 | No |
| 23 | NCOR2 | na |  |  | 14701 | -0.480 | -0.1922 | No |
| 24 | JAG2 | na |  |  | 15291 | -0.563 | -0.2018 | No |
| 25 | JAG1 | na |  |  | 15596 | -0.604 | -0.1950 | No |
| 26 | FZD1 | na |  |  | 15971 | -0.681 | -0.1889 | No |
| 27 | NOTCH4 | na |  |  | 16533 | -0.819 | -0.1875 | No |
| 28 | HDAC5 | na |  |  | 16964 | -0.940 | -0.1746 | No |
| 29 | NUMB | na |  |  | 16985 | -0.948 | -0.1400 | No |
| 30 | FRAT1 | na |  |  | 17269 | -1.029 | -0.1162 | No |
| 31 | CCND2 | na |  |  | 17385 | -1.070 | -0.0820 | No |
| 32 | CSNK1E | na |  |  | 17497 | -1.122 | -0.0457 | No |
| 33 | NCSTN | na |  |  | 17508 | -1.125 | -0.0039 | No |
| 34 | FZD8 | na |  |  | 17554 | -1.133 | 0.0363 | No |
| 35 | AXIN2 | na |  |  | 17977 | -1.317 | 0.0637 | No |
Table: GSEA details [plain text format]

  

Fig 2: HALLMARK\_WNT\_BETA\_CATENIN\_SIGNALING      
 Blue-Pink O' Gram in the Space of the Analyzed GeneSet

  

Fig 3: HALLMARK\_WNT\_BETA\_CATENIN\_SIGNALING: Random ES distribution      
 Gene set null distribution of ES for **HALLMARK\_WNT\_BETA\_CATENIN\_SIGNALING**

  
