# Supplementary material for: Heparin-based hydrogel scaffolding alters the transcriptomic profile and increases the chemoresistance of MDA-MB-231 triple-negative breast cancer cells
Source: Biomater Sci. 2020 Feb 13;8(10):2786–96. doi: 10.1039/c9bm01481k (PMC7497406; doi:10.1039/c9bm01481k)
Supplement: Supplementary file 2 [file BM-008-C9BM01481K-s002.zip › Supplementary File 4/EGFvControl/Pathways/my_analysis.Gsea.1545200981068/HALLMARK_G2M_CHECKPOINT.html]

Details for gene set HALLMARK\_G2M\_CHECKPOINT[GSEA]

|  || Dataset | expr.class.cls#EGF\_versus\_CONTROL.class.cls#EGF\_versus\_CONTROL\_repos |
| Phenotype | class.cls#EGF\_versus\_CONTROL\_repos |
| Upregulated in class | EGF |
| GeneSet | HALLMARK\_G2M\_CHECKPOINT |
| Enrichment Score (ES) | 0.6168415 |
| Normalized Enrichment Score (NES) | 2.8949919 |
| Nominal p-value | 0.0 |
| FDR q-value | 0.0 |
| FWER p-Value | 0.0 |
Table: GSEA Results Summary

  

Fig 1: Enrichment plot: HALLMARK\_G2M\_CHECKPOINT      
 Profile of the Running ES Score & Positions of GeneSet Members on the Rank Ordered List

  

| PROBE | DESCRIPTION (from dataset) | GENE SYMBOL | GENE\_TITLE | RANK IN GENE LIST | RANK METRIC SCORE | RUNNING ES | CORE ENRICHMENT || 1 | CDC25A | na |  |  | 107 | 2.312 | 0.0060 | Yes |
| 2 | AURKA | na |  |  | 163 | 2.181 | 0.0141 | Yes |
| 3 | UCK2 | na |  |  | 168 | 2.173 | 0.0249 | Yes |
| 4 | SLC7A5 | na |  |  | 213 | 2.074 | 0.0330 | Yes |
| 5 | EZH2 | na |  |  | 227 | 2.053 | 0.0426 | Yes |
| 6 | SLC7A1 | na |  |  | 311 | 1.936 | 0.0480 | Yes |
| 7 | AURKB | na |  |  | 328 | 1.916 | 0.0568 | Yes |
| 8 | ODC1 | na |  |  | 415 | 1.835 | 0.0616 | Yes |
| 9 | NOLC1 | na |  |  | 443 | 1.809 | 0.0693 | Yes |
| 10 | BUB1 | na |  |  | 455 | 1.797 | 0.0777 | Yes |
| 11 | CDC6 | na |  |  | 472 | 1.786 | 0.0859 | Yes |
| 12 | CDKN3 | na |  |  | 562 | 1.729 | 0.0899 | Yes |
| 13 | CDC20 | na |  |  | 590 | 1.712 | 0.0971 | Yes |
| 14 | GINS2 | na |  |  | 593 | 1.709 | 0.1056 | Yes |
| 15 | SUV39H1 | na |  |  | 628 | 1.686 | 0.1123 | Yes |
| 16 | CDKN2C | na |  |  | 635 | 1.679 | 0.1205 | Yes |
| 17 | PLK4 | na |  |  | 650 | 1.670 | 0.1281 | Yes |
| 18 | CCNA2 | na |  |  | 725 | 1.626 | 0.1324 | Yes |
| 19 | DDX39A | na |  |  | 731 | 1.621 | 0.1403 | Yes |
| 20 | POLE | na |  |  | 760 | 1.605 | 0.1470 | Yes |
| 21 | POLQ | na |  |  | 792 | 1.594 | 0.1534 | Yes |
| 22 | CDC45 | na |  |  | 795 | 1.593 | 0.1613 | Yes |
| 23 | WRN | na |  |  | 815 | 1.583 | 0.1683 | Yes |
| 24 | PRIM2 | na |  |  | 835 | 1.575 | 0.1752 | Yes |
| 25 | SRSF1 | na |  |  | 863 | 1.566 | 0.1817 | Yes |
| 26 | NUP50 | na |  |  | 877 | 1.561 | 0.1888 | Yes |
| 27 | MCM2 | na |  |  | 906 | 1.550 | 0.1952 | Yes |
| 28 | TPX2 | na |  |  | 997 | 1.516 | 0.1981 | Yes |
| 29 | CDC7 | na |  |  | 1001 | 1.514 | 0.2055 | Yes |
| 30 | PRC1 | na |  |  | 1054 | 1.491 | 0.2103 | Yes |
| 31 | PLK1 | na |  |  | 1074 | 1.488 | 0.2168 | Yes |
| 32 | KIF15 | na |  |  | 1127 | 1.469 | 0.2215 | Yes |
| 33 | HMGN2 | na |  |  | 1134 | 1.466 | 0.2285 | Yes |
| 34 | DKC1 | na |  |  | 1144 | 1.464 | 0.2354 | Yes |
| 35 | KIF23 | na |  |  | 1186 | 1.445 | 0.2406 | Yes |
| 36 | TACC3 | na |  |  | 1207 | 1.439 | 0.2468 | Yes |
| 37 | RBM14 | na |  |  | 1263 | 1.424 | 0.2510 | Yes |
| 38 | DTYMK | na |  |  | 1286 | 1.417 | 0.2570 | Yes |
| 39 | CUL1 | na |  |  | 1288 | 1.415 | 0.2641 | Yes |
| 40 | RACGAP1 | na |  |  | 1310 | 1.409 | 0.2701 | Yes |
| 41 | BRCA2 | na |  |  | 1333 | 1.399 | 0.2760 | Yes |
| 42 | KIF11 | na |  |  | 1334 | 1.399 | 0.2830 | Yes |
| 43 | E2F1 | na |  |  | 1340 | 1.395 | 0.2898 | Yes |
| 44 | CCNF | na |  |  | 1350 | 1.393 | 0.2963 | Yes |
| 45 | HMMR | na |  |  | 1351 | 1.393 | 0.3034 | Yes |
| 46 | PRMT5 | na |  |  | 1360 | 1.391 | 0.3099 | Yes |
| 47 | TFDP1 | na |  |  | 1387 | 1.381 | 0.3155 | Yes |
| 48 | SMC4 | na |  |  | 1418 | 1.372 | 0.3209 | Yes |
| 49 | TRA2B | na |  |  | 1432 | 1.369 | 0.3271 | Yes |
| 50 | LMNB1 | na |  |  | 1433 | 1.368 | 0.3340 | Yes |
| 51 | BIRC5 | na |  |  | 1448 | 1.364 | 0.3401 | Yes |
| 52 | E2F2 | na |  |  | 1450 | 1.364 | 0.3469 | Yes |
| 53 | RBL1 | na |  |  | 1513 | 1.342 | 0.3504 | Yes |
| 54 | NUSAP1 | na |  |  | 1541 | 1.335 | 0.3557 | Yes |
| 55 | MCM6 | na |  |  | 1672 | 1.301 | 0.3554 | Yes |
| 56 | SLC12A2 | na |  |  | 1686 | 1.297 | 0.3613 | Yes |
| 57 | MAD2L1 | na |  |  | 1695 | 1.296 | 0.3674 | Yes |
| 58 | KIF4A | na |  |  | 1715 | 1.291 | 0.3729 | Yes |
| 59 | E2F3 | na |  |  | 1781 | 1.273 | 0.3759 | Yes |
| 60 | RASAL2 | na |  |  | 1823 | 1.262 | 0.3801 | Yes |
| 61 | SRSF2 | na |  |  | 1835 | 1.260 | 0.3859 | Yes |
| 62 | XPO1 | na |  |  | 1845 | 1.259 | 0.3917 | Yes |
| 63 | KPNA2 | na |  |  | 1925 | 1.241 | 0.3938 | Yes |
| 64 | LBR | na |  |  | 1968 | 1.230 | 0.3978 | Yes |
| 65 | EXO1 | na |  |  | 1972 | 1.227 | 0.4038 | Yes |
| 66 | KIF2C | na |  |  | 1975 | 1.227 | 0.4099 | Yes |
| 67 | CENPF | na |  |  | 1986 | 1.224 | 0.4156 | Yes |
| 68 | CBX1 | na |  |  | 2000 | 1.220 | 0.4210 | Yes |
| 69 | KIF20B | na |  |  | 2009 | 1.217 | 0.4267 | Yes |
| 70 | NUP98 | na |  |  | 2025 | 1.214 | 0.4321 | Yes |
| 71 | NDC80 | na |  |  | 2032 | 1.212 | 0.4378 | Yes |
| 72 | STAG1 | na |  |  | 2035 | 1.212 | 0.4438 | Yes |
| 73 | BARD1 | na |  |  | 2041 | 1.211 | 0.4497 | Yes |
| 74 | CKS1B | na |  |  | 2048 | 1.207 | 0.4554 | Yes |
| 75 | SMC2 | na |  |  | 2050 | 1.207 | 0.4615 | Yes |
| 76 | TMPO | na |  |  | 2091 | 1.199 | 0.4654 | Yes |
| 77 | TOP2A | na |  |  | 2151 | 1.189 | 0.4683 | Yes |
| 78 | STIL | na |  |  | 2163 | 1.186 | 0.4737 | Yes |
| 79 | MCM3 | na |  |  | 2175 | 1.183 | 0.4791 | Yes |
| 80 | CENPE | na |  |  | 2189 | 1.181 | 0.4843 | Yes |
| 81 | SNRPD1 | na |  |  | 2237 | 1.171 | 0.4878 | Yes |
| 82 | CUL4A | na |  |  | 2245 | 1.168 | 0.4933 | Yes |
| 83 | KPNB1 | na |  |  | 2253 | 1.166 | 0.4988 | Yes |
| 84 | BUB3 | na |  |  | 2328 | 1.149 | 0.5007 | Yes |
| 85 | DR1 | na |  |  | 2339 | 1.147 | 0.5059 | Yes |
| 86 | ORC6 | na |  |  | 2344 | 1.146 | 0.5115 | Yes |
| 87 | CHAF1A | na |  |  | 2367 | 1.140 | 0.5161 | Yes |
| 88 | FBXO5 | na |  |  | 2371 | 1.139 | 0.5217 | Yes |
| 89 | CCND1 | na |  |  | 2400 | 1.133 | 0.5259 | Yes |
| 90 | KIF22 | na |  |  | 2457 | 1.122 | 0.5286 | Yes |
| 91 | POLA2 | na |  |  | 2529 | 1.105 | 0.5304 | Yes |
| 92 | ESPL1 | na |  |  | 2577 | 1.097 | 0.5335 | Yes |
| 93 | DBF4 | na |  |  | 2587 | 1.096 | 0.5385 | Yes |
| 94 | UBE2S | na |  |  | 2671 | 1.081 | 0.5396 | Yes |
| 95 | AMD1 | na |  |  | 2680 | 1.080 | 0.5446 | Yes |
| 96 | RAD54L | na |  |  | 2696 | 1.077 | 0.5493 | Yes |
| 97 | G3BP1 | na |  |  | 2756 | 1.068 | 0.5515 | Yes |
| 98 | SRSF10 | na |  |  | 2782 | 1.063 | 0.5556 | Yes |
| 99 | CDK1 | na |  |  | 2921 | 1.040 | 0.5536 | Yes |
| 100 | CHEK1 | na |  |  | 2954 | 1.034 | 0.5571 | Yes |
| 101 | RAD23B | na |  |  | 2977 | 1.030 | 0.5611 | Yes |
| 102 | PBK | na |  |  | 3038 | 1.019 | 0.5631 | Yes |
| 103 | E2F4 | na |  |  | 3090 | 1.008 | 0.5655 | Yes |
| 104 | MYBL2 | na |  |  | 3098 | 1.007 | 0.5702 | Yes |
| 105 | RPA2 | na |  |  | 3128 | 1.001 | 0.5737 | Yes |
| 106 | KIF5B | na |  |  | 3136 | 1.000 | 0.5784 | Yes |
| 107 | MAPK14 | na |  |  | 3187 | 0.990 | 0.5807 | Yes |
| 108 | MCM5 | na |  |  | 3202 | 0.986 | 0.5850 | Yes |
| 109 | NEK2 | na |  |  | 3222 | 0.981 | 0.5889 | Yes |
| 110 | TTK | na |  |  | 3293 | 0.971 | 0.5901 | Yes |
| 111 | TRAIP | na |  |  | 3411 | 0.951 | 0.5888 | Yes |
| 112 | SYNCRIP | na |  |  | 3486 | 0.939 | 0.5896 | Yes |
| 113 | GSPT1 | na |  |  | 3557 | 0.928 | 0.5906 | Yes |
| 114 | NASP | na |  |  | 3598 | 0.921 | 0.5931 | Yes |
| 115 | SMC1A | na |  |  | 3638 | 0.915 | 0.5957 | Yes |
| 116 | NCL | na |  |  | 3900 | 0.867 | 0.5863 | Yes |
| 117 | MYC | na |  |  | 3907 | 0.866 | 0.5904 | Yes |
| 118 | MNAT1 | na |  |  | 4011 | 0.849 | 0.5892 | Yes |
| 119 | H2AFZ | na |  |  | 4017 | 0.848 | 0.5932 | Yes |
| 120 | TROAP | na |  |  | 4037 | 0.845 | 0.5965 | Yes |
| 121 | RAD21 | na |  |  | 4041 | 0.844 | 0.6006 | Yes |
| 122 | H2AFV | na |  |  | 4185 | 0.824 | 0.5972 | Yes |
| 123 | ORC5 | na |  |  | 4298 | 0.808 | 0.5954 | Yes |
| 124 | FANCC | na |  |  | 4416 | 0.789 | 0.5932 | Yes |
| 125 | SFPQ | na |  |  | 4431 | 0.788 | 0.5965 | Yes |
| 126 | MTF2 | na |  |  | 4447 | 0.786 | 0.5996 | Yes |
| 127 | CENPA | na |  |  | 4451 | 0.785 | 0.6034 | Yes |
| 128 | KATNA1 | na |  |  | 4477 | 0.780 | 0.6060 | Yes |
| 129 | PAPD7 | na |  |  | 4532 | 0.773 | 0.6071 | Yes |
| 130 | YTHDC1 | na |  |  | 4581 | 0.766 | 0.6084 | Yes |
| 131 | UBE2C | na |  |  | 4657 | 0.755 | 0.6083 | Yes |
| 132 | UPF1 | na |  |  | 4673 | 0.753 | 0.6113 | Yes |
| 133 | STMN1 | na |  |  | 4739 | 0.740 | 0.6116 | Yes |
| 134 | CDK4 | na |  |  | 4801 | 0.730 | 0.6121 | Yes |
| 135 | ILF3 | na |  |  | 4902 | 0.718 | 0.6104 | Yes |
| 136 | SLC38A1 | na |  |  | 4939 | 0.715 | 0.6121 | Yes |
| 137 | PDS5B | na |  |  | 4949 | 0.714 | 0.6153 | Yes |
| 138 | CASP8AP2 | na |  |  | 5043 | 0.702 | 0.6139 | Yes |
| 139 | PTTG1 | na |  |  | 5055 | 0.700 | 0.6168 | Yes |
| 140 | CCNB2 | na |  |  | 5148 | 0.685 | 0.6155 | No |
| 141 | HNRNPU | na |  |  | 5265 | 0.670 | 0.6127 | No |
| 142 | PURA | na |  |  | 5295 | 0.665 | 0.6145 | No |
| 143 | CTCF | na |  |  | 5690 | 0.611 | 0.5969 | No |
| 144 | CUL3 | na |  |  | 5854 | 0.591 | 0.5913 | No |
| 145 | NOTCH2 | na |  |  | 6144 | 0.549 | 0.5789 | No |
| 146 | H2AFX | na |  |  | 6160 | 0.547 | 0.5808 | No |
| 147 | CKS2 | na |  |  | 6345 | 0.522 | 0.5738 | No |
| 148 | LIG3 | na |  |  | 6372 | 0.517 | 0.5750 | No |
| 149 | EGF | na |  |  | 6597 | 0.493 | 0.5657 | No |
| 150 | SS18 | na |  |  | 6739 | 0.475 | 0.5607 | No |
| 151 | PRPF4B | na |  |  | 6789 | 0.469 | 0.5605 | No |
| 152 | ATF5 | na |  |  | 6791 | 0.469 | 0.5628 | No |
| 153 | FOXN3 | na |  |  | 7322 | 0.406 | 0.5369 | No |
| 154 | ODF2 | na |  |  | 7370 | 0.399 | 0.5365 | No |
| 155 | RPS6KA5 | na |  |  | 7480 | 0.388 | 0.5327 | No |
| 156 | HNRNPD | na |  |  | 7535 | 0.381 | 0.5318 | No |
| 157 | CUL5 | na |  |  | 7547 | 0.379 | 0.5331 | No |
| 158 | CDC27 | na |  |  | 7997 | 0.325 | 0.5111 | No |
| 159 | EWSR1 | na |  |  | 8059 | 0.319 | 0.5095 | No |
| 160 | PAFAH1B1 | na |  |  | 8291 | 0.294 | 0.4989 | No |
| 161 | CCNT1 | na |  |  | 9070 | 0.205 | 0.4590 | No |
| 162 | MEIS2 | na |  |  | 9256 | 0.187 | 0.4502 | No |
| 163 | INCENP | na |  |  | 9447 | 0.164 | 0.4410 | No |
| 164 | HIRA | na |  |  | 10115 | 0.094 | 0.4064 | No |
| 165 | SMARCC1 | na |  |  | 10226 | 0.078 | 0.4010 | No |
| 166 | TNPO2 | na |  |  | 10730 | 0.021 | 0.3746 | No |
| 167 | CHMP1A | na |  |  | 10950 | 0.002 | 0.3631 | No |
| 168 | HIF1A | na |  |  | 11136 | -0.018 | 0.3535 | No |
| 169 | SQLE | na |  |  | 11156 | -0.021 | 0.3526 | No |
| 170 | ABL1 | na |  |  | 11459 | -0.057 | 0.3370 | No |
| 171 | TOP1 | na |  |  | 11743 | -0.091 | 0.3226 | No |
| 172 | MEIS1 | na |  |  | 11987 | -0.124 | 0.3104 | No |
| 173 | CDC25B | na |  |  | 12061 | -0.134 | 0.3072 | No |
| 174 | HUS1 | na |  |  | 12612 | -0.201 | 0.2793 | No |
| 175 | SAP30 | na |  |  | 12691 | -0.212 | 0.2763 | No |
| 176 | CDKN1B | na |  |  | 13864 | -0.360 | 0.2164 | No |
| 177 | ATRX | na |  |  | 13969 | -0.372 | 0.2129 | No |
| 178 | ARID4A | na |  |  | 14716 | -0.483 | 0.1760 | No |
| 179 | MT2A | na |  |  | 14804 | -0.498 | 0.1740 | No |
| 180 | HIST1H2BK | na |  |  | 14950 | -0.507 | 0.1689 | No |
| 181 | SMAD3 | na |  |  | 15810 | -0.650 | 0.1270 | No |
| 182 | PML | na |  |  | 15940 | -0.675 | 0.1236 | No |
| 183 | TLE3 | na |  |  | 15944 | -0.676 | 0.1268 | No |
| 184 | EFNA5 | na |  |  | 16633 | -0.841 | 0.0949 | No |
| 185 | NUMA1 | na |  |  | 17337 | -1.054 | 0.0632 | No |
| 186 | TGFB1 | na |  |  | 17599 | -1.148 | 0.0553 | No |
| 187 | MARCKS | na |  |  | 17808 | -1.228 | 0.0505 | No |
| 188 | DMD | na |  |  | 17999 | -1.329 | 0.0472 | No |
| 189 | BCL3 | na |  |  | 18068 | -1.364 | 0.0505 | No |
| 190 | HMGB3 | na |  |  | 18612 | -1.764 | 0.0308 | No |
Table: GSEA details [plain text format]

  

Fig 2: HALLMARK\_G2M\_CHECKPOINT      
 Blue-Pink O' Gram in the Space of the Analyzed GeneSet

  

Fig 3: HALLMARK\_G2M\_CHECKPOINT: Random ES distribution      
 Gene set null distribution of ES for **HALLMARK\_G2M\_CHECKPOINT**

  
