# Supplementary material for: Heparin-based hydrogel scaffolding alters the transcriptomic profile and increases the chemoresistance of MDA-MB-231 triple-negative breast cancer cells
Source: Biomater Sci. 2020 Feb 13;8(10):2786–96. doi: 10.1039/c9bm01481k (PMC7497406; doi:10.1039/c9bm01481k)
Supplement: Supplementary file 2 [file BM-008-C9BM01481K-s002.zip › Supplementary File 4/EGFvControl/Pathways/my_analysis.Gsea.1545200981068/HALLMARK_UV_RESPONSE_UP.html]

Details for gene set HALLMARK\_UV\_RESPONSE\_UP[GSEA]

|  || Dataset | expr.class.cls#EGF\_versus\_CONTROL.class.cls#EGF\_versus\_CONTROL\_repos |
| Phenotype | class.cls#EGF\_versus\_CONTROL\_repos |
| Upregulated in class | EGF |
| GeneSet | HALLMARK\_UV\_RESPONSE\_UP |
| Enrichment Score (ES) | 0.27666998 |
| Normalized Enrichment Score (NES) | 1.228648 |
| Nominal p-value | 0.1 |
| FDR q-value | 0.17336527 |
| FWER p-Value | 0.982 |
Table: GSEA Results Summary

  

Fig 1: Enrichment plot: HALLMARK\_UV\_RESPONSE\_UP      
 Profile of the Running ES Score & Positions of GeneSet Members on the Rank Ordered List

  

| PROBE | DESCRIPTION (from dataset) | GENE SYMBOL | GENE\_TITLE | RANK IN GENE LIST | RANK METRIC SCORE | RUNNING ES | CORE ENRICHMENT || 1 | CYP1A1 | na |  |  | 96 | 2.351 | 0.0159 | Yes |
| 2 | FEN1 | na |  |  | 251 | 2.028 | 0.0258 | Yes |
| 3 | CYB5B | na |  |  | 290 | 1.967 | 0.0413 | Yes |
| 4 | TFRC | na |  |  | 359 | 1.882 | 0.0545 | Yes |
| 5 | PPT1 | na |  |  | 379 | 1.863 | 0.0701 | Yes |
| 6 | CCNE1 | na |  |  | 408 | 1.841 | 0.0850 | Yes |
| 7 | C4BPB | na |  |  | 508 | 1.768 | 0.0955 | Yes |
| 8 | TUBA4A | na |  |  | 624 | 1.689 | 0.1045 | Yes |
| 9 | DDX21 | na |  |  | 775 | 1.599 | 0.1108 | Yes |
| 10 | FKBP4 | na |  |  | 865 | 1.565 | 0.1201 | Yes |
| 11 | CASP3 | na |  |  | 1155 | 1.458 | 0.1179 | Yes |
| 12 | POLE3 | na |  |  | 1179 | 1.447 | 0.1296 | Yes |
| 13 | TYRO3 | na |  |  | 1408 | 1.374 | 0.1298 | Yes |
| 14 | RFC4 | na |  |  | 1417 | 1.372 | 0.1416 | Yes |
| 15 | GLS | na |  |  | 1485 | 1.350 | 0.1501 | Yes |
| 16 | PPIF | na |  |  | 1496 | 1.347 | 0.1615 | Yes |
| 17 | TARS | na |  |  | 1506 | 1.343 | 0.1730 | Yes |
| 18 | NAT1 | na |  |  | 1522 | 1.340 | 0.1841 | Yes |
| 19 | SIGMAR1 | na |  |  | 1591 | 1.321 | 0.1923 | Yes |
| 20 | PARP2 | na |  |  | 1635 | 1.310 | 0.2017 | Yes |
| 21 | RASGRP1 | na |  |  | 1769 | 1.276 | 0.2061 | Yes |
| 22 | PPAT | na |  |  | 1786 | 1.272 | 0.2166 | Yes |
| 23 | CEBPG | na |  |  | 1827 | 1.261 | 0.2257 | Yes |
| 24 | SPR | na |  |  | 2096 | 1.198 | 0.2223 | Yes |
| 25 | CCND3 | na |  |  | 2241 | 1.169 | 0.2251 | Yes |
| 26 | CHKA | na |  |  | 2251 | 1.166 | 0.2350 | Yes |
| 27 | STIP1 | na |  |  | 2297 | 1.157 | 0.2429 | Yes |
| 28 | CDK2 | na |  |  | 2311 | 1.153 | 0.2525 | Yes |
| 29 | CYB5R1 | na |  |  | 2601 | 1.093 | 0.2471 | Yes |
| 30 | AMD1 | na |  |  | 2680 | 1.080 | 0.2526 | Yes |
| 31 | HMOX1 | na |  |  | 3011 | 1.026 | 0.2444 | Yes |
| 32 | PPP1R2 | na |  |  | 3049 | 1.016 | 0.2515 | Yes |
| 33 | GRPEL1 | na |  |  | 3510 | 0.935 | 0.2357 | Yes |
| 34 | EIF2S3 | na |  |  | 3771 | 0.890 | 0.2300 | Yes |
| 35 | AP2S1 | na |  |  | 3824 | 0.880 | 0.2351 | Yes |
| 36 | EIF5 | na |  |  | 3879 | 0.872 | 0.2400 | Yes |
| 37 | DNAJA1 | na |  |  | 3884 | 0.871 | 0.2475 | Yes |
| 38 | ARRB2 | na |  |  | 3886 | 0.870 | 0.2552 | Yes |
| 39 | CDC5L | na |  |  | 3924 | 0.864 | 0.2609 | Yes |
| 40 | BTG3 | na |  |  | 3985 | 0.853 | 0.2654 | Yes |
| 41 | TGFBRAP1 | na |  |  | 4109 | 0.835 | 0.2664 | Yes |
| 42 | CLTB | na |  |  | 4410 | 0.790 | 0.2576 | Yes |
| 43 | SLC25A4 | na |  |  | 4552 | 0.770 | 0.2571 | Yes |
| 44 | PSMC3 | na |  |  | 4703 | 0.748 | 0.2559 | Yes |
| 45 | YKT6 | na |  |  | 4749 | 0.739 | 0.2601 | Yes |
| 46 | NPTXR | na |  |  | 4772 | 0.735 | 0.2655 | Yes |
| 47 | GGH | na |  |  | 5069 | 0.698 | 0.2562 | Yes |
| 48 | PRPF3 | na |  |  | 5083 | 0.696 | 0.2617 | Yes |
| 49 | ATP6V1F | na |  |  | 5085 | 0.696 | 0.2678 | Yes |
| 50 | ACAA1 | na |  |  | 5086 | 0.695 | 0.2740 | Yes |
| 51 | FOSB | na |  |  | 5209 | 0.678 | 0.2736 | Yes |
| 52 | HNRNPU | na |  |  | 5265 | 0.670 | 0.2767 | Yes |
| 53 | CLCN2 | na |  |  | 5457 | 0.643 | 0.2724 | No |
| 54 | CHRNA5 | na |  |  | 5953 | 0.576 | 0.2515 | No |
| 55 | H2AFX | na |  |  | 6160 | 0.547 | 0.2456 | No |
| 56 | FURIN | na |  |  | 6470 | 0.506 | 0.2339 | No |
| 57 | MARK2 | na |  |  | 6654 | 0.485 | 0.2286 | No |
| 58 | GCH1 | na |  |  | 6881 | 0.459 | 0.2208 | No |
| 59 | ATF3 | na |  |  | 7410 | 0.395 | 0.1967 | No |
| 60 | EPCAM | na |  |  | 7501 | 0.385 | 0.1954 | No |
| 61 | TMBIM6 | na |  |  | 7503 | 0.385 | 0.1987 | No |
| 62 | LYN | na |  |  | 7669 | 0.366 | 0.1933 | No |
| 63 | UROD | na |  |  | 7917 | 0.336 | 0.1834 | No |
| 64 | NXF1 | na |  |  | 8013 | 0.323 | 0.1813 | No |
| 65 | POLG2 | na |  |  | 8033 | 0.322 | 0.1831 | No |
| 66 | PDLIM3 | na |  |  | 8401 | 0.284 | 0.1664 | No |
| 67 | MAOA | na |  |  | 8409 | 0.283 | 0.1685 | No |
| 68 | ATP6V1C1 | na |  |  | 8435 | 0.280 | 0.1697 | No |
| 69 | MGAT1 | na |  |  | 8501 | 0.272 | 0.1687 | No |
| 70 | HTR7 | na |  |  | 8579 | 0.264 | 0.1670 | No |
| 71 | HSPA2 | na |  |  | 9174 | 0.194 | 0.1376 | No |
| 72 | POLR2H | na |  |  | 9188 | 0.193 | 0.1387 | No |
| 73 | PLCL1 | na |  |  | 9245 | 0.188 | 0.1374 | No |
| 74 | RPN1 | na |  |  | 9273 | 0.183 | 0.1376 | No |
| 75 | CNP | na |  |  | 10235 | 0.077 | 0.0879 | No |
| 76 | HSPA13 | na |  |  | 10533 | 0.050 | 0.0728 | No |
| 77 | E2F5 | na |  |  | 10816 | 0.012 | 0.0581 | No |
| 78 | ONECUT1 | na |  |  | 10898 | 0.006 | 0.0539 | No |
| 79 | CDC34 | na |  |  | 10929 | 0.005 | 0.0524 | No |
| 80 | ALAS1 | na |  |  | 11139 | -0.018 | 0.0416 | No |
| 81 | TCHH | na |  |  | 11472 | -0.059 | 0.0247 | No |
| 82 | KLHDC3 | na |  |  | 11619 | -0.073 | 0.0177 | No |
| 83 | IL6ST | na |  |  | 11649 | -0.077 | 0.0168 | No |
| 84 | BTG2 | na |  |  | 11938 | -0.118 | 0.0028 | No |
| 85 | SULT1A1 | na |  |  | 12401 | -0.170 | -0.0199 | No |
| 86 | ASNS | na |  |  | 12529 | -0.188 | -0.0249 | No |
| 87 | PRKCD | na |  |  | 12636 | -0.204 | -0.0287 | No |
| 88 | BAK1 | na |  |  | 12720 | -0.218 | -0.0311 | No |
| 89 | TST | na |  |  | 12730 | -0.219 | -0.0296 | No |
| 90 | STARD3 | na |  |  | 13040 | -0.250 | -0.0436 | No |
| 91 | RXRB | na |  |  | 13164 | -0.268 | -0.0477 | No |
| 92 | PDAP1 | na |  |  | 13491 | -0.315 | -0.0620 | No |
| 93 | WIZ | na |  |  | 13581 | -0.328 | -0.0637 | No |
| 94 | BID | na |  |  | 13709 | -0.347 | -0.0673 | No |
| 95 | APOM | na |  |  | 13804 | -0.350 | -0.0691 | No |
| 96 | PRKACA | na |  |  | 14275 | -0.418 | -0.0901 | No |
| 97 | RAB27A | na |  |  | 14331 | -0.422 | -0.0892 | No |
| 98 | SHOX2 | na |  |  | 14531 | -0.448 | -0.0957 | No |
| 99 | DGAT1 | na |  |  | 14826 | -0.502 | -0.1066 | No |
| 100 | NR4A1 | na |  |  | 15488 | -0.595 | -0.1360 | No |
| 101 | EPHX1 | na |  |  | 15777 | -0.644 | -0.1454 | No |
| 102 | STK25 | na |  |  | 15908 | -0.667 | -0.1462 | No |
| 103 | MRPL23 | na |  |  | 15933 | -0.673 | -0.1415 | No |
| 104 | GPX3 | na |  |  | 16209 | -0.732 | -0.1494 | No |
| 105 | CREG1 | na |  |  | 16229 | -0.739 | -0.1438 | No |
| 106 | DNAJB1 | na |  |  | 16236 | -0.741 | -0.1376 | No |
| 107 | GRINA | na |  |  | 16335 | -0.764 | -0.1359 | No |
| 108 | RHOB | na |  |  | 16399 | -0.781 | -0.1323 | No |
| 109 | ALDOA | na |  |  | 16455 | -0.799 | -0.1281 | No |
| 110 | BCL2L11 | na |  |  | 16498 | -0.810 | -0.1231 | No |
| 111 | BSG | na |  |  | 16641 | -0.844 | -0.1230 | No |
| 112 | SQSTM1 | na |  |  | 16663 | -0.848 | -0.1166 | No |
| 113 | HYAL2 | na |  |  | 16802 | -0.887 | -0.1159 | No |
| 114 | TAP1 | na |  |  | 17413 | -1.084 | -0.1383 | No |
| 115 | NFKBIA | na |  |  | 17437 | -1.096 | -0.1297 | No |
| 116 | JUNB | na |  |  | 17499 | -1.123 | -0.1229 | No |
| 117 | CXCL2 | na |  |  | 17501 | -1.124 | -0.1130 | No |
| 118 | CDKN1C | na |  |  | 17571 | -1.139 | -0.1065 | No |
| 119 | DLG4 | na |  |  | 17663 | -1.172 | -0.1008 | No |
| 120 | FOS | na |  |  | 17698 | -1.185 | -0.0921 | No |
| 121 | AQP3 | na |  |  | 17703 | -1.187 | -0.0817 | No |
| 122 | RRAD | na |  |  | 17756 | -1.204 | -0.0737 | No |
| 123 | SLC6A8 | na |  |  | 17923 | -1.288 | -0.0710 | No |
| 124 | HLA-F | na |  |  | 17948 | -1.299 | -0.0607 | No |
| 125 | ICAM1 | na |  |  | 17954 | -1.306 | -0.0494 | No |
| 126 | IRF1 | na |  |  | 18023 | -1.342 | -0.0410 | No |
| 127 | ENO2 | na |  |  | 18286 | -1.481 | -0.0416 | No |
| 128 | IL6 | na |  |  | 18776 | -2.020 | -0.0492 | No |
| 129 | BTG1 | na |  |  | 18836 | -2.149 | -0.0332 | No |
| 130 | SOD2 | na |  |  | 18970 | -2.521 | -0.0178 | No |
| 131 | BMP2 | na |  |  | 19119 | -3.339 | 0.0041 | No |
Table: GSEA details [plain text format]

  

Fig 2: HALLMARK\_UV\_RESPONSE\_UP      
 Blue-Pink O' Gram in the Space of the Analyzed GeneSet

  

Fig 3: HALLMARK\_UV\_RESPONSE\_UP: Random ES distribution      
 Gene set null distribution of ES for **HALLMARK\_UV\_RESPONSE\_UP**

  
