# Supplementary material for: Heparin-based hydrogel scaffolding alters the transcriptomic profile and increases the chemoresistance of MDA-MB-231 triple-negative breast cancer cells
Source: Biomater Sci. 2020 Feb 13;8(10):2786–96. doi: 10.1039/c9bm01481k (PMC7497406; doi:10.1039/c9bm01481k)
Supplement: Supplementary file 2 [file BM-008-C9BM01481K-s002.zip › Supplementary File 4/EGFvControl/Pathways/my_analysis.Gsea.1545200981068/HALLMARK_IL6_JAK_STAT3_SIGNALING.html]

Details for gene set HALLMARK\_IL6\_JAK\_STAT3\_SIGNALING[GSEA]

|  || Dataset | expr.class.cls#EGF\_versus\_CONTROL.class.cls#EGF\_versus\_CONTROL\_repos |
| Phenotype | class.cls#EGF\_versus\_CONTROL\_repos |
| Upregulated in class | CONTROL |
| GeneSet | HALLMARK\_IL6\_JAK\_STAT3\_SIGNALING |
| Enrichment Score (ES) | -0.5089243 |
| Normalized Enrichment Score (NES) | -2.0784495 |
| Nominal p-value | 0.0 |
| FDR q-value | 2.5E-4 |
| FWER p-Value | 0.001 |
Table: GSEA Results Summary

  

Fig 1: Enrichment plot: HALLMARK\_IL6\_JAK\_STAT3\_SIGNALING      
 Profile of the Running ES Score & Positions of GeneSet Members on the Rank Ordered List

  

| PROBE | DESCRIPTION (from dataset) | GENE SYMBOL | GENE\_TITLE | RANK IN GENE LIST | RANK METRIC SCORE | RUNNING ES | CORE ENRICHMENT || 1 | PTPN1 | na |  |  | 1865 | 1.253 | -0.0760 | No |
| 2 | CSF2RA | na |  |  | 1888 | 1.248 | -0.0557 | No |
| 3 | CBL | na |  |  | 2825 | 1.057 | -0.0865 | No |
| 4 | HMOX1 | na |  |  | 3011 | 1.026 | -0.0785 | No |
| 5 | TNFRSF12A | na |  |  | 3094 | 1.008 | -0.0655 | No |
| 6 | PDGFC | na |  |  | 3258 | 0.976 | -0.0573 | No |
| 7 | PTPN2 | na |  |  | 3585 | 0.923 | -0.0585 | No |
| 8 | CD44 | na |  |  | 3954 | 0.860 | -0.0630 | No |
| 9 | PTPN11 | na |  |  | 4074 | 0.840 | -0.0548 | No |
| 10 | HAX1 | na |  |  | 4113 | 0.835 | -0.0424 | No |
| 11 | GRB2 | na |  |  | 4705 | 0.748 | -0.0605 | No |
| 12 | STAT1 | na |  |  | 5448 | 0.645 | -0.0882 | No |
| 13 | STAM2 | na |  |  | 5658 | 0.616 | -0.0885 | No |
| 14 | FAS | na |  |  | 6266 | 0.532 | -0.1111 | No |
| 15 | ACVR1B | na |  |  | 10312 | 0.067 | -0.3214 | No |
| 16 | IL17RB | na |  |  | 10623 | 0.035 | -0.3369 | No |
| 17 | IFNAR1 | na |  |  | 11221 | -0.030 | -0.3676 | No |
| 18 | IL6ST | na |  |  | 11649 | -0.077 | -0.3886 | No |
| 19 | STAT3 | na |  |  | 12572 | -0.194 | -0.4335 | No |
| 20 | JUN | na |  |  | 12605 | -0.199 | -0.4317 | No |
| 21 | BAK1 | na |  |  | 12720 | -0.218 | -0.4340 | No |
| 22 | IL7 | na |  |  | 13349 | -0.295 | -0.4617 | No |
| 23 | IL13RA1 | na |  |  | 13405 | -0.303 | -0.4594 | No |
| 24 | MYD88 | na |  |  | 13595 | -0.329 | -0.4636 | No |
| 25 | TNFRSF1B | na |  |  | 13603 | -0.331 | -0.4583 | No |
| 26 | CXCL10 | na |  |  | 13845 | -0.359 | -0.4647 | No |
| 27 | IL15RA | na |  |  | 14050 | -0.385 | -0.4688 | No |
| 28 | IL17RA | na |  |  | 14201 | -0.405 | -0.4696 | No |
| 29 | TYK2 | na |  |  | 14465 | -0.439 | -0.4759 | No |
| 30 | LTBR | na |  |  | 14490 | -0.443 | -0.4695 | No |
| 31 | TLR2 | na |  |  | 15128 | -0.535 | -0.4936 | No |
| 32 | IL3RA | na |  |  | 15196 | -0.545 | -0.4878 | No |
| 33 | EBI3 | na |  |  | 15602 | -0.605 | -0.4985 | Yes |
| 34 | IL10RB | na |  |  | 15636 | -0.613 | -0.4897 | Yes |
| 35 | TNF | na |  |  | 15826 | -0.653 | -0.4884 | Yes |
| 36 | SOCS3 | na |  |  | 16002 | -0.686 | -0.4858 | Yes |
| 37 | IRF9 | na |  |  | 16304 | -0.759 | -0.4885 | Yes |
| 38 | SOCS1 | na |  |  | 16397 | -0.781 | -0.4799 | Yes |
| 39 | TNFRSF1A | na |  |  | 16404 | -0.783 | -0.4668 | Yes |
| 40 | CD14 | na |  |  | 16595 | -0.833 | -0.4624 | Yes |
| 41 | MAP3K8 | na |  |  | 16847 | -0.904 | -0.4600 | Yes |
| 42 | PIM1 | na |  |  | 17218 | -1.013 | -0.4620 | Yes |
| 43 | TNFRSF21 | na |  |  | 17238 | -1.017 | -0.4455 | Yes |
| 44 | IFNGR1 | na |  |  | 17299 | -1.042 | -0.4307 | Yes |
| 45 | CXCL1 | na |  |  | 17379 | -1.069 | -0.4165 | Yes |
| 46 | CXCL3 | na |  |  | 17464 | -1.110 | -0.4019 | Yes |
| 47 | CSF2 | na |  |  | 17557 | -1.135 | -0.3872 | Yes |
| 48 | TGFB1 | na |  |  | 17599 | -1.148 | -0.3696 | Yes |
| 49 | IRF1 | na |  |  | 18023 | -1.342 | -0.3687 | Yes |
| 50 | IL4R | na |  |  | 18107 | -1.380 | -0.3494 | Yes |
| 51 | STAT2 | na |  |  | 18119 | -1.384 | -0.3262 | Yes |
| 52 | CRLF2 | na |  |  | 18168 | -1.407 | -0.3046 | Yes |
| 53 | IL1B | na |  |  | 18217 | -1.433 | -0.2825 | Yes |
| 54 | IL1R1 | na |  |  | 18283 | -1.480 | -0.2605 | Yes |
| 55 | LEPR | na |  |  | 18407 | -1.575 | -0.2399 | Yes |
| 56 | IFNGR2 | na |  |  | 18421 | -1.594 | -0.2132 | Yes |
| 57 | CD9 | na |  |  | 18486 | -1.630 | -0.1886 | Yes |
| 58 | A2M | na |  |  | 18524 | -1.668 | -0.1619 | Yes |
| 59 | OSMR | na |  |  | 18646 | -1.810 | -0.1371 | Yes |
| 60 | LTB | na |  |  | 18739 | -1.948 | -0.1085 | Yes |
| 61 | IL6 | na |  |  | 18776 | -2.020 | -0.0757 | Yes |
| 62 | ITGB3 | na |  |  | 18931 | -2.380 | -0.0429 | Yes |
| 63 | CXCL11 | na |  |  | 19118 | -3.311 | 0.0042 | Yes |
Table: GSEA details [plain text format]

  

Fig 2: HALLMARK\_IL6\_JAK\_STAT3\_SIGNALING      
 Blue-Pink O' Gram in the Space of the Analyzed GeneSet

  

Fig 3: HALLMARK\_IL6\_JAK\_STAT3\_SIGNALING: Random ES distribution      
 Gene set null distribution of ES for **HALLMARK\_IL6\_JAK\_STAT3\_SIGNALING**

  
