# Supplementary material for: Heparin-based hydrogel scaffolding alters the transcriptomic profile and increases the chemoresistance of MDA-MB-231 triple-negative breast cancer cells
Source: Biomater Sci. 2020 Feb 13;8(10):2786–96. doi: 10.1039/c9bm01481k (PMC7497406; doi:10.1039/c9bm01481k)
Supplement: Supplementary file 2 [file BM-008-C9BM01481K-s002.zip › Supplementary File 4/EGFvControl/Pathways/my_analysis.Gsea.1545200981068/HALLMARK_XENOBIOTIC_METABOLISM.html]

Details for gene set HALLMARK\_XENOBIOTIC\_METABOLISM[GSEA]

|  || Dataset | expr.class.cls#EGF\_versus\_CONTROL.class.cls#EGF\_versus\_CONTROL\_repos |
| Phenotype | class.cls#EGF\_versus\_CONTROL\_repos |
| Upregulated in class | CONTROL |
| GeneSet | HALLMARK\_XENOBIOTIC\_METABOLISM |
| Enrichment Score (ES) | -0.2006357 |
| Normalized Enrichment Score (NES) | -0.94333106 |
| Nominal p-value | 0.58196723 |
| FDR q-value | 0.6239393 |
| FWER p-Value | 1.0 |
Table: GSEA Results Summary

  

Fig 1: Enrichment plot: HALLMARK\_XENOBIOTIC\_METABOLISM      
 Profile of the Running ES Score & Positions of GeneSet Members on the Rank Ordered List

  

| PROBE | DESCRIPTION (from dataset) | GENE SYMBOL | GENE\_TITLE | RANK IN GENE LIST | RANK METRIC SCORE | RUNNING ES | CORE ENRICHMENT || 1 | CYP1A1 | na |  |  | 96 | 2.351 | 0.0141 | Yes |
| 2 | FBP1 | na |  |  | 101 | 2.344 | 0.0329 | Yes |
| 3 | NQO1 | na |  |  | 141 | 2.223 | 0.0489 | Yes |
| 4 | SLC1A5 | na |  |  | 171 | 2.171 | 0.0650 | Yes |
| 5 | AOX1 | na |  |  | 211 | 2.076 | 0.0799 | Yes |
| 6 | ASL | na |  |  | 340 | 1.901 | 0.0886 | Yes |
| 7 | LCAT | na |  |  | 365 | 1.875 | 0.1026 | Yes |
| 8 | KARS | na |  |  | 427 | 1.823 | 0.1142 | Yes |
| 9 | GSS | na |  |  | 682 | 1.655 | 0.1143 | Yes |
| 10 | SSR3 | na |  |  | 684 | 1.654 | 0.1277 | Yes |
| 11 | TMEM97 | na |  |  | 776 | 1.599 | 0.1359 | Yes |
| 12 | AP4B1 | na |  |  | 819 | 1.581 | 0.1465 | Yes |
| 13 | PGRMC1 | na |  |  | 1119 | 1.473 | 0.1428 | Yes |
| 14 | CBR1 | na |  |  | 1267 | 1.424 | 0.1466 | Yes |
| 15 | MTHFD1 | na |  |  | 1295 | 1.413 | 0.1567 | Yes |
| 16 | PTS | na |  |  | 1498 | 1.346 | 0.1570 | Yes |
| 17 | SAR1B | na |  |  | 1670 | 1.303 | 0.1586 | Yes |
| 18 | PTGR1 | na |  |  | 1689 | 1.297 | 0.1682 | Yes |
| 19 | PAPSS2 | na |  |  | 1742 | 1.283 | 0.1759 | Yes |
| 20 | ETS2 | na |  |  | 1829 | 1.261 | 0.1817 | Yes |
| 21 | ELOVL5 | na |  |  | 1901 | 1.246 | 0.1881 | Yes |
| 22 | GART | na |  |  | 1995 | 1.220 | 0.1931 | Yes |
| 23 | UPP1 | na |  |  | 2597 | 1.095 | 0.1704 | Yes |
| 24 | PEMT | na |  |  | 2609 | 1.093 | 0.1787 | Yes |
| 25 | HPRT1 | na |  |  | 2703 | 1.076 | 0.1826 | Yes |
| 26 | PTGES3 | na |  |  | 2955 | 1.034 | 0.1778 | Yes |
| 27 | MPP2 | na |  |  | 2988 | 1.029 | 0.1845 | Yes |
| 28 | HMOX1 | na |  |  | 3011 | 1.026 | 0.1917 | Yes |
| 29 | SLC35D1 | na |  |  | 3115 | 1.004 | 0.1944 | Yes |
| 30 | GCNT2 | na |  |  | 3217 | 0.983 | 0.1971 | Yes |
| 31 | ADH5 | na |  |  | 3376 | 0.958 | 0.1966 | Yes |
| 32 | AHCY | na |  |  | 3594 | 0.921 | 0.1927 | Yes |
| 33 | ACP1 | na |  |  | 3596 | 0.921 | 0.2001 | Yes |
| 34 | HACL1 | na |  |  | 3804 | 0.883 | 0.1964 | No |
| 35 | SLC12A4 | na |  |  | 4131 | 0.832 | 0.1861 | No |
| 36 | ATP2A2 | na |  |  | 4160 | 0.828 | 0.1913 | No |
| 37 | TTPA | na |  |  | 4555 | 0.770 | 0.1769 | No |
| 38 | MCCC2 | na |  |  | 4886 | 0.719 | 0.1654 | No |
| 39 | ARPP19 | na |  |  | 5182 | 0.681 | 0.1555 | No |
| 40 | BCAT1 | na |  |  | 5614 | 0.624 | 0.1379 | No |
| 41 | SLC35B1 | na |  |  | 5725 | 0.606 | 0.1371 | No |
| 42 | ALDH9A1 | na |  |  | 5781 | 0.599 | 0.1390 | No |
| 43 | FBLN1 | na |  |  | 6025 | 0.567 | 0.1309 | No |
| 44 | FAS | na |  |  | 6266 | 0.532 | 0.1226 | No |
| 45 | GSTT2 | na |  |  | 6301 | 0.527 | 0.1251 | No |
| 46 | SERTAD1 | na |  |  | 6364 | 0.518 | 0.1261 | No |
| 47 | SHMT2 | na |  |  | 6749 | 0.474 | 0.1098 | No |
| 48 | GCLC | na |  |  | 6772 | 0.472 | 0.1124 | No |
| 49 | GSTM4 | na |  |  | 6850 | 0.462 | 0.1121 | No |
| 50 | GCH1 | na |  |  | 6881 | 0.459 | 0.1143 | No |
| 51 | CNDP2 | na |  |  | 7074 | 0.437 | 0.1078 | No |
| 52 | FMO3 | na |  |  | 7222 | 0.419 | 0.1035 | No |
| 53 | ATOH8 | na |  |  | 7419 | 0.394 | 0.0964 | No |
| 54 | PDLIM5 | na |  |  | 7457 | 0.391 | 0.0976 | No |
| 55 | EPHA2 | na |  |  | 7485 | 0.387 | 0.0993 | No |
| 56 | CROT | na |  |  | 7490 | 0.386 | 0.1023 | No |
| 57 | TMBIM6 | na |  |  | 7503 | 0.385 | 0.1048 | No |
| 58 | CYP2J2 | na |  |  | 7536 | 0.380 | 0.1062 | No |
| 59 | CDA | na |  |  | 7578 | 0.376 | 0.1071 | No |
| 60 | ALDH2 | na |  |  | 7882 | 0.339 | 0.0939 | No |
| 61 | NMT1 | na |  |  | 7979 | 0.328 | 0.0915 | No |
| 62 | GAD1 | na |  |  | 7981 | 0.328 | 0.0942 | No |
| 63 | TGFB2 | na |  |  | 8117 | 0.315 | 0.0896 | No |
| 64 | TPST1 | na |  |  | 8204 | 0.304 | 0.0876 | No |
| 65 | GCKR | na |  |  | 8298 | 0.293 | 0.0851 | No |
| 66 | MAOA | na |  |  | 8409 | 0.283 | 0.0816 | No |
| 67 | ENTPD5 | na |  |  | 8443 | 0.280 | 0.0821 | No |
| 68 | CYP2E1 | na |  |  | 8482 | 0.274 | 0.0824 | No |
| 69 | IDH1 | na |  |  | 8504 | 0.271 | 0.0835 | No |
| 70 | HES6 | na |  |  | 8713 | 0.244 | 0.0745 | No |
| 71 | DHRS7 | na |  |  | 8780 | 0.239 | 0.0730 | No |
| 72 | AKR1C3 | na |  |  | 9385 | 0.174 | 0.0427 | No |
| 73 | ACOX1 | na |  |  | 9429 | 0.166 | 0.0418 | No |
| 74 | GSTO1 | na |  |  | 9438 | 0.165 | 0.0427 | No |
| 75 | LONP1 | na |  |  | 9444 | 0.164 | 0.0438 | No |
| 76 | RAP1GAP | na |  |  | 9901 | 0.116 | 0.0208 | No |
| 77 | PGD | na |  |  | 9934 | 0.111 | 0.0200 | No |
| 78 | ARG2 | na |  |  | 10070 | 0.097 | 0.0137 | No |
| 79 | DCXR | na |  |  | 10151 | 0.088 | 0.0102 | No |
| 80 | PINK1 | na |  |  | 10456 | 0.058 | -0.0052 | No |
| 81 | ACOX2 | na |  |  | 10488 | 0.054 | -0.0064 | No |
| 82 | MARCH6 | na |  |  | 11022 | -0.006 | -0.0344 | No |
| 83 | ALAS1 | na |  |  | 11139 | -0.018 | -0.0403 | No |
| 84 | HGFAC | na |  |  | 11660 | -0.080 | -0.0670 | No |
| 85 | POR | na |  |  | 11704 | -0.087 | -0.0685 | No |
| 86 | ID2 | na |  |  | 11940 | -0.118 | -0.0799 | No |
| 87 | SLC46A3 | na |  |  | 11962 | -0.121 | -0.0800 | No |
| 88 | PYCR1 | na |  |  | 12337 | -0.160 | -0.0983 | No |
| 89 | RETSAT | na |  |  | 12351 | -0.162 | -0.0977 | No |
| 90 | DHPS | na |  |  | 12406 | -0.171 | -0.0992 | No |
| 91 | BCAR1 | na |  |  | 12438 | -0.176 | -0.0993 | No |
| 92 | SLC6A6 | na |  |  | 12600 | -0.197 | -0.1062 | No |
| 93 | PPARD | na |  |  | 12731 | -0.219 | -0.1112 | No |
| 94 | CYB5A | na |  |  | 12910 | -0.233 | -0.1187 | No |
| 95 | PMM1 | na |  |  | 12923 | -0.235 | -0.1174 | No |
| 96 | UGDH | na |  |  | 13035 | -0.249 | -0.1212 | No |
| 97 | NFS1 | na |  |  | 13041 | -0.250 | -0.1195 | No |
| 98 | AKR1C2 | na |  |  | 13166 | -0.268 | -0.1238 | No |
| 99 | ABHD6 | na |  |  | 13203 | -0.274 | -0.1235 | No |
| 100 | DDT | na |  |  | 13546 | -0.322 | -0.1388 | No |
| 101 | PLG | na |  |  | 13548 | -0.322 | -0.1362 | No |
| 102 | FAH | na |  |  | 13686 | -0.343 | -0.1406 | No |
| 103 | ACO2 | na |  |  | 13859 | -0.360 | -0.1467 | No |
| 104 | GNMT | na |  |  | 13953 | -0.370 | -0.1486 | No |
| 105 | CASP6 | na |  |  | 13966 | -0.372 | -0.1462 | No |
| 106 | ETFDH | na |  |  | 14057 | -0.387 | -0.1478 | No |
| 107 | CYP2S1 | na |  |  | 14138 | -0.397 | -0.1488 | No |
| 108 | ACP2 | na |  |  | 14143 | -0.398 | -0.1457 | No |
| 109 | SMOX | na |  |  | 14262 | -0.416 | -0.1486 | No |
| 110 | BLVRB | na |  |  | 14274 | -0.418 | -0.1457 | No |
| 111 | GSR | na |  |  | 14564 | -0.456 | -0.1572 | No |
| 112 | ACOX3 | na |  |  | 14801 | -0.497 | -0.1656 | No |
| 113 | MT2A | na |  |  | 14804 | -0.498 | -0.1616 | No |
| 114 | LEAP2 | na |  |  | 14937 | -0.505 | -0.1644 | No |
| 115 | ALDH3A1 | na |  |  | 14969 | -0.509 | -0.1619 | No |
| 116 | VNN1 | na |  |  | 15302 | -0.564 | -0.1748 | No |
| 117 | ABCC2 | na |  |  | 15386 | -0.580 | -0.1744 | No |
| 118 | CAT | na |  |  | 15662 | -0.617 | -0.1838 | No |
| 119 | CYFIP2 | na |  |  | 15765 | -0.639 | -0.1840 | No |
| 120 | EPHX1 | na |  |  | 15777 | -0.644 | -0.1794 | No |
| 121 | MAN1A1 | na |  |  | 15990 | -0.684 | -0.1849 | No |
| 122 | COMT | na |  |  | 16042 | -0.693 | -0.1820 | No |
| 123 | ECH1 | na |  |  | 16356 | -0.770 | -0.1921 | No |
| 124 | TNFRSF1A | na |  |  | 16404 | -0.783 | -0.1883 | No |
| 125 | NPC1 | na |  |  | 16451 | -0.798 | -0.1842 | No |
| 126 | NINJ1 | na |  |  | 16489 | -0.808 | -0.1796 | No |
| 127 | IGFBP1 | na |  |  | 16692 | -0.855 | -0.1832 | No |
| 128 | BPHL | na |  |  | 16938 | -0.932 | -0.1885 | No |
| 129 | DHRS1 | na |  |  | 17170 | -1.001 | -0.1925 | No |
| 130 | APOE | na |  |  | 17293 | -1.040 | -0.1905 | No |
| 131 | PC | na |  |  | 17387 | -1.072 | -0.1866 | No |
| 132 | DDAH2 | na |  |  | 17483 | -1.116 | -0.1826 | No |
| 133 | UPB1 | na |  |  | 17533 | -1.133 | -0.1759 | No |
| 134 | VTN | na |  |  | 17582 | -1.142 | -0.1692 | No |
| 135 | PSMB10 | na |  |  | 17606 | -1.151 | -0.1610 | No |
| 136 | CFB | na |  |  | 17623 | -1.158 | -0.1524 | No |
| 137 | SPINT2 | na |  |  | 17657 | -1.169 | -0.1447 | No |
| 138 | CSAD | na |  |  | 17881 | -1.265 | -0.1461 | No |
| 139 | PDK4 | na |  |  | 17892 | -1.269 | -0.1363 | No |
| 140 | JUP | na |  |  | 17913 | -1.281 | -0.1270 | No |
| 141 | PTGES | na |  |  | 17960 | -1.311 | -0.1187 | No |
| 142 | ABCC3 | na |  |  | 18067 | -1.363 | -0.1132 | No |
| 143 | HSD11B1 | na |  |  | 18258 | -1.461 | -0.1113 | No |
| 144 | IL1R1 | na |  |  | 18283 | -1.480 | -0.1005 | No |
| 145 | LPIN2 | na |  |  | 18431 | -1.604 | -0.0952 | No |
| 146 | XDH | na |  |  | 18541 | -1.683 | -0.0873 | No |
| 147 | CYP27A1 | na |  |  | 18645 | -1.808 | -0.0780 | No |
| 148 | GABARAPL1 | na |  |  | 19059 | -2.914 | -0.0760 | No |
| 149 | ITIH4 | na |  |  | 19125 | -3.366 | -0.0521 | No |
| 150 | KYNU | na |  |  | 19126 | -3.367 | -0.0247 | No |
| 151 | PROS1 | na |  |  | 19137 | -3.498 | 0.0032 | No |
Table: GSEA details [plain text format]

  

Fig 2: HALLMARK\_XENOBIOTIC\_METABOLISM      
 Blue-Pink O' Gram in the Space of the Analyzed GeneSet

  

Fig 3: HALLMARK\_XENOBIOTIC\_METABOLISM: Random ES distribution      
 Gene set null distribution of ES for **HALLMARK\_XENOBIOTIC\_METABOLISM**

  
