# Supplementary material for: Heparin-based hydrogel scaffolding alters the transcriptomic profile and increases the chemoresistance of MDA-MB-231 triple-negative breast cancer cells
Source: Biomater Sci. 2020 Feb 13;8(10):2786–96. doi: 10.1039/c9bm01481k (PMC7497406; doi:10.1039/c9bm01481k)
Supplement: Supplementary file 2 [file BM-008-C9BM01481K-s002.zip › Supplementary File 4/EGFvControl/Pathways/my_analysis.Gsea.1545200981068/HALLMARK_HYPOXIA.html]

Details for gene set HALLMARK\_HYPOXIA[GSEA]

|  || Dataset | expr.class.cls#EGF\_versus\_CONTROL.class.cls#EGF\_versus\_CONTROL\_repos |
| Phenotype | class.cls#EGF\_versus\_CONTROL\_repos |
| Upregulated in class | CONTROL |
| GeneSet | HALLMARK\_HYPOXIA |
| Enrichment Score (ES) | -0.5433389 |
| Normalized Enrichment Score (NES) | -2.5954623 |
| Nominal p-value | 0.0 |
| FDR q-value | 0.0 |
| FWER p-Value | 0.0 |
Table: GSEA Results Summary

  

Fig 1: Enrichment plot: HALLMARK\_HYPOXIA      
 Profile of the Running ES Score & Positions of GeneSet Members on the Rank Ordered List

  

| PROBE | DESCRIPTION (from dataset) | GENE SYMBOL | GENE\_TITLE | RANK IN GENE LIST | RANK METRIC SCORE | RUNNING ES | CORE ENRICHMENT || 1 | FBP1 | na |  |  | 101 | 2.344 | 0.0075 | No |
| 2 | PGM2 | na |  |  | 395 | 1.849 | 0.0021 | No |
| 3 | NEDD4L | na |  |  | 473 | 1.786 | 0.0078 | No |
| 4 | ADORA2B | na |  |  | 492 | 1.775 | 0.0165 | No |
| 5 | PLAC8 | na |  |  | 500 | 1.772 | 0.0258 | No |
| 6 | F3 | na |  |  | 545 | 1.742 | 0.0330 | No |
| 7 | MT1E | na |  |  | 578 | 1.719 | 0.0407 | No |
| 8 | HOXB9 | na |  |  | 655 | 1.667 | 0.0457 | No |
| 9 | SCARB1 | na |  |  | 794 | 1.593 | 0.0472 | No |
| 10 | CTGF | na |  |  | 1080 | 1.486 | 0.0403 | No |
| 11 | BCL2 | na |  |  | 1163 | 1.454 | 0.0439 | No |
| 12 | EGFR | na |  |  | 1241 | 1.429 | 0.0476 | No |
| 13 | DTNA | na |  |  | 1386 | 1.383 | 0.0476 | No |
| 14 | ETS1 | na |  |  | 1416 | 1.372 | 0.0535 | No |
| 15 | CAV1 | na |  |  | 1542 | 1.335 | 0.0542 | No |
| 16 | JMJD6 | na |  |  | 1940 | 1.237 | 0.0401 | No |
| 17 | SIAH2 | na |  |  | 2015 | 1.216 | 0.0428 | No |
| 18 | PPP1R3C | na |  |  | 2401 | 1.133 | 0.0288 | No |
| 19 | CHST3 | na |  |  | 2403 | 1.132 | 0.0349 | No |
| 20 | HMOX1 | na |  |  | 3011 | 1.026 | 0.0086 | No |
| 21 | GCNT2 | na |  |  | 3217 | 0.983 | 0.0032 | No |
| 22 | PRKCA | na |  |  | 4313 | 0.806 | -0.0500 | No |
| 23 | CXCR4 | na |  |  | 4387 | 0.794 | -0.0495 | No |
| 24 | EXT1 | na |  |  | 5006 | 0.707 | -0.0781 | No |
| 25 | NR3C1 | na |  |  | 5093 | 0.695 | -0.0788 | No |
| 26 | STC2 | na |  |  | 5348 | 0.658 | -0.0886 | No |
| 27 | TPST2 | na |  |  | 5659 | 0.616 | -0.1015 | No |
| 28 | CSRP2 | na |  |  | 5704 | 0.608 | -0.1005 | No |
| 29 | TGM2 | na |  |  | 6481 | 0.505 | -0.1385 | No |
| 30 | NDST1 | na |  |  | 6690 | 0.481 | -0.1468 | No |
| 31 | DDIT3 | na |  |  | 6794 | 0.469 | -0.1497 | No |
| 32 | ANGPTL4 | na |  |  | 6796 | 0.468 | -0.1472 | No |
| 33 | TES | na |  |  | 6856 | 0.461 | -0.1478 | No |
| 34 | SLC37A4 | na |  |  | 6924 | 0.453 | -0.1489 | No |
| 35 | ATF3 | na |  |  | 7410 | 0.395 | -0.1722 | No |
| 36 | AKAP12 | na |  |  | 7591 | 0.374 | -0.1796 | No |
| 37 | TPD52 | na |  |  | 7821 | 0.347 | -0.1897 | No |
| 38 | RBPJ | na |  |  | 8652 | 0.254 | -0.2320 | No |
| 39 | TIPARP | na |  |  | 8676 | 0.250 | -0.2318 | No |
| 40 | PPP1R15A | na |  |  | 8701 | 0.246 | -0.2317 | No |
| 41 | SDC2 | na |  |  | 8869 | 0.228 | -0.2393 | No |
| 42 | IRS2 | na |  |  | 9101 | 0.201 | -0.2503 | No |
| 43 | FOXO3 | na |  |  | 9106 | 0.201 | -0.2494 | No |
| 44 | HDLBP | na |  |  | 9190 | 0.193 | -0.2527 | No |
| 45 | PYGM | na |  |  | 9209 | 0.191 | -0.2526 | No |
| 46 | HK1 | na |  |  | 9367 | 0.177 | -0.2599 | No |
| 47 | CHST2 | na |  |  | 9739 | 0.132 | -0.2787 | No |
| 48 | CITED2 | na |  |  | 9844 | 0.121 | -0.2835 | No |
| 49 | UGP2 | na |  |  | 9929 | 0.111 | -0.2873 | No |
| 50 | ILVBL | na |  |  | 10005 | 0.106 | -0.2907 | No |
| 51 | GPC1 | na |  |  | 10254 | 0.075 | -0.3033 | No |
| 52 | ENO3 | na |  |  | 10445 | 0.059 | -0.3129 | No |
| 53 | MAP3K1 | na |  |  | 10453 | 0.059 | -0.3130 | No |
| 54 | GALK1 | na |  |  | 11038 | -0.007 | -0.3436 | No |
| 55 | XPNPEP1 | na |  |  | 11807 | -0.100 | -0.3834 | No |
| 56 | B3GALT6 | na |  |  | 12175 | -0.140 | -0.4019 | No |
| 57 | NDST2 | na |  |  | 12298 | -0.154 | -0.4075 | No |
| 58 | SLC6A6 | na |  |  | 12600 | -0.197 | -0.4222 | No |
| 59 | JUN | na |  |  | 12605 | -0.199 | -0.4214 | No |
| 60 | SLC2A1 | na |  |  | 12606 | -0.200 | -0.4203 | No |
| 61 | SAP30 | na |  |  | 12691 | -0.212 | -0.4235 | No |
| 62 | SLC25A1 | na |  |  | 13138 | -0.264 | -0.4455 | No |
| 63 | RRAGD | na |  |  | 13146 | -0.265 | -0.4445 | No |
| 64 | SRPX | na |  |  | 13308 | -0.288 | -0.4514 | No |
| 65 | VHL | na |  |  | 13524 | -0.320 | -0.4609 | No |
| 66 | PGM1 | na |  |  | 13543 | -0.322 | -0.4601 | No |
| 67 | CDKN1B | na |  |  | 13864 | -0.360 | -0.4749 | No |
| 68 | CASP6 | na |  |  | 13966 | -0.372 | -0.4782 | No |
| 69 | KLF6 | na |  |  | 14086 | -0.390 | -0.4824 | No |
| 70 | PDGFB | na |  |  | 14224 | -0.409 | -0.4873 | No |
| 71 | HK2 | na |  |  | 14409 | -0.434 | -0.4946 | No |
| 72 | DUSP1 | na |  |  | 14724 | -0.484 | -0.5085 | No |
| 73 | MT2A | na |  |  | 14804 | -0.498 | -0.5099 | No |
| 74 | BCAN | na |  |  | 14893 | -0.502 | -0.5118 | No |
| 75 | CP | na |  |  | 15210 | -0.548 | -0.5254 | No |
| 76 | STBD1 | na |  |  | 15538 | -0.600 | -0.5393 | No |
| 77 | PFKP | na |  |  | 15615 | -0.609 | -0.5400 | Yes |
| 78 | PLAUR | na |  |  | 15641 | -0.614 | -0.5380 | Yes |
| 79 | WSB1 | na |  |  | 15673 | -0.620 | -0.5362 | Yes |
| 80 | GPI | na |  |  | 15768 | -0.641 | -0.5377 | Yes |
| 81 | NAGK | na |  |  | 15821 | -0.653 | -0.5369 | Yes |
| 82 | HEXA | na |  |  | 15861 | -0.660 | -0.5353 | Yes |
| 83 | CDKN1A | na |  |  | 15950 | -0.678 | -0.5363 | Yes |
| 84 | PDK1 | na |  |  | 15998 | -0.686 | -0.5350 | Yes |
| 85 | EDN2 | na |  |  | 16086 | -0.701 | -0.5358 | Yes |
| 86 | FOSL2 | na |  |  | 16125 | -0.710 | -0.5339 | Yes |
| 87 | EFNA3 | na |  |  | 16136 | -0.713 | -0.5305 | Yes |
| 88 | HSPA5 | na |  |  | 16140 | -0.715 | -0.5268 | Yes |
| 89 | GRHPR | na |  |  | 16141 | -0.715 | -0.5229 | Yes |
| 90 | ZNF292 | na |  |  | 16428 | -0.791 | -0.5336 | Yes |
| 91 | ALDOA | na |  |  | 16455 | -0.799 | -0.5306 | Yes |
| 92 | TPI1 | na |  |  | 16603 | -0.835 | -0.5338 | Yes |
| 93 | IGFBP1 | na |  |  | 16692 | -0.855 | -0.5338 | Yes |
| 94 | MAFF | na |  |  | 16697 | -0.856 | -0.5293 | Yes |
| 95 | PFKL | na |  |  | 16708 | -0.859 | -0.5252 | Yes |
| 96 | COL5A1 | na |  |  | 16811 | -0.890 | -0.5257 | Yes |
| 97 | TGFB3 | na |  |  | 16834 | -0.900 | -0.5220 | Yes |
| 98 | ADM | na |  |  | 16860 | -0.908 | -0.5183 | Yes |
| 99 | INHA | na |  |  | 16874 | -0.912 | -0.5140 | Yes |
| 100 | PLIN2 | na |  |  | 16877 | -0.913 | -0.5092 | Yes |
| 101 | GYS1 | na |  |  | 16931 | -0.931 | -0.5069 | Yes |
| 102 | PHKG1 | na |  |  | 16951 | -0.936 | -0.5028 | Yes |
| 103 | GBE1 | na |  |  | 16983 | -0.947 | -0.4993 | Yes |
| 104 | KDELR3 | na |  |  | 17046 | -0.961 | -0.4973 | Yes |
| 105 | FAM162A | na |  |  | 17146 | -0.993 | -0.4971 | Yes |
| 106 | PIM1 | na |  |  | 17218 | -1.013 | -0.4953 | Yes |
| 107 | SLC2A5 | na |  |  | 17292 | -1.039 | -0.4935 | Yes |
| 108 | HS3ST1 | na |  |  | 17312 | -1.048 | -0.4888 | Yes |
| 109 | TNFAIP3 | na |  |  | 17494 | -1.121 | -0.4922 | Yes |
| 110 | CDKN1C | na |  |  | 17571 | -1.139 | -0.4900 | Yes |
| 111 | BNIP3L | na |  |  | 17581 | -1.142 | -0.4842 | Yes |
| 112 | IDS | na |  |  | 17625 | -1.160 | -0.4802 | Yes |
| 113 | FOS | na |  |  | 17698 | -1.185 | -0.4775 | Yes |
| 114 | SDC4 | na |  |  | 17717 | -1.190 | -0.4720 | Yes |
| 115 | EFNA1 | na |  |  | 17738 | -1.198 | -0.4665 | Yes |
| 116 | DDIT4 | na |  |  | 17752 | -1.202 | -0.4606 | Yes |
| 117 | SDC3 | na |  |  | 17812 | -1.229 | -0.4570 | Yes |
| 118 | TGFBI | na |  |  | 17865 | -1.259 | -0.4529 | Yes |
| 119 | ANKZF1 | na |  |  | 17879 | -1.263 | -0.4467 | Yes |
| 120 | KDM3A | na |  |  | 17911 | -1.281 | -0.4414 | Yes |
| 121 | P4HA2 | na |  |  | 17972 | -1.316 | -0.4374 | Yes |
| 122 | P4HA1 | na |  |  | 18001 | -1.330 | -0.4316 | Yes |
| 123 | PFKFB3 | na |  |  | 18027 | -1.345 | -0.4256 | Yes |
| 124 | AK4 | na |  |  | 18050 | -1.351 | -0.4194 | Yes |
| 125 | GAA | na |  |  | 18088 | -1.375 | -0.4139 | Yes |
| 126 | ZFP36 | na |  |  | 18138 | -1.393 | -0.4089 | Yes |
| 127 | LOX | na |  |  | 18158 | -1.403 | -0.4022 | Yes |
| 128 | GLRX | na |  |  | 18169 | -1.407 | -0.3951 | Yes |
| 129 | NFIL3 | na |  |  | 18225 | -1.442 | -0.3901 | Yes |
| 130 | PGF | na |  |  | 18251 | -1.458 | -0.3835 | Yes |
| 131 | KLF7 | na |  |  | 18264 | -1.466 | -0.3762 | Yes |
| 132 | ENO2 | na |  |  | 18286 | -1.481 | -0.3692 | Yes |
| 133 | PDK3 | na |  |  | 18298 | -1.493 | -0.3616 | Yes |
| 134 | BHLHE40 | na |  |  | 18387 | -1.556 | -0.3578 | Yes |
| 135 | IGFBP3 | na |  |  | 18475 | -1.624 | -0.3535 | Yes |
| 136 | ISG20 | na |  |  | 18505 | -1.647 | -0.3461 | Yes |
| 137 | PNRC1 | na |  |  | 18528 | -1.672 | -0.3381 | Yes |
| 138 | ATP7A | na |  |  | 18638 | -1.802 | -0.3340 | Yes |
| 139 | PAM | na |  |  | 18737 | -1.945 | -0.3286 | Yes |
| 140 | TMEM45A | na |  |  | 18749 | -1.966 | -0.3185 | Yes |
| 141 | PPARGC1A | na |  |  | 18762 | -1.989 | -0.3083 | Yes |
| 142 | IL6 | na |  |  | 18776 | -2.020 | -0.2980 | Yes |
| 143 | PGK1 | na |  |  | 18818 | -2.107 | -0.2887 | Yes |
| 144 | BTG1 | na |  |  | 18836 | -2.149 | -0.2779 | Yes |
| 145 | TPBG | na |  |  | 18842 | -2.163 | -0.2663 | Yes |
| 146 | LXN | na |  |  | 18850 | -2.183 | -0.2548 | Yes |
| 147 | ERRFI1 | na |  |  | 18935 | -2.395 | -0.2462 | Yes |
| 148 | VLDLR | na |  |  | 18950 | -2.433 | -0.2337 | Yes |
| 149 | PRDX5 | na |  |  | 18957 | -2.467 | -0.2206 | Yes |
| 150 | SELENBP1 | na |  |  | 18959 | -2.473 | -0.2072 | Yes |
| 151 | AMPD3 | na |  |  | 18995 | -2.631 | -0.1947 | Yes |
| 152 | STC1 | na |  |  | 18997 | -2.647 | -0.1803 | Yes |
| 153 | MXI1 | na |  |  | 19008 | -2.689 | -0.1662 | Yes |
| 154 | SLC2A3 | na |  |  | 19034 | -2.803 | -0.1523 | Yes |
| 155 | WISP2 | na |  |  | 19038 | -2.829 | -0.1370 | Yes |
| 156 | S100A4 | na |  |  | 19044 | -2.881 | -0.1216 | Yes |
| 157 | CCNG2 | na |  |  | 19058 | -2.911 | -0.1064 | Yes |
| 158 | CA12 | na |  |  | 19077 | -2.973 | -0.0912 | Yes |
| 159 | NDRG1 | na |  |  | 19083 | -3.028 | -0.0750 | Yes |
| 160 | RORA | na |  |  | 19112 | -3.279 | -0.0586 | Yes |
| 161 | KLHL24 | na |  |  | 19115 | -3.283 | -0.0408 | Yes |
| 162 | PPFIA4 | na |  |  | 19170 | -4.119 | -0.0212 | Yes |
| 163 | ALDOC | na |  |  | 19173 | -4.164 | 0.0013 | Yes |
Table: GSEA details [plain text format]

  

Fig 2: HALLMARK\_HYPOXIA      
 Blue-Pink O' Gram in the Space of the Analyzed GeneSet

  

Fig 3: HALLMARK\_HYPOXIA: Random ES distribution      
 Gene set null distribution of ES for **HALLMARK\_HYPOXIA**

  
