# Supplementary material for: Heparin-based hydrogel scaffolding alters the transcriptomic profile and increases the chemoresistance of MDA-MB-231 triple-negative breast cancer cells
Source: Biomater Sci. 2020 Feb 13;8(10):2786–96. doi: 10.1039/c9bm01481k (PMC7497406; doi:10.1039/c9bm01481k)
Supplement: Supplementary file 2 [file BM-008-C9BM01481K-s002.zip › Supplementary File 4/EGFvControl/Pathways/my_analysis.Gsea.1545200981068/HALLMARK_ALLOGRAFT_REJECTION.html]

Details for gene set HALLMARK\_ALLOGRAFT\_REJECTION[GSEA]

|  || Dataset | expr.class.cls#EGF\_versus\_CONTROL.class.cls#EGF\_versus\_CONTROL\_repos |
| Phenotype | class.cls#EGF\_versus\_CONTROL\_repos |
| Upregulated in class | CONTROL |
| GeneSet | HALLMARK\_ALLOGRAFT\_REJECTION |
| Enrichment Score (ES) | -0.3473968 |
| Normalized Enrichment Score (NES) | -1.6035224 |
| Nominal p-value | 0.0 |
| FDR q-value | 0.009755166 |
| FWER p-Value | 0.103 |
Table: GSEA Results Summary

  

Fig 1: Enrichment plot: HALLMARK\_ALLOGRAFT\_REJECTION      
 Profile of the Running ES Score & Positions of GeneSet Members on the Rank Ordered List

  

| PROBE | DESCRIPTION (from dataset) | GENE SYMBOL | GENE\_TITLE | RANK IN GENE LIST | RANK METRIC SCORE | RUNNING ES | CORE ENRICHMENT || 1 | IL11 | na |  |  | 57 | 2.570 | 0.0205 | No |
| 2 | IL18 | na |  |  | 152 | 2.202 | 0.0357 | No |
| 3 | IL12A | na |  |  | 170 | 2.171 | 0.0547 | No |
| 4 | NME1 | na |  |  | 764 | 1.603 | 0.0382 | No |
| 5 | ABCE1 | na |  |  | 1023 | 1.506 | 0.0385 | No |
| 6 | MRPL3 | na |  |  | 1117 | 1.473 | 0.0471 | No |
| 7 | EIF3D | na |  |  | 1221 | 1.435 | 0.0548 | No |
| 8 | EGFR | na |  |  | 1241 | 1.429 | 0.0669 | No |
| 9 | RARS | na |  |  | 1261 | 1.424 | 0.0789 | No |
| 10 | NLRP3 | na |  |  | 1376 | 1.386 | 0.0856 | No |
| 11 | ETS1 | na |  |  | 1416 | 1.372 | 0.0961 | No |
| 12 | EIF3J | na |  |  | 1474 | 1.355 | 0.1055 | No |
| 13 | CD8A | na |  |  | 1548 | 1.334 | 0.1138 | No |
| 14 | BRCA1 | na |  |  | 1620 | 1.312 | 0.1221 | No |
| 15 | IGSF6 | na |  |  | 1867 | 1.253 | 0.1207 | No |
| 16 | UBE2N | na |  |  | 1878 | 1.251 | 0.1316 | No |
| 17 | CCND3 | na |  |  | 2241 | 1.169 | 0.1233 | No |
| 18 | SRGN | na |  |  | 2364 | 1.142 | 0.1274 | No |
| 19 | MTIF2 | na |  |  | 2454 | 1.122 | 0.1330 | No |
| 20 | TAP2 | na |  |  | 2498 | 1.112 | 0.1409 | No |
| 21 | RPS19 | na |  |  | 2521 | 1.107 | 0.1498 | No |
| 22 | CSK | na |  |  | 2951 | 1.034 | 0.1368 | No |
| 23 | GCNT1 | na |  |  | 3320 | 0.967 | 0.1264 | No |
| 24 | EIF5A | na |  |  | 3538 | 0.930 | 0.1235 | No |
| 25 | HLA-DOB | na |  |  | 3796 | 0.884 | 0.1181 | No |
| 26 | GALNT1 | na |  |  | 4239 | 0.817 | 0.1024 | No |
| 27 | RPL9 | na |  |  | 4359 | 0.799 | 0.1035 | No |
| 28 | DEGS1 | na |  |  | 4669 | 0.753 | 0.0942 | No |
| 29 | RPS3A | na |  |  | 5141 | 0.686 | 0.0758 | No |
| 30 | STAT1 | na |  |  | 5448 | 0.645 | 0.0656 | No |
| 31 | ELF4 | na |  |  | 5569 | 0.630 | 0.0651 | No |
| 32 | BCAT1 | na |  |  | 5614 | 0.624 | 0.0685 | No |
| 33 | HDAC9 | na |  |  | 6002 | 0.570 | 0.0534 | No |
| 34 | RPS9 | na |  |  | 6233 | 0.536 | 0.0463 | No |
| 35 | FAS | na |  |  | 6266 | 0.532 | 0.0495 | No |
| 36 | JAK2 | na |  |  | 6438 | 0.510 | 0.0452 | No |
| 37 | DARS | na |  |  | 6451 | 0.508 | 0.0492 | No |
| 38 | IL27RA | na |  |  | 7090 | 0.435 | 0.0197 | No |
| 39 | TRAF2 | na |  |  | 7139 | 0.428 | 0.0211 | No |
| 40 | F2R | na |  |  | 7430 | 0.393 | 0.0095 | No |
| 41 | DYRK3 | na |  |  | 7484 | 0.387 | 0.0103 | No |
| 42 | AARS | na |  |  | 7584 | 0.375 | 0.0085 | No |
| 43 | LYN | na |  |  | 7669 | 0.366 | 0.0075 | No |
| 44 | MAP3K7 | na |  |  | 7759 | 0.355 | 0.0060 | No |
| 45 | TPD52 | na |  |  | 7821 | 0.347 | 0.0060 | No |
| 46 | GLMN | na |  |  | 7921 | 0.335 | 0.0039 | No |
| 47 | TGFB2 | na |  |  | 8117 | 0.315 | -0.0034 | No |
| 48 | EIF3A | na |  |  | 8140 | 0.312 | -0.0017 | No |
| 49 | ABI1 | na |  |  | 8474 | 0.275 | -0.0167 | No |
| 50 | AKT1 | na |  |  | 8668 | 0.251 | -0.0245 | No |
| 51 | TLR1 | na |  |  | 8781 | 0.239 | -0.0282 | No |
| 52 | TLR6 | na |  |  | 8874 | 0.227 | -0.0309 | No |
| 53 | RPL39 | na |  |  | 8970 | 0.216 | -0.0339 | No |
| 54 | NCK1 | na |  |  | 9082 | 0.204 | -0.0379 | No |
| 55 | INHBA | na |  |  | 10150 | 0.088 | -0.0930 | No |
| 56 | TLR3 | na |  |  | 10160 | 0.087 | -0.0927 | No |
| 57 | HIF1A | na |  |  | 11136 | -0.018 | -0.1436 | No |
| 58 | ACVR2A | na |  |  | 11326 | -0.042 | -0.1531 | No |
| 59 | SOCS5 | na |  |  | 11349 | -0.045 | -0.1539 | No |
| 60 | INHBB | na |  |  | 11465 | -0.058 | -0.1593 | No |
| 61 | CD40 | na |  |  | 11978 | -0.123 | -0.1851 | No |
| 62 | WARS | na |  |  | 12021 | -0.127 | -0.1861 | No |
| 63 | EIF4G3 | na |  |  | 12565 | -0.193 | -0.2128 | No |
| 64 | LIF | na |  |  | 12907 | -0.232 | -0.2285 | No |
| 65 | LY75 | na |  |  | 12984 | -0.242 | -0.2303 | No |
| 66 | HCLS1 | na |  |  | 13062 | -0.251 | -0.2320 | No |
| 67 | IL7 | na |  |  | 13349 | -0.295 | -0.2443 | No |
| 68 | HLA-DQA1 | na |  |  | 13363 | -0.297 | -0.2423 | No |
| 69 | IFNAR2 | na |  |  | 13468 | -0.310 | -0.2449 | No |
| 70 | CAPG | na |  |  | 14567 | -0.458 | -0.2983 | No |
| 71 | IRF7 | na |  |  | 14815 | -0.500 | -0.3066 | No |
| 72 | PRKCG | na |  |  | 15097 | -0.528 | -0.3165 | No |
| 73 | PTPN6 | na |  |  | 15123 | -0.532 | -0.3130 | No |
| 74 | TLR2 | na |  |  | 15128 | -0.535 | -0.3083 | No |
| 75 | CCL2 | na |  |  | 15276 | -0.560 | -0.3109 | No |
| 76 | RIPK2 | na |  |  | 15343 | -0.573 | -0.3091 | No |
| 77 | TNF | na |  |  | 15826 | -0.653 | -0.3284 | No |
| 78 | IL15 | na |  |  | 15840 | -0.654 | -0.3231 | No |
| 79 | ST8SIA4 | na |  |  | 16024 | -0.691 | -0.3264 | No |
| 80 | IKBKB | na |  |  | 16100 | -0.704 | -0.3239 | No |
| 81 | ACHE | na |  |  | 16288 | -0.755 | -0.3268 | No |
| 82 | SOCS1 | na |  |  | 16397 | -0.781 | -0.3253 | No |
| 83 | BCL10 | na |  |  | 16617 | -0.838 | -0.3291 | No |
| 84 | EREG | na |  |  | 16952 | -0.936 | -0.3381 | No |
| 85 | ICOSLG | na |  |  | 17131 | -0.989 | -0.3384 | Yes |
| 86 | IFNGR1 | na |  |  | 17299 | -1.042 | -0.3376 | Yes |
| 87 | ITGB2 | na |  |  | 17342 | -1.055 | -0.3301 | Yes |
| 88 | CCND2 | na |  |  | 17385 | -1.070 | -0.3226 | Yes |
| 89 | TAP1 | na |  |  | 17413 | -1.084 | -0.3141 | Yes |
| 90 | TGFB1 | na |  |  | 17599 | -1.148 | -0.3133 | Yes |
| 91 | TIMP1 | na |  |  | 17600 | -1.148 | -0.3028 | Yes |
| 92 | PSMB10 | na |  |  | 17606 | -1.151 | -0.2925 | Yes |
| 93 | TAPBP | na |  |  | 17642 | -1.164 | -0.2837 | Yes |
| 94 | CRTAM | na |  |  | 17700 | -1.186 | -0.2758 | Yes |
| 95 | CFP | na |  |  | 17862 | -1.258 | -0.2728 | Yes |
| 96 | ICAM1 | na |  |  | 17954 | -1.306 | -0.2656 | Yes |
| 97 | HLA-E | na |  |  | 18047 | -1.349 | -0.2581 | Yes |
| 98 | BCL3 | na |  |  | 18068 | -1.364 | -0.2467 | Yes |
| 99 | HLA-G | na |  |  | 18076 | -1.368 | -0.2345 | Yes |
| 100 | IL4R | na |  |  | 18107 | -1.380 | -0.2235 | Yes |
| 101 | APBB1 | na |  |  | 18115 | -1.382 | -0.2112 | Yes |
| 102 | IL1B | na |  |  | 18217 | -1.433 | -0.2034 | Yes |
| 103 | CD74 | na |  |  | 18418 | -1.591 | -0.1994 | Yes |
| 104 | IFNGR2 | na |  |  | 18421 | -1.594 | -0.1849 | Yes |
| 105 | UBE2D1 | na |  |  | 18424 | -1.599 | -0.1704 | Yes |
| 106 | CD47 | na |  |  | 18478 | -1.626 | -0.1583 | Yes |
| 107 | HLA-DMB | na |  |  | 18502 | -1.645 | -0.1445 | Yes |
| 108 | B2M | na |  |  | 18622 | -1.775 | -0.1345 | Yes |
| 109 | HLA-DOA | na |  |  | 18684 | -1.870 | -0.1206 | Yes |
| 110 | LTB | na |  |  | 18739 | -1.948 | -0.1056 | Yes |
| 111 | HLA-DMA | na |  |  | 18758 | -1.979 | -0.0885 | Yes |
| 112 | IL6 | na |  |  | 18776 | -2.020 | -0.0709 | Yes |
| 113 | C2 | na |  |  | 18918 | -2.345 | -0.0568 | Yes |
| 114 | GBP2 | na |  |  | 18974 | -2.541 | -0.0365 | Yes |
| 115 | CTSS | na |  |  | 18977 | -2.550 | -0.0133 | Yes |
| 116 | HLA-DRA | na |  |  | 19014 | -2.714 | 0.0096 | Yes |
Table: GSEA details [plain text format]

  

Fig 2: HALLMARK\_ALLOGRAFT\_REJECTION      
 Blue-Pink O' Gram in the Space of the Analyzed GeneSet

  

Fig 3: HALLMARK\_ALLOGRAFT\_REJECTION: Random ES distribution      
 Gene set null distribution of ES for **HALLMARK\_ALLOGRAFT\_REJECTION**

  
