# Supplementary material for: Heparin-based hydrogel scaffolding alters the transcriptomic profile and increases the chemoresistance of MDA-MB-231 triple-negative breast cancer cells
Source: Biomater Sci. 2020 Feb 13;8(10):2786–96. doi: 10.1039/c9bm01481k (PMC7497406; doi:10.1039/c9bm01481k)
Supplement: Supplementary file 2 [file BM-008-C9BM01481K-s002.zip › Supplementary File 4/EGFvControl/Pathways/my_analysis.Gsea.1545200981068/HALLMARK_COAGULATION.html]

Details for gene set HALLMARK\_COAGULATION[GSEA]

|  || Dataset | expr.class.cls#EGF\_versus\_CONTROL.class.cls#EGF\_versus\_CONTROL\_repos |
| Phenotype | class.cls#EGF\_versus\_CONTROL\_repos |
| Upregulated in class | CONTROL |
| GeneSet | HALLMARK\_COAGULATION |
| Enrichment Score (ES) | -0.6006292 |
| Normalized Enrichment Score (NES) | -2.6072645 |
| Nominal p-value | 0.0 |
| FDR q-value | 0.0 |
| FWER p-Value | 0.0 |
Table: GSEA Results Summary

  

Fig 1: Enrichment plot: HALLMARK\_COAGULATION      
 Profile of the Running ES Score & Positions of GeneSet Members on the Rank Ordered List

  

| PROBE | DESCRIPTION (from dataset) | GENE SYMBOL | GENE\_TITLE | RANK IN GENE LIST | RANK METRIC SCORE | RUNNING ES | CORE ENRICHMENT || 1 | PLAU | na |  |  | 387 | 1.854 | -0.0002 | No |
| 2 | F3 | na |  |  | 545 | 1.742 | 0.0105 | No |
| 3 | DUSP14 | na |  |  | 563 | 1.729 | 0.0283 | No |
| 4 | FBN1 | na |  |  | 1368 | 1.388 | 0.0012 | No |
| 5 | SERPINB2 | na |  |  | 2053 | 1.207 | -0.0215 | No |
| 6 | GNG12 | na |  |  | 2585 | 1.096 | -0.0374 | No |
| 7 | F2RL2 | na |  |  | 3255 | 0.977 | -0.0618 | No |
| 8 | ARF4 | na |  |  | 3968 | 0.856 | -0.0898 | No |
| 9 | S100A1 | na |  |  | 4847 | 0.723 | -0.1280 | No |
| 10 | ANXA1 | na |  |  | 5268 | 0.669 | -0.1427 | No |
| 11 | WDR1 | na |  |  | 5355 | 0.657 | -0.1401 | No |
| 12 | ADAM9 | na |  |  | 5789 | 0.598 | -0.1563 | No |
| 13 | MASP2 | na |  |  | 6415 | 0.512 | -0.1834 | No |
| 14 | FURIN | na |  |  | 6470 | 0.506 | -0.1808 | No |
| 15 | PEF1 | na |  |  | 6964 | 0.449 | -0.2017 | No |
| 16 | SIRT2 | na |  |  | 7355 | 0.401 | -0.2178 | No |
| 17 | CTSE | na |  |  | 7387 | 0.398 | -0.2151 | No |
| 18 | CTSK | na |  |  | 7786 | 0.351 | -0.2321 | No |
| 19 | MMP3 | na |  |  | 7932 | 0.334 | -0.2361 | No |
| 20 | CTSH | na |  |  | 9066 | 0.206 | -0.2931 | No |
| 21 | F8 | na |  |  | 9146 | 0.197 | -0.2951 | No |
| 22 | RAC1 | na |  |  | 9267 | 0.186 | -0.2994 | No |
| 23 | PREP | na |  |  | 10004 | 0.106 | -0.3368 | No |
| 24 | LGMN | na |  |  | 10233 | 0.077 | -0.3479 | No |
| 25 | ACOX2 | na |  |  | 10488 | 0.054 | -0.3606 | No |
| 26 | LAMP2 | na |  |  | 11032 | -0.007 | -0.3889 | No |
| 27 | RABIF | na |  |  | 11147 | -0.020 | -0.3947 | No |
| 28 | CFH | na |  |  | 11540 | -0.062 | -0.4145 | No |
| 29 | CSRP1 | na |  |  | 11580 | -0.067 | -0.4158 | No |
| 30 | GNB2 | na |  |  | 11716 | -0.089 | -0.4219 | No |
| 31 | SPARC | na |  |  | 12049 | -0.132 | -0.4379 | No |
| 32 | PRSS23 | na |  |  | 12321 | -0.157 | -0.4503 | No |
| 33 | TMPRSS6 | na |  |  | 13328 | -0.292 | -0.4998 | No |
| 34 | PLG | na |  |  | 13548 | -0.322 | -0.5078 | No |
| 35 | LTA4H | na |  |  | 13613 | -0.333 | -0.5075 | No |
| 36 | CASP9 | na |  |  | 13830 | -0.355 | -0.5150 | No |
| 37 | ISCU | na |  |  | 13836 | -0.357 | -0.5114 | No |
| 38 | MMP15 | na |  |  | 13999 | -0.378 | -0.5158 | No |
| 39 | PDGFB | na |  |  | 14224 | -0.409 | -0.5231 | No |
| 40 | F12 | na |  |  | 14282 | -0.419 | -0.5215 | No |
| 41 | MST1 | na |  |  | 14350 | -0.426 | -0.5204 | No |
| 42 | DPP4 | na |  |  | 14594 | -0.462 | -0.5281 | No |
| 43 | ANG | na |  |  | 15384 | -0.579 | -0.5631 | No |
| 44 | FYN | na |  |  | 15435 | -0.588 | -0.5594 | No |
| 45 | USP11 | na |  |  | 16082 | -0.700 | -0.5856 | No |
| 46 | APOC1 | na |  |  | 16366 | -0.773 | -0.5921 | Yes |
| 47 | CRIP2 | na |  |  | 16445 | -0.795 | -0.5875 | Yes |
| 48 | THBD | na |  |  | 16696 | -0.856 | -0.5914 | Yes |
| 49 | MAFF | na |  |  | 16697 | -0.856 | -0.5821 | Yes |
| 50 | GP1BA | na |  |  | 16758 | -0.872 | -0.5758 | Yes |
| 51 | SH2B2 | na |  |  | 16896 | -0.920 | -0.5730 | Yes |
| 52 | MMP1 | na |  |  | 16900 | -0.921 | -0.5632 | Yes |
| 53 | RGN | na |  |  | 17446 | -1.101 | -0.5798 | Yes |
| 54 | CAPN2 | na |  |  | 17466 | -1.111 | -0.5688 | Yes |
| 55 | C1R | na |  |  | 17490 | -1.119 | -0.5579 | Yes |
| 56 | HTRA1 | na |  |  | 17558 | -1.135 | -0.5491 | Yes |
| 57 | S100A13 | na |  |  | 17565 | -1.138 | -0.5371 | Yes |
| 58 | TIMP1 | na |  |  | 17600 | -1.148 | -0.5264 | Yes |
| 59 | C3 | na |  |  | 17617 | -1.156 | -0.5148 | Yes |
| 60 | CFB | na |  |  | 17623 | -1.158 | -0.5025 | Yes |
| 61 | APOA1 | na |  |  | 17673 | -1.176 | -0.4923 | Yes |
| 62 | MMP11 | na |  |  | 17836 | -1.248 | -0.4873 | Yes |
| 63 | TFPI2 | na |  |  | 17846 | -1.252 | -0.4742 | Yes |
| 64 | RAPGEF3 | na |  |  | 18003 | -1.331 | -0.4680 | Yes |
| 65 | CAPN5 | na |  |  | 18247 | -1.455 | -0.4649 | Yes |
| 66 | KLF7 | na |  |  | 18264 | -1.466 | -0.4499 | Yes |
| 67 | GSN | na |  |  | 18275 | -1.477 | -0.4345 | Yes |
| 68 | LRP1 | na |  |  | 18393 | -1.561 | -0.4237 | Yes |
| 69 | SERPINA1 | na |  |  | 18420 | -1.593 | -0.4078 | Yes |
| 70 | CD9 | na |  |  | 18486 | -1.630 | -0.3936 | Yes |
| 71 | CLU | na |  |  | 18498 | -1.641 | -0.3764 | Yes |
| 72 | A2M | na |  |  | 18524 | -1.668 | -0.3596 | Yes |
| 73 | CFD | na |  |  | 18540 | -1.683 | -0.3422 | Yes |
| 74 | DUSP6 | na |  |  | 18544 | -1.690 | -0.3241 | Yes |
| 75 | MSRB2 | na |  |  | 18621 | -1.773 | -0.3089 | Yes |
| 76 | PLAT | na |  |  | 18660 | -1.832 | -0.2910 | Yes |
| 77 | CTSB | na |  |  | 18701 | -1.896 | -0.2726 | Yes |
| 78 | CTSO | na |  |  | 18747 | -1.958 | -0.2538 | Yes |
| 79 | ITGA2 | na |  |  | 18843 | -2.164 | -0.2353 | Yes |
| 80 | TIMP3 | na |  |  | 18862 | -2.233 | -0.2121 | Yes |
| 81 | C1S | na |  |  | 18893 | -2.301 | -0.1888 | Yes |
| 82 | BMP1 | na |  |  | 18897 | -2.311 | -0.1639 | Yes |
| 83 | C2 | na |  |  | 18918 | -2.345 | -0.1396 | Yes |
| 84 | ITGB3 | na |  |  | 18931 | -2.380 | -0.1144 | Yes |
| 85 | PROS1 | na |  |  | 19137 | -3.498 | -0.0873 | Yes |
| 86 | PECAM1 | na |  |  | 19167 | -4.033 | -0.0452 | Yes |
| 87 | CFI | na |  |  | 19177 | -4.319 | 0.0011 | Yes |
Table: GSEA details [plain text format]

  

Fig 2: HALLMARK\_COAGULATION      
 Blue-Pink O' Gram in the Space of the Analyzed GeneSet

  

Fig 3: HALLMARK\_COAGULATION: Random ES distribution      
 Gene set null distribution of ES for **HALLMARK\_COAGULATION**

  
