# Supplementary material for: Heparin-based hydrogel scaffolding alters the transcriptomic profile and increases the chemoresistance of MDA-MB-231 triple-negative breast cancer cells
Source: Biomater Sci. 2020 Feb 13;8(10):2786–96. doi: 10.1039/c9bm01481k (PMC7497406; doi:10.1039/c9bm01481k)
Supplement: Supplementary file 2 [file BM-008-C9BM01481K-s002.zip › Supplementary File 4/EGFvControl/Pathways/my_analysis.Gsea.1545200981068/HALLMARK_KRAS_SIGNALING_DN.html]

Details for gene set HALLMARK\_KRAS\_SIGNALING\_DN[GSEA]

|  || Dataset | expr.class.cls#EGF\_versus\_CONTROL.class.cls#EGF\_versus\_CONTROL\_repos |
| Phenotype | class.cls#EGF\_versus\_CONTROL\_repos |
| Upregulated in class | CONTROL |
| GeneSet | HALLMARK\_KRAS\_SIGNALING\_DN |
| Enrichment Score (ES) | -0.3508187 |
| Normalized Enrichment Score (NES) | -1.505055 |
| Nominal p-value | 0.015345269 |
| FDR q-value | 0.018379506 |
| FWER p-Value | 0.206 |
Table: GSEA Results Summary

  

Fig 1: Enrichment plot: HALLMARK\_KRAS\_SIGNALING\_DN      
 Profile of the Running ES Score & Positions of GeneSet Members on the Rank Ordered List

  

| PROBE | DESCRIPTION (from dataset) | GENE SYMBOL | GENE\_TITLE | RANK IN GENE LIST | RANK METRIC SCORE | RUNNING ES | CORE ENRICHMENT || 1 | EDN1 | na |  |  | 77 | 2.466 | 0.0317 | No |
| 2 | CCNA1 | na |  |  | 344 | 1.897 | 0.0453 | No |
| 3 | DTNB | na |  |  | 516 | 1.762 | 0.0618 | No |
| 4 | SLC16A7 | na |  |  | 1140 | 1.465 | 0.0505 | No |
| 5 | THRB | na |  |  | 1217 | 1.435 | 0.0673 | No |
| 6 | SYNPO | na |  |  | 1629 | 1.311 | 0.0648 | No |
| 7 | ENTPD7 | na |  |  | 1950 | 1.235 | 0.0659 | No |
| 8 | BARD1 | na |  |  | 2041 | 1.211 | 0.0787 | No |
| 9 | SERPINB2 | na |  |  | 2053 | 1.207 | 0.0956 | No |
| 10 | GTF3C5 | na |  |  | 2734 | 1.071 | 0.0756 | No |
| 11 | EPHA5 | na |  |  | 3211 | 0.984 | 0.0649 | No |
| 12 | CDKAL1 | na |  |  | 3276 | 0.973 | 0.0757 | No |
| 13 | RSAD2 | na |  |  | 3298 | 0.971 | 0.0886 | No |
| 14 | HTR1D | na |  |  | 3523 | 0.933 | 0.0904 | No |
| 15 | TFCP2L1 | na |  |  | 4124 | 0.832 | 0.0711 | No |
| 16 | TGM1 | na |  |  | 4424 | 0.788 | 0.0669 | No |
| 17 | IFI44L | na |  |  | 4958 | 0.713 | 0.0493 | No |
| 18 | SKIL | na |  |  | 5109 | 0.691 | 0.0515 | No |
| 19 | P2RX6 | na |  |  | 5316 | 0.663 | 0.0503 | No |
| 20 | GPR19 | na |  |  | 5471 | 0.642 | 0.0515 | No |
| 21 | MSH5 | na |  |  | 5772 | 0.600 | 0.0445 | No |
| 22 | EGF | na |  |  | 6597 | 0.493 | 0.0086 | No |
| 23 | C5 | na |  |  | 6610 | 0.490 | 0.0150 | No |
| 24 | BMPR1B | na |  |  | 7163 | 0.426 | -0.0077 | No |
| 25 | NRIP2 | na |  |  | 7504 | 0.385 | -0.0199 | No |
| 26 | CPEB3 | na |  |  | 7983 | 0.327 | -0.0402 | No |
| 27 | TGFB2 | na |  |  | 8117 | 0.315 | -0.0426 | No |
| 28 | EFHD1 | na |  |  | 8368 | 0.286 | -0.0515 | No |
| 29 | KCND1 | na |  |  | 8604 | 0.261 | -0.0600 | No |
| 30 | RIBC2 | na |  |  | 8916 | 0.221 | -0.0731 | No |
| 31 | CHST2 | na |  |  | 9739 | 0.132 | -0.1142 | No |
| 32 | GPR3 | na |  |  | 9832 | 0.123 | -0.1172 | No |
| 33 | SPTBN2 | na |  |  | 10601 | 0.038 | -0.1568 | No |
| 34 | H2AFY2 | na |  |  | 10654 | 0.031 | -0.1591 | No |
| 35 | GAMT | na |  |  | 10799 | 0.014 | -0.1664 | No |
| 36 | FGGY | na |  |  | 11329 | -0.042 | -0.1935 | No |
| 37 | COPZ2 | na |  |  | 11557 | -0.064 | -0.2045 | No |
| 38 | ITGB1BP2 | na |  |  | 11689 | -0.083 | -0.2101 | No |
| 39 | ABCG4 | na |  |  | 11727 | -0.090 | -0.2107 | No |
| 40 | SPHK2 | na |  |  | 11799 | -0.098 | -0.2130 | No |
| 41 | BTG2 | na |  |  | 11938 | -0.118 | -0.2186 | No |
| 42 | MFSD6 | na |  |  | 12169 | -0.140 | -0.2286 | No |
| 43 | TAS2R4 | na |  |  | 12411 | -0.172 | -0.2387 | No |
| 44 | HSD11B2 | na |  |  | 12481 | -0.181 | -0.2397 | No |
| 45 | STAG3 | na |  |  | 12518 | -0.187 | -0.2389 | No |
| 46 | SLC29A3 | na |  |  | 12724 | -0.218 | -0.2464 | No |
| 47 | DLK2 | na |  |  | 12916 | -0.234 | -0.2530 | No |
| 48 | FGFR3 | na |  |  | 12921 | -0.235 | -0.2498 | No |
| 49 | MX1 | na |  |  | 13486 | -0.314 | -0.2748 | No |
| 50 | PDE6B | na |  |  | 13571 | -0.326 | -0.2745 | No |
| 51 | CACNA1F | na |  |  | 14009 | -0.379 | -0.2918 | No |
| 52 | NR6A1 | na |  |  | 14115 | -0.394 | -0.2916 | No |
| 53 | ATP6V1B1 | na |  |  | 14371 | -0.428 | -0.2988 | No |
| 54 | SHOX2 | na |  |  | 14531 | -0.448 | -0.3006 | No |
| 55 | SGK1 | na |  |  | 14627 | -0.466 | -0.2988 | No |
| 56 | UPK3B | na |  |  | 15175 | -0.542 | -0.3196 | No |
| 57 | CHRNG | na |  |  | 15234 | -0.552 | -0.3146 | No |
| 58 | PRODH | na |  |  | 15336 | -0.571 | -0.3116 | No |
| 59 | EDN2 | na |  |  | 16086 | -0.701 | -0.3407 | Yes |
| 60 | CELSR2 | na |  |  | 16281 | -0.754 | -0.3399 | Yes |
| 61 | CCDC106 | na |  |  | 16332 | -0.764 | -0.3314 | Yes |
| 62 | IDUA | na |  |  | 16415 | -0.787 | -0.3243 | Yes |
| 63 | MAST3 | na |  |  | 16473 | -0.805 | -0.3157 | Yes |
| 64 | GPRC5C | na |  |  | 16500 | -0.811 | -0.3053 | Yes |
| 65 | CLSTN3 | na |  |  | 16577 | -0.830 | -0.2972 | Yes |
| 66 | SNN | na |  |  | 16639 | -0.843 | -0.2882 | Yes |
| 67 | FAM46C | na |  |  | 16752 | -0.870 | -0.2815 | Yes |
| 68 | GP1BA | na |  |  | 16758 | -0.872 | -0.2691 | Yes |
| 69 | SLC25A23 | na |  |  | 17044 | -0.961 | -0.2701 | Yes |
| 70 | KRT15 | na |  |  | 17048 | -0.962 | -0.2563 | Yes |
| 71 | ASB7 | na |  |  | 17128 | -0.988 | -0.2461 | Yes |
| 72 | PTGFR | na |  |  | 17255 | -1.023 | -0.2379 | Yes |
| 73 | PDK2 | na |  |  | 17351 | -1.059 | -0.2275 | Yes |
| 74 | SLC6A14 | na |  |  | 17400 | -1.077 | -0.2145 | Yes |
| 75 | MYO15A | na |  |  | 17474 | -1.113 | -0.2022 | Yes |
| 76 | PTPRJ | na |  |  | 17595 | -1.147 | -0.1918 | Yes |
| 77 | MAGIX | na |  |  | 17730 | -1.194 | -0.1815 | Yes |
| 78 | CAMK1D | na |  |  | 17787 | -1.219 | -0.1668 | Yes |
| 79 | MTHFR | na |  |  | 18103 | -1.379 | -0.1633 | Yes |
| 80 | TCF7L1 | na |  |  | 18199 | -1.424 | -0.1477 | Yes |
| 81 | PLAG1 | na |  |  | 18376 | -1.548 | -0.1344 | Yes |
| 82 | KCNN1 | na |  |  | 18493 | -1.637 | -0.1168 | Yes |
| 83 | LFNG | na |  |  | 18494 | -1.637 | -0.0931 | Yes |
| 84 | NR4A2 | na |  |  | 18503 | -1.645 | -0.0697 | Yes |
| 85 | TFF2 | na |  |  | 18860 | -2.224 | -0.0561 | Yes |
| 86 | YPEL1 | na |  |  | 18907 | -2.335 | -0.0247 | Yes |
| 87 | CYP39A1 | na |  |  | 19023 | -2.750 | 0.0092 | Yes |
Table: GSEA details [plain text format]

  

Fig 2: HALLMARK\_KRAS\_SIGNALING\_DN      
 Blue-Pink O' Gram in the Space of the Analyzed GeneSet

  

Fig 3: HALLMARK\_KRAS\_SIGNALING\_DN: Random ES distribution      
 Gene set null distribution of ES for **HALLMARK\_KRAS\_SIGNALING\_DN**

  
