# Supplementary material for: Heparin-based hydrogel scaffolding alters the transcriptomic profile and increases the chemoresistance of MDA-MB-231 triple-negative breast cancer cells
Source: Biomater Sci. 2020 Feb 13;8(10):2786–96. doi: 10.1039/c9bm01481k (PMC7497406; doi:10.1039/c9bm01481k)
Supplement: Supplementary file 2 [file BM-008-C9BM01481K-s002.zip › Supplementary File 4/EGFvControl/Pathways/my_analysis.Gsea.1545200981068/HALLMARK_ESTROGEN_RESPONSE_LATE.html]

Details for gene set HALLMARK\_ESTROGEN\_RESPONSE\_LATE[GSEA]

|  || Dataset | expr.class.cls#EGF\_versus\_CONTROL.class.cls#EGF\_versus\_CONTROL\_repos |
| Phenotype | class.cls#EGF\_versus\_CONTROL\_repos |
| Upregulated in class | CONTROL |
| GeneSet | HALLMARK\_ESTROGEN\_RESPONSE\_LATE |
| Enrichment Score (ES) | -0.3420837 |
| Normalized Enrichment Score (NES) | -1.6333041 |
| Nominal p-value | 0.0026809652 |
| FDR q-value | 0.008177219 |
| FWER p-Value | 0.082 |
Table: GSEA Results Summary

  

Fig 1: Enrichment plot: HALLMARK\_ESTROGEN\_RESPONSE\_LATE      
 Profile of the Running ES Score & Positions of GeneSet Members on the Rank Ordered List

  

| PROBE | DESCRIPTION (from dataset) | GENE SYMBOL | GENE\_TITLE | RANK IN GENE LIST | RANK METRIC SCORE | RUNNING ES | CORE ENRICHMENT || 1 | HOMER2 | na |  |  | 95 | 2.356 | 0.0093 | No |
| 2 | SLC7A5 | na |  |  | 213 | 2.074 | 0.0158 | No |
| 3 | SNX10 | na |  |  | 237 | 2.043 | 0.0270 | No |
| 4 | CCNA1 | na |  |  | 344 | 1.897 | 0.0329 | No |
| 5 | PRLR | na |  |  | 347 | 1.892 | 0.0443 | No |
| 6 | CDC6 | na |  |  | 472 | 1.786 | 0.0486 | No |
| 7 | SLC26A2 | na |  |  | 537 | 1.748 | 0.0559 | No |
| 8 | CDC20 | na |  |  | 590 | 1.712 | 0.0636 | No |
| 9 | GINS2 | na |  |  | 593 | 1.709 | 0.0738 | No |
| 10 | PLK4 | na |  |  | 650 | 1.670 | 0.0810 | No |
| 11 | SCARB1 | na |  |  | 794 | 1.593 | 0.0832 | No |
| 12 | SLC22A5 | na |  |  | 839 | 1.573 | 0.0904 | No |
| 13 | FKBP4 | na |  |  | 865 | 1.565 | 0.0986 | No |
| 14 | RBBP8 | na |  |  | 927 | 1.541 | 0.1048 | No |
| 15 | FKBP5 | na |  |  | 1120 | 1.472 | 0.1036 | No |
| 16 | HSPA4L | na |  |  | 1129 | 1.468 | 0.1121 | No |
| 17 | BCL2 | na |  |  | 1163 | 1.454 | 0.1192 | No |
| 18 | KIF20A | na |  |  | 1348 | 1.393 | 0.1180 | No |
| 19 | FABP5 | na |  |  | 1404 | 1.376 | 0.1235 | No |
| 20 | MOCS2 | na |  |  | 1459 | 1.359 | 0.1289 | No |
| 21 | PPIF | na |  |  | 1496 | 1.347 | 0.1352 | No |
| 22 | CAV1 | na |  |  | 1542 | 1.335 | 0.1409 | No |
| 23 | XRCC3 | na |  |  | 1668 | 1.303 | 0.1423 | No |
| 24 | DHRS2 | na |  |  | 1688 | 1.297 | 0.1491 | No |
| 25 | PAPSS2 | na |  |  | 1742 | 1.283 | 0.1542 | No |
| 26 | SFN | na |  |  | 1900 | 1.246 | 0.1535 | No |
| 27 | ELOVL5 | na |  |  | 1901 | 1.246 | 0.1610 | No |
| 28 | METTL3 | na |  |  | 1947 | 1.236 | 0.1662 | No |
| 29 | SIAH2 | na |  |  | 2015 | 1.216 | 0.1700 | No |
| 30 | TOP2A | na |  |  | 2151 | 1.189 | 0.1702 | No |
| 31 | STIL | na |  |  | 2163 | 1.186 | 0.1768 | No |
| 32 | GLA | na |  |  | 2226 | 1.173 | 0.1807 | No |
| 33 | CCND1 | na |  |  | 2400 | 1.133 | 0.1785 | No |
| 34 | SORD | na |  |  | 2578 | 1.097 | 0.1758 | No |
| 35 | RNASEH2A | na |  |  | 2596 | 1.095 | 0.1816 | No |
| 36 | HSPB8 | na |  |  | 2666 | 1.081 | 0.1845 | No |
| 37 | HPRT1 | na |  |  | 2703 | 1.076 | 0.1892 | No |
| 38 | DNAJC1 | na |  |  | 2956 | 1.034 | 0.1822 | No |
| 39 | FDFT1 | na |  |  | 3116 | 1.004 | 0.1799 | No |
| 40 | MICB | na |  |  | 3420 | 0.950 | 0.1698 | No |
| 41 | RABEP1 | na |  |  | 3446 | 0.946 | 0.1742 | No |
| 42 | CHPT1 | na |  |  | 3549 | 0.928 | 0.1745 | No |
| 43 | MYOF | na |  |  | 3905 | 0.867 | 0.1611 | No |
| 44 | CD44 | na |  |  | 3954 | 0.860 | 0.1638 | No |
| 45 | MYB | na |  |  | 3976 | 0.854 | 0.1679 | No |
| 46 | BTG3 | na |  |  | 3985 | 0.853 | 0.1727 | No |
| 47 | FARP1 | na |  |  | 4232 | 0.817 | 0.1647 | No |
| 48 | PRKAR2B | na |  |  | 4603 | 0.763 | 0.1499 | No |
| 49 | SLC2A8 | na |  |  | 5054 | 0.700 | 0.1305 | No |
| 50 | NXT1 | na |  |  | 5060 | 0.699 | 0.1345 | No |
| 51 | UNC13B | na |  |  | 5301 | 0.665 | 0.1259 | No |
| 52 | ABHD2 | na |  |  | 5583 | 0.627 | 0.1150 | No |
| 53 | ITPK1 | na |  |  | 5790 | 0.598 | 0.1078 | No |
| 54 | TH | na |  |  | 6131 | 0.551 | 0.0933 | No |
| 55 | JAK2 | na |  |  | 6438 | 0.510 | 0.0803 | No |
| 56 | CYP26B1 | na |  |  | 6716 | 0.478 | 0.0686 | No |
| 57 | TSTA3 | na |  |  | 6733 | 0.476 | 0.0707 | No |
| 58 | FRK | na |  |  | 6801 | 0.467 | 0.0700 | No |
| 59 | PDZK1 | na |  |  | 6915 | 0.454 | 0.0668 | No |
| 60 | SLC29A1 | na |  |  | 6979 | 0.449 | 0.0662 | No |
| 61 | ARL3 | na |  |  | 7166 | 0.426 | 0.0591 | No |
| 62 | TFAP2C | na |  |  | 7746 | 0.357 | 0.0308 | No |
| 63 | CDH1 | na |  |  | 8288 | 0.294 | 0.0042 | No |
| 64 | SLC1A4 | na |  |  | 8321 | 0.291 | 0.0043 | No |
| 65 | PDLIM3 | na |  |  | 8401 | 0.284 | 0.0018 | No |
| 66 | RAPGEFL1 | na |  |  | 9332 | 0.180 | -0.0459 | No |
| 67 | TPD52L1 | na |  |  | 9466 | 0.161 | -0.0519 | No |
| 68 | MEST | na |  |  | 10122 | 0.093 | -0.0858 | No |
| 69 | AMFR | na |  |  | 10146 | 0.089 | -0.0864 | No |
| 70 | DCXR | na |  |  | 10151 | 0.088 | -0.0861 | No |
| 71 | ADD3 | na |  |  | 10185 | 0.084 | -0.0873 | No |
| 72 | DHCR7 | na |  |  | 10300 | 0.069 | -0.0929 | No |
| 73 | ACOX2 | na |  |  | 10488 | 0.054 | -0.1024 | No |
| 74 | OPN3 | na |  |  | 10587 | 0.039 | -0.1073 | No |
| 75 | IL17RB | na |  |  | 10623 | 0.035 | -0.1089 | No |
| 76 | MAPT | na |  |  | 11463 | -0.058 | -0.1526 | No |
| 77 | JAK1 | na |  |  | 11550 | -0.063 | -0.1568 | No |
| 78 | AFF1 | na |  |  | 11645 | -0.077 | -0.1612 | No |
| 79 | IL6ST | na |  |  | 11649 | -0.077 | -0.1609 | No |
| 80 | ID2 | na |  |  | 11940 | -0.118 | -0.1754 | No |
| 81 | WFS1 | na |  |  | 11974 | -0.122 | -0.1764 | No |
| 82 | DLG5 | na |  |  | 12042 | -0.131 | -0.1792 | No |
| 83 | PRSS23 | na |  |  | 12321 | -0.157 | -0.1928 | No |
| 84 | ST14 | na |  |  | 12353 | -0.163 | -0.1934 | No |
| 85 | IGSF1 | na |  |  | 12513 | -0.186 | -0.2007 | No |
| 86 | TNNC1 | na |  |  | 12621 | -0.201 | -0.2051 | No |
| 87 | COX6C | na |  |  | 12625 | -0.202 | -0.2040 | No |
| 88 | TST | na |  |  | 12730 | -0.219 | -0.2081 | No |
| 89 | FGFR3 | na |  |  | 12921 | -0.235 | -0.2167 | No |
| 90 | CLIC3 | na |  |  | 12943 | -0.238 | -0.2163 | No |
| 91 | GALE | na |  |  | 12953 | -0.240 | -0.2154 | No |
| 92 | UGDH | na |  |  | 13035 | -0.249 | -0.2181 | No |
| 93 | TJP3 | na |  |  | 13413 | -0.305 | -0.2361 | No |
| 94 | BAG1 | na |  |  | 13638 | -0.338 | -0.2458 | No |
| 95 | BATF | na |  |  | 13925 | -0.367 | -0.2586 | No |
| 96 | NAB2 | na |  |  | 14090 | -0.391 | -0.2648 | No |
| 97 | TOB1 | na |  |  | 14149 | -0.399 | -0.2654 | No |
| 98 | ALDH3A2 | na |  |  | 14178 | -0.402 | -0.2645 | No |
| 99 | BLVRB | na |  |  | 14274 | -0.418 | -0.2669 | No |
| 100 | TSPAN13 | na |  |  | 14288 | -0.419 | -0.2651 | No |
| 101 | FOXC1 | na |  |  | 14625 | -0.465 | -0.2799 | No |
| 102 | SGK1 | na |  |  | 14627 | -0.466 | -0.2771 | No |
| 103 | NCOR2 | na |  |  | 14701 | -0.480 | -0.2780 | No |
| 104 | SLC9A3R1 | na |  |  | 14781 | -0.492 | -0.2792 | No |
| 105 | NMU | na |  |  | 14798 | -0.496 | -0.2770 | No |
| 106 | PTPN6 | na |  |  | 15123 | -0.532 | -0.2908 | No |
| 107 | LLGL2 | na |  |  | 15669 | -0.619 | -0.3157 | No |
| 108 | ETFB | na |  |  | 15698 | -0.626 | -0.3133 | No |
| 109 | XBP1 | na |  |  | 16003 | -0.687 | -0.3251 | No |
| 110 | ST6GALNAC2 | na |  |  | 16255 | -0.746 | -0.3338 | No |
| 111 | CELSR2 | na |  |  | 16281 | -0.754 | -0.3305 | No |
| 112 | FAM102A | na |  |  | 16306 | -0.759 | -0.3272 | No |
| 113 | KLF4 | na |  |  | 16438 | -0.793 | -0.3292 | No |
| 114 | CKB | na |  |  | 16470 | -0.804 | -0.3260 | No |
| 115 | TRIM29 | na |  |  | 16652 | -0.846 | -0.3303 | No |
| 116 | RPS6KA2 | na |  |  | 16764 | -0.874 | -0.3309 | No |
| 117 | EGR3 | na |  |  | 16779 | -0.878 | -0.3263 | No |
| 118 | DYNLT3 | na |  |  | 17081 | -0.972 | -0.3362 | Yes |
| 119 | PKP3 | na |  |  | 17086 | -0.974 | -0.3305 | Yes |
| 120 | ALDH3B1 | na |  |  | 17123 | -0.985 | -0.3264 | Yes |
| 121 | ATP2B4 | na |  |  | 17125 | -0.987 | -0.3204 | Yes |
| 122 | PERP | na |  |  | 17157 | -0.998 | -0.3160 | Yes |
| 123 | ABCA3 | na |  |  | 17487 | -1.118 | -0.3265 | Yes |
| 124 | NRIP1 | na |  |  | 17528 | -1.131 | -0.3217 | Yes |
| 125 | IMPA2 | na |  |  | 17564 | -1.138 | -0.3167 | Yes |
| 126 | ASS1 | na |  |  | 17575 | -1.141 | -0.3103 | Yes |
| 127 | PLA2G16 | na |  |  | 17607 | -1.151 | -0.3049 | Yes |
| 128 | SERPINA5 | na |  |  | 17678 | -1.177 | -0.3014 | Yes |
| 129 | FOS | na |  |  | 17698 | -1.185 | -0.2952 | Yes |
| 130 | NBL1 | na |  |  | 17771 | -1.213 | -0.2917 | Yes |
| 131 | IDH2 | na |  |  | 17795 | -1.224 | -0.2854 | Yes |
| 132 | TFPI2 | na |  |  | 17846 | -1.252 | -0.2805 | Yes |
| 133 | EMP2 | na |  |  | 17852 | -1.255 | -0.2731 | Yes |
| 134 | PTGES | na |  |  | 17960 | -1.311 | -0.2708 | Yes |
| 135 | MAPK13 | na |  |  | 17998 | -1.329 | -0.2646 | Yes |
| 136 | LSR | na |  |  | 18058 | -1.356 | -0.2595 | Yes |
| 137 | CACNA2D2 | na |  |  | 18089 | -1.376 | -0.2527 | Yes |
| 138 | ZFP36 | na |  |  | 18138 | -1.393 | -0.2468 | Yes |
| 139 | KRT19 | na |  |  | 18142 | -1.395 | -0.2385 | Yes |
| 140 | CPE | na |  |  | 18145 | -1.396 | -0.2301 | Yes |
| 141 | SEMA3B | na |  |  | 18153 | -1.402 | -0.2220 | Yes |
| 142 | CISH | na |  |  | 18183 | -1.416 | -0.2149 | Yes |
| 143 | TMPRSS3 | na |  |  | 18244 | -1.453 | -0.2092 | Yes |
| 144 | SERPINA1 | na |  |  | 18420 | -1.593 | -0.2087 | Yes |
| 145 | HR | na |  |  | 18450 | -1.613 | -0.2005 | Yes |
| 146 | CD9 | na |  |  | 18486 | -1.630 | -0.1924 | Yes |
| 147 | ISG20 | na |  |  | 18505 | -1.647 | -0.1833 | Yes |
| 148 | TFF1 | na |  |  | 18525 | -1.668 | -0.1742 | Yes |
| 149 | TIAM1 | na |  |  | 18560 | -1.701 | -0.1657 | Yes |
| 150 | AGR2 | na |  |  | 18642 | -1.805 | -0.1590 | Yes |
| 151 | TPBG | na |  |  | 18842 | -2.163 | -0.1563 | Yes |
| 152 | SCNN1A | na |  |  | 18942 | -2.414 | -0.1468 | Yes |
| 153 | RAB31 | na |  |  | 18945 | -2.421 | -0.1322 | Yes |
| 154 | AREG | na |  |  | 18983 | -2.586 | -0.1185 | Yes |
| 155 | LAMC2 | na |  |  | 18985 | -2.588 | -0.1028 | Yes |
| 156 | PDCD4 | na |  |  | 18987 | -2.595 | -0.0871 | Yes |
| 157 | WISP2 | na |  |  | 19038 | -2.829 | -0.0725 | Yes |
| 158 | CA12 | na |  |  | 19077 | -2.973 | -0.0565 | Yes |
| 159 | MDK | na |  |  | 19103 | -3.166 | -0.0386 | Yes |
| 160 | ANXA9 | na |  |  | 19127 | -3.371 | -0.0193 | Yes |
| 161 | PLXNB1 | na |  |  | 19158 | -3.785 | 0.0021 | Yes |
Table: GSEA details [plain text format]

  

Fig 2: HALLMARK\_ESTROGEN\_RESPONSE\_LATE      
 Blue-Pink O' Gram in the Space of the Analyzed GeneSet

  

Fig 3: HALLMARK\_ESTROGEN\_RESPONSE\_LATE: Random ES distribution      
 Gene set null distribution of ES for **HALLMARK\_ESTROGEN\_RESPONSE\_LATE**

  
