# Supplementary material for: Heparin-based hydrogel scaffolding alters the transcriptomic profile and increases the chemoresistance of MDA-MB-231 triple-negative breast cancer cells
Source: Biomater Sci. 2020 Feb 13;8(10):2786–96. doi: 10.1039/c9bm01481k (PMC7497406; doi:10.1039/c9bm01481k)
Supplement: Supplementary file 3 [file BM-008-C9BM01481K-s003.pdf]

Electronic Supplementary Material (ESI) for Biomaterials Science.
This journal is © The Royal Society of Chemistry 2020

Video 1. Simulation of the diffusion of EGF. COMSOL Multiphysics is used to model the diffusion of EGF in the porous HP-B hydrogel environment. 50 ng/ml of pre-mixed EGF with less than 0.05% free EGF in the gel shows cumulative release over time corresponding the experimental data. The video file gives a visual representation of the minimal release of EGF in the HP-B hydrogel.

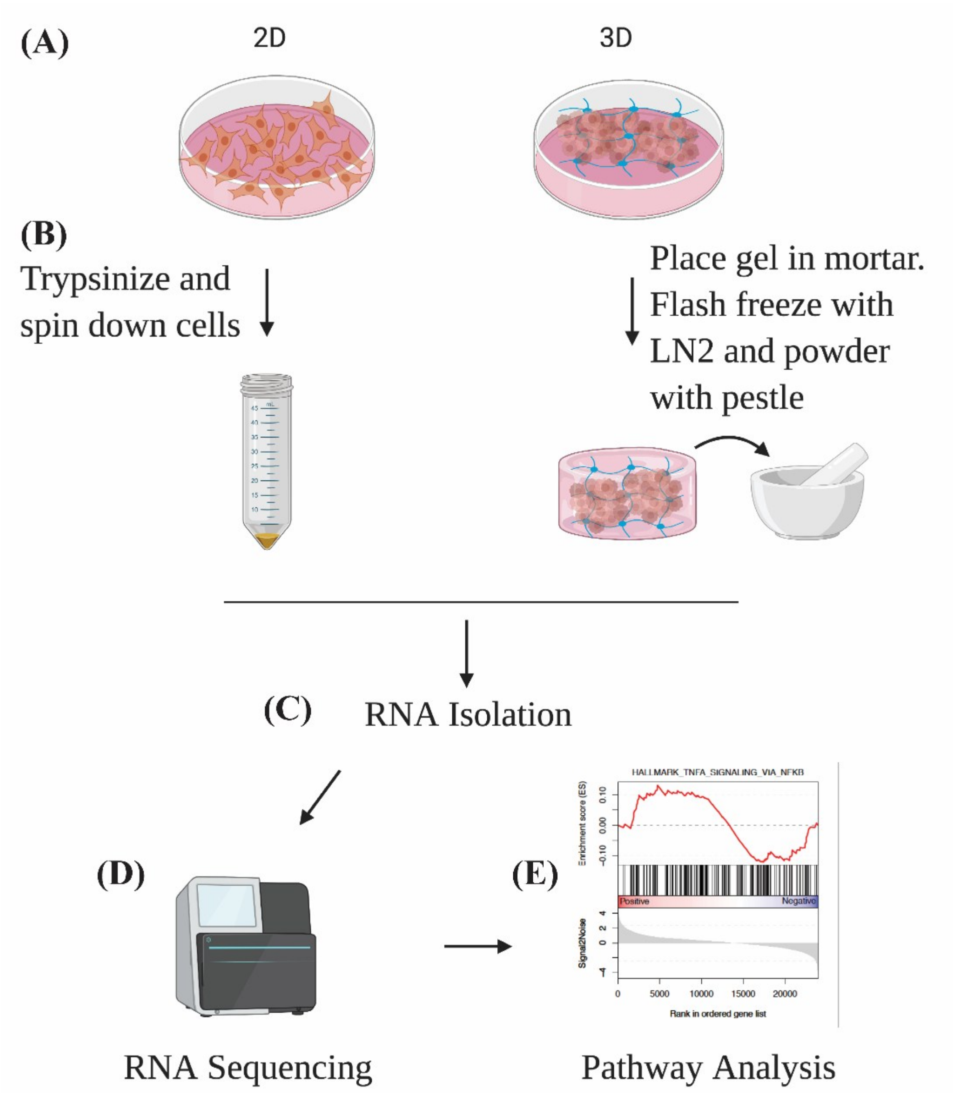

Supplementary Figure 1. RNA seq protocol to quantify transcriptomic differences in 2D and 3D cultures. Cells were grown in their respective microenvironments: (A) Cells in 2D culture include both the control group and the EGF (aq) stimulated group on dish, whereas both hydrogel groups with and without EGF(s) are represented by the 3D culture. (B) Adherent cells grown on the dish were detached using trypsin and spun down to collect the cells. Whereas the gel was retrieved from the wells and transferred to a mortar. Liquid nitrogen (LN2) was used to flash freeze the gel and the encapsulated cells. The pestle was then used to powder the gel. (C) All the samples were processed using Trizol reagent. Chloroform was used for phase separation. RNeasy Mini kit was used to isolate whole RNA. (D) Whole RNA was then sent to the sequencing center. Sequencing was done on the Illumina NextSeq 500/550 High Output kit V2 and the raw data files were obtained. (E) The data was aligned to the genome to quantify gene expression. Further analysis on differential gene expression and hallmark pathways was carried out.

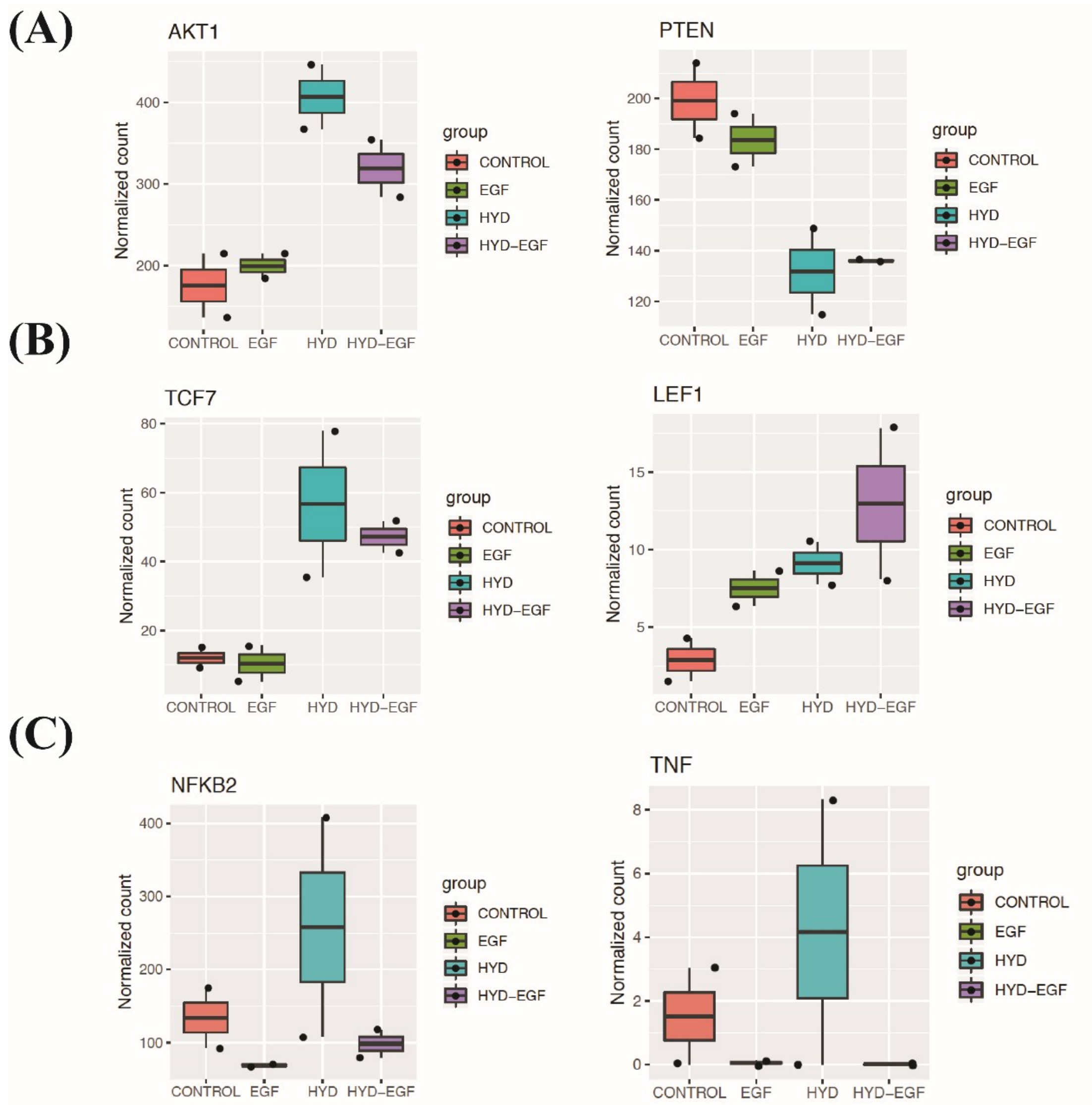

Supplementary Figure 2. Differentially expressed genes regulating critical pathways (A) AKT1 levels in the 3D environments remained high despite overall downregulation of the PI3K-AKT-MTOR signaling. PTEN levels were also lower in 3D environments. **(B)** TCF7 and LEF1 are upregulated in the 3D microenvironment and are key regulators of the canonical Wnt/β-catenin signaling pathway. **(C)** TNF and NF-kB2 are upregulated in conditions without supplemented EGF, both aqueous and solid-phase.

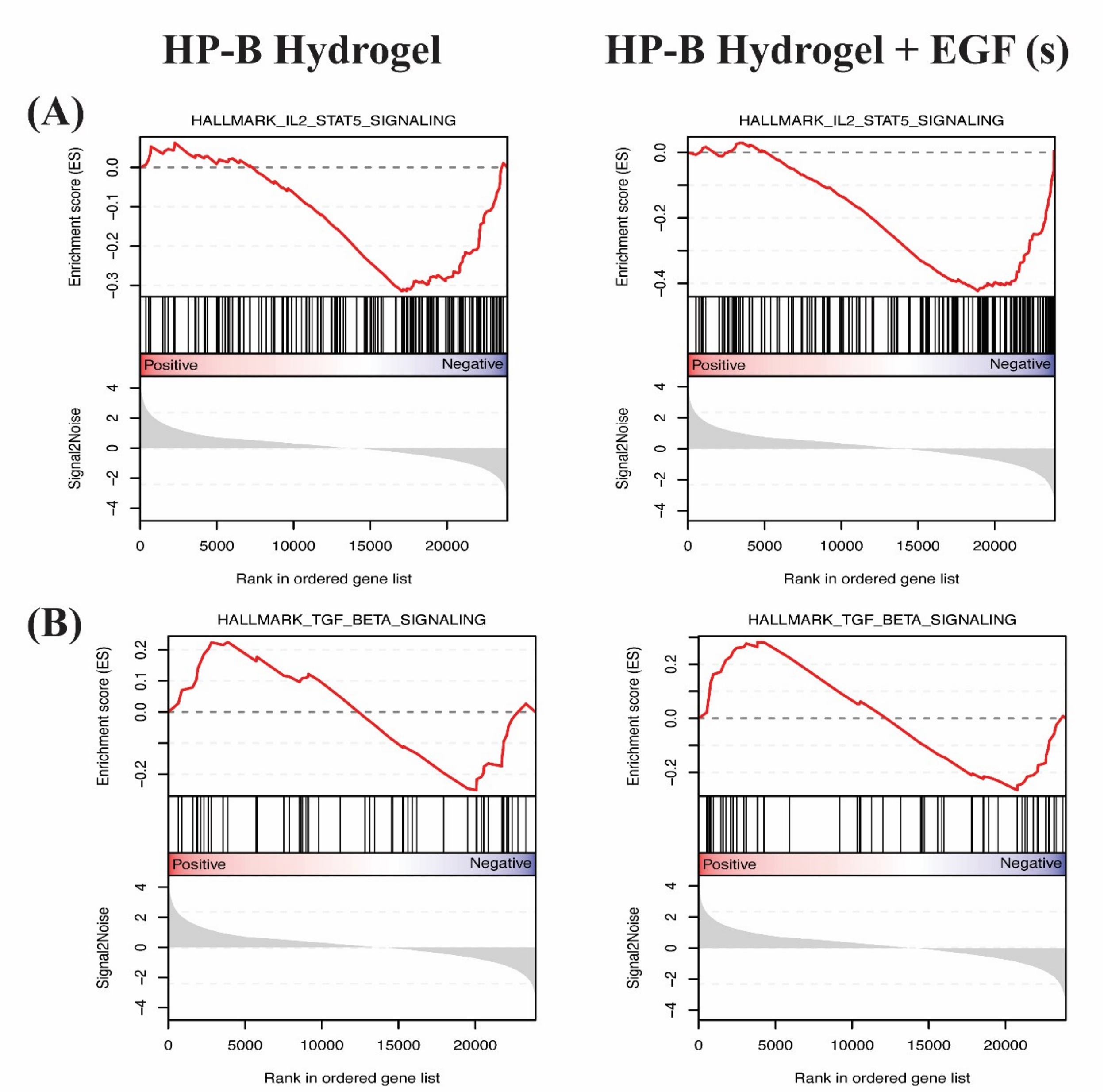

Supplementary Figure 3. Hallmark Pathways involved in immune response regulation. (A) IL2-STAT5 signaling is downregulated in both HP-B hydrogel groups. **(B)** TGF-beta signaling, is upregulated in both HP-B hydrogel environments.
